# Hydrosulfonylation of Alkenes with Sulfonyl Chlorides under Visible Light Activation

**DOI:** 10.1002/anie.202004070

**Published:** 2020-05-07

**Authors:** Sandrine M. Hell, Claudio F. Meyer, Antonio Misale, Jeroen B. I. Sap, Kirsten E. Christensen, Michael C. Willis, Andrés A. Trabanco, Véronique Gouverneur

**Affiliations:** ^1^ University of Oxford Chemistry Research Laboratory 12 Mansfield Road Oxford OX1 3TA UK; ^2^ Discovery Chemistry Janssen Research and Development Jarama 75A 45007 Toledo Spain

**Keywords:** alkenes, hydrosulfonylation, photochemistry, radicals, sulfonyl chlorides

## Abstract

Sulfonyl chlorides are inexpensive reactants extensively explored for functionalization, but never considered for radical hydrosulfonylation of alkenes. Herein, we report that tris(trimethylsilyl)silane is an ideal hydrogen atom donor enabling highly effective photoredox‐catalyzed hydrosulfonylation of electron‐deficient alkenes with sulfonyl chlorides. To increase the generality of this transformation, polarity‐reversal catalysis (PRC) was successfully implemented for alkenes bearing alkyl substituents. This late‐stage functionalization method tolerates a remarkably wide range of functional groups, is operationally simple, scalable, and allows access to building blocks which are important for medicinal chemistry and drug discovery.

## Graphical abstract

**Radical new approach**: Sulfonyl chlorides have been applied in the radical hydrosulfonylation of alkenes. Tris(trimethylsilyl)silane is an ideal hydrogen atom donor enabling highly effective photoredox‐catalyzed hydrosulfonylation of electron‐deficient alkenes with sulfonyl chlorides. To increase the generality of this transformation, polarity‐reversal catalysis was successfully implemented for alkenes bearing alkyl substituents.

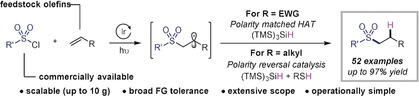

## Introduction

**Scheme 1:**
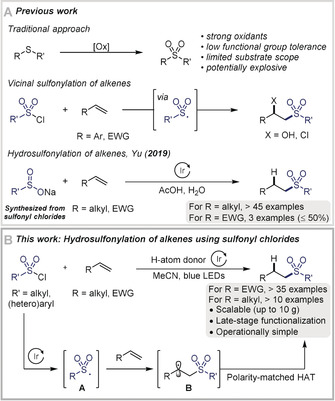
A) Selected routes to functionalized sulfones. B) Photoredox‐catalyzed hydrosulfonylation of alkenes using sulfonyl chlorides (this work).

Sulfones are prevalent motifs in marketed drugs and natural products due to their unique electronic and structural properties.^1^ Today, sulfone‐containing molecules are considered routinely in medicinal chemistry programs searching for anti‐inflammatory, antimicrobial, anticancer, anti‐HIV, and antimalarial activities.^1^ Traditionally, sulfone derivatives are prepared by oxidation of a sulfide or sulfoxide with strong oxidants such as peracids or hydrogen peroxide (Scheme 1 A).^2^ These conditions limit applicability in the context of late‐stage functionalization of complex molecules. Other methods include alkylation of sulfinate salts, Friedel–Crafts type sulfonylation of arenes, and addition reactions to alkenes and alkynes.^3^ Most of these methods focus on the construction of C(sp^2^)‐sulfones. In recent years, the development of radical processes for the formation of C(sp^3^)‐sulfonylated fragments has gained considerable interest.^4^ In these transformations, sulfonyl chlorides, metal sulfinates, sulfonyl selenides, sulfonyl azides, and allyl sulfones have been used as precursors to generate sulfonyl radicals for the functionalization of electron‐rich olefins, styrene derivatives and unactivated alkenes.^5^, ^6^ These procedures typically feature atom‐transfer radical addition (ATRA) leading to vicinal difunctionalized motifs.^6^ Despite these advances, methods enabling direct hydrosulfonylation of alkenes remain underexplored.^7^ Very recently, Yu and co‐workers reported a photoredox‐catalyzed hydrosulfonylation of unactivated alkenes using sulfinate salts as radical source, a class of reagents often synthesized from sulfonyl chlorides.7b This transformation is best suited for alkenes bearing alkyl groups; electron‐deficient alkenes are less reactive and/or give mixture of products (Scheme 1 A).

As part of our ongoing research on photoredox‐catalyzed hydrofunctionalization of alkenes,^8^ this state‐of‐play encouraged us to develop an operationally simple and scalable protocol enabling hydrosulfonylation of a broad range of substrates using commercially and readily available sulfonyl chlorides (Scheme 1 B). Sulfonyl chlorides are suitable for ATRA leading to net chlorosulfonylation,5a, 5c, 5f, 5j, 6a, 6b, 6e, 6h, 6i but re‐routing this process towards hydrosulfonylation of alkenes has not been accomplished. Mechanistically, this process would require the addition of sulfonyl radical **A** to the alkene, generating the C‐centered radical **B**, followed by hydrogen atom transfer (HAT) to afford the hydrosulfonylated product. For successful HAT, a polarity match between radical **B** and the hydrogen atom donor (HAD) is necessary. Therefore, fine‐tuning of the reaction conditions will be necessary for this protocol to be applicable to alkenes of different electronic profiles. Electrophilic radicals would require a nucleophilic HAD for a polarity matched HAT, while an electrophilic HAD would be necessary for nucleophilic radicals.

## Results and Discussion

### Hydrosulfonylation of Electron‐Deficient Alkenes

Processes other than hydrosulfonylation have demonstrated that sulfonyl radical **A** can undergo regioselective Giese‐addition to an electron‐deficient alkene affording electrophilic radical **C** (Table 1).6h, 7 For such a radical, a nucleophilic HAD is required for a polarity‐matched HAT. Sulfonyl radical **A** could also undergo direct H‐atom abstraction to form sulfinic acid **D**, a competitive HAT pathway prompting us to test a range of H‐atom donors. We chose 4‐fluorobenzenesulfonyl chloride as sulfonyl radical source, *N*‐phenylacrylamide (**2 a**) as the model alkene, and *fac*‐Ir(ppy)_3_ as photocatalyst to test the viability of this transformation. Acetonitrile was selected as the reaction solvent.^9^

**Table 1:** Reaction design and optimization of the reaction conditions for the hydrosulfonylation of *N*‐phenylacrylamide.^[a]^ 
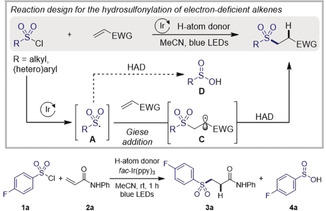

Entry	H‐donor	Yield [%]^[b]^ (**3 a**)	Yield [%]^[b]^ (**4 a**)
1	–	traces	2
2	THF (solvent)	traces	4
3	Ph_3_CH	traces	traces
4	Et_3_SiH	traces	traces
5	Ph_3_SiH	traces	4
**6**	**(TMS)_3_SiH**	**90 (82^[c]^)**	**11**
7	Bu_3_SnH	28	59
8	Hantzsch ester	34	3
9	PhSH	26	3
10^[d]^	(TMS)_3_SiH	0	0
11^[e]^	(TMS)_3_SiH	23	4

[a] Reaction conditions: **1 a** (0.25 mmol), **2 a** (0.1 mmol), *fac*‐Ir(ppy)_3_ (0.5 mol %), H‐atom donor (0.2 mmol), MeCN (0.6 mL) under blue light (*λ*_max_=450 nm) irradiation for 1 h. [b] Yield determined by ^19^F NMR using α,α,α‐trifluorotoluene as internal standard. [c] Yields of isolated products. [d] Reaction performed in the dark. [e] Reaction performed in the absence of photocatalyst.

As expected, the choice of HAD influences the outcome of the reaction (Table 1, entries 1–9). In the absence of HAD, the reaction did not proceed (entry 1). When tetrahydrofuran, triphenylmethane, triethylsilane, or triphenylsilane were used, only traces of desired product **3 a** and sulfinic acid **4 a** were formed (entries 2–5).8b, 8c, 10, 11 With its favorable Si−H bond dissociation energy (BDE=351.5 kJ mol^−1^),^12^ tris(trimethylsilyl)silane enabled access to the hydrosulfonylated product **3 a** in 90 % yield along with 11 % yield of **4 a** (entry 6). The yield was not further improved by using tributyltin hydride, Hantzsch ester, or thiophenol as alternative HAD (entries 7–9). Light irradiation is essential for the reaction to proceed (entry 10). In the absence of photocatalyst, the reaction proceeded with significantly lower yield (entry 11).^13^, ^14^ Further screening of reaction conditions showed that more than one photocatalyst was suitable for this transformation.^9^ However, early experimentation on representative starting materials indicated that *fac*‐Ir(ppy)_3_ gave higher yields with the organophotocatalyst Eosin Y being more often second best.

**Scheme 2:**
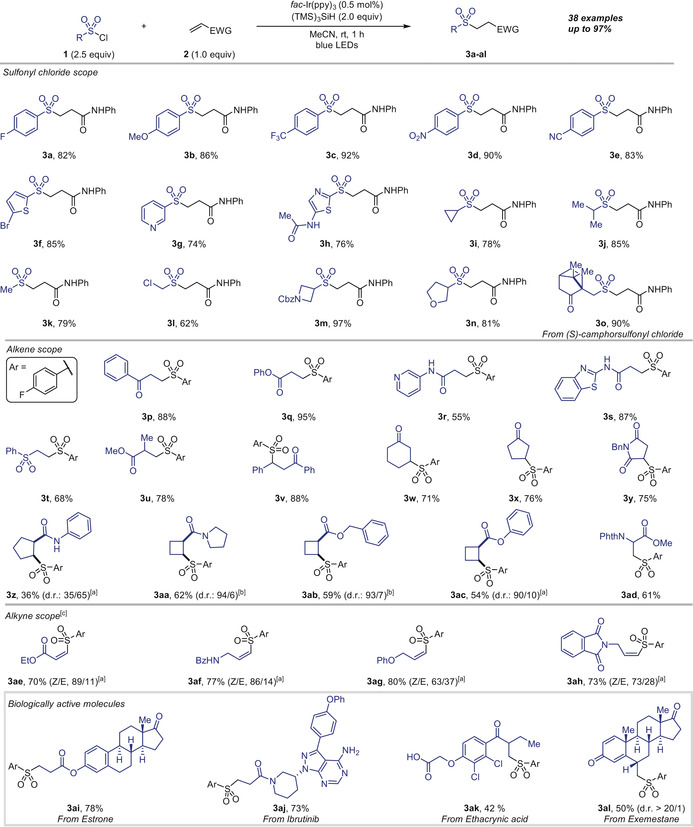
Substrate scope. Reaction conditions: **1 a**–**o** (1.25 mmol), **2 a**–**x** (0.5 mmol), (TMS)_3_SiH (1.0 mmol), *fac*‐Ir(ppy)_3_ (0.5 mol %), MeCN (3.0 mL), blue LED irradiation (*λ*_max_=450 nm), room temperature, 1 h. Yields of isolated products. [a] The isomers were separated by silica flash column chromatography. [b] The minor isomer was not isolated. [c] 16 h reaction time.

The generality of this approach was investigated on a range of sulfonyl chlorides and alkenes (Scheme 2). Methoxy, trifluoromethyl, nitro, nitrile, halide, ketone, ether, amide, and carbamate‐substituted sulfonyl chlorides were all tolerated under the reaction conditions, and the desired hydrosulfonylated products were obtained in moderate to excellent yields (**3 a**–**o**). A broad variety of heteroarylsulfonyl chlorides containing thiophene, thiazole, and pyridine were suitable (**3 f**–**h**). Alkyl sulfonyl chlorides gave access to dialkyl sulfones in good yields (**3 i**–**o**). Heterocycloalkyl sulfonyl chlorides afforded the hydrosulfonylated products in high yields (**3 m**,**n**). Moreover, a camphor derivative was isolated in excellent yield (**3 o**). Linear terminal, *gem*‐disubstituted and 1,2‐disubstituted alkenes gave the desired products in good to excellent yields (**3 p**–**v**). Alkenes substituted with a heteroaryl group, such as a pyridyl or benzothiazyl were converted to the hydrosulfonylated product in moderate to good yield (**3 r**,**s**). A series of cyclic alkenes including cyclohexene, cyclopentene, and cyclobutene provided highly desirable 1,2‐disubstituted building blocks in good yields and with excellent diastereoselectivities (**3 w**–**ac**). Furthermore, the hydrosulfonylation of a dehydroalanine derivative was achieved in 61 % yield (**3 ad**). We also investigated the addition of sulfonyl radicals onto alkynes. Both electron‐deficient and unactivated alkynes led to the corresponding vinyl sulfones(**3 ae**–**3 ah**) in good yields, and moderate to high *Z*‐selectivity. Finally, this procedure was validated on biologically active molecules. Hydrosulfonylated analogues of an Estrone derivative, Ibrutinib, and Ethacrynic acid were isolated in moderate to good yields (**3 ai**–**3 ak**). The hydrosulfonylation of Exemestane afforded the desired product (**3 al**) in moderate yield, excellent diastereoselectivity and regioselectivity for the *exo*‐1,6‐system.

**Scheme 3:**
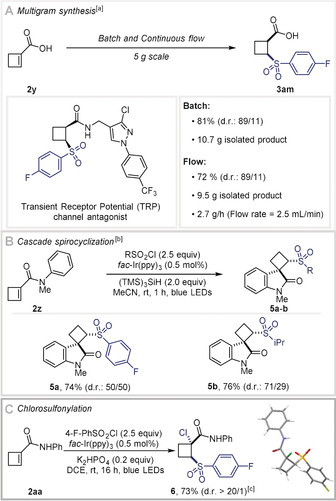
Further applications of the hydrosulfonylation of cyclobutene derivatives. Yields of isolated products. [a] Reaction conditions: see Supporting Information. [b] The diastereoisomers were separated by silica flash column chromatography. [c] Stereochemistry was assigned by single‐crystal X‐ray diffraction analysis.

With the aim of scaling‐up this transformation to multigram, we explored the synthesis of a precursor to a family of Transient Receptor Potential (TRP) channel antagonists (Scheme 3 A).2d Hydrosulfonylation was accomplished on a 5 g scale, affording **3 am** in good yield (10.7 g, 81 %) and diastereoselectivity (89/11). Considering the benefits of performing photochemical reactions in continuous processing, we also optimized the conditions for a continuous flow protocol. A 5 g reaction in a solvent mixture of acetonitrile/*N*,*N*‐dimethylformamide (3:1) was performed in flow with a residence time of 4 min (10 mL reactor volume) allowing access to the desired hydrosulfonylated product **3 am** in good yield (9.5 g, 72 %) and identical level of diastereocontrol with increased productivity (2.7 g h^−1^). Collectively, these results suggest that this methodology could be applicable to large‐scale production in batch or under continuous flow conditions. We also explored the possibility to increase structural complexity through quaternization of the intermediate α‐carbonyl radical derived from an alkene substituted with an *N*‐arylated tertiary amide (Scheme 3 B).

Under the standard reaction conditions, alkene **1 z** led to a cascade spirocyclization to afford cyclobutylspirooxindoles **5 a**,**b**.^15^ Aryl‐ and alkylsulfone‐containing spirocycles could be accessed in good yields and moderate diastereomeric ratio (**5 a**,**b**). When 1,2‐dichloroethane was used as solvent and no HAD was present, the ATRA product was obtained in high yield and excellent diastereoselectivity (**6**, Scheme 3 C).6a, 6b, 6e, 6g, 6h

### Hydrosulfonylation of Other Alkenes

**Scheme 4:**
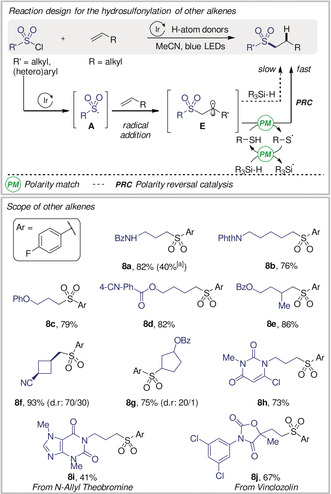
Scope of other alkenes. Reaction conditions: **1 a** (1.25 mmol), **7 a**–**j** (0.5 mmol), (TMS)_3_SiH (1.0 mmol), 4‐mercaptophenol (0.1 mmol), *fac*‐Ir(ppy)_3_ (0.5 mol %), MeCN (3.0 mL), blue LED irradiation (*λ*_max_=450 nm), room temperature, 16 h. Yields of isolated products. [a] Without thiol catalyst.

The hydrosulfonylation of alkenes other than those bearing an electron‐withdrawing group was studied next (Scheme 4).

Preliminary results showed that under our standard reaction conditions, alkene **7 a** afforded the hydrosulfonylated product in only 40 % (**8 a**),^16^ a result possibly stemming from a poor polarity match between the nucleophilic radical intermediate **E** and the nucleophilic HAD (TMS)_3_SiH. We reasoned that a two‐step polarity‐matched process using an H‐atom transfer catalyst could improve the outcome of this reaction (polarity‐reversal catalysis (PRC)^17^). When a catalytic amount of a thiol catalyst (electrophilic HAD) was added to the standard reaction conditions, the yield of **8 a** increased to 82 %. Under these modified conditions, various alkenes (**7 a**–**j**) were tolerated, affording the desired products in high yields (**8 a**–**j**). Terminal and *gem*‐disubstituted alkenes were suitable for this transformation, leading to the desired products in good yields (**8 a**–**f**). The hydrosulfonylation of a cyclopentene derivative gave **8 g** in good yield and excellent diastereomeric ratio. Finally, alkene‐containing biologically active molecules were also suitable substrates, and afforded **8 i** and **8 j** in moderate to good yields.

### Mechanistic Considerations

**Scheme 5:**
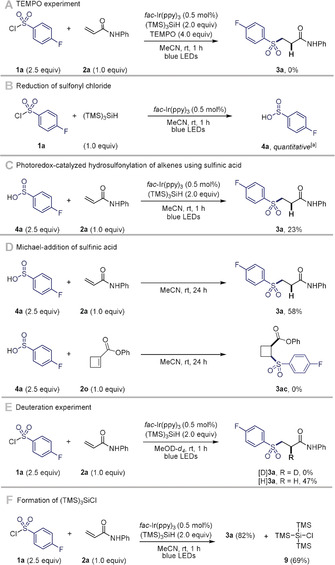
A–F) Mechanistic investigations. Yields of isolated products. [a] Yield determined by ^19^F NMR using α,α,α‐trifluorotoluene as internal standard.

We next turned our attention to the reaction mechanism. Control experiments showed that no hydrosulfonylated product **3 a** derived from the electron‐deficient alkene **2 a** was formed in the presence of the radical scavenger TEMPO (2,2,6,6‐tetramethylpiperidin‐1‐yl)oxyl); **2 a** was fully recovered (Scheme 5 A).^18^ When the reaction was performed in absence of alkene, quantitative reduction of sulfonyl chloride **1 a** to sulfinic acid **4 a** was observed (Scheme 5 B). The use of sulfinic acid **4 a** instead of sulfonyl chloride under our standard reaction conditions afforded **3 a**, albeit in significantly lower yield (Scheme 5 C). Previous reports showed that sulfinic acids readily undergo Michael addition.^19^ However, the efficiency of this addition is highly dependent on the alkene as illustrated with **2 a** and **2 o** (Scheme 5 D). Furthermore, the reaction of **2 a** in MeOD‐*d_4_* did not lead to deuterium incorporation, an observation advocating against the formation of an enol‐type intermediate, and in favor of direct H‐atom transfer between (TMS)_3_SiH and a radical intermediate (Scheme 5 E; selective formation of [H]**3 a** over [D]**3 a**). This deuteration experiment demonstrates that an ionic pathway involving the Michael‐addition of a sulfinic acid can be ruled out under these reaction conditions.^11^ This is further supported by the nature of the spiro‐cyclized product obtained from **2 z** (Scheme 3 B). Furthermore, (TMS)_3_SiCl **9** was isolated as a side‐product of the reaction, which is consistent with the oxidation of the silyl radical (Scheme 5 F).

**Scheme 6:**
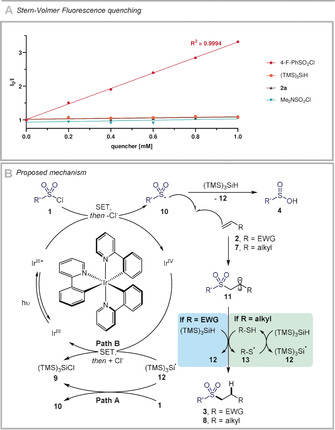
A) Stern–Volmer fluorescence quenching experiments. B) Proposed mechanism for the hydrosulfonylation of alkenes.

Stern–Volmer fluorescence quenching experiments provide additional information (Scheme 6 A). The excited state of *fac*‐Ir(ppy)_3_ is quenched by sulfonyl chloride **1 a**, implying that a direct single‐electron reduction of the sulfonyl chloride, leading to the formation of sulfonyl radical anion is plausible. This reactivity profile contrasts with that of *N*,*N*‐dimethylsulfamoyl chloride.8d Notably, (TMS)_3_SiH does not quench the excited state of *fac*‐Ir(ppy)_3_, advocating against an initiation scenario whereby [Ir^III^*] could undergo single‐electron transfer with tris(trimethylsilyl)silane to generate the Si‐centered radical **12** upon loss of a proton.^20^

Based on these observations, we suggest a plausible mechanism for this transformation (Scheme 6 B). Irradiation with light affords the excited triplet state of the iridium photocatalyst. Single‐electron transfer to sulfonyl chloride **1** renders the sulfonyl radical **10** along with a chloride anion after fragmentation of the initially formed radical anion intermediate. In presence of a nucleophilic HAD such as (TMS)_3_SiH, **10** can react to form **4**, a suitable but less efficient reagent for hydrosulfonylation under our reaction conditions. Alternatively, addition of **10** to the alkene furnishes the C‐centered radical **11**. For electron‐deficient alkenes (R=EWG), the electrophilic radical **11** is trapped by (TMS)_3_SiH to afford the desired hydrosulfonylated product **3** along with the silyl radical **12**. For other alkenes (R=alkyl), the nucleophilic radical **11** could undergo H‐atom transfer with the thiol polarity‐reversal catalyst, generating the desired product **8** and thiyl radical **13**, which can undergo H‐atom abstraction from (TMS)_3_SiH to afford silyl radical **12** along with the regenerated thiol catalyst. The observation that the reaction is possible in the absence of photocatalyst although more effective in its presence (Table 1, entry 11), favours a photoinitiation‐radical chain propagation pathway (Scheme 6, Path A). The regeneration of the iridium photocatalyst in its ground state by the oxidation of **12** is also viable (Path B).

## Conclusion

In summary, we have developed a general photoredox‐catalyzed hydrosulfonylation of alkenes using commercially and readily available sulfonyl chlorides. This methodology allows access to an extensive range of hydrosulfonylated products derived from alkenes with a large spectrum of reactivity. Spirocyclic oxindoles can be obtained in one step by a radical cascade cyclization. This method was applied to the late‐stage sulfonylation of biologically active molecules, and the multigram synthesis of a pharmaceutically relevant building block was demonstrated.

## Conflict of interest

The authors declare no conflict of interest.

## Supporting information

As a service to our authors and readers, this journal provides supporting information supplied by the authors. Such materials are peer reviewed and may be re‐organized for online delivery, but are not copy‐edited or typeset. Technical support issues arising from supporting information (other than missing files) should be addressed to the authors.

Supplementary
