# Supplementary material for: Hydrosulfonylation of Alkenes with Sulfonyl Chlorides under Visible Light Activation
Source: Angew Chem Int Ed Engl. 2020 May 7;59(28):11620–6. doi: 10.1002/anie.202004070 (PMC7384135; doi:10.1002/anie.202004070)
Supplement: Supplementary file 1 — Supplementary [file ANIE-59-11620-s001.pdf]

Angewandte Chemie
Eine Zeitschrift der Gesellschaft Deutscher Chemiker

Supporting Information

Hydrosulfonylation of Alkenes with Sulfonyl Chlorides under Visible Light Activation

*Sandrine M. Hell[+], Claudio F. Meyer[+], Antonio Misale, Jeroen B. I. Sap, Kirsten E. Christensen, Michael C. Willis, Andrés A. Trabanco, and Véronique Gouverneur**

anie_202004070_sm_miscellaneous_information.pdf

Table of contents:

1. General Information **S2**

2. Synthesis of starting materials (**2z**, **2aa, 7d**) **S3**

3. Optimization of the reaction conditions **S6**

4. Mechanistic investigations **S8**

5. Scale-up experiments (**3am**) **S14**

6. General procedures **S18**

7. Characterizations **S19**

8. Determination of the stereochemistry for compounds **3aa**, **3ab**, **3ac**, **5a**, **5b** **S54**

9. References **S55**

10. Spectra **S56**

11. X-ray - Crystallographic Data for **6** **S166**

1. General Information

All NMR spectra were recorded on Bruker DPX-400 or a Bruker Avance II HD spectrometer with standard pulse sequences operating at 400 and 500 MHz, respectively, using $CDCl_3$ or DMSO-d_6 as solvents. ^{1}H and ^{13}C NMR spectral data are reported as chemical shifts (δ) in parts per million (ppm) relative to the solvent peak using the Bruker internal referencing procedure (edlock). ^{19}F NMR spectra are referenced relative to $CFCl_3$ in $CDCl_3$. Coupling constants (J) are measured in hertz (Hz). The following abbreviations are used to describe multiplicities s=singlet, d=doublet, t=triplet, q=quartet, m=multiplet, dd=doublet of doublets, dt=doublet of triplets, td=triplet of doublets, br=broad,. NMR spectra were processed in MestreNova. High resolution mass spectra (HRMS, *m*/*z*) were recorded on a Bruker MicroTOF spectrometer using positive electrospray ionization (ESI). Melting points of solids were measured on a DSC823^e (Differential Scanning Analysis) Mettler Toledo apparatus. IUPAC names were obtained using the ChemDraw service. Weighing was performed with a 4 decimal place balance. All reactions for the hydrosulfonylation of alkenes were conducted in non-dried glassware with magnetic stirring. All solvents were used as received without further purification. $(TMS)_3SiH$ was purchased from Sigma-Aldrich, TCI and Combi-blocks. Photocatalyst *fac*-Ir(ppy)$_3$ was purchased from Sigma-Aldrich. Sulfonyl chlorides were purchased from Sigma-Aldrich, Combi-blocks, TCI and Enamine. Flash column chromatography was performed over silica gel C60 (40−60 μm). All commercially available substrates were purchased from commercial suppliers or otherwise synthesized according to literature.[1-14] Reactions were performed in 7 mL vials with two Kessil LEDSs (35W, 450 nm, approximately 4 cm away from the light source). The yields were determined by isolation on SiO_2 gel column chromatography. The scale-up experiment in batch was performed using a Radleys Reactor Ready of 1 or 2 Liters and the temperature controlled using a Julabo A80 unit. The scale-up experiment in continuous flow was performed in a Vapourtec photoreactor (fluoropolymer tube, 1.3 mm i.d., 10 mL). If the purity of the compounds was not satisfying, an analytical sample was further purified by reverse phase HPLC purification. Low temperature single crystal X-ray diffraction data were collected using a (Rigaku) Oxford Diffraction SuperNova A diffractometer. Data were reduced using CrysAlisPro, solved using SuperFlip[15] and refined using CRYSTALS.[16] Full crystallographic data (in CIF format) is available as ESI and has been deposited with the Cambridge Crystallographic Data Centre (CCDC 1973291).

2. Synthesis of starting materials (2y, 2aa, 7d).

Compounds 2a, **2b**, **2d**, **2e, 2l**, **2m**, **2n**, **2o**, **2u**, **2y, 7a, 7b, 7d, 7e, 7g, 7h** and **7i** were synthesized according to literature.[1-14]

General procedure 1: Preparation of cycloalkene-1-carboxamides (2y, 2aa)

The desired amine (5.5 mmol, 1.1 equiv) was added to a solution of carboxylic acid (5 mmol, 1.0 equiv), HATU (2.1 g, 5.5 mmol, 1.1 equiv), and *N,N*-Diisopropylethylamine (1.75 mL, 10 mmol, 2.0 equiv) in DCM (50 mL). The reaction was stirred for 16 h, after which time the reaction mixture was diluted with DCM (30 mL) and washed with an aqueous solution of HCl (1M, 30 mL) and brine (sat., 30 mL). The organic layer was dried (Na_2SO_4), filtered and concentrated *in vacuo*. The crude product was purified by flash chromatography (silica, EtOAc in Heptane, 0/100 to 40/60). The desired fractions were collected and concentrated *in vacuo* to give the desired amide as a white solid.

N-methyl-*N*-phenylcyclobut-1-ene-1-carboxamide (2z)

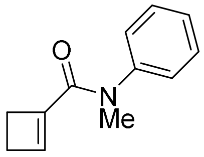

General procedure 1 was followed to obtain **2z** (605 mg, 3.2 mmol, 64%) as a white solid.
^{1}H NMR (400 MHz, $CDCl_3$) δ 7.40 - 7.28 (m, 3H), 7.21 - 7.17 (m, 2H), 5.66 (s, 1H), 3.27 (s, 3H), 2.24 - 2.08 (m, 4H); **^{13}C NMR** (101 MHz, $CDCl_3$) δ 162.8, 143.8, 143.3, 141.4, 129.5, 128.0, 127.9, 37.8, 31.0, 26.8; **HRMS** (ESI-TOF) calculated for $C_{12}H_{14}ON$ $[M+H]^+$: 188.1075; found 188.1075; **m.p.**: 63 - 67 °C; **IR** (neat) 2981, 2360, 1625, 1593, 1574, 1493, 1417, 1379, 1305, 1256, 1169, 1108, 1072, 1032, 1010, 951, 910, 878, 862, 842, 777, 740, 705, 682.

***N*-phenylcyclobut-1-ene-1-carboxamide (2aa)**

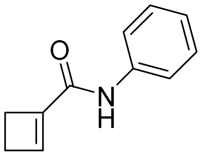

General procedure 1 was followed to obtain **2aa** (424 mg, 2.5 mmol, 49%) as a white solid.
^{1}H NMR (400 MHz, $CDCl_3$) δ 7.61 - 7.54 (m, 2H), 7.46 (br s, 1H), 7.35 - 7.28 (m, 2H), 7.14 - 7.07 (m, 1H), 6.73 (s, 1H), 2.79 (dd, J = 3.1, 2.9 Hz, 2H), 2.51 - 2.47 (m, 2H); **^{13}C NMR** (101 MHz, $CDCl_3$) δ 160.7, 141.9, 141.8, 137.7, 129.1, 124.4, 120.0, 28.8, 26.2; **HRMS** (ESI-TOF) calculated for $C_{11}H_{12}ON$ $[M+H]^+$: 174.0913; found 174.0911; **m.p.**: 139 - 143 °C; **IR (neat)** 2981, 1650, 1599, 1579, 1531, 1440, 1384, 1339, 1323, 1253, 1156, 1117, 1076, 1030, 957, 749, 687, 617.

But-3-en-1-yl 4-cyanobenzoate (7d)

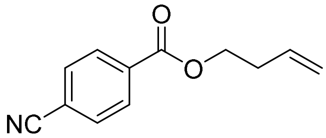

4-Cyanobenzoyl chloride (2.0 g, 12.1 mmol, 1.0 equiv) was added to a stirred solution of 3-buten-1-ol (870 mg, 12.1 mmol, 1.0 equiv) and triethylamine (2.5 mL, 18.1 mmol, 1.5 equiv) in DCM (25 mL) at 0 °C. The reaction mixture was warmed to room temperature and stirred for 72 hours., quenched by dropwise addition of a saturated aqueous solution of NH_4Cl and extracted with DCM. The organic layer was separated, dried (Na_2SO_4), filtered and concentrated *in vacuo*. The crude product was purified by flash column chromatography (silica, EtOAc in Heptane, 0/100 to 25/75). The desired fractions were collected and concentrated in vacuo to yield but-3-en-1-yl 4-cyanobenzoate **7d** (1.86 g, 9.2 mmol, 77%) as a colourless oil.

^{1}H NMR (400 MHz, $CDCl_3$) δ 8.15 - 8.10 (m, 2H), 7.76 - 7.71 (m, 2H), 5.92 - 5.78 (m, 1H), 5.21 - 5.08 (m, 2H), 4.43 - 4.37 (m, 2H), 2.57 - 2.49 (m, 2H); **^{13}C NMR** (101 MHz, $CDCl_3$) δ 165.0, 134.3, 133.8, 132.3, 130.2, 118.1, 117.8, 116.5, 64.8, 33.2; **HRMS** (ESI-TOF) calculated for

$C_{12}H_{12}NO_2$ $[M+H]^+$: 202.0863; found 202.0865; **IR** (neat) 2232, 1720, 1407, 1269, 1177, 1105, 1019, 920, 862, 766, 691, 642.

3. Optimization of the reaction conditions

Table S1: Optimization of the reaction conditions for the hydrosulfonylation of *N*-phenylacrylamide[a]

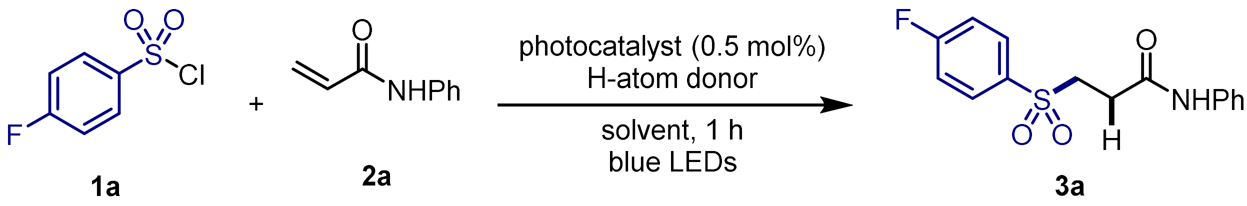

Entry	Catalyst	H-donor	Solvent	Yield
1	*fac*-Ir(ppy)$_3$	-	MeCN	traces
2[b]	*fac*-Ir(ppy)$_3$	-	THF	traces
3	*fac*-Ir(ppy)$_3$	Ph_3CH	MeCN	traces
4	*fac*-Ir(ppy)$_3$	Et_3SiH	MeCN	traces
5	*fac*-Ir(ppy)$_3$	Ph_3SiH	MeCN	traces
6	*fac*-Ir(ppy)$_3$	(TMS)$_3$SiH	MeCN	90% (82%)[c]
7	*fac*-Ir(ppy)$_3$	Bu_3SnH	MeCN	28%
8	*fac*-Ir(ppy)$_3$	Hantzsch ester	MeCN	34%
9	*fac*-Ir(ppy)$_3$	PhSH	MeCN	26%
10	*fac*-Ir(ppy)$_3$	(TMS)$_3$SiH	DMF	65%
11	*fac*-Ir(ppy)$_3$	(TMS)$_3$SiH	MeOH	49%
12	*fac*-Ir(ppy)$_3$	(TMS)$_3$SiH	DMSO	traces
13	[Ir(ppy)$_2$(dtbby)$_2$)PF$_6$]	(TMS)$_3$SiH	MeCN	55%
14	Ir[dCF$_3$(ppy)$_2$](dtbbpy)PF$_6$	(TMS)$_3$SiH	MeCN	88%
15	Eosin Y	(TMS)$_3$SiH	MeCN	78%
16[d]	*fac*-Ir(ppy)$_3$	(TMS)$_3$SiH	MeCN	81%
17[e]	*fac*-Ir(ppy)$_3$	(TMS)$_3$SiH	MeCN	55%
18[f]	*fac*-Ir(ppy)$_3$	(TMS)$_3$SiH	MeCN	72%
19[g]	*fac*-Ir(ppy)$_3$	(TMS)$_3$SiH	MeCN	nd
20[h]	-	(TMS)$_3$SiH	MeCN	23%
21[i]	*fac*-Ir(ppy)$_3$	(TMS)$_3$SiH	MeCN	81%

[a] Reaction condition: **1a** (0.25 mmol), **2a** (0.1 mmol), photocatalyst (0.5 mol%), (TMS)$_3$SiH (0.2 mmol), solvent (0.6 mL) under blue light (λ = 450 nm) irradiation for 1 hour. The yield was determined by ^{19}F NMR using α,α,i-trifluorotoluene as internal standard. [b] THF was used as solvent and H-atom donor [c] Yields of isolated product. [d] 1.5 equiv. of H-donor was used. [e] 2.5 equiv. of H-donor was used. [f] 1.5 equiv. of **1a** [g] In the dark. [h] In absence of photocatalyst, the reaction was yielding an inconsistent complex mixture of products; [i] Reaction performed under nitrogen atmosphere and in degassed solvent. *fac*-Ir(ppy)$_3$: tris[2-phenylpyridinato-C^2,*N*]iridium(III); [Ir(ppy)$_2$(dtbby)$_2$)PF$_6$]: [4,4′-*Bis*(1,1-dimethylethyl)-2,2′-bipyridine-*N*1,*N*1′]*bis*[2-(2-pyridinyl-*N*)phenyl-C]iridium(III) hexafluorophosphate; Ir[dCF$_3$(ppy)$_2$](dtbbpy)PF$_6$: [4,4′-Bis(1,1-dimethylethyl)-2,2′-bipyridine-*N*1,*N*1′]bis[3,5-difluoro-2-[5-(trifluoromethyl)-2-pyridinyl-*N*]phenyl-C]Iridium(III) hexafluorophosphate.

NOTE:

- The amount of sulfonyl chloride can be reduced (< 2.5 equivalents) depending on the substitution pattern of the sulfone as well as the olefin used. The substrate scope described in the paper has been performed using 2.5 equivalents of sulfonyl chloride in order to promote higher yields for all substrates.

- The temperature of the reaction mixture could reach 35 °C depending on the distance of the vial to the kessil lights. Fluctuation of the temperature (from room temperature using a fan to 60 degrees in flow) didn't impact the outcome of the reaction.

- Desulfonylation has never been observed under our reaction conditions.

4. Mechanistic investigations

4.1. Radical-trapping experiment with TEMPO

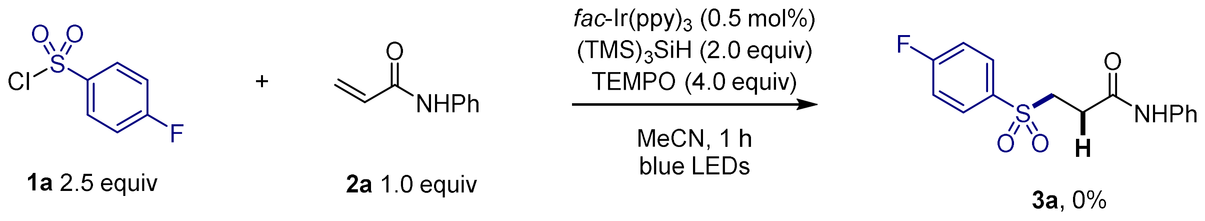

4-fluorobenzenesulfonyl chloride (243 mg, 1.3 mmol, 2.5 equiv) was added to a stirred suspension of *N*-phenylacrylamide (74 mg, 0.5 mmol, 1.0 equiv), *fac*-Ir(ppy)3 (1.70 mg, 0.0025 mmol, 0.5 mol%), MeCN (3.0 mL), (TMS)3SiH (309 µL, 1.0 mmol, 2.0 equiv) and (2,2,6,6-tetramethylpiperidin-1-yl)oxyl (TEMPO) (313 mg, 2.0 mmol, 4.0 equiv). The reaction mixture was stirred under blue LED irradiation for 1 hour. LCMS analysis of the crude reaction mixture showed that no product was formed (starting material remaining).

4.2. Reduction of sulfonyl chloride to sulfinic acid 4a

4-fluorobenzenesulfonyl chloride (19.4 mg, 0.1 mmol, 1.0 equiv) was added to a stirred suspension of *fac*-Ir(ppy)$_3$ (0.34 mg, 0.005 mmol, 0.5 mol%), (TMS)$_3$SiH (31 uL, 0.1 mmol, 1.0 equiv), MeCN (0.6 mL) and α,α,α-trifluorotoluene (12.3 uL, 0.1 mmol, 1.0 equiv; used as internal standard). The vial was stirred under blue LED irradiation with two Kessil LEDSs (35W, 450 nm, approximately 4 cm away from the light source) at 30 °C for 30 minutes. The crude reaction mixture was analysed by quantitative ^{19}F NMR (using α,α,α-trifluorotoluene as internal standard).

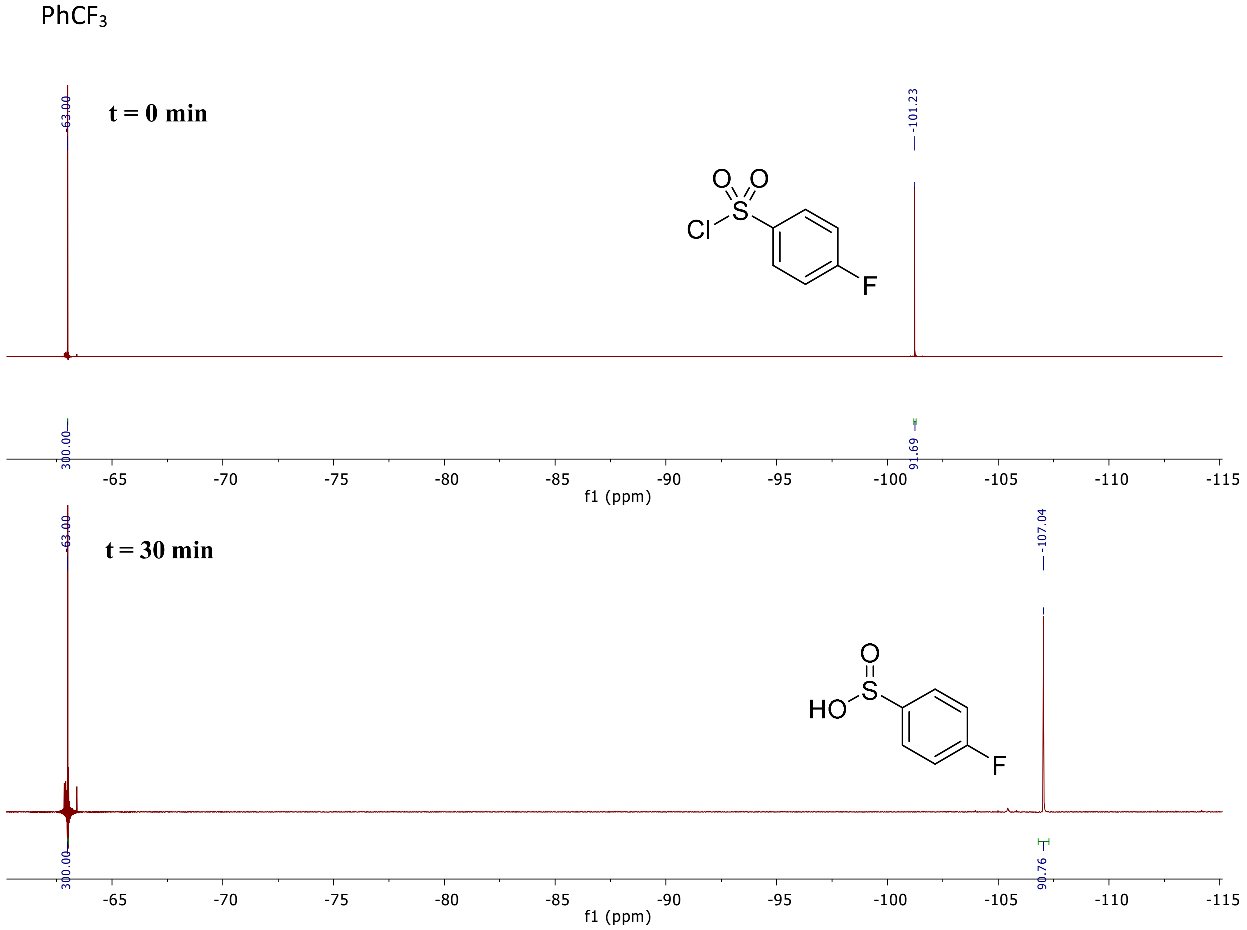

Figure S1: Quantitative ^{19}F NMR of the reaction mixture before and after irradiation with blue LEDs.

4.3 Photoredox-catalyzed hydrosulfonylation of alkenes using sulfinic acid 4a

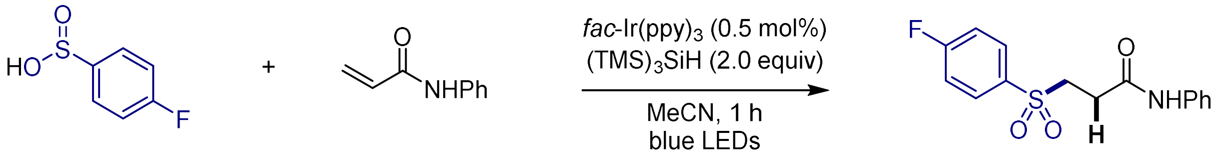

4a (2.5 equiv) **2a** (1.0 equiv) **3a**, 23%

4-fluorobenzenesulfinic acid (200 mg, 1.3 mmol, 2.5 equiv) was added to a stirred suspension of *N*-phenylacrylamide (74 mg, 0.5 mmol, 1.0 equiv), *fac*-Ir(ppy)$_3$ (1.70 mg, 0.0025 mmol, 0.5 mol%), MeCN (3.0 mL) and (TMS)$_3$SiH (309 µL, 1.0 mmol, 2.0 equiv). The reaction mixture was stirred under blue LED irradiation for 1 hour. The solution was concentrated *in vacuo* and purified by flash column chromaography (silica, EtOAc in Heptane, 0/100 to 100/0) to afford **3a** in 23% yield (35 mg, 0.11 mmol).

4.4. Michael addition of sulfinic acids to electron-deficient alkenes

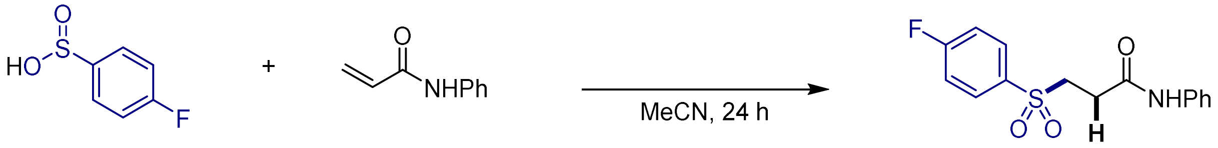

4a (2.5 equiv) **2a** (1.0 equiv) **3a**, 58%

4-fluorobenzenesulfinic acid (200 mg, 1.3 mmol, 2.5 equiv) was added to a stirred suspension of *N*-phenylacrylamide (74 mg, 0.5 mmol, 1.0 equiv) in MeCN (3.0 mL). The reaction mixture was stirred under blue LED irradiation for 1 hour. The solution was concentrated *in vacuo* and purified by flash column chromaography (silica, EtOAc in Heptane, 0/100 to 100/0) to afford **3a** in 58% yield (89 mg, 0.29 mmol).

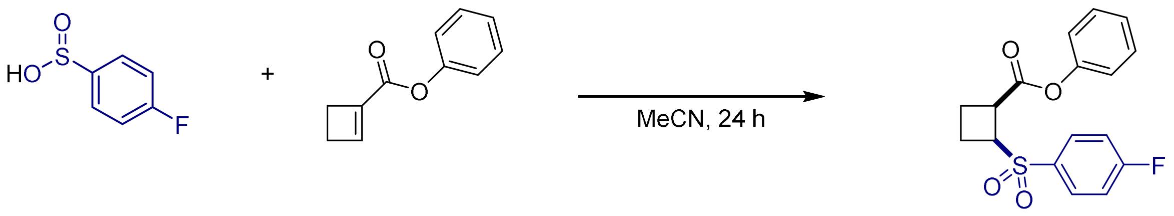

4a (2.5 equiv) **2o** (1.0 equiv) **3ac**, 0%

4-fluorobenzenesulfinic acid (200 mg, 1.3 mmol, 2.5 equiv) was added to a stirred suspension of phenyl cyclobut-1-ene-1-carboxylate (87 mg, 0.5 mmol, 1.0 equiv) in MeCN (3.0 mL). The reaction mixture was stirred under blue LED irradiation for 1 hour. LCMS analysis of the crude reaction mixture showed no conversion towards the desired product **3ac**.

Previous reports showed that sulfinic acids can undergo Michael addition to electron-deficient alkenes.[17] Nevertheless, as shown above, the efficiency of this addition is dependent on the alkene. An ionic pathway would involve the formation of an enolate intermediate. Based on deuteration experiments (see section 4.4. below), the cascade cyclization affording cyclobutylspirooxindoles **5** as well as the chlorosulfonylation (see Scheme 3 in the manuscript), the reaction most likely proceeds *via* a radical pathway.

4.5. Deuteration experiment

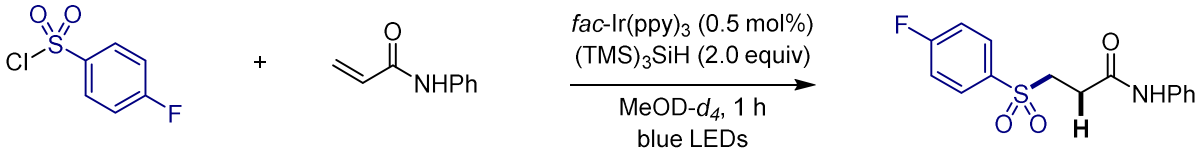

1a (2.5 equiv) **2a** (1.0 equiv) [D]**3a**, R = D, 0% [H]**3a**, R = H, 47%

4-fluorobenzenesulfonyl chloride (243 mg, 1.3 mmol, 2.5 equiv) was added to a stirred suspension of *N*-phenylacrylamide (74 mg, 0.5 mmol, 1.0 equiv), *fac*-Ir(ppy)3 (1.70 mg, 0.0025 mmol, 0.5 mol%), MeOD-d_4 (3.0 mL) and (TMS)3SiH (309 µL, 1.0 mmol, 2.0 equiv). The reaction mixture was stirred under blue LED irradiation for 1 hour. The solution was concentrated *in vacuo* and purified by flash column chromaogography (silica, EtOAc in Heptane, 0/100 to 100/0) to afford [H]**3a** in 47% yield (72 mg, 0.23 mmol).

4.6. Isolation of Chlorotris(trimethylsilyl)silane (9)

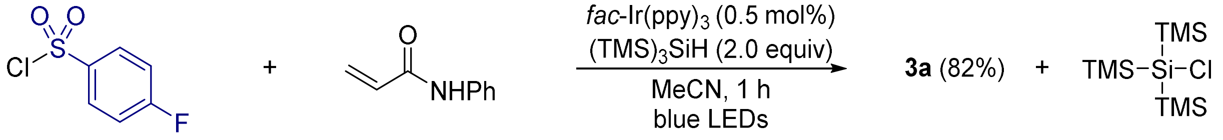

1a (2.5 equiv) **2a** (1.0 equiv) **9** (69%)

4-fluorobenzenesulfonyl chloride (243 mg, 1.3 mmol, 2.5 equiv) was added to a stirred suspension of *N*-phenylacrylamide (74 mg, 0.5 mmol, 1.0 equiv), *fac*-Ir(ppy)3 (1.70 mg, 0.0025 mmol, 0.5 mol%), MeCN (3.0 mL) and (TMS)3SiH (309 µL, 1.0 mmol, 2.0 equiv). The reaction mixture was stirred under blue LED irradiation for 1 hour. The solvent was removed *in vacuo* and the residue was purified by column chromatography (silica, EtOAc in heptane 0/100 to 20/80) to yield **9** (98 mg, 0.34 mmol, 69%) as a colourless oil. **^{1}H NMR** (400 MHz, $CDCl_3$) δ 0.23 (s, 27H); **^{13}C NMR** (101 MHz, $CDCl_3$) δ -0.6. Spectroscopic data were in accordance with a commercial sample.

4.7 Stern-Volmer fluorescence quenching study

A Stern-Volmer fluorescence quenching study of *fac*-Ir(ppy)$_3$ was performed with different concentrations of reaction components in MeCN under an argon atmosphere.

A solution of *fac*-Ir(ppy)$_3$ (0.01 mM) was treated with 0.2-1.0 mM of either sulfonyl chloride **1a**, alkene **2a**, (TMS)$_3$SiH or *N,N*-dimethylsulfamoyl chloride in MeCN. The samples were irradiated at 370 nm and luminescence was measured over a range of 450 – 700 nm (λ_{max} = 532 nm). I_0/I was plotted against the different concentrations of the quenchers (Figure S2).

c[mM]	4-F-PhSO$_2$Cl	(TMS)$_3$SiH	**2a**	Me$_2$NSO$_2$Cl	TEMPO
0.0	1.000	1.000	1.000	1.000	1.000
0.2	1.508	1.068	1.087	0.941	0.993
0.4	1.905	1.055	1.004	0.910	1.086
0.6	2.402	1.064	1.039	0.910	1.166
0.8	2.842	1.104	1.054	1.060	1.166
1.0	3.318	1.080	1.106	1.068	0.986

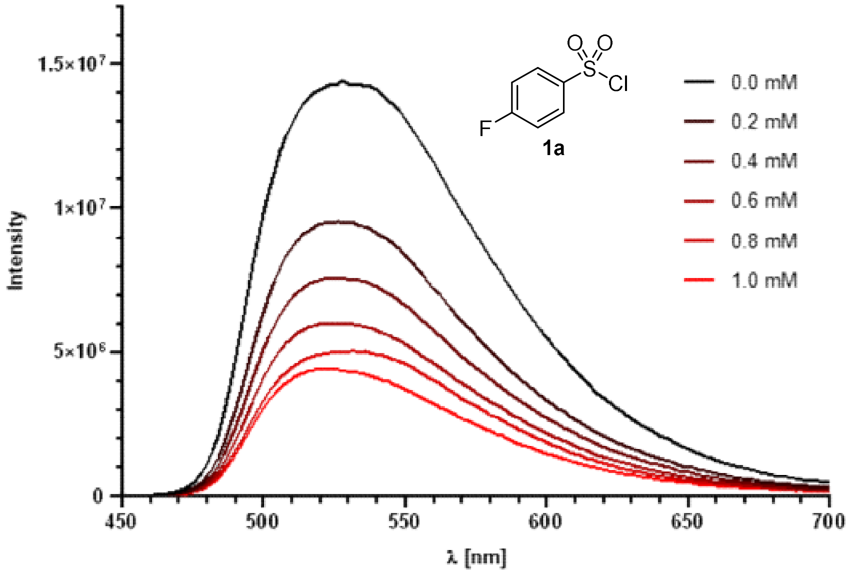

Figure S2: Stern-Volmer fluorescence quenching experiment

Note: (TMS)$_3$SiH and *fac*-Ir(ppy)$_3$ are poorly insoluble in MeCN and sonication was necessary to solubilise all of the reagents.

5. Scale-up experiments

The scale-up experiments (in batch and in continuous flow) have been performed on the synthesis of compound **3am**.

5.1 Scale-up experiment in batch

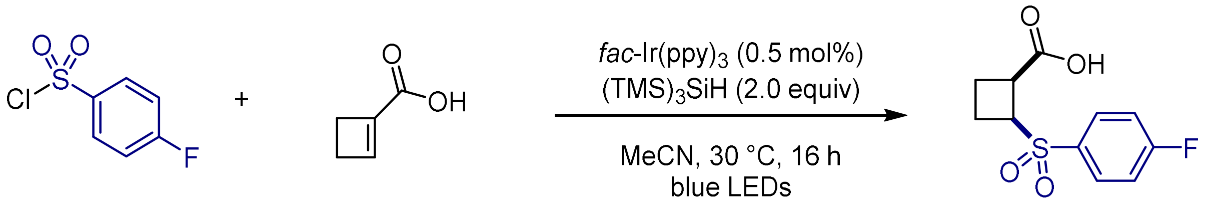

1a (2.5 equiv) **2y** (1.0 equiv) **3am**, 81% (d.r.: 89/11)

Scheme S1: Multigram synthesis of **3am** in batch.

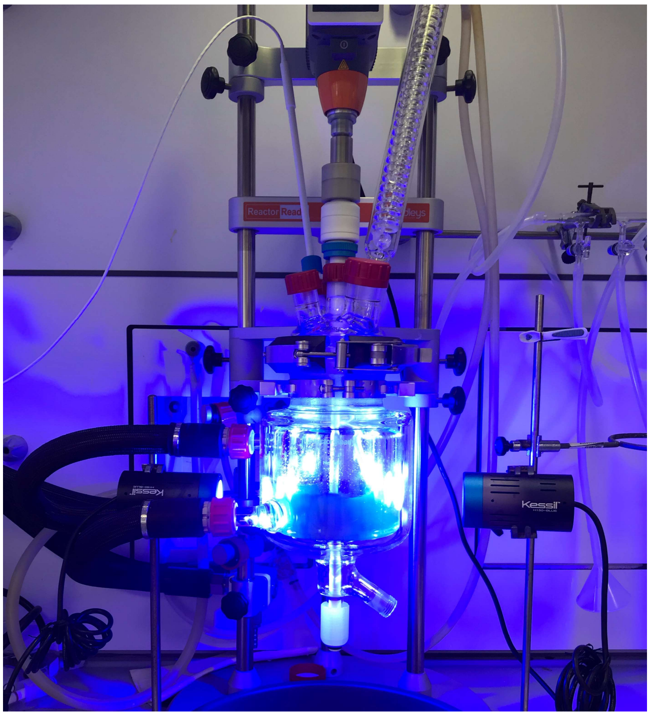

Figure S3: Scale-up experiment in batch performed in a Radleys Reactor Ready of 1 Liter.

A stock solution of cyclobut-1-ene-1-carboxylic acid **2y** in MeCN (185 mL, 0.276 M, 50.97 mmol, 1.0 equiv)* was added to a 500 mL round bottom flask. To that solution was added 4-

fluorobenzenesulfonyl chloride (24.8 g, 127.4 mmol, 2.5 equiv) and *fac*-Ir(ppy)$_3$ (167.6 mg, 0.25 mmol, 0.5 mol%). This suspension was transfered to the Radley 1L reactor, followed by the addition of (TMS)Si$_3$H (31.4 mL, 101.9 mmol, 2.0 equiv) and MeCN (115 mL). The reactor was irradiated with two Kessil H150-Blue LED lights (34 W, 5 cm distance, see picture above) for 16 h at 30 °C. The reaction mixture was collected from the outlet and concentrated *in vacuo*. The crude was purified by flash column chromatography (silica, sample dry-loading with Celite®, EtOAc in Heptane with 1% acetic acid, 0/100 to 100/0). The desired fractions were collected and concentrated *in vacuo* to give the desired hydrosulfonylated product **3am** as mixture of diastereoisomers (89/11, *cis*/*trans*) and as an off-white solid (10.7 g, 41.5 mmol, 81%). Trituration with Et$_2$O afforded exclusively the *cis*-diastereoisomer as a white solid (9.1 g, 35.3 mmol, 69%).

* The approximate concentration of the solution was calculated using quantitative ^{1}H NMR. To a sample of the solution containing **2y** in MeCN (20 μL) was added CH_2Br_2 (7.0 μL, 0.1 mmol) and $CDCl_3$ (ca. 0.5 mL). The concentration of the **2y** stock solution was determined by ^{1}H NMR spectroscopy to be 0.276 M (n = 3).

5.2 Scale-up experiment in continuous flow

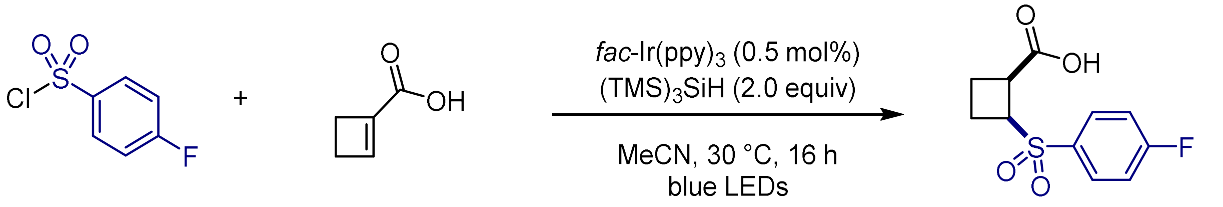

1a (2.5 equiv) **2y** (1.0 equiv) **3am**, 72% (d.r.: 89/11)

Scheme S2: Multigram synthesis of **3am** in continuous flow.

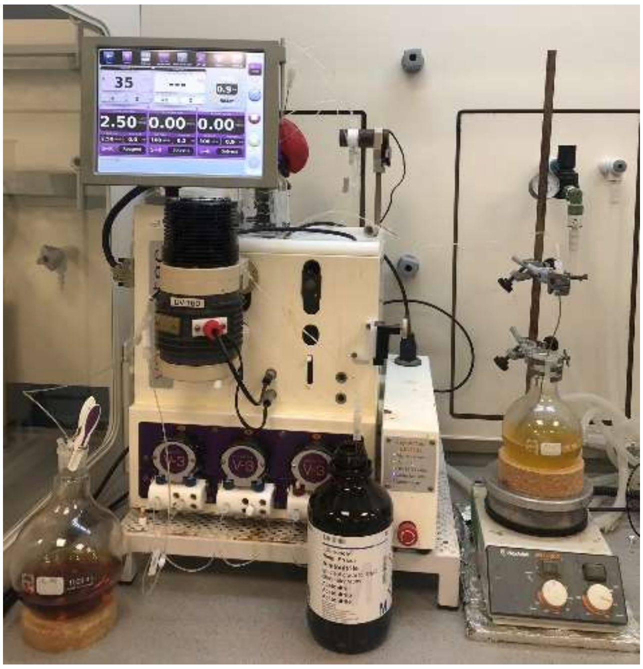

Figure S4: Scale-up experiment in continuous flow performed in a Vapourtec photoreactor

A stock solution of cyclobut-1-ene-1-carboxylic acid **2y** in MeCN (185 mL, 0.276M, 50.97 mmol, 1.0 equiv)* was added to a 500 mL round bottom flask. To that solution was added 4-fluorobenzenesulfonyl chloride (24.8 g, 127.4 mmol, 2.5 equiv), *fac*-Ir(ppy)$_3$ (167.6 mg, 0.25 mmol, 0.5 mol%), (TMS)Si$_3$H (31.4 mL, 101.9 mmol, 2.0 equiv), MeCN (100 mL) and DMF (170 mL). The solution was pumped through a Vapourtec photoreactor (fluoropolymer tube, 1.3 mm i.d., 10 mL) and the liquid flowrate was set at 2.5 mL/min (4 min residence time). The reactor was irradiated with 54 blue LEDs (410 nm, total power 24 W) at 30 °C (internal temperature of the photoreactor: 35 °C). The reaction mixture collected from the outlet was concentrated *in vacuo*.

The crude was purified by flash column chromatography (silica, EtOAc in Heptane with 1% acetic acid, 0/100 to 100/0). The desired fractions were collected and concentrated *in vacuo* to give the desired hydrosulfonylated product **3am** as a mixture of diastereoisomers (89/11, *cis*/*trans*) as an off-white solid (9.5 g, 36.8 mmol, 72%). Trituration with Et_2O afforded exclusively the *cis*-diastereoisomer as white solid (7.3 g, 28.3 mmol, 56%).

6. General procedures

General procedure A: Hydrosulfonylation of electron-deficient alkenes

To a 7 mL vial equipped with a magnetic stir bar, was added alkene (0.50 mmol, 1.0 equiv), *fac*-Ir(ppy)$_3$ (1.70 mg, 0.0025 mmol, 0.5 mol%), MeCN (3.0 mL), (TMS)$_3$SiH (309 uL, 1.0 mmol, 2.0 equiv) and sulfonyl chloride (1.25 mmol, 2.5 equiv) under air. The vial was equipped with a Teflon septum and stirred under blue LED irradiation with two Kessil LEDs (35W, 450 nm, approximately 4 cm away from the reaction mixture) for 1 hour. The solvent was removed *in vacuo* and the residue was purified by column chromatography (silica, EtOAc in heptane 0/100 to 100/0) to yield the desired product(s).

General procedure B: Hydrosulfonylation of alkynes

To a 7 mL vial equipped with a magnetic stir bar, was added alkyne (0.50 mmol, 1.0 equiv), *fac*-Ir(ppy)$_3$ (1.70 mg, 0.0025 mmol, 0.5 mol%), MeCN (3.0 mL), (TMS)$_3$SiH (309 uL, 1.0 mmol, 2.0 equiv) and sulfonyl chloride (1.25 mmol, 2.5 equiv) under air. The vial was equipped with a Teflon septum and stirred under blue LED irradiation with two Kessil LEDs (35W, 450 nm, approximately 4 cm away from the reaction mixture) for 16 hours. The solvent was removed *in vacuo* and the residue was purified by column chromatography (silica, EtOAc in heptane 0/100 to 100/0) to yield the desired product(s).

General procedure C: Hydrosulfonylation of unactivated alkenes

To a 7 mL vial equipped with a magnetic stir bar, was added alkene (0.50 mmol, 1.0 equiv), *fac*-Ir(ppy)$_3$ (1.70 mg, 0.0025 mmol, 0.5 mol%), 4-mercaptophenol (13 mg, 0.1 mmol, 0.2 equiv), MeCN (3.0 mL), (TMS)$_3$SiH (309 uL, 1.0 mmol, 2.0 equiv) and sulfonyl chloride (1.25 mmol, 2.5 equiv) under air. The vial was equipped with a Teflon septum and stirred under blue LED irradiation with two Kessil LEDs (35W, 450 nm, approximately 4 cm away from the reaction mixture) for 16 hours. The solvent was removed *in vacuo* and the residue was purified by column chromatography (silica, EtOAc in heptane 0/100 to 100/0) to yield the desired product(s).

7. Characterizations

3-((4-fluorophenyl)sulfonyl)-*N*-phenylpropanamide (3a)

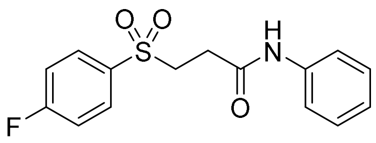

General procedure A was followed to obtain **3a** (126 mg, 0.41 mmol, 82%) as a white solid.
^{1}H NMR (500 MHz, $CDCl_3$) δ 8.12 (s, 1H), 7.99 - 7.94 (m, 2H), 7.47 - 7.43 (m, 2H), 7.31 - 7.27 (m, 2H), 7.26 - 7.21 (m, 2H), 7.13 - 7.09 (m, 1H), 3.58 (t, J = 8.1 Hz, 2H), 2.95 (t, J = 8.1 Hz, 2H); **^{13}C NMR** (126 MHz, $CDCl_3$) δ 167.1, 166.5 (d, $J_{C\text{-}F}$ = 257.8 Hz), 137.65, 134.8 (d, $J_{C\text{-}F}$ = 3.2 Hz), 131.1 (d, $J_{C\text{-}F}$ = 9.7 Hz), 129.1, 124.7, 120.1, 117.0 (d, $J_{C\text{-}F}$ = 22.6 Hz), 52.2, 29.8; **^{19}F NMR** (471 MHz, $CDCl_3$) δ -102.5 (m, 1F); **HRMS** (ESI-TOF) calculated for $C_{15}H_{15}FO_3N^{32}S$ $[M+H]^+$: 308.0751; found 308.0759; **m.p.**: 143 - 144 °C; **IR** (neat): 2981, 1689, 1661, 1594, 1527, 1493, 1445, 1399, 1312, 1292, 1256, 1226, 1180, 1157, 1139, 1084, 1055, 1015, 966, 951, 904, 839, 821, 794, 774, 752, 727, 693, 670, 651.

3-((4-methoxyphenyl)sulfonyl)-*N*-phenylpropanamide (3b)

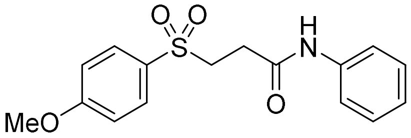

General procedure A was followed to obtain **3b** (137 mg, 0.43 mmol, 86%) as a white solid.
^{1}H NMR (400 MHz, $CDCl_3$) δ 8.45 (s, 1H), 7.85 - 7.80 (m, 2H), 7.45 - 7.39 (m, 2H), 7.25 - 7.18 (m, 2H), 7.06 - 7.00 (m, 1H), 6.97 - 6.92 (m, 2H), 3.78 (s, 3H), 3.53 (t, J = 7.9 Hz, 1H), 2.89 (t, J = 7.8 Hz, 1H); **^{13}C NMR** (101 MHz, $CDCl_3$) δ 167.5, 164.1, 137.9, 130.3, 129.9, 128.9, 124.4, 120.0, 114.7, 55.7, 52.2, 30.1; **HRMS** (ESI-TOF) calculated for $C_{16}H_{18}NO_4{}^{32}S$ $[M+H]^+$: 320.0951; found 320.0956; **m.p.**: 111 - 113 °C; **IR** (neat) 2981, 1690, 1595, 1545, 1496, 1445, 1425, 1315, 1292, 1264, 1172, 1138, 1087, 1051, 1027, 952, 835, 804, 804, 760, 728, 697, 653.

***N*-phenyl-3-((4-(trifluoromethyl)phenyl)sulfonyl)propenamide (3c)**

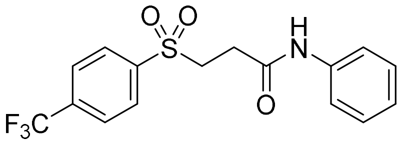

General procedure A was followed to obtain **3c** (164 mg, 0.46 mmol, 92%) as a white solid. **^{1}H NMR** (500 MHz, DMSO-*d$_6$*) δ 10.02 (s, 1H), 8.15 (d, J = 8.1 Hz, 2H), 8.02 (d, J = 8.2 Hz, 2H), 7.45 (d, J = 7.2 Hz, 2H), 7.26 (t, J = 8.3 Hz, 2H), 7.02 (t, J = 7.4 Hz, 1H), 3.73 (t, J = 7.3 Hz, 2H), 2.71 (t, J = 7.3 Hz, 2H); **^{13}C NMR** (126 MHz, DMSO-*d$_6$*) δ 167.0, 142.5, 138.8, 133.5 (q, $J_{C\text{-}F}$ = 32.4 Hz), 129.0, 128.7, 126.6 (q, $J_{C\text{-}F}$ = 3.8 Hz), 123.3, 123.3 (q, $J_{C\text{-}F}$ = 273.0 Hz), 119.0, 50.5, 29.4; **^{19}F NMR** (471 MHz, DMSO-*d$_6$*) δ -61.8 (s, 3F); **HRMS** (ESI-TOF) calculated for $C_{16}H_{15}F_3NO_3{}^{32}S$ $[M+H]^+$: 358.0718; found 358.0728; **m.p.**: 112 - 114 °C; **IR** (neat) 2981, 2889, 1656, 1531, 1498, 1319, 1271, 1258, 1169, 1147, 1109, 1062, 1018, 849, 792, 776, 735, 696.

3-((4-nitrophenyl)sulfonyl)-*N*-phenylpropanamide (3d)

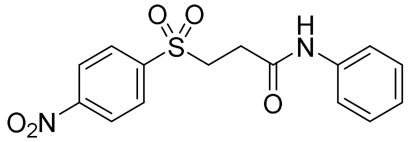

General procedure A was followed to obtain **3d** (150 mg, 0.45 mmol, 90%) as a white solid. **^{1}H NMR** (400 MHz, DMSO-*d$_6$*) δ 10.01 (s, 1H), 8.45 - 8.39 (m, 2H), 8.22 - 8.16 (m, 2H), 7.48 - 7.42 (m, 2H), 7.28 - 7.22 (m, 2H), 7.04 - 6.98 (m, 1H), 3.76 (t, J = 7.3 Hz, 2H), 2.72 (t, J = 7.3 Hz, 2H); **^{13}C NMR** (101 MHz, DMSO-*d$_6$*) δ 167.0, 150.5, 143.9, 138.7, 129.8, 128.7, 124.6, 123.3, 119.0, 50.6, 29.3; **HRMS** (ESI-TOF) calculated for $C_{15}H_{13}N_2O_5{}^{32}S$ $[M-H]^-$: 333.0551; found 333.0548; **m.p.**: 185 - 188 °C; **IR** (neat) 2981, 2889, 1692, 1543, 1527, 1495, 1442, 1383, 1353, 1303, 1250, 1142, 1083, 1011, 957, 854, 815, 773, 759, 737, 704, 693, 655, 606.

3-((4-cyanophenyl)sulfonyl)-*N*-phenylpropanamide (3e)

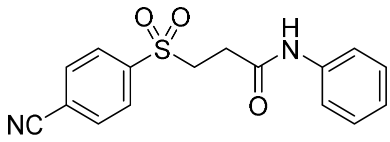

General procedure A was followed to obtain **3e** (131 mg, 0.42 mmol, 83%) as a white solid.
^{1}H NMR (400 MHz, DMSO-*d$_6$*) δ 10.01 (s, 1H), 8.16 - 8.07 (m, 4H), 7.48 - 7.44 (m, 2H), 7.31 - 7.24 (m, 2H), 7.07 - 7.00 (m, 1H), 3.72 (t, *J* = 7.3 Hz, 2H), 2.69 (t, *J* = 7.4 Hz, 2H); **^{13}C NMR** (101 MHz, DMSO-*d$_6$*) δ 167.0, 142.6, 138.8, 133.5, 128.7, 128.7, 123.3, 119.0, 117.5, 116.3, 50.4, 29.3; **HRMS** (ESI-TOF) calculated for $C_{16}H_{13}N_2O_3{}^{32}S$ $[M-H]^-$: 313.0652; found 313.0648; **m.p.**: 157 - 159 °C; **IR** (neat) 2981, 2889, 1693, 1600, 1538, 1496, 1439, 1394, 1356, 1315, 1249, 1177, 1149, 1132, 1083, 1024, 985, 968, 954, 845, 818, 788, 761, 747, 721, 691, 655.

3-((5-bromothiophen-2-yl)sulfonyl)-*N*-phenylpropanamide (3f)

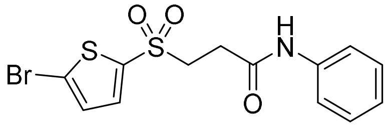

General procedure A was followed to obtain **3f** (159 mg, 0.43 mmol, 85%) as a white solid.
^{1}H NMR (400 MHz, $CDCl_3$) δ 7.67 (s, 1H), 7.47 (d, *J* = 4.0 Hz, 1H), 7.43 (d, *J* = 7.7 Hz, 2H), 7.30 (t, *J* = 7.9 Hz, 2H), 7.15 - 7.08 (m, 2H), 3.65 (t, *J* = 7.7 Hz, 2H), 2.90 (t, *J* = 7.8 Hz, 2H); **^{13}C NMR** (101 MHz, $CDCl_3$) δ 166.7, 140.3, 137.5, 134.9, 131.4, 129.2, 124.9, 123.0, 120.1, 53.4, 30.4; **HRMS** (ESI-TOF) calculated for $C_{13}H_{13}NO_3{}^{79}Br^{32}S_2$ $[M+H]^+$: 375.9515; found 375.9497; **m.p.**: 139 - 142 °C; **IR** (neat) 2981, 2889, 1674, 1600, 1535, 1497, 1474, 1462, 1442, 1396, 1318, 1253, 1198, 1149, 1129, 1078, 1019, 989, 967, 955, 808, 778, 751, 709, 688, 665.

N-phenyl-3-(pyridin-3-ylsulfonyl)propenamide (3g)

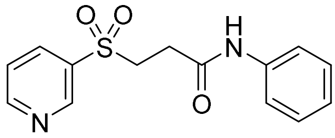

General procedure A was followed to obtain **3f** (107 mg, 0.37 mmol, 74%) as a white solid.
^{1}H NMR (400 MHz, DMSO-d_6) δ 10.04 (s, 1H), 9.06 (dd, J = 2.4, 0.9 Hz, 1H), 8.90 (dd, J = 4.9, 1.6 Hz, 1H), 8.32 (ddd, J = 8.0, 2.4, 1.6 Hz, 1H), 7.69 (ddd, J = 8.2, 4.8, 0.8 Hz, 1H), 7.50 - 7.45 (m, 2H), 7.31 - 7.24 (m, 2H), 7.06 - 6.99 (m, 1H), 3.73 (t, J = 7.4 Hz, 2H), 2.73 (t, J = 7.4 Hz, 2H); **^{13}C NMR** (101 MHz, DMSO-d_6) δ 167.1, 154.4, 148.5, 138.8, 136.2, 135.1, 128.7, 124.4, 123.3, 119.1, 50.9, 29.4; **HRMS** (ESI-TOF) calculated for $C_{14}H_{15}N_2O_3{}^{32}S$ $[M+H]^+$: 291.0798; found 291.0804; **m.p.**: 181 - 183 °C; **IR** (neat) 2981, 2889, 1687, 1599, 1546, 1489, 1444, 1383, 1301, 1255, 1155, 1103, 1083, 956, 828, 809, 776, 753, 701, 688, 623.

3-((5-acetamidothiazol-2-yl)sulfonyl)-N-phenylpropanamide (3h)

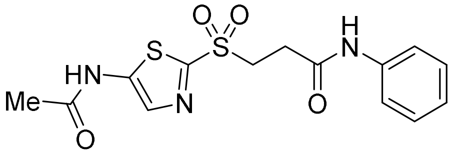

General procedure A was followed to obtain **3h** (134 mg, 0.38 mmol, 76%) as a white solid.
^{1}H NMR (500 MHz, DMSO-d_6) δ 12.78 (s, 1H), 10.04 (s, 1H), 8.08 (s, 1H), 7.51 - 7.47 (m, 2H), 7.30 - 7.25 (m, 2H), 7.05 - 7.00 (m, 1H), 3.69 (t, J = 7.3 Hz, 2H), 2.76 (t, J = 7.3 Hz, 2H), 2.19 (s, 3H); **^{13}C NMR** (126 MHz, DMSO-d_6) δ 169.8, 167.2, 163.7, 145.7, 138.8, 128.7, 126.8, 123.3, 119.1, 52.7, 30.0, 22.4; **HRMS** (ESI-TOF) calculated for $C_{14}H_{14}N_3O_4{}^{32}S_2$ $[M-H]^-$: 352.0431; found 352.0434; **m.p.**: 238 - 240 °C; **IR** (neat) 2981, 2889, 1687, 1675, 1552, 1498, 1443, 1381, 1316, 1300, 1277, 1255, 1229, 1178, 1142, 1126, 1958, 1025, 1004, 956, 797, 772, 753, 706, 691, 671.

3-(cyclopropylsulfonyl)-*N*-phenylpropanamide (3i)

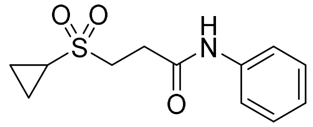

General procedure A was followed to obtain **3i** (99 mg, 0.39 mmol, 78%) as a white solid. **^{1}H NMR** (400 MHz, $CDCl_3$) δ 8.12 (s, 1H), 7.53 - 7.48 (m, 2H), 7.34 - 7.27 (m, 2H), 7.13 - 7.07 (m, 1H), 3.52 (t, *J* = 7.4 Hz, 2H), 2.95 (t, *J* = 7.5 Hz, 2H), 2.47 (tt, *J* = 7.9, 4.8 Hz, 1H), 1.30 -1.21 (m, 2H), 1.12 - 1.02 (m, 2H); **^{13}C NMR** (101 MHz, $CDCl_3$) δ 167.6, 137.8, 129.2, 124.7, 120.1, 49.5, 30.1, 29.5, 5.1; **HRMS** (ESI-TOF) calculated for $C_{12}H_{16}NO_3{}^{32}S$ $[M+H]^+$: 254.0845; found 254.0849; **m.p.**: 128 - 130 °C; **IR** (neat) 2981, 2889, 1689, 1596, 1541, 1500, 1488, 1442, 1422, 1383, 1307, 1269, 1252, 1186, 1128, 1084, 1072, 1040, 956, 887, 829, 752, 739, 698, 681.

3-(isopropylsulfonyl)-*N*-phenylpropanamide (3j)

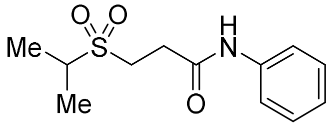

General procedure A was followed to obtain **3j** (108 mg, 0.43 mmol, 85%) as a white solid. **^{1}H NMR** (400 MHz, $CDCl_3$) δ 8.31 (s, 1H), 7.51 (d, *J* = 7.4 Hz, 2H), 7.32 - 7.26 (m, 2H), 7.12 - 7.06 (m, 1H) 3.40 (t, *J* = 7.8 Hz, 2H), 3.18 (hept, *J* = 6.8 Hz, 1H), 2.97 (t, *J* = 7.8 Hz, 2H), 1.42 (d, *J* = 6.9 Hz, 6H); **^{13}C NMR** (101 MHz, $CDCl_3$) δ 167.8, 137.9, 129.1, 124.6, 120.1, 54.1, 44.8, 28.4, 15.4; **HRMS** (ESI-TOF) calculated for $C_{12}H_{18}NO_3{}^{32}S$ $[M+H]^+$: 256.1002; found 256.1013; **m.p.**: 120 - 122 °C; **IR** (neat) 2981, 1672, 1602, 1547, 1499, 1462, 1440, 1422, 1383, 1307, 1253, 1154, 1139, 1125, 1081, 1060, 953, 908, 878, 804, 753, 726, 694, 664.

3-(methylsulfonyl)-*N*-phenylpropanamide (3k)

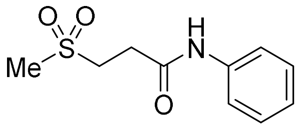

General procedure A was followed to obtain **3k** (90 mg, 0.40 mmol, 79%) as a white solid.
^{1}H NMR (400 MHz, DMSO-d_6) δ 10.11 (s, 1H), 7.60 - 7.55 (m, 2H), 7.34 - 7.27 (m, 2H), 7.07 - 7.02 (m, 1H), 3.43 (t, J = 7.6 Hz, 2H), 3.02 (s, 3H), 2.81 (t, J = 7.6 Hz, 2H); **^{13}C NMR** (101 MHz, DMSO-d_6) δ 167.9, 139.0, 128.7, 123.3, 119.1, 49.5, 40.5, 28.4; **HRMS** (ESI-TOF) calculated for $C_{10}H_{14}NO_3{}^{32}S$ [M+H]$^+$: 228.0689; found 228.0693; **m.p.**: 120 - 122 °C; **IR** (neat) 2981, 2889, 1671, 1598, 1543, 1503, 1491, 1448, 1431, 1382, 1296, 1269, 1249, 1190, 1130, 1081, 1026, 995, 057, 918, 813, 753, 700, 676.

3-((chloromethyl)sulfonyl)-*N*-phenylpropanamide (3l)

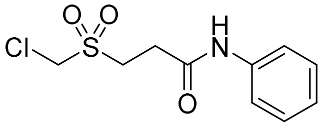

General procedure A was followed to obtain **3l** (81 mg, 0.31 mmol, 62%) as a white solid.
^{1}H NMR (400 MHz, DMSO-d_6) δ 10.15 (s, 1H), 7.60 - 7.55 (m, 2H), 7.34 - 7.27 (m, 2H), 7.08 - 7.02 (m, 1H), 5.15 (s, 2H), 3.58 (t, J = 7.4 Hz, 2H), 2.86 (t, J = 7.5 Hz, 2H); **^{13}C NMR** (101 MHz, DMSO-d_6) δ 167.5, 138.9, 128.8, 123.4, 119.1, 55.8, 45.7, 28.2; **HRMS** (ESI-TOF) calculated for $C_{10}H_{13}ClNO_3{}^{32}S$ [M+H]$^+$: 262.0299; found 262.0299; **m.p.**: 141 - 145 °C; **IR** (neat) 2981, 2889, 1676, 1604, 1544, 1489, 1440, 1392, 1307, 1277, 1250, 1160, 1121, 1081, 1050, 1024, 991, 954, 878, 827, 798, 766, 745, 699, 676.

Benzyl 3-((3-oxo-3-(phenylamino)propyl)sulfonyl)azetidine-1-carboxylate (3m)

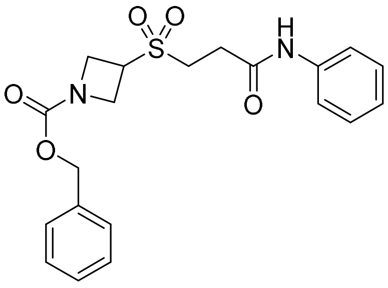

General procedure A was followed to obtain **3m** (195 mg, 0.49 mmol, 97%) as a white solid. **^{1}H NMR** (400 MHz, DMSO-d_6) δ 10.12 (br s, 1H), 7.59 - 7.54 (m, 2H), 7.40 - 7.27 (m, 7H), 7.08 - 7.01 (m, 1H), 5.06 (s, 2H), 4.41- 4.32 (m, 1H), 4.31 - 4.19 (br s, 2H), 4.18 - 4.03 (br s, 2H), 3.47 (t, J = 7.4 Hz, 2H), 2.80 (t, J = 7.4 Hz, 2H) ; **^{13}C NMR** (101 MHz, DMSO-d_6) δ 167.6, 155.6, 138.9, 136.5, 128.8, 128.4, 128.0, 127.7, 123.4, 119.1, 66.1, 49.5 (br s), 49.0 (br s), 48.2, 46.4, 28.3; **HRMS** (ESI-TOF) calculated for $C_{20}H_{23}N_2O_5{}^{32}S$ $[M+H]^+$: 403.1322; found 403.1327; **m.p.**: 146 - 148 °C; **IR** (neat) 2981, 2889, 1704, 1663, 1601, 1545, 1440, 1408, 1381, 1356, 1316, 1299, 1284, 1254, 1202, 1138, 1115, 1080, 1054, 1026, 998, 968, 951, 820, 758, 739, 696, 673, 629, 608.

***N*-phenyl-3-((tetrahydrofuran-3-yl)sulfonyl)propanamide (3n)**

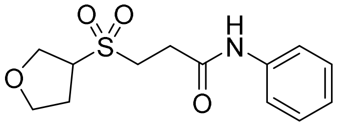

General procedure A was followed to obtain **3n** (115 mg, 0.41 mmol, 81%) as a white solid. **^{1}H NMR** (500 MHz, $CDCl_3$) δ 7.92 (s, 1H), 7.49 (d, J = 7.3 Hz, 2H), 7.34 - 7.29 (m, 2H), 7.14 - 7.09 (m, 1H), 4.25 (dd, J = 10.2, 5.1 Hz, 1H), 4.06 (dd, J = 10.2, 7.9 Hz, 1H), 4.03 - 3.97 (m, 1H), 3.85 - 3.79 (m, 1H), 3.78 - 3.71 (m, 1H), 3.46 - 3.38 (m, 2H), 2.96 (t, J = 7.2 Hz, 2H), 2.45 - 2.36 (m, 1H), 2.35 - 2.28 (m, 1H); **^{13}C NMR** (126 MHz, $CDCl_3$) δ 167.5, 137.7, 129.2, 124.8, 120.1, 68.5, 67.2, 61.9, 47.4, 28.7, 27.3; **HRMS** (ESI-TOF) calculated for $C_{13}H_{18}NO_4{}^{32}S$ $[M+H]^+$: 284.0951; found 284.0956; **m.p.**: 125 - 128 °C; **IR** (neat) 2981, 2888, 1660, 1600, 1541, 1443, 1382, 1302, 1252, 1137, 1075, 968, 924, 742, 717, 691.

3-((((1*S*)-7,7-dimethyl-2-oxobicyclo[2.2.1]heptan-1-yl)methyl)sulfonyl)-*N*-phenylpropanamide (3o)

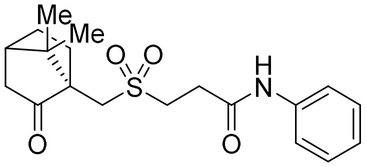

General procedure A was followed to obtain **3o** (163 mg, 0.45 mmol, 90%) as a white solid. **^{1}H NMR** (400 MHz, $CDCl_3$) δ 8.40 (s, 1H), 7.56 - 7.51 (m, 2H), 7.31 - 7.25 (m, 2H), 7.11 - 7.05 (m, 1H), 3.61 (ddd, J = 7.8, 6.7, 3.2 Hz, 2H), 3.53 (d, J = 14.9 Hz, 1H), 3.07 - 2.91 (m, 2H), 2.86 (d, J = 14.9 Hz, 1H), 2.45 - 2.32 (m, 2H), 2.11 (t, J = 4.5 Hz, 1H), 2.06 - 1.96 (m, 1H), 1.92 (d, J = 18.5 Hz, 1H), 1.79 (ddd, J = 14.0, 9.3, 4.7 Hz, 1H), 1.41 (td, J = 9.2, 4.7 Hz, 1H), 1.02 (s, 3H), 0.85 (s, 3H); **^{13}C NMR** (101 MHz, $CDCl_3$) δ 215.4, 167.8, 138.0, 129.0, 124.4, 120.1, 58.8, 51.3, 50.9, 48.8, 42.7, 42.5, 29.7, 27.1, 24.9, 19.7, 19.6; **HRMS** (ESI-TOF) calculated for $C_{19}H_{26}NO_4{}^{32}S$ $[M+H]^+$: 364.1577; found 364.1584; **IR** (neat) 2980, 2889, 1704, 1667, 1599, 1545, 1491, 1462, 1441, 1381, 1299, 1251, 1132, 1082, 952, 879, 816, 740, 698, 673.

3-((4-fluorophenyl)sulfonyl)-1-phenylpropan-1-one (3p)

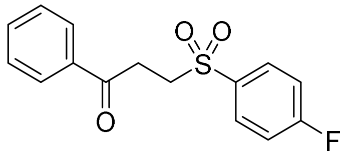

General procedure A was followed to obtain **3p** (129 mg, 0.44 mmol, 88%) as a white solid. **^{1}H NMR** (500 MHz, $CDCl_3$) δ 8.02 - 7.97 (m, 2H), 7.96 - 7.92 (m, 2H), 7.65 - 7.60 (m, 1H), 7.52 - 7.48 (m, 2H), 7.30 - 7.24 (m, 2H), 3.59 (ddd, J = 8.3, 6.4, 1.8 Hz, 2H), 3.53 (ddd, J = 8.3, 6.4, 1.8 Hz, 2H); **^{13}C NMR** (126 MHz, $CDCl_3$) δ 195.4, 166.1 (d, $J_{C\text{-}F}$ = 256.9 Hz), 135.9, 135.3 (d, $J_{C\text{-}F}$ = 3.2 Hz), 134.0, 131.1 (d, $J_{C\text{-}F}$ = 9.5 Hz), 129.0, 128.2, 116.9 (d, $J_{C\text{-}F}$ = 22.6 Hz), 51.3, 31.5; **^{19}F NMR** (471 MHz, $CDCl_3$) δ -102.9 - -103.0 (m, 1F); **HRMS** (ESI-TOF) calculated for $C_{15}H_{14}FO_3{}^{32}S$ $[M+H]^+$: 293.0642; found 293.0646; **m.p.**: 143 - 145 °C; **IR** (neat) 1679, 1590, 1493, 1415, 1356, 1315, 1290, 1270, 1258, 1235, 1200, 1161, 1143, 1131, 1084, 1059, 1014, 973, 946, 832, 817, 743, 695, 673, 637.

Phenyl 3-((4-fluorophenyl)sulfonyl)propanoate (3q)

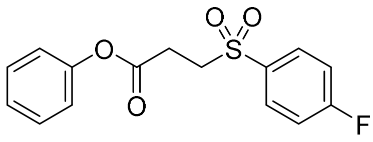

General procedure A was followed to obtain **3q** (146 mg, 0.48 mmol, 95%) as a white solid. **^{1}H NMR** (400 MHz, $CDCl_3$) δ 8.00 - 7.93 (m, 2H), 7.39 - 7.32 (m, 2H), 7.29 - 7.19 (m, 3H), 7.06 - 7.01 (m, 2H), 3.53 (t, J = 7.5 Hz, 2H), 3.00 (t, J = 7.5 Hz, 2H); **^{13}C NMR** (101 MHz, $CDCl_3$) δ 168.7, 166.1 (d, $J_{C\text{-}F}$ = 257.1 Hz), 150.4, 134.7 (d, $J_{C\text{-}F}$ = 3.2 Hz), 131.2 (d, $J_{C\text{-}F}$ = 9.7 Hz), 129.6, 126.3, 121.3, 116.9 (d, $J_{C\text{-}F}$ = 22.6 Hz), 51.7, 28.1; **^{19}F NMR** (471 MHz, $CDCl_3$) δ -102.6 - -102.7 (m, 1F); **HRMS** (ESI-TOF) calculated for $C_{15}H_{14}FO_4{}^{32}S$ $[M+H]^+$: 309.0591; found 309.0593; **m.p.**: 104 - 106 °C; **IR** (neat) 2981, 2889, 1750, 1591, 1492, 1462, 1381, 1310, 1291, 1251, 1193, 1155, 1085, 1022, 954, 902, 819, 803, 773, 751, 713, 697.

3-((4-fluorophenyl)sulfonyl)-*N*-(pyridin-3-yl)propenamide (3r)

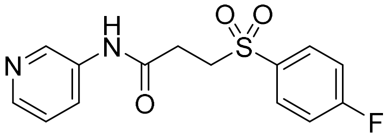

General procedure A was followed to obtain **3r** (85 mg, 0.28 mmol, 55%) as a white solid. **^{1}H NMR** (400 MHz, DMSO-d_6) δ 10.26 (s, 1H), 8.63 (dd, J = 2.7, 0.8 Hz, 1H), 8.24 (dd, J = 4.7, 1.5 Hz, 1H), 8.03 - 7.96 (m, 2H), 7.91 (ddd, J = 8.3, 2.6, 1.5 Hz, 1H), 7.52 - 7.44 (m, 2H), 7.31 (ddd, J = 8.4, 4.7, 0.8 Hz, 1H), 3.65 (t, J = 7.4 Hz, 2H), 2.72 (t, J = 7.4 Hz, 2H); **^{13}C NMR** (101 MHz, DMSO-d_6) δ 167.9, 165.2 (d, $J_{C\text{-}F}$ = 252.9 Hz), 144.3, 140.7, 135.5, 134.9 (d, $J_{C\text{-}F}$ = 3.0 Hz), 131.17 (d, $J_{C\text{-}F}$ = 9.9 Hz), 126.0, 123.6, 116.7 (d, $J_{C\text{-}F}$ = 22.8 Hz), 50.7, 29.5; **^{19}F NMR** (377 MHz, DMSO-d_6) δ -102.4 - -102.5 (m, 1F); **HRMS** (ESI-TOF) calculated for $C_{14}H_{14}FN_2O_3{}^{32}S$ $[M+H]^+$: 309.0704; found 309.0714; **m.p.**: 156 - 159 °C; **IR** (neat) 1589, 1496, 1446, 1308, 1291, 1222, 1149, 1120, 1099, 1083, 1012, 839, 808, 767, 749, 733, 706, 686.

N-(benzo[d]thiazol-2-yl)-3-((4-fluorophenyl)sulfonyl)propenamide (3s)

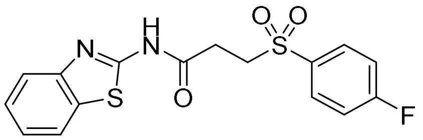

General procedure A was followed to obtain **3s** (158 mg, 0.43 mmol, 87%) as a white solid. **^{1}H NMR** (400 MHz, DMSO-d_6) δ 8.02 - 7.97 (m, 2H), 7.97 - 7.93 (m, 1H), 7.74 - 7.71 (m, 1H), 7.53 - 7.46 (m, 2H), 7.45 - 7.39 (m, 1H), 7.32 - 7.26 (m, 1H), 3.70 (t, J = 7.2 Hz, 2H), 2.88 (t, J = 7.3 Hz, 2H); **^{13}C NMR** (101 MHz, DMSO-d_6) δ 168.7, 165.2 (d, $J_{C\text{-}F}$ = 252.9 Hz), 157.7, 148.5, 134.8 (d, $J_{C\text{-}F}$ = 2.9 Hz), 131.4, 131.2 (d, $J_{C\text{-}F}$ = 9.9 Hz), 126.1, 123.6, 121.7, 120.6, 116.7 (d, $J_{C\text{-}F}$ = 22.9 Hz), 50.3, 28.8; **^{19}F NMR** (471 MHz, DMSO-d_6) δ -104.6 - -104.7 (m, 1F); **HRMS** (ESI-TOF) calculated for $C_{16}H_{12}FN_2O_3{}^{32}S_2$ [M-H]$^-$: 363.0279; found 363.0278; **m.p.**: 200 -204 °C; **IR** (neat) 1698, 1592, 1546, 1492, 1455, 1445, 1319, 1288, 1268, 1238, 1149, 1086, 1046, 1024, 983, 877, 832, 759, 727, 706, 667.

1-fluoro-4-((2-(phenylsulfonyl)ethyl)sulfonyl)benzene (3t)

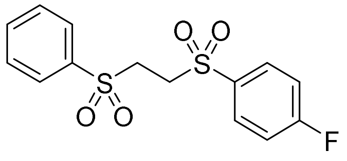

General procedure A was followed to obtain **3t** (112 mg, 0.34 mmol, 68%) as a white solid. **^{1}H NMR** (500 MHz, $CDCl_3$) δ 7.92 - 7.86 (m, 4H), 7.73 - 7.69 (m, 1H), 7.62 - 7.57 (m, 2H), 7.29 - 7.24 (m, 2H), 3.45 (s, 4H); **^{13}C NMR** (126 MHz, $CDCl_3$) δ 166.4 (d, $J_{C\text{-}F}$ = 258.2 Hz), 138.1, 134.7, 134.2 (d, $J_{C\text{-}F}$ = 3.2 Hz), 131.2 (d, $J_{C\text{-}F}$ = 9.7 Hz), 129.9, 128.2, 117.2 (d, $J_{C\text{-}F}$ = 22.9 Hz), 49.7, 49.5; **^{19}F NMR** (471 MHz, $CDCl_3$) δ -101.5 - -101.6 (m, 1F); **HRMS** (ESI-TOF) calculated for $C_{14}H_{13}FNaO_4{}^{32}S_2$ [M+H]$^+$: 351.0131; found 351.0132; **m.p.**: 177 - 178 °C; **IR** (neat) 1589, 1496, 1446, 1308, 1291, 1222, 1149, 1120, 1099, 1083, 1012, 839, 808, 767, 749, 733, 706, 686. Compound **3t** was found to be unstable under various ionization techniques and HRMS could therefore not be obtained.

Methyl 3-((4-fluorophenyl)sulfonyl)-2-methylpropanoate (3u)

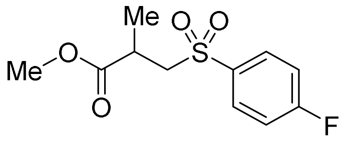

General procedure A was followed to obtain **3u** (101 mg, 0.39 mmol, 78%) as a white solid. ^{1}**H NMR** (400 MHz, $CDCl_3$) δ 7.93 - 7.85 (m, 2H), 7.25 - 7.18 (m, 2H), 3.66 (dd, J = 14.2, 7.7 Hz, 1H), 3.59 (s, 3H), 3.06 (dd, J = 14.2, 5.1 Hz, 1H), 3.02 - 2.92 (m, 1H), 1.28 (d, J = 7.1 Hz, 3H); ^{13}**C NMR** (101 MHz, $CDCl_3$) δ 173.9, 166.0 (d, J_{C-F} = 256.6 Hz), 135.3 (d, J_{C-F} = 3.2 Hz), 131.1 (d, J_{C-F} = 9.7 Hz), 116.7 (d, J_{C-F} = 22.7 Hz), 58.9, 52.4, 34.8, 17.9; ^{19}**F NMR** (471 MHz, $CDCl_3$) δ -103.1 - -103.2 (m, 1F); **HRMS** (ESI-TOF) calculated for $C_{11}H_{14}FO_4{}^{32}S$ $[M+H]^+$: 261.0591; found 261.0602; **IR** (neat) 1735, 1591, 1493, 1459, 1405, 1320, 1290, 1230, 1141, 1085, 837, 758, 673.

3-((4-fluorophenyl)sulfonyl)-1,3-diphenylpropan-1-one (3v)

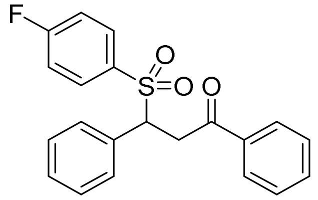

General procedure A was followed to obtain **3v** (162 mg, 0.44 mmol, 88%) as a white solid. ^{1}**H NMR** (400 MHz, $CDCl_3$) δ 7.97 - 7.92 (m, 2H), 7.61 - 7.50 (m, 3H), 7.49 - 7.43 (m, 2H), 7.29 - 7.17 (m, 5H), 7.08 - 7.01 (m, 2H), 4.93 (dd, J = 9.6, 3.6 Hz, 1H), 4.14 (dd, J = 17.9, 3.6 Hz, 1H), 3.93 (dd, J = 17.9, 9.6 Hz, 1H); ^{13}**C NMR** (101 MHz, $CDCl_3$) δ 194.9, 165.9 (d, J_{C-F} = 256.6 Hz), 136.2, 133.9, 133.1 (d, J_{C-F} = 3.2 Hz), 132.6, 132.0 (d, J_{C-F} = 9.7 Hz), 129.9 129.1, 128.9, 128.7, 128.3, 116.2 (d, J_{C-F} = 22.6 Hz), 66.8, 36.9; ^{19}**F NMR** (471 MHz, $CDCl_3$) δ -103.1 - -103.2 (m, 1F); **HRMS** (ESI-TOF) calculated for $C_{21}H_{18}FO_3{}^{32}S$ $[M+H]^+$: 369.0955; found 369.0962; **m.p.**: 170 - 172 °C; **IR** (neat) 2981, 2888, 1688, 1591, 1492, 1449, 1382, 1314, 1289, 1235, 1142, 1085, 1015, 957, 832, 817, 778, 750, 730, 698, 688, 672, 641, 615.

3-((4-fluorophenyl)sulfonyl)cyclohexan-1-one (3w)

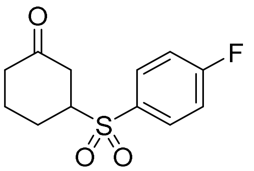

General procedure A was followed to obtain **3w** (97 mg, 0.36 mmol, 71%) as a white solid. **^{1}H NMR** (400 MHz, $CDCl_3$) δ 7.93 - 7.87 (m, 2H), 7.39- 7.32 (m, 2H), 3.36 - 3.25 (m, 1H), 2.64 - 2.54 (m, 2H), 2.47 - 2.39 (m, 1H), 2.37 - 2.18 (3H), 1.99 - 1.86 (m, 1H), 1.72 - 1.59 (m, 1H); **^{13}C NMR** (101 MHz, $CDCl_3$) δ 206.2, 166.2 (d, $J_{C\text{-}F}$ = 257.4 Hz), 132.8 (d, $J_{C\text{-}F}$ = 3.2 Hz), 131.9 (d, $J_{C\text{-}F}$ = 9.7 Hz), 116.9 (d, $J_{C\text{-}F}$ = 22.7 Hz), 62.5, 40.5, 40.4, 23.8, 23.4; **^{19}F NMR** (471 MHz, $CDCl_3$) δ -102.4 - -102.5 (m, 1F); **HRMS** (ESI-TOF) calculated for $C_{12}H_{17}FNO_3{}^{32}S$ $[M+NH_4]^+$: 274.0908; found 274.0910; **m.p.**: 84 - 87 °C; **IR** (neat) 1713, 1590, 1493, 1311, 1286, 1267, 1227, 1138, 1084, 1058, 843, 818, 776, 720, 674.

3-((4-fluorophenyl)sulfonyl)cyclopentan-1-one (3x)

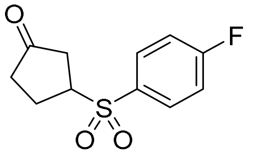

General procedure A was followed to obtain **3x** (92 mg, 0.38 mmol, 76%) as a white solid. **^{1}H NMR** (400 MHz, $CDCl_3$) δ 7.98 - 7.90 (m, 2H), 7.34 - 7.25 (m , 2H), 3.76 (p, J = 7.7 Hz, 1H), 2.72 - 2.62 (m, 1H), 2.60 - 2.37 (m, 3H), 2.35 - 2.20 (m, 2H); **^{13}C NMR** (101 MHz, $CDCl_3$) δ 212.5, 166.3 (d, $J_{C\text{-}F}$ = 257.5 Hz), 133.6 (d, $J_{C\text{-}F}$ = 3.3 Hz), 131.6 (d, $J_{C\text{-}F}$ = 9.6 Hz), 117.1 (d, $J_{C\text{-}F}$ = 22.6 Hz), 61.0, 38.7, 37.0, 23.3; **^{19}F NMR** (471 MHz, $CDCl_3$) δ -102.3 - -102.4 (m, 1F);**HRMS** (ESI-TOF) calculated for $C_{11}H_{11}O_3F^{23}Na^{32}S$ $[M+Na]^+$: 265.0305; found 265.0307; **m.p.**: 99 - 101 °C; **IR** (neat) 2981, 1738, 1700, 1589, 1493, 1473, 1462, 1383, 1323, 1287, 1253, 1227, 1141, 1081, 1014, 954, 903, 850, 837, 819, 789, 754, 124, 691, 655, 635.

1-benzyl-3-((4-fluorophenyl)sulfonyl)pyrrolidine-2,5-dione (3y)

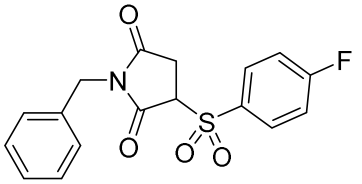

General procedure A was followed to obtain **3y** (130 mg, 0.38 mmol, 75%) as a white solid.
^{1}H NMR (500 MHz, $CDCl_3$) δ 7.86 - 7.81 (m, 2H), 7.30 - 7.24 (m, 5H), 7.20 - 7.14 (m, 2H), 4.59 (d, J = 4.5 Hz, 2H), 4.30 (dd, J = 9.6, 3.8 Hz, 1H), 3.32 (dd, J = 19.1, 3.8 Hz, 1H), 3.07 (dd, J = 19.1, 9.6 Hz, 1H); **^{13}C NMR** (101 MHz, $CDCl_3$) δ 172.5, 168.4, 166.6 (d, $J_{C\text{-}F}$ = 258.7 Hz), 134.7, 132.5 (d, $J_{C\text{-}F}$ = 10.0 Hz), 132.3 (d, $J_{C\text{-}F}$ = 3.2 Hz), 128.8, 128.8, 128.4, 116.9 (d, $J_{C\text{-}F}$ = 22.8 Hz), 63.6, 43.2, 30.0; **^{19}F NMR** (471 MHz, $CDCl_3$) δ -100.7 - -100.8 (m, 1F); **HRMS** (ESI-TOF) calculated for $C_{17}H_{13}FO_4N^{32}S$ $[M-H]^-$: 346.0555; found 346.0550; **m.p.**: 181 - 183 °C; **IR** (neat) 2981, 2914, 1699, 1588, 1492, 1447, 1399, 1356, 1324, 1293, 1237, 1214, 1157, 1136, 1084, 1011, 955, 908, 836, 788, 754, 701, 655, 632, 614.

***trans*-2-((4-fluorophenyl)sulfonyl)-*N*-phenylcyclopentane-1-carboxamide (*trans*-3z)**

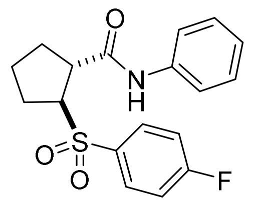

General procedure A was followed to obtain ***trans*-3z** (113 mg, 0.33 mmol, 65%) as a white solid.
^{1}H NMR (500 MHz, DMSO-d_6) δ 9.95 (s, 1H), 7.98 - 7.92 (m, 2H), 7.44 - 7.36 (m, 4H), 7.29 - 7.23 (m, 2H), 7.04 - 7.00 (m, 1H), 4.09 (dt, J = 9.1, 7.2 Hz, 1H), 3.23 (dt, J = 9.1, 8.1 Hz, 1H), 2.16 - 2.07 (m, 1H), 2.06 - 1.97 (m, 2H), 1.79 - 1.71 (m, 1H), 1.71 - 1.59 (m, 2H); **^{13}C NMR** (126 MHz, DMSO-d_6) δ 170.6, 165.1 (d, $J_{C\text{-}F}$ = 253.1 Hz), 138.7, 134.4 (d, $J_{C\text{-}F}$ = 3.0 Hz), 131.5 (d, $J_{C\text{-}F}$ = 9.9 Hz), 128.6, 123.3, 119.1, 116.6 (d, $J_{C\text{-}F}$ = 22.8 Hz), 65.2, 46.8, 32.8, 27.0, 25.6; **^{19}F NMR** (471 MHz, DMSO-d_6) δ -104.7 - -104.8 (m); **HRMS** (ESI-TOF) calculated for $C_{18}H_{19}FO_3N^{32}S$ $[M+H]^+$: 348.1064; found 348.1070; **m.p.**: 175 - 178 °C; **IR** (neat) 1727, 1589, 1493, 1452, 1408,

1365, 1319, 1295, 1225, 1174, 1157, 1144, 1099, 1082, 1024, 955, 857, 844, 832, 820, 732, 709, 697, 647.

***cis*-2-((4-fluorophenyl)sulfonyl)-*N*-phenylcyclopentane-1-carboxamide (*cis*-3z)**

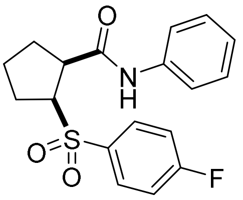

General procedure A was followed to obtain ***cis*-3z** (61 mg, 0.18 mmol, 35%) as a white solid. **^{1}H NMR** (400 MHz, DMSO-*d$_6$*) δ 9.77 (s, 1H), 7.88 - 7.82 (m, 2H), 7.50 - 7.45 (m, 2H), 7.37 - 7.30 (m, 2H), 7.29 - 7.23 (m, 2H), 7.05 - 6.99 (m, 1H), 4.09 (dt, J = 7.7 Hz, 1H), 3.16 (dt, J = 7.1 Hz, 1H), 2.34 - 2.21 (m, 1H), 2.04 - 1.76 (m, 4H), 1.71 - 1.58 (m, 1H); **^{13}C NMR** (101 MHz, DMSO-*d$_6$*) δ 169.0, 164.9 (d, $J_{C\text{-}F}$ = 252.3 Hz), 139.0, 135.7 (d, $J_{C\text{-}F}$ = 2.9 Hz), 131.2 (d, $J_{C\text{-}F}$ = 9.9 Hz), 128.4, 123.0, 119.2, 116.3 (d, $J_{C\text{-}F}$ = 22.9 Hz), 66.6, 46.8, 28.2, 26.3, 22.2; **^{19}F NMR** (471 MHz, DMSO-*d$_6$*) δ -105.2 - -105.3 (m, 1F); **HRMS** (ESI-TOF) calculated for $C_{18}H_{19}FO_3N^{32}S$ $[M+H]^+$: 348.1064; found 348.1069; **m.p.**: 188 - 191 °C; **IR** (neat) 1662, 1590, 1547, 1491, 1445, 1395, 1317, 1292, 1253, 1238, 1203, 1193, 1145, 1129, 1098, 1080, 1013, 963, 932, 905, 885, 850, 823, 792, 755, 722, 695, 632, 616.

***cis*-2-((4-fluorophenyl)sulfonyl)cyclobutyl)(pyrrolidin-1-yl)methanone (3aa)**

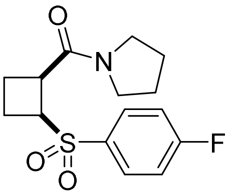

General procedure A was followed to obtain **3aa** (96 mg, 0.31 mmol, 62%) as a white solid. **^{1}H NMR** (500 MHz, DMSO-*d$_6$*) δ 7.92 - 7.86 (m, 2H), 7.52 - 7.46 (m, 2H), 4.49 - 4.43 (m, 1H), 3.65 (ddd, J = 8.8 Hz, 1H), 3.28 - 3.19 (m, 2H), 3.09 - 3.02 (m, 1H), 2.96 - 2.90 (m, 1H), 2.65 - 2.55 (m, 1H), 2.28 - 2.18 (m, 2H), 1.83 - 1.76 (m, 1H), 1.76 - 1.68 (m, 1H), 1.68 - 1.61 (m, 1H), 1.61 - 1.53 (m, 1H), 1.53 - 1.44 (m, 1H); **^{13}C NMR** (126 MHz, DMSO-*d$_6$*) δ 166.0, 165.4 (d, J =

252.7 Hz), 134.8 (d, J = 3.0 Hz), 131.5 (d, J = 9.8 Hz), 116.4 (d, J = 22.7 Hz), 59.3, 45.2, 45.1, 39.4, 25.2, 23.5, 20.7, 20.6; **^{19}F NMR** (471 MHz, DMSO-d_6) δ -104.9 - -104.9 (m, 1F); **HRMS** (ESI-TOF) calculated for $C_{15}H_{19}FO_3N^{32}S$ $[M+H]^+$: 312.1064; found 312.1069; **m.p.**: 155 - 159 °C; **IR** (neat) 2981, 2889, 1737, 1702, 1617, 1590, 1493, 1380, 1349, 1316, 1292, 1231, 1193, 1141, 1084, 1048, 1024, 996, 822, 763, 752, 718, 690, 669.

Benzyl-*cis*-2-((4-fluorophenyl)sulfonyl)cyclobutane-1-carboxylate (3ab)

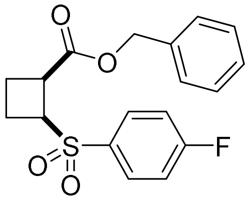

General procedure A was followed to obtain **3ab** (103 mg, 0.30 mmol, 59%) as a white solid. **^{1}H NMR** (500 MHz, $CDCl_3$) δ 7.88 - 7.82 (m, 2H), 7.45 - 7.41 (m, 2H), 7.40 - 7.32 (m, 3H), 7.17 - 7.11 (m, 2H), 5.29 (d, J = 12.2 Hz, 1H), 5.15 (d, J = 12.1 Hz, 1H), 4.08 (dt, J = 9.1, 8.4 Hz, 1H), 3.51 - 3.43 (m, 1H), 2.76 - 2.66 (m, 1H), 2.63 - 2.54 (m, 1H), 2.23 - 2.14 (m, 1H), 2.14 - 2.05 (m, 1H); **^{13}C NMR** (126 MHz, $CDCl_3$) δ 170.4, 165.9 (d, J = 256.4 Hz), 135.6, 134.9 (d, J = 3.2 Hz), 131.3 (d, J = 9.6 Hz), 128.8, 128.7, 128.5, 116.5 (d, J = 22.5 Hz), 67.7, 59.2, 40.9, 22.5, 20.6; **^{19}F NMR** (471 MHz, $CDCl_3$) δ -103.4 - -103.5 (m, 1F); **HRMS** (ESI-TOF) calculated for $C_{18}H_{21}FO_4N^{32}S$ $[M+NH_4]^+$: 366.1170; found 366.1173; **m.p.**: 114-118 °C; **IR** (neat) 2981, 2889, 1726, 1586, 1383, 1349, 1317, 1269, 1251, 1233, 1181, 1145, 1081, 1030, 1009, 956, 847, 821, 772, 752, 740, 704, 673, 654, 606.

2-phenyl-*trans*-2-((4-fluorophenyl)sulfonyl)cyclobutane-1-carboxylate (*trans*-3ac)

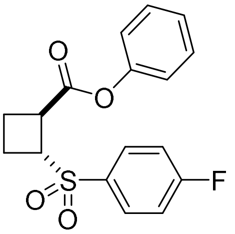

General procedure A was followed to obtain ***trans*-3ac** (8 mg, 0.03 mmol, 5%) as a white solid.

^{1}**H NMR** (500 MHz, $CDCl_3$) δ 7.98 - 7.92 (m, 2H), 7.37 - 7.31 (m, 2H), 7.25 - 7.19 (m, 3H), 6.88 - 6.83 (m, 2H), 4.24 - 4.15 (m, 1H), 3.84 - 3.75 (m, 1H), 2.72 - 2.61 (m, 1H), 2.51 - 2.41 (m, 1H), 2.38 - 2.23 (m, 2H); ^{13}**C NMR** (126 MHz, $CDCl_3$) δ 170.3, 166.2 (d, $J_{C\text{-}F}$ = 257.0 Hz), 150.3, 133.9 (d, $J_{C\text{-}F}$ = 3.2 Hz), 131.5 (d, $J_{C\text{-}F}$ = 9.7 Hz), 129.6, 126.3, 121.2, 116.9 (d, $J_{C\text{-}F}$ = 22.6 Hz), 58.5, 39.2, 21.3, 19.4; ^{19}**F NMR** (471 MHz, $CDCl_3$) δ -102.9 - -103.0 (m, 1F); **HRMS** (ESI-TOF) calculated for $C_{17}H_{14}FO_4{}^{32}S$ $[M-H]^-$: 333.0602; found 333.0596 ; **IR** (neat) 2981, 2889, 1738, 1588, 1492, 1461, 1382, 1345, 1317, 1294, 1267, 1232, 1192, 1161, 1138, 1085, 1046, 1015, 982, 954, 913, 879, 843, 819, 764, 743, 719, 689, 650.

2-phenyl-*cis*-2-((4-fluorophenyl)sulfonyl)cyclobutane-1-carboxylate (*cis*-3ac)

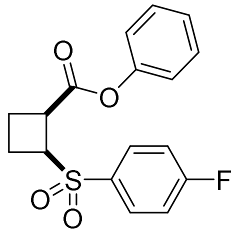

General procedure A was followed to obtain ***cis*-3ac** (82 mg, 0.25 mmol, 49%) as a white solid. ^{1}**H NMR** (500 MHz, $CDCl_3$) δ 7.94 - 7.89 (m, 2H), 7.42 - 7.38 (m, 2H), 7.31 - 7.28 (m, 2H), 7.27 - 7.22 (m, 1H), 7.21 - 7.16 (m, 2H), 4.24 - 4.15 (m, 1H), 3.73 - 3.65 (m, 1H), 2.78 2.63 (m, 2H), 2.28 - 2.15 (m, 2H); ^{13}**C NMR** (126 MHz, $CDCl_3$) δ 169.2, 166.0 (d, $J_{C\text{-}F}$= 256.5 Hz), 150.9, 134.7 (d, $J_{C\text{-}F}$ = 3.1 Hz), 131.4 (d, $J_{C\text{-}F}$ = 9.6 Hz), 129.5, 126.1, 121.8, 116.6 (d, $J_{C\text{-}F}$ = 22.7 Hz), 59.4, 40.7, 22.4, 20.6; ^{19}**F NMR** (471 MHz, $CDCl_3$) δ -103.2 - -103.3 (m, 1F); **HRMS** (ESI-TOF) calculated for $C_{17}H_{14}FO_4{}^{32}S$ $[M-H]^-$: 333.0602; found 333.0598 ; **m.p.**: 133 - 135 °C; **IR** (neat) 2978, 1726, 1541, 1486, 1453, 1369, 1356, 1308, 1261, 1177, 1123, 1092, 1058, 1040, 993, 950, 893, 817, 788, 756, 655, 621.

Methyl 2-(1,3-dioxoisoindolin-2-yl)-3-((4-fluorophenyl)sulfonyl)propanoate (3ad)

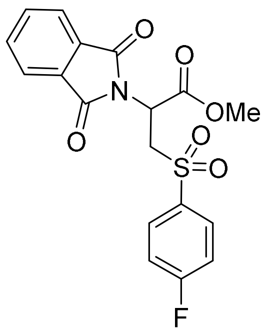

General procedure A was followed to obtain **3ad** (119 mg, 0.31 mmol, 61%) as a white solid. ^{1}**H NMR** (400 MHz, $CDCl_3$) δ 7.90 - 7.83 (m, 2H), 7.83 - 7.78 (m, 2H), 7.77 - 7.71 (m, 2H), 7.11 - 7.03 (m, 2H), 5.33 (dd, J = 11.5, 2.4 Hz, 1H), 4.29 (dd, J = 15.2, 11.5 Hz, 1H), 3.98 (dd, J = 15.2, 2.5 Hz, 1H), 3.72 (s, 3H); ^{13}**C NMR** (101 MHz, $CDCl_3$) δ 167.2, 167.2, 165.8 (d, $J_{C\text{-}F}$ = 224.8 Hz), 134.6, 134.5 (d, $J_{C\text{-}F}$ = 3.1 Hz), 131.5, 131.2 (d, $J_{C\text{-}F}$ = 9.8 Hz), 123.8, 116.7 (d, $J_{C\text{-}F}$ = 22.8 Hz), 53.8, 53.1, 46.9; ^{19}**F NMR** (471 MHz, $CDCl_3$) δ -102.6 - -102.7 (m, 1F); **HRMS** (ESI-TOF) calculated for $C_{18}H_{15}FNO_6{}^{32}S$ $[M+H]^+$: 392.0599; found 392.0599; **m.p.**: 155 - 159 °C; **IR** (neat) 2981, 2889, 1775, 1735, 1718, 1589, 1492, 1470, 1438, 1383, 1309, 1291, 1256, 1142, 1085, 1072, 1014, 1002, 956, 911, 873, 853, 839, 820, 806, 787, 756, 712, 656.

Ethyl (*E*)-3-((4-fluorophenyl)sulfonyl)acrylate ((*E*)-3ae)

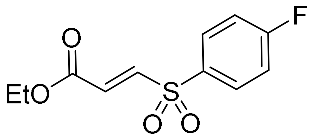

General procedure B was followed to obtain **(*E*)-3ae** (14 mg, 0.04 mmol, 8%) as a colourless oil. ^{1}**H NMR** (400 MHz, $CDCl_3$) δ 7.91 - 7.84 (m, 2H), 7.24 - 7.15 (m, 3H), 6.77 (d, J = 15.2 Hz, 1H), 4.19 (q, J = 7.1 Hz, 2H), 1.24 (t, J = 7.1 Hz, 3H); ^{13}**C NMR** (101 MHz, $CDCl_3$) δ 166.4 (d, $J_{C\text{-}F}$ = 257.9 Hz), 163.5, 143.1, 134.7 (d, $J_{C\text{-}F}$ = 3.2 Hz), 131.5 (d, $J_{C\text{-}F}$ = 9.8 Hz), 131.4, 117.2 (d, $J_{C\text{-}F}$ = 22.7 Hz), 62.3, 14.2; ^{19}**F NMR** (471 MHz, $CDCl_3$) δ -101.8 - -101.9 (m, 1F); **HRMS** (ESI-TOF) calculated for $C_{11}H_{12}FO_4{}^{32}S$ $[M+H]^+$: 359.0435; found 359.0436; **IR** (neat) 1787, 1632, 1486,

1328, 1305, 1289, 1271, 1225, 1196, 1111, 1093, 1039, 963, 905, 857, 842, 805, 784, 753, 688, 631.

Ethyl (*Z*)-3-((4-fluorophenyl)sulfonyl)acrylate ((*Z*)-3ae)

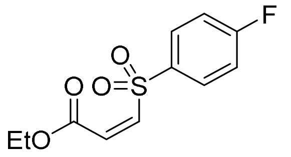

General procedure B was followed to obtain **(*Z*)-3ae** (112 mg, 0.31 mmol, 62%) as a white solid. **^{1}H NMR** (400 MHz, $CDCl_3$) δ 8.07 - 8.00 (m, 2H), 7.28 - 7.21 (m, 2H), 6.53 (s, 2H), 4.36 (q, J = 7.2 Hz, 2H), 1.39 (t, J = 7.2 Hz, 3H); **^{13}C NMR** (101 MHz, $CDCl_3$) δ 167.4, 164.5 (d, $J_{C\text{-}F}$ = 87.0 Hz), 135.6 (d, $J_{C\text{-}F}$ = 3.1 Hz), 135.2, 132.3, 131.4 (d, $J_{C\text{-}F}$ = 9.6 Hz), 116.8 (d, $J_{C\text{-}F}$ = 22.6 Hz), 62.4, 14.1; **^{19}F NMR** (471 MHz, $CDCl_3$) δ -102.7 - 102.7 (m, 1F); **HRMS** (ESI-TOF) calculated for $C_{11}H_{12}FO_4{}^{32}S$ $[M+H]^+$: 359.0435; found 359.0439; **m.p.**: 99 - 101 °C; **IR** (neat) 1726, 1625, 1589, 1494, 1367, 1340, 1317, 1294, 1232, 1166, 1142, 1100, 1083, 1024, 942, 867, 841, 819, 760, 742, 703, 657, 629.

(*E*)-4-((4-fluorophenyl)sulfonyl)-*N*-phenylbut-3-enamide ((*E*)-3af)

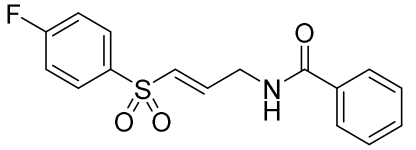

General procedure B was followed to obtain **(*E*)-3af** (18 mg, 0.06 mmol, 11%) as a colourless oil.

^{1}H NMR (400 MHz, DMSO-d_6) δ 8.80 (t, J = 5.6 Hz, 1H), 7.98 - 7.91 (m, 2H), 7.88 - 7.84 (m, 2H), 7.58 - 7.43 (m, 5H), 6.98 (dt, J = 15.2, 4.4 Hz, 1H), 6.82 (dt, J = 15.1, 1.8 Hz, 1H), 4.17 - 4.12 (m, 2H); **^{13}C NMR** (101 MHz, $CDCl_3$) δ 167.6, 165.8 (d, $J_{C\text{-}F}$ = 256.5 Hz), 143.60, 136.0 (d, $J_{C\text{-}F}$ = 3.2 Hz), 133.5, 132.0, 130.8, 130.6 (d, $J_{C\text{-}F}$ = 9.6 Hz), 128.7, 127.2, 116.8 (d, $J_{C\text{-}F}$ = 22.7 Hz), 40.0; **^{19}F NMR** (377 MHz, DMSO-d_6) δ -104.9 - -105.0 (m, 1F); **HRMS** (ESI-TOF)

calculated for $C_{16}H_{15}FO_3N^{32}S$ $[M+H]^+$: 320.0751; found 320.0752; **IR** (neat) 2981, 2680, 1640, 1591, 1493, 1384, 1313, 1290, 1233, 1192, 1141, 1084, 1026, 956, 934, 865, 837, 818, 737, 716, 691, 671, 617.

(*Z*)-4-((4-fluorophenyl)sulfonyl)-*N*-phenylbut-3-enamide ((*Z*)-3af)

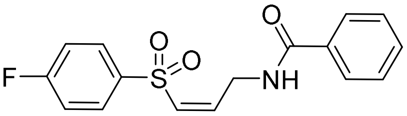

General procedure B was followed to obtain **(*Z*)-3af** (106 mg, 0.33 mmol, 66%) as a white solid. **^{1}H NMR** (400 MHz, DMSO-d_6) δ 8.86 (t, J = 5.5 Hz, 1H), 8.10 - 8.03 (m, 2H), 7.87 - 7.83 (m, 2H), 7.56 - 7.43 (m, 5H), 6.64 (dt, J = 11.2, 2.3 Hz, 1H), 6.43 (dt, J = 11.4, 5.8 Hz, 1H), 4.51 (td, J = 5.7, 2.2 Hz, 2H); **^{13}C NMR** (101 MHz, DMSO-d_6) δ 166.5, 165.0 (d, $J_{C\text{-}F}$ = 253.1 Hz), 145.4, 137.1 (d, $J_{C\text{-}F}$ = 2.9 Hz), 133.9, 131.3, 130.2 (d, $J_{C\text{-}F}$ = 9.9 Hz), 129.4, 128.2, 127.1, 116.8 (d, $J_{C\text{-}F}$ = 22.9 Hz), 36.8; **^{19}F NMR** (377 MHz, DMSO-d$_6$) δ -104.5 - -104.7 (m, 1F); **HRMS** (ESI-TOF) calculated for $C_{16}H_{15}FO_3N^{32}S$ $[M+H]^+$: 320.0751; found 320.0777; **m.p.**: 99 - 103 °C; **IR** (neat) 2981, 1633, 1589, 1578, 1535, 1492, 1428, 1374, 1330, 1307, 1292, 1264, 1221, 1181, 1158, 1141, 1085, 1065, 1031, 1011, 983, 954, 839, 814, 756, 737, 717, 703, 691, 675, 652, 631, 616.

(*E*)-1-fluoro-4-((3-phenoxyprop-1-en-1-yl)sulfonyl)benzene ((*E*)-3ag)

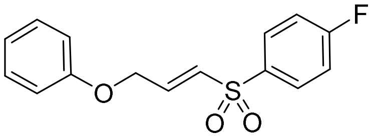

General procedure B was followed to obtain **(*Z*)-3ag** (43 mg, 0.15 mmol, 30%) as a white solid. **^{1}H NMR** (500 MHz, $CDCl_3$) δ 8.00 - 7.95 (m, 2H), 7.36 - 7.30 (m, 2H), 7.30 - 7.24 (m, 2H), 7.18 (dt, J = 15.0, 3.3 Hz, 1H), 7.06 - 7.01 (m, 1H), 6.94 - 6.90 (m, 2H), 6.81 (dt, J = 15.0, 2.2 Hz, 1H), 4.79 (dd, J = 3.4, 2.2 Hz, 2H); **^{13}C NMR** (126 MHz, $CDCl_3$) δ 165.8 (d, $J_{C\text{-}F}$ = 256.3 Hz), 157.6, 141.0, 136.3 (d, $J_{C\text{-}F}$ = 3.2 Hz), 131.2, 130.8 (d, $J_{C\text{-}F}$ = 9.6 Hz), 129.8, 121.9, 116.8 (d, $J_{C\text{-}F}$ = 22.6 Hz), 114.7, 65.6; **^{19}F NMR** (471 MHz, $CDCl_3$) δ -103.6 - - 103.7 (m, 1F); **HRMS** (ESI-TOF)

calculated for $C_{15}H_{12}FO_3{}^{32}S$ [M-H]$^-$: 291.0497; found 291.0490; **m.p.**: 105 - 107 °C; **IR** (neat) 2981, 2889, 1767, 1702, 1638, 1587, 1491, 1446, 1385, 1315, 1294, 1286, 1254, 1236, 1159, 1141, 1087, 1025, 998, 958, 890, 872, 845, 834, 817, 777, 751, 711, 691, 668.

(*Z*)-1-fluoro-4-((3-phenoxyprop-1-en-1-yl)sulfonyl)benzene ((*Z*)-3ag)

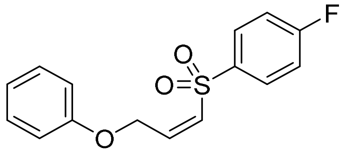

General procedure B was followed to obtain **(*Z*)-3ag** (74 mg, 0.25 mmol, 50%) as a white solid. **^{1}H NMR** (500 MHz, $CDCl_3$) δ 8.01 - 7.94 (m, 2H), 7.35 - 7.25 (m, 4H), 7.04 - 6.99 (m, 1H), 6.95 - 6.90 (m, 2H), 6.56 (dt, J = 11.5, 4.9 Hz, 1H), 6.36 (dt, J = 11.5, 2.4 Hz, 1H), 5.26 (dd, J = 5.0, 2.4 Hz, 2H); **^{13}C NMR** (126 MHz, $CDCl_3$) δ 166.0 (d, $J_{C\text{-}F}$ = 256.9 Hz), 157.8, 143.2, 136.7 (d, $J_{C\text{-}F}$ = 3.2 Hz), 130.5 (d, $J_{C\text{-}F}$ = 9.6 Hz), 130.1, 129.8, 121.6, 116.9 (d, $J_{C\text{-}F}$ = 22.6 Hz), 114.8, 64.0; **^{19}F NMR** (377 MHz, $CDCl_3$) δ -102.9 - -102.9 (m, 1F); **HRMS** (ESI-TOF) calculated for $C_{15}H_{12}FO_3{}^{32}S$ [M-H]$^-$: 291.0497; found 291.0492; **m.p.**: 82 - 83 °C; **IR** (neat) 2981, 2888, 1588, 1494, 1446, 1383, 1317, 1294, 1236, 1206, 1178, 1162, 1149, 1138, 1083, 1044, 1012, 956, 840, 817, 770, 755, 713, 702, 690, 656, 632.

(*Z*)-2-(3-((4-fluorophenyl)sulfonyl)allyl)isoindoline-1,3-dione ((*Z*)-3ah)

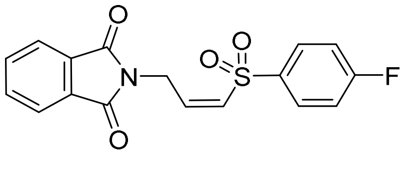

General procedure B was followed to obtain **(*Z*)-3ah** (91 mg, 0.26 mmol, 53%) as a white solid. **^{1}H NMR** (500 MHz, $CDCl_3$) δ 8.12 - 8.06 (m, 2H), 7.85 (dd, J = 5.4, 3.1 Hz, 2H), 7.74 (dd, J = 5.5, 3.0 Hz, 2H), 7.27 (t, J = 8.6 Hz, 2H), 6.32 (dt, J = 11.2, 2.0 Hz, 1H), 6.19 (dt, J = 11.5, 6.0

Hz, 1H), 5.07 (dd, J = 6.0, 2.1 Hz, 2H); **^{13}C NMR** (126 MHz, $CDCl_3$) δ 167.7, 166.0 (d, J_{C-F} = 256.5 Hz), 140.4, 136.5 (d, J_{C-F} = 3.2 Hz), 134.4, 131.9, 131.3, 130.8 (d, J_{C-F} = 9.7 Hz), 123.6, 116.8 (d, J_{C-F} = 22.6 Hz), 35.0; **^{19}F NMR** (471 MHz, $CDCl_3$) δ -103.1 - -103.2 (m, 1F); **HRMS** (ESI-TOF) calculated for $C_{17}H_{13}FNO_4{}^{32}S$ $[M+H]^+$: 346.0544; found 346.0551; **m.p.**: 128 - 130 °C; **IR** (neat) 3658, 2981, 2888, 1766, 1702, 1591, 1495, 1471, 1417, 1395, 1372, 1316, 1294, 1252, 1221, 1144, 1113, 1084, 952, 842, 832, 815, 796, 762, 738, 718, 692, 654.

(*E*)-2-(3-((4-fluorophenyl)sulfonyl)allyl)isoindoline-1,3-dione ((*E*)-3ah)

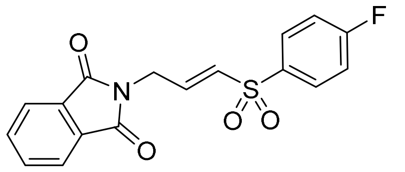

General procedure B was followed to obtain **(*Z*)-3ah** (35 mg, 0.10 mmol, 20%) as a white solid. **^{1}H NMR** (500 MHz, $CDCl_3$) δ 7.89 - 7.83 (m, 4H), 7.76 (dd, J = 5.5, 3.1 Hz, 2H), 7.20 (t, J = 8.5 Hz, 2H), 6.96 (dt, J = 15.1, 5.3 Hz, 1H), 6.43 (dt, J = 15.1, 1.7 Hz, 1H), 4.47 (dd, J = 5.3, 1.7 Hz, 2H); **^{13}C NMR** (126 MHz, $CDCl_3$) δ 167.4, 165.9 (d, J_{C-F} = 256.4 Hz), 139.6, 136.0 (d, J_{C-F} = 3.2 Hz), 134.6, 132.8, 131.9, 130.8 (d, J_{C-F} = 9.6 Hz), 123.8, 116.9 (d, J_{C-F} = 22.8 Hz), 37.6; **^{19}F NMR** (471 MHz, $CDCl_3$) δ -103.4 - - 103.5 (m, 1F); **HRMS** (ESI-TOF) calculated for $C_{17}H_{13}FNO_4{}^{32}S$ $[M+H]^+$: 346.0544; found 346.0544; **m.p.**: 162 - 166 °C; **IR** (neat) 3658, 2981, 2888, 1767, 1703, 1590, 1494, 1471, 1393, 1318, 1292, 1252, 1145, 1114, 1084, 952, 832, 815, 762, 738, 718, 691, 655.

(8*R*,9*S*,13*S*,14*S*)-13-methyl-17-oxo-7,8,9,11,12,13,14,15,16,17-decahydro-6*H*-cyclopenta[*a*]phenanthren-3-yl 3-((4-fluorophenyl)sulfonyl)propanoate (3ai)

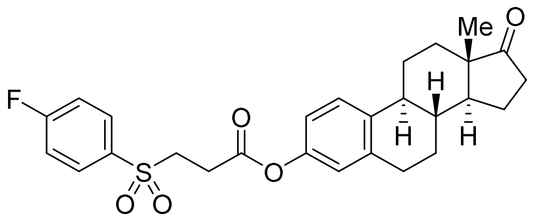

General procedure A was followed to obtain **3ai** (189 mg, 0.39 mmol, 78%) as a white solid. **^{1}H NMR** (500 MHz, $CDCl_3$) δ 7.97 - 7.92 (m, 2H), 7.25 - 7.22 (m, 3H), 6.77 (dd, J = 8.5, 2.6 Hz, 1H), 6.71 (d, J = 2.5 Hz, 1H), 3.50 (t, J = 7.5 Hz, 2H), 2.97 (t, J = 7.5 Hz, 2H), 2.88 - 2.84 (m, 2H), 2.51 - 2.44 (m, 1H), 2.39 - 2.32 (m, 1H), 2.28 - 2.19 (m, 1H), 2.17 - 2.06 (m, 1H), 2.06 - 1.90 (m, 3H), 1.65 - 1.35 (m, 6H), 0.87 (s, 3H); **^{13}C NMR** (126 MHz, $CDCl_3$) δ 220.8, 169.0, 166.2 (d, $J_{C\text{-}F}$ = 257.3 Hz), 148.3, 138.4, 138.0, 134.7 (d, $J_{C\text{-}F}$ = 3.1 Hz), 131.3 (d, $J_{C\text{-}F}$ = 9.6 Hz), 126.7, 121.4, 118.5, 117.0 (d, $J_{C\text{-}F}$ = 22.8 Hz), 51.8, 50.6, 48.1, 44.3, 38.1, 36.0, 31.7, 29.5, 28.1, 26.4, 25.9, 21.7, 14.0; **^{19}F NMR** (471 MHz, $CDCl_3$) δ -102.5 - -102.6 (m, 1F); **HRMS** (ESI-TOF) calculated for $C_{27}H_{33}FO_5N^{32}S$ $[M+NH_4]^+$: 502.2058; found 502.2065; **IR** (neat) 2971, 1692, 1589, 1520, 1489, 1467, 1440, 1380, 1342, 1315, 1295, 1234, 1159, 1130, 1085, 1051, 951, 838, 816, 789, 757, 707, 690, 669, 617.

(*R*)-1-(3-(4-amino-3-(4-phenoxyphenyl)-1*H*-pyrazolo[3,4-*d*]pyrimidin-1-yl)piperidin-1-yl)-3-((4-fluorophenyl)sulfonyl)propan-1-one (3aj)

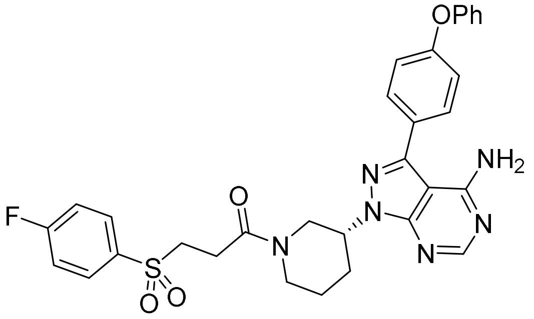

General procedure A was followed to obtain **3aj** (219 mg, 0.37 mmol, 73%) as a white solid. NMR analysis showed two rotamers.

^{1}H NMR (500 MHz, $CDCl_3$) δ 8.38 (s, 0.5H), 8.33 (s, 0.5H), 7.97 - 7.88 (m, 2H), 7.67 - 7.60 (m, 2H), 7.42 - 7.36 (m, 2H), 7.29 - 7.19 (m, 2H), 7.18 - 7.12 (m, 3H), 7.11 - 7.06 (m, 2H), 5.91 - 5.55 (m, 2H), 4.88 - 4.76 (m, 1H), 4.73 - 4.63 (m, 0.5H), 4.40 - 4.33 (m, 0.5H), 4.03 - 3.97 (m, 0.5H), 3.89 - 3.82 (m, 0.5H), 3.74 (br dd, *J* = 13.3, 10.1 Hz, 0.5H), 3.54 - 3.40 (m, 2H), 3.32 (dd, *J* = 12.8, 10.7 Hz, 0.5H), 3.23 - 3.14 (m, 0.5H), 2.96 - 2.84 (m, 2H), 2.83 - 2.75 (m, 0.5H), 2.44 - 2.27 (m, 1H), 2.27 - 2.19 (m, 1H), 2.06 - 1.99 (m, 0.5H), 1.97 - 1.90 (m, 0.5H), 1.77- 1.68 (m, 0.5H), 1.68 - 1.57 (m, 0.5H); **^{13}C NMR** (126 MHz, $CDCl_3$) δ 167.4, 167.3, 166.0 (d, $J_{C\text{-}F}$ = 255.0 Hz), 165.7 (d, $J_{C\text{-}F}$ = 255.7 Hz), 158.7, 158.6, 158.2, 158.0, 156.4, 156.4, 156.1, 155.9, 154.5, 154.3, 144.2, 144.0, 135.4 (d, $J_{C\text{-}F}$= 3.2 Hz), 135.3 (d, *J* = 3.2 Hz), 131.0 (dd, $J_{C\text{-}F}$ = 9.0 Hz), 131.0 (d, $J_{C\text{-}F}$ = 9.0 Hz), 130.1, 130.1, 130.1, 130.1, 127.9, 127.7, 124.2, 124.2, 119.7, 119.7, 119.3, 119.2, 116.9 (d, $J_{C\text{-}F}$ = 22.9 Hz), 116.8 (d, $J_{C\text{-}F}$ = 22.8 Hz), 98.8, 98.6, 53.2, 52.4, 52.4, 52.3, 49.6, 46.2, 45.6, 42.3, 30.2, 30.0, 26.3, 26.2, 25.0, 23.8; **^{19}F NMR** (471 MHz, $CDCl_3$) δ -103.0 - 103.0 (m, 0.5F), -103.0 -103.0 (m, 0.5F); **HRMS** (ESI-TOF) calculated for $C_{31}H_{30}FO_4N_6{}^{32}S$ $[M+H]^+$: 601.2028; found 601.2028; **IR** (neat) 2981, 2361, 2341, 1697, 1632, 1589, 1522, 1491, 1444, 1395, 1356, 1322, 1292, 1237, 1148, 1085, 1010, 954, 837, 802, 788, 755, 693, 669, 655.

2-(2,3-dichloro-4-(2-(((4-fluorophenyl)sulfonyl)methyl)butanoyl)phenoxy)acetic acid (3ak)

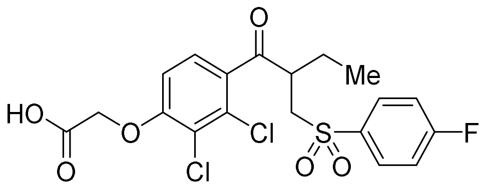

General procedure A was followed to obtain **3aj** (97 mg, 0.21 mmol, 42%) as a white solid. **^{1}H NMR** (500 MHz, $CDCl_3$) δ 7.93 - 7.87 (m, 2H), 7.60 (d, J = 8.6 Hz, 1H), 7.22 (t, J = 8.5 Hz, 2H), 6.84 (d, J = 8.5 Hz, 1H), 4.76 (s, 2H), 3.99 - 3.84 (m, 2H), 3.20 - 3.13 (m, 1H), 1.87 - 1.76 (m, 1H), 1.63 - 1.52 (m, 1H), 0.86 (t, J = 7.4 Hz, 3H); **^{13}C NMR** (101 MHz, $CDCl_3$) δ 199.9, 171.7, 166.1 (d, $J_{C\text{-}F}$ = 257.0 Hz), 156.5, 135.7 (d, $J_{C\text{-}F}$ = 3.2 Hz), 132.7, 132.5, 130.9 (d, $J_{C\text{-}F}$ = 9.6 Hz), 128.5, 124.3, 116.9 (d, $J_{C\text{-}F}$ = 22.7 Hz), 110.8, 65.9, 56.1, 45.5, 24.9, 10.7; **^{19}F NMR** (471 MHz, $CDCl_3$) δ -102.8 - -102.9 (m, 1F); **HRMS** (ESI-TOF) calculated for $C_{19}H_{16}{}^{35}Cl_2FO_6{}^{32}S$ [M-H]$^-$: 461.0034; found 461.0024; **IR** (neat) 2980, 2888, 2360, 2342, 2160, 2034, 1693, 1633, 1589, 1526, 1492, 1463, 1441, 1381, 1316, 1293, 1236, 1149, 1085, 1011, 951, 837, 817, 788, 756, 692, 669, 655, 631, 617.

(6*S*, 8*R*, 9*S*, 10*R*, 13*S*, 14*S*)-6-(((4-fluorophenyl)sulfonyl)methyl)-10,13-dimethyl-7,8,9,10,11,12,13,14,15,16-decahydro-3*H*-cyclopenta[*a*]phenanthrene-3,17(6*H*)-dione (3al)

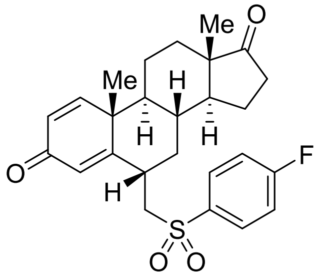

General procedure A was followed to obtain **3al** (114 mg, 0.25 mmol, 50%) as a white solid. **^{1}H NMR** (400 MHz, $CDCl_3$) δ 7.99 - 7.92 (m, 2H), 7.32 - 7.24 (m, 2H), 7.05 (d, J = 10.2 Hz, 1H), 6.22 (dd, J = 10.2, 1.7 Hz, 1H), 5.81 (t, J = 1.6 Hz, 1H), 3.44 (dd, J = 13.5, 3.4 Hz, 1H), 3.36 - 3.26 (m, 1H), 3.13 (dd, J = 13.5, 8.9 Hz, 1H), 2.64 (dt, J = 12.6, 4.1 Hz, 1H), 2.48 (dd, J = 19.5, 8.6 Hz, 1H), 2.08 (dt, J = 19.2, 9.0 Hz, 1H), 2.01 - 1.91 (m, 2H), 1.90 - 1.80 (m, 2H), 1.77 - 1.58 (m, 2H), 1.31 (s, 3H), 1.30 - 1.19 (m, 2H), 1.12 - 0.98 (m, 2H), 0.95 (s, 3H); **^{13}C NMR** (126 MHz, $CDCl_3$) δ 219.6, 185.3, 167.2, 166.3 (d, $J_{C\text{-}F}$ = 256.7 Hz), 156.1, 136.4 (d, $J_{C\text{-}F}$ = 3.6 Hz), 130.8 (d, $J_{C\text{-}F}$ = 9.6 Hz), 127.3, 121.6, 117.1 (d, $J_{C\text{-}F}$ = 22.8 Hz), 58.4, 53.6, 50.3, 47.8, 44.2, 39.2, 35.7, 35.1, 34.2, 31.2, 22.4, 22.0, 18.9, 14.0; **^{19}F NMR** (471 MHz, $CDCl_3$) δ -102.5 - -102.6 (m, 1F); **HRMS** (ESI-TOF) calculated for $C_{26}H_{30}FO_4{}^{32}S$ $[M+H]^+$: 457.1843; found 457.1846; **IR** (neat) 2981, 2889, 2361, 2341, 1735, 1660, 1621, 1590, 1494, 1462, 1381, 1312, 1291, 1239, 1146, 1086, 1010, 953, 888, 818, 717, 669.

***cis*-2-((4-fluorophenyl)sulfonyl)cyclobutane-1-carboxylic acid (3am)**

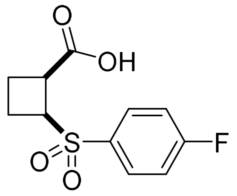

(See section 3; Scale-up experiments)

^{1}H NMR (400 MHz, DMSO-*d*$_6$) δ 12.43 (s, 1H), 7.96 - 7.89 (m, 2H), 7.50 - 7.42 (m, 2H), 4.50 - 4.40 (m, 1H), 3.48 - 3.40 (m, 1H), 2.39 - 2.26 (m, 2H), 2.20 - 2.09 (m, 1H), 2.02 - 1.91 (m, 1H); **^{13}C NMR** (101 MHz, DMSO-*d*$_6$) δ 171.9, 165.5 (d, $J_{C\text{-}F}$ = 252.5 Hz), 135.8 (d, $J_{C\text{-}F}$ = 3.0 Hz), 131.6 (d, $J_{C\text{-}F}$ = 9.9 Hz), 116.8 (d, $J_{C\text{-}F}$ = 22.7 Hz), 58.6, 40.2, 21.3, 20.9; **^{19}F NMR** (471 MHz, DMSO-*d*$_6$) δ -105.1 - -105.2 (m, 1F); **HRMS** (ESI-TOF) calculated for $C_{11}H_{10}FO_4{}^{32}S$ [M-H]$^-$: 257.0289; found 257.0288; **m.p.**: 185 - 187 °C; **IR** (neat) 2981, 2888, 1744, 1587, 1493, 1440, 1382, 1312, 1285, 1269, 1237, 1204, 1165, 1132, 1084, 1013, 955, 847, 836, 818, 779, 759, 718, 689, 650.

***cis*-2-((4-fluorophenyl)sulfonyl)-1'-methylspiro[cyclobutane-1,3'-indolin]-2'-one (*cis*-5a)**

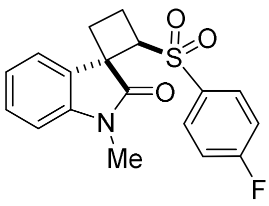

General procedure A was followed to obtain ***cis*-5a** (64 mg, 0.19 mmol, 37%) as a white solid. **^{1}H NMR** (400 MHz, $CDCl_3$) δ 7.69 - 7.64 (m, 2H), 7.24 - 7.19 (m, 2H), 7.05 - 6.99 (m, 3H), 6.64 (d, J = 7.8 Hz, 1H), 4.27 - 4.20 (m, 1H), 3.23 - 3.14 (m, 1H), 3.13 (s, 3H), 2.50 - 2.28 (m, 3H); **^{13}C NMR** (101 MHz, $CDCl_3$) δ 175.2, 165.8 (d, $J_{C\text{-}F}$ = 256.3 Hz), 143.21, 134.7 (d, $J_{C\text{-}F}$ = 3.2 Hz), 131.1 (d, $J_{C\text{-}F}$ = 9.7 Hz), 129.5, 129.3, 122.7, 122.3, 115.9 (d, $J_{C\text{-}F}$ = 22.6 Hz), 108.0, 65.3, 51.9, 28.4, 26.4, 21.0; **^{19}F NMR** (471 MHz, $CDCl_3$) δ -103.6 - -103.7 (m, 1F); **HRMS** (ESI-TOF) calculated for $C_{18}H_{17}FO_3N^{32}S$ $[M+H]^+$: 346.0908; found 346.0915; **m.p.**: 144 - 146 °C; **IR** (neat) 2981, 2889, 1737, 1702, 1615, 1590, 1493, 1474, 1429, 1380, 1350, 1331, 1316, 1286, 1258, 1230, 1192, 1140, 1099, 1084, 1014, 954, 884, 849, 820, 775, 751, 713, 698, 669.

***trans*-2-((4-fluorophenyl)sulfonyl)-1'-methylspiro[cyclobutane-1,3'-indolin]-2'-one (*trans*-5a)**

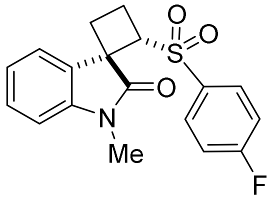

General procedure A was followed to obtain ***trans*-5a** (64 mg, 0.19 mmol, 37%) as a white solid. **^{1}H NMR** (400 MHz, $CDCl_3$) δ 7.83 - 7.79 (m, 1H), 7.34 - 7.28 (m, 3H), 7.18 (td, J = 7.6, 1.1 Hz, 1H), 6.99 - 6.92 (m, 2H), 6.62 (d, J = 7.7 Hz, 1H), 4.48 - 4.41 (m, 1H), 3.16 - 3.03 (m, 1H), 2.89 (s, 3H), 2.72 - 2.60 (m, 1H), 2.58 - 2.48 (m, 1H), 2.31 - 2.24 (m, 1H); **^{13}C NMR** (101 MHz, $CDCl_3$) δ 176.1, 165.8 (d, $J_{C\text{-}F}$ = 256.4 Hz), 143.5, 134.0 (d, $J_{C\text{-}F}$ = 3.2 Hz), 131.0 (d, $J_{C\text{-}F}$ = 9.6 Hz), 129.1, 127.0, 126.5, 122.8, 116.0 (d, $J_{C\text{-}F}$ = 22.6 Hz), 107.9, 61.9, 50.4, 28.6, 26.3, 20.0; **^{19}F NMR** (471 MHz, $CDCl_3$) δ -103.73 - -103.81 (m, 1F); **HRMS** (ESI-TOF) calculated for $C_{18}H_{17}FO_3N^{32}S$

[M+H]$^+$: 346.0908; found 346.0908; **m.p.**: 164 - 166 °C; **IR** (neat) 1713, 1614, 1588, 1493, 1468, 1427, 1406, 1376, 1348, 1310, 1292, 1271, 1240, 1128, 1188, 1143, 1105, 1084, 1053, 1024, 1009, 970, 937, 846, 816, 807, 793, 742, 711, 698, 655, 626.

***cis*-2-(isopropylsulfonyl)-1'-methylspiro[cyclobutane-1,3'-indolin]-2'-one (*cis*-5b)**

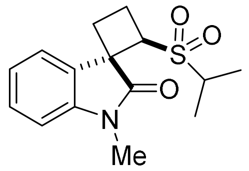

General procedure A was followed to obtain ***cis*-5b** (32 mg, 0.11 mmol, 22%) as a white solid. **^{1}H NMR** (400 MHz, $CDCl_3$) δ 7.34 - 7.27 (m, 2H), 7.08 (td, J = 7.5, 1.0 Hz, 1H), 6.80 (d, J = 7.7 Hz, 1H), 4.16 - 4.07 (m, 1H), 3.38 - 3.26 (m, 1H), 3.23 (s, 3H), 2.99 (h, J = 6.9 Hz, 1H), 2.54 - 2.44 (m, 1H), 2.42 - 2.30 (m, 2H), 1.22 (d, J = 6.8 Hz, 3H), 1.18 (d, J = 6.9 Hz, 3H); **^{13}C NMR** (101 MHz, $CDCl_3$) δ 176.4, 143.8, 130.0, 129.5, 122.7, 122.3, 108.3, 60.7, 54.1, 51.9, 28.3, 26.6, 21.6, 15.3, 15.1; **HRMS** (ESI-TOF) calculated for $C_{15}H_{20}O_3N^{32}S$ [M+H]$^+$: 294.1158; found 294.1162; **m.p.**: 186 - 188 °C; **IR** (neat) 2980, 2889, 1704, 1611, 1491, 1470, 1418, 1378, 1349, 1307, 1264, 1220, 1140, 1130, 1115, 1099, 1059, 1020, 1009, 972, 938, 898, 879, 855, 811, 789, 754, 692, 666, 609.

***trans*-2-(isopropylsulfonyl)-1'-methylspiro[cyclobutane-1,3'-indolin]-2'-one (*trans*-5b)**

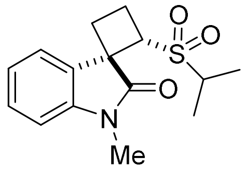

General procedure A was followed to obtain ***trans*-5b** (79 mg, 0.27 mmol, 54%) as a white solid. **^{1}H NMR** (400 MHz, $CDCl_3$) δ 7.83 - 7.79 (m, 1H), 7.31 (td, J = 7.7, 1.2 Hz, 1H), 7.13 (td, J = 7.6, 1.0 Hz, 1H), 6.82 (dt, J = 7.7, 0.8 Hz, 1H), 4.34 (t, J = 8.7 Hz, 1H), 3.21 (s, 4H), 3.08 - 2.95 (m,

1H), 2.73 - 2.61 (m, 2H), 2.58 - 2.48 (m, 1H), 2.39 - 2.30 (m, 1H), 1.20 (d, J = 6.8 Hz, 3H), 1.19 (d, J = 6.9 Hz, 3H); **^{13}C NMR** (101 MHz, $CDCl_3$) δ 177.1, 143.3, 129.1, 127.9, 126.3, 122.9, 108.2, 56.9, 53.4, 50.5, 29.3, 26.7, 21.0, 15.5, 14.4; **HRMS** (ESI-TOF) calculated for $C_{15}H_{20}O_3N^{32}S$ $[M+H]^+$: 294.1158; found 294.1163; **m.p.**: 166 - 168 °C; **IR** (neat) 2981, 2889, 1704, 1611, 1491, 1470, 1418, 1377, 1348, 1292, 1260, 1140, 1127, 1093, 1051, 1018, 955, 939, 898, 880, 855, 805, 789, 755, 690, 666, 611.

***trans*-1-chloro-2-((4-fluorophenyl)sulfonyl)-*N*-phenylcyclobutane-1-carboxamide (6)**

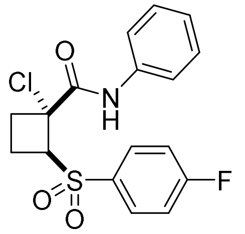

4-fluorobenzenesulfonyl chloride (243 mg, 1.3 mmol, 2.5 equiv) was added to a stirred suspension of *N*-phenylacrylamide (74 mg, 0.5 mmol, 1.0 equiv), *fac*-Ir(ppy)$_3$ (1.70 mg, 0.0025 mmol, 0.5 mol%), MeCN (3.0 mL) and K_2HPO_4 (17.4 mg, 0.1 mmol, 0.2 equiv). The reaction mixture was stirred under blue LED irradiation for 1 hour. The solution was concentrated *in vacuo* and purified by flash column chromaography (silica, EtOAc in Heptane, 0/100 to 100/0) to afford **6** (134 mg, 0.37 mmol, 73%) as a white solid. **^{1}H NMR** (500 MHz, $CDCl_3$) δ 8.32 (s, 1H), 7.85 - 7.79 (m, 2H), 7.59 - 7.55 (m, 2H), 7.39 - 7.34 (m, 2H), 7.19 - 7.14 (m, 3H), 4.31 (t, J = 9.7 Hz, 1H), 3.31 - 3.22 (m, 1H), 2.52 (dt, J = 20.0, 10.3 Hz, 1H), 2.36 (dt, J = 11.5, 9.7 Hz, 1H), 2.25 - 2.16 (m, 1H); **^{13}C NMR** (126 MHz, $CDCl_3$) δ 166.2 (d, $J_{C\text{-}F}$ = 257.7 Hz), 163.4, 137.2, 133.9 (d, $J_{C\text{-}F}$ = 3.2 Hz), 131.4 (d, $J_{C\text{-}F}$ = 9.7 Hz), 129.1, 125.2, 120.6, 116.8 (d, $J_{C\text{-}F}$ = 22.8 Hz), 69.3, 66.1, 33.1, 20.9; **^{19}F NMR** (471 MHz, $CDCl_3$): δ - 102.0 - - 102.1 (m, 1F); **HRMS** (ESI-TOF) calculated for $C_{17}H_{14}FO_3NCl^{32}S$ $[M-H]^-$: 366.0372; found 366.0365; **m.p.**: 103 - 106 °C; **IR** (neat) 2981, 1691, 1588, 1488, 1439, 1400, 1321, 1298, 1257, 1233, 1150, 1095, 1051, 1021, 990, 973, 903, 839, 814, 757, 707, 668. Single Crystal Data for **6**: $C_{17}H_{15}ClFNO_3S$, Mr =367.83. 150 K – orthorhombic, P21/n, a = 6.8699(2) Å, b = 27.6557(7) Å, c = 8.8183(2) Å, V = 1644.22(7) Å, Data/restraints/parameters – 3403/0/217.

4-((4-fluorophenyl)sulfonyl)-*N*-phenylbutanamide (8a)

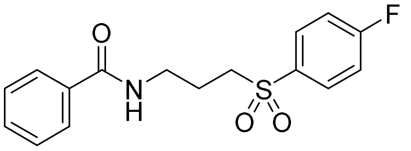

General procedure B was followed to obtain **8a** (64 mg, 0.20 mmol, 40%) as a white solid. **General procedure C** was followed to obtain **8a** (132 mg, 0.41 mmol, 82%) as a white solid. **^{1}H NMR** (400 MHz, $CDCl_3$) δ 7.93 - 7.87 (m, 2H), 7.77 - 7.73 (m, 2H), 7.50 - 7.45 (m, 1H), 7.42 - 7.37 (m, 2H), 7.25 - 7.18 (m, 2H), 6.81 - 6.72 (m, 1H), 3.58 (q, J = 6.4 Hz, 2H), 3.22 - 3.16 (m, 2H), 2.08 (p, J = 6.7 Hz, 2H); **^{13}C NMR** (101 MHz, $CDCl_3$) δ 167.9, 166.1 (d, $J_{C\text{-}F}$ = 256.8 Hz), 135.2 (d, $J_{C\text{-}F}$ = 3.2 Hz), 134.2, 131.8, 131.0 (d, $J_{C\text{-}F}$ = 9.5 Hz), 128.7, 127.1, 116.9 (d, $J_{C\text{-}F}$ = 22.6 Hz), 54.3, 38.4, 23.2; **^{19}F NMR** (377 MHz, $CDCl_3$) δ -103.0 - -103.1 (m, 1F); **HRMS** (ESI-TOF) calculated for $C_{16}H_{17}FO_3N^{32}S$ $[M+H]^+$: 322.0908; found 322.0916; **m.p.**: 136 - 138 °C; **IR** (neat) 1640, 1592, 1579, 1543, 1489, 1459, 1450, 1286, 1234, 1185, 1158, 1141, 1087, 1036, 1012, 999, 977, 858, 835, 818, 801, 771, 731, 717, 694, 672, 658, 619.

12-(5-((4-fluorophenyl)sulfonyl)pentyl)isoindoline-1,3-dione

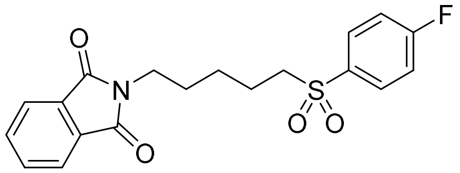

General procedure C was followed to obtain **8b** (143 mg, 0.38 mmol, 76%) as a white solid. **^{1}H NMR** (500 MHz, $CDCl_3$) δ 7.95 - 7.89 (m, 2H), 7.84 - 7.80 (m, 2H), 7.74 - 7.70 (m, 2H), 7.28 - 7.22 (m, 2H), 3.65 (t, J = 7.1 Hz, 2H), 3.13 - 3.07 (m, 2H), 1.81 - 1.72 (m, 2H), 1.71 - 1.63 (m, 2H), 1.48 - 1.39 (m, 2H); **^{13}C NMR** (126 MHz, $CDCl_3$) δ 168.3, 165.7 (d, $J_{C\text{-}F}$ = 256.1 Hz), 135.1 (d, $J_{C\text{-}F}$ = 3.2 Hz), 134.0, 131.9, 130.9 (d, $J_{C\text{-}F}$ = 9.6 Hz), 123.1, 116.6 (d, $J_{C\text{-}F}$ = 22.6 Hz), 56.0, 37.3, 28.0, 25.4, 22.3; **^{19}F NMR** (471 MHz, $CDCl_3$) δ -103.6 - -103.7 (m, 1F); **HRMS** (ESI-TOF) calculated for $C_{19}H_{19}FO_4N^{32}S$ $[M+H]^+$: 376.1013; found 376.1025; **m.p.**: 120 - 122 °C; **IR** (neat): 1771, 1707, 1640, 1592, 1543, 1494, 1466, 1441, 1400, 1378, 1340, 1319, 1286, 1255, 1230, 1199,

1187, 1162, 1145, 1087, 1074, 1049, 1014, 1000, 959, 941, 873, 835, 817, 794, 766, 721, 712, 693, 671, 622.

1-fluoro-4-((3-phenoxypropyl)sulfonyl)benzene (8c)

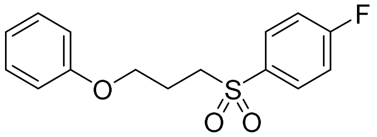

General procedure C was followed to obtain **8c** (116 mg, 0.40 mmol, 79%) as a colourless oil. **^{1}H NMR** (500 MHz, $CDCl_3$) δ 7.97 - 7.91 (m, 2H), 7.29 - 7.19 (m, 4H), 6.94 (t, J = 7.4 Hz, 1H), 6.84 - 6.78 (m, 2H), 4.01 (t, J = 5.9 Hz, 2H), 3.34 - 3.28 (m, 2H), 2.25 - 2.16 (m, 2H); **^{13}C NMR** (126 MHz, $CDCl_3$) δ 165.9 (d, $J_{C\text{-}F}$ = 256.5 Hz), 158.3, 135.2 (d, $J_{C\text{-}F}$ = 3.3 Hz), 131.0 (d, $J_{C\text{-}F}$ = 9.6 Hz), 129.6, 121.3, 116.8 (d, $J_{C\text{-}F}$ = 22.7 Hz), 114.5, 65.3, 53.5, 23.1; **^{19}F NMR** (471 MHz, $CDCl_3$) δ -103.3 - -103.4 (m, 1F); **HRMS** (ESI-TOF) calculated for $C_{15}H_{14}FO_3{}^{32}S$ $[M\text{-}H]^-$: 293.0653 found 293.0659; **IR** (neat): 1589, 1493, 1473, 1405, 1316, 1288, 1237, 1172, 1140, 1085, 1040, 1014, 930, 886, 838, 819, 781, 755, 729, 692, 672, 634.

4-((4-fluorophenyl)sulfonyl)butyl 4-cyanobenzoate (8d)

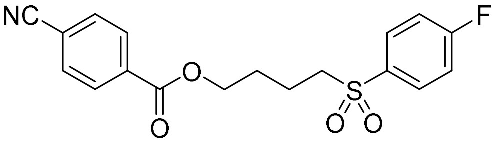

General procedure C was followed to obtain **8d** (147 mg, 0.41 mmol, 82%) as a colourless oil. **^{1}H NMR** (400 MHz, $CDCl_3$) δ 8.10 - 8.05 (m, 2H), 7.95 - 7.89 (m, 2H), 7.76 - 7.72 (m, 2H), 7.26 - 7.19 (m, 2H), 4.38 - 4.29 (m, 2H), 3.21 - 3.08 (m, 2H), 1.96 - 1.82 (m, 4H); **^{13}C NMR** (101 MHz, $CDCl_3$) δ 165.5 (d, $J_{C\text{-}F}$ = 258.7 Hz), 164.8, 135.1 (d, $J_{C\text{-}F}$ = 3.2 Hz), 133.8, 132.3, 131.0 (d, $J_{C\text{-}F}$ = 9.6 Hz), 130.1, 118.0, 116.7 (d, $J_{C\text{-}F}$ = 22.6 Hz), 116.5, 64.5, 55.8, 27.3, 19.6; **^{19}F NMR** (377 MHz, $CDCl_3$) δ -103.0 - -103.1 (m, 1F); **HRMS** (ESI-TOF) calculated for $C_{18}H_{15}FO_4N^{32}S$ $[M+H]^+$: 360.0711; found 360.0696; **IR** (neat) 2232, 1715, 1591, 1492, 1472, 14004, 1313, 1272, 1221, 1178, 1141, 1123, 1111, 1085, 1034, 1020, 961, 905, 868, 838, 778, 765, 738, 694.

4-((4-fluorophenyl)sulfonyl)-3-methylbutyl benzoate (8e)

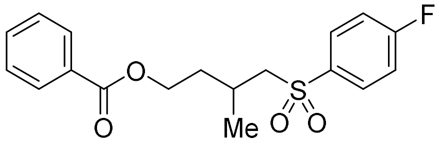

General procedure C was followed to obtain **8e** (151 mg, 0.43 mmol, 86%) as a white solid.
^{1}H NMR (400 MHz, $CDCl_3$) δ 7.99 - 7.94 (m, 2H), 7.92 - 7.86 (m, 2H), 7.56 (ddt, J = 7.9, 6.9, 1.3 Hz, 1H), 7.46 - 7.40 (m, 2H), 7.18 - 7.11 (m, 2H), 4.37 - 4.28 (m, 2H), 3.18 (dd, J = 14.2, 5.3 Hz, 1H), 3.01 (dd, J = 14.2, 7.3 Hz, 1H), 2.35 - 2.23 (m, 2H), 2.06 - 1.96 (m, 2H), 1.79 - 1.67 (m, 2H), 1.18 (d, J = 6.8 Hz, 3H); **^{13}C NMR** (101 MHz, $CDCl_3$) δ 166.8 (d, $J_{C\text{-}F}$ = 263.2 Hz), 164.6, 136.0 (d, $J_{C\text{-}F}$ = 3.3 Hz), 133.2, 130.8 (d, $J_{C\text{-}F}$ = 9.6 Hz), 130.1, 129.6, 128.5, 116.7 (d, $J_{C\text{-}F}$ = 22.6 Hz), 62.4, 62.1, 35.2, 26.3, 19.9; **^{19}F NMR** (471 MHz, $CDCl_3$) δ -103.3 - -103.4 (m, 1F); **HRMS** (ESI-TOF) calculated for $C_{18}H_{20}FO_4{}^{32}S$ $[M+H]^+$: 351.1061; found 351.1065; **m.p.**: 63 - 65 °C; **IR** (neat): 1712, 1588, 1491, 1469, 1455, 1405, 1390, 1359, 1310, 1278, 1243, 1230, 1194, 1178, 1142, 1123, 1097, 1083, 1072, 1032, 1000, 954, 854, 843, 820, 801, 765, 713, 690, 677, 632, 618.

(1s,3s)-3-(((4-fluorophenyl)sulfonyl)methyl)cyclobutane-1-carbonitrile (8f)

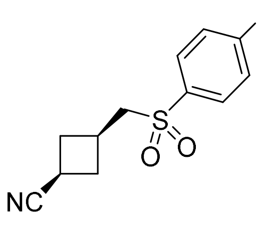

General procedure C was followed to obtain **8f** (118 mg, 0.47 mmol, 93%) as a white solid (mixture of both diastereomers, d.r: 60/40).
^{1}H NMR (400 MHz, $CDCl_3$) δ 7.94 - 7.87 (m, 2H), 7.30 - 7.23 (m, 2H), 3.26 (d, J = 7.3 Hz, 1H), 3.25 (d, J = 7.4 Hz, 1H), 3.15 - 3.07 (m, 0.4H), 3.07 - 2.96 (m, 1H), 2.87 - 2.73 (m, 0.6H), 2.61 - 2.47 (m, 2H), 2.23 - 2.12 (m, 2H); **^{13}C NMR** (101 MHz, $CDCl_3$) δ 166.5 (d, $J_{C\text{-}F}$ = 257.1 Hz), 166.0 (d, $J_{C\text{-}F}$ = 257.1 Hz), 135.3 (d, $J_{C\text{-}F}$ = 3.2 Hz), 135.1 (d, $J_{C\text{-}F}$ = 3.2 Hz), 130.9 (d, $J_{C\text{-}F}$ = 9.5 Hz), 130.9 (d, $J_{C\text{-}F}$ = 9.6 Hz), 122.2, 121.2, 117.0 (d, $J_{C\text{-}F}$ = 22.5 Hz), 116.9 (d, $J_{C\text{-}F}$ = 22.6 Hz), 61.3, 60.7, 32.8, 31.4, 27.7, 27.2, 20.2, 19.3; **^{19}F NMR** (471 MHz, $CDCl_3$) δ -102.6 - -102.8 (m,

1F); **HRMS** (ESI-TOF) calculated for $C_{12}H_{16}FO_2N_2{}^{32}S$ $[M+NH_4]^+$: 271.0911; found 271.0915; **m.p.**: 100 - 102 °C; **IR** (neat): 2947, 2360, 2341, 1587, 1493, 1406, 1310, 1287, 1225, 1142, 1099, 1085, 1012, 844, 818, 780, 754, 671.

3-((4-fluorophenyl)sulfonyl)cyclopentyl benzoate (8g)

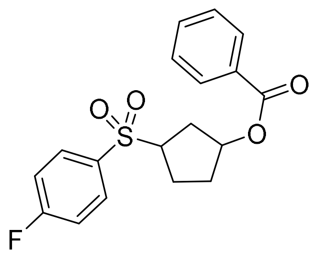

General procedure C was followed to obtain **8g** (137 mg, 0.38 mmol, 75%) as a white solid.
^{1}H NMR (500 MHz, $CDCl_3$) δ 7.99 - 7.93 (m, 4H), 7.60 - 7.55 (m, 1H), 7.47 - 7.41 (m, 2H), 7.30 - 7.25 (m, 2H), 5.57 - 5.52 (m, 1H), 3.83 - 3.74 (m, 1H), 2.44 (ddd, J = 14.5, 8.9, 5.4 Hz, 1H), 2.36 - 2.27 (m, 1H), 2.26 - 2.17 (m, 3H), 2.08 - 1.98 (m, 1H); **^{13}C NMR** (126 MHz, $CDCl_3$) δ 166.0 (d, $J_{C\text{-}F}$ = 256.2 Hz), 165.9, 134.7 (d, $J_{C\text{-}F}$ = 3.4 Hz), 133.3, 131.4 (d, $J_{C\text{-}F}$ = 9.6 Hz), 130.1, 129.6, 128.5, 116.83 (d, $J_{C\text{-}F}$ = 22.6 Hz), 76.6, 62.9, 34.4, 32.0, 25.0; **^{19}F NMR** (471 MHz, $CDCl_3$) δ -103.2 - -103.3 (m, 1F); **HRMS** (ESI-TOF) calculated for $C_{18}H_{21}FO_4N^{32}S$ $[M+NH_4]^+$: 366.1170; found 366.1178; **m.p.**: 120 - 122 °C; **IR** (neat): 1713, 1590, 1493, 1451, 1404, 1359, 1314, 1271, 1233, 1142, 1112, 1084, 1070, 1026, 1014, 967, 911, 839, 711, 685, 671.
Stereochemistry of **8g** could not be assigned.

6-chloro-1-(3-((4-fluorophenyl)sulfonyl)propyl)-3-methylpyrimidine-2,4(1H,3H)-dione (8h)

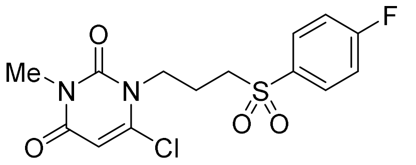

General procedure C was followed to obtain **8h** (131 mg, 0.37 mmol, 73%) as a white solid.
^{1}H NMR (400 MHz, $CDCl_3$) δ 7.99 - 7.92 (m, 2H), 7.31 - 7.24 (m, 2H), 5.94 (s, 1H), 4.21 (t, J = 7.3 Hz, 2H), 3.30 (s, 3H), 3.20 (t, J = 7.6 Hz, 2H), 2.22 - 2.11 (m, 2H); **^{13}C NMR** (101 MHz, $CDCl_3$) δ 166.1 (d, $J_{C\text{-}F}$ = 257.0 Hz), 160.6, 151.1, 145.0, 134.8 (d, $J_{C\text{-}F}$ = 3.2 Hz), 131.1 (d, $J_{C\text{-}F}$ = 9.7 Hz), 116.93 (d, $J_{C\text{-}F}$ = 22.6 Hz), 102.5, 53.7, 45.4, 28.4, 22.4;**^{19}F NMR** (471 MHz, $CDCl_3$) δ -102.73 (tt, J = 8.7, 4.5 Hz, 1F); **HRMS** (ESI-TOF) calculated for $C_{14}H_{15}ClFO_4N_2{}^{32}S$ $[M+H]^+$: 361.0420; found 361.0428.; **m.p.**: 136 - 138 °C; **IR** (neat): 1701, 1665, 1605, 1591, 1494, 1438, 1418, 1401, 1376, 1351, 1314, 1284, 1237, 1204, 1158, 1140, 1085, 1030, 962, 861, 819, 774, 756, 690, 667, 652, 634.

1-(3-((4-fluorophenyl)sulfonyl)propyl)-3,7-dimethyl-3,7-dihydro-1H-purine-2,6-dione (8i)

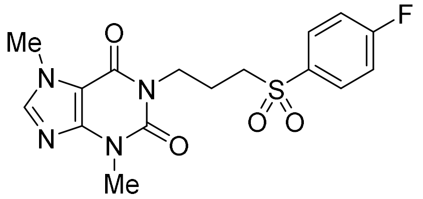

General procedure C was followed to obtain **8i** (74 mg, 0.21 mmol, 41%) as a white solid.
^{1}H NMR (400 MHz, $CDCl_3$) δ 7.93 - 7.88 (m, 2H), 7.51 - 7.50 (m, 1H), 7.24 - 7.18 (m, 2H), 4.07 (t, J = 6.7 Hz, 2H), 3.95 (s, 3H), 3.53 (s, 3H), 3.21 - 316 (m, 2H), 2.11 - 2.02 (m, 2H); **^{13}C NMR** (101 MHz, $CDCl_3$) δ 166.0 (d, $J_{C\text{-}F}$ = 256.3 Hz), 155.2, 152.0, 149.1, 141.9, 135.0 (d, $J_{C\text{-}F}$ = 3.2 Hz), 131.2 (d, $J_{C\text{-}F}$ = 9.6 Hz), 116.7 (d, $J_{C\text{-}F}$ = 22.6 Hz), 107.6, 54.5, 39.6, 33.7, 29.9, 22.1; **^{19}F NMR** (471 MHz, $CDCl_3$) δ -103.5 (s, 1F); **HRMS** (ESI-TOF) calculated for $C_{16}H_{18}FO_4N_4{}^{32}S$ $[M+H]^+$: 381.1027; found 381.1030; **m.p.**: 159 - 161 °C; **IR** (neat): 1710, 1656, 1587, 1548, 1489, 1445, 1406, 1376, 1354, 1310, 1281, 1229, 1197, 1182, 1142, 1112, 1097, 1085, 1028, 1010, 1001, 898, 879, 838, 822, 804, 784, 748, 731, 635, 611.

3-(3,5-dichlorophenyl)-5-(2-((4-fluorophenyl)sulfonyl)ethyl)-5-methyloxazolidine-2,4-dione (8j)

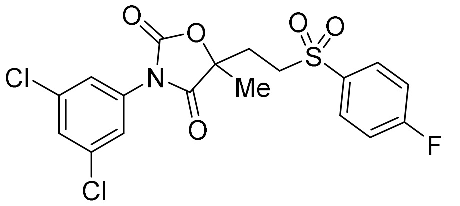

General procedure C was followed to obtain **8i** (148 mg, 0.34 mmol, 67%) as a white solid.
^{1}H NMR (400 MHz, $CDCl_3$) δ 7.97 - 7.91 (m, 2H), 7.45 - 7.42 (m, 3H), 7.32 - 7.27 (m, 2H), 3.27 (ddd, J = 13.7, 10.4, 5.6 Hz, 2H), 3.16 (ddd, J = 13.7, 10.6, 5.9 Hz, 2H), 2.49 - 2.32 (m, 2H), 1.67 (s, 3H); **^{13}C NMR** (101 MHz, DMSO-d_6) δ 172.8, 165.3 (d, $J_{C\text{-}F}$ = 252.9 Hz), 152.0, 134.9 (d, $J_{C\text{-}F}$ = 3.0 Hz), 134.0, 133.1, 131.2 (d, $J_{C\text{-}F}$ = 9.9 Hz), 128.6, 125.8, 116.8 (d, $J_{C\text{-}F}$ = 22.8 Hz), 84.1, 49.3, 28.7, 20.9; **^{19}F NMR** (471 MHz, DMSO-d_6) δ -104.4 - -104.6 (m, 1F); **HRMS** (ESI-TOF) calculated for $C_{18}H_{13}Cl_2FO_5N^{32}S$ $[M+H]^+$: 443.9881; found 443.9873; **IR** (neat) 1816, 1730, 1577, 1492, 1455, 1397, 1379, 1307, 1232, 1179, 1138, 1085, 1053, 1027, 961, 938, 864, 834, 806, 764, 746, 677, 657, 640, 622.

8. Determination of the stereochemistry for compounds 3aa, 3ab, 3ac, 5a, 5b

The determination of the stereochemistry was achieved using NOESY (Nuclear Overhauser Effect SpectroscopY) analysis.

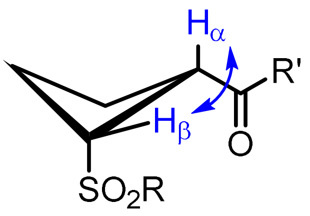

***cis*-configuration:**

NOE interaction detected between H_α and H_β.

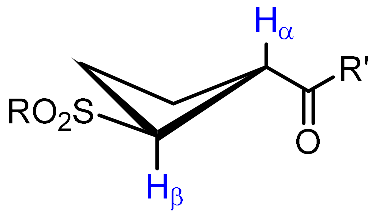

***trans*-configuration:**

No NOE interaction detected between H_α and H_β.

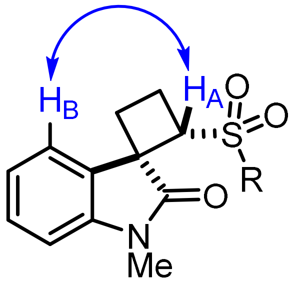

***cis*-configuration:**

NOE interaction detected between H_A and H_B.

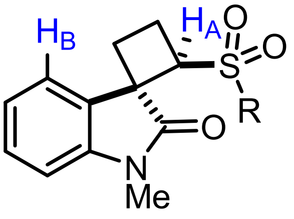

***trans*-configuration:**

No NOE interaction detected between H_A and H_B.

9. References

[1] X.-J. Tang, W. R. Dolbier, Jr., *Angew. Chem. Int. Etd*. **2015**, *54*, 4246–4249; *Angew. Chem.* **2015**, *127*, 4320–4323.

[2] J. Eriksson, O. Åberg, B. Långström, *Eur. J. Org. Chem*. **2007**, *3*, 455–461.

[3] O. A. Al-Fulaij, A.-Z. A. Elassar, A. F. El-asmy, *J. Appl. Polym.* **2015**, *43,* 42712–42726.

[4] D. B. Bagal, S.-W. Park, H.-J. Song, S. Chang, *Chem. Commun.* **2017**, *53*, 8798–8801.

[5] C. J. Teskey, P. Adler, C.R. Goncalves, N. Maulide, *Angew. Chem. Int. Ed.* **2019**, *58*, 447–451; *Angew. Chem.* **2019**, *131*, 456–460.

[6] H.-D. Xu, W. Zhang, D.-X. Shu, J. B. Werness, W.-P. Tang, *Angew. Chem. Int. Ed.* **2008**, *47*, 8933–8936; *Angew. Chem.* **2008**, *120*, 9065–9068.

[7] X.-J. Tang, W. R. Dolbier, Jr., *Angew. Chem. Int. Etd*. **2015**, *54*, 4246–4249; *Angew. Chem.* **2015**, *127*, 4320–4323.

[8] A. Song, K. A. Parker, N. S. Sampson, *J. Am. Chem. Soc*. **2009**, *131,* 3444–3445.

[9] J. E. Enholm, T. Low, D. Cooper, I. Ghivirija, *Tetrahedron* **2012**, *68*, 6920–6927.

[10] J. L. Romine, Z. Yang, G. Wang, V. N. Nguyen, J. A. Bender, D. R. St. Laurent, M. Belema, WO/2014/065791, **2014**.

[11] P. J. Bhuyan, H. N. Borah, J. S. Sandhu, *J. Chem. Soc.*, Perkin Trans. **1999**, *1*, 3083–3084.

[12] H. K. Hun, F. T. Luo, *J. Ch. Chem. Soc.* **2012**, *59*, 394.

[13] Y. Xie, P.-W. Sun, Y. Li, S. Wang, M. Ye and Z. Li, *Angew.Chem.Int.Ed.*, **2019**, *58*, 7097–7101; *Angew. Chem.* **2019**, *131*, 7171 –7175.

[14] Q. Y. Lin, X. H. Xu, K. Zhang, F. L. Qing, *Angew. Chem. Int. Ed.*, **2016**, *55*, 1479–1483; *Angew. Chem.* **2016**, *128*, 1501–1505.

[15] L. Palatinus, G. Chapuis, *J. Appl. Cryst.* **2007**, *40*, 786–790.

[16] a) P. W. Betteridge, J. R. Carruthers, R. I. Cooper, K. Prout, D. J. Watkin, *J. Appl. Cryst.* **2003**, *36*, 1487; b) R. I. Cooper, A. L. Thompson, D. J. Watkin, *J. Appl. Cryst.* **2010**, *43*, 1100-1107.

[17] L. Li, Y. Liu, Y. Peng, L. Yu, X. Wu, H. Yan, *Angew. Chem. Int. Ed.* **2016**, *55*, 331–335; *Angew. Chem.* **2016**, *128*, 339–343.

10. Spectra

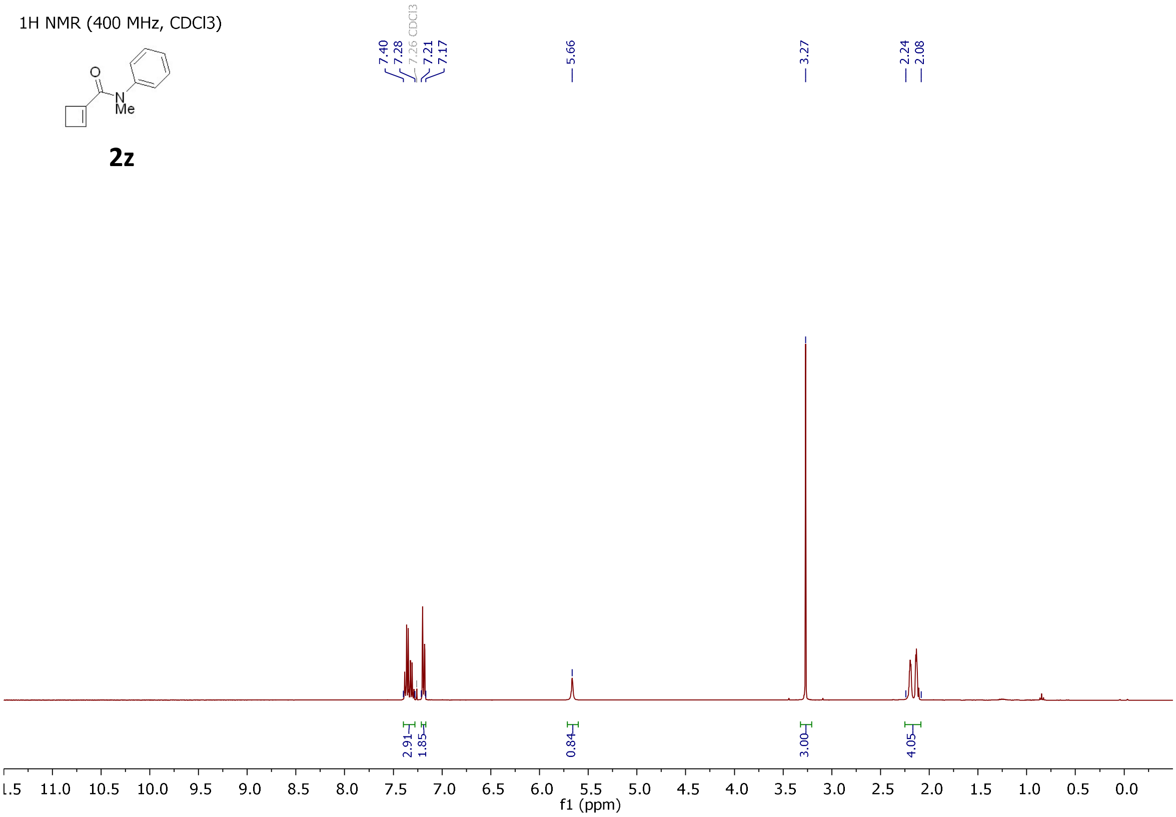
1H NMR (400 MHz, CDCl3)
O
N
Me
2z
7.40
7.28
7.26 CDCl3
7.21
7.17
5.66
3.27
2.24
2.08
2.91
1.85
0.84
3.00
4.05
f1 (ppm)

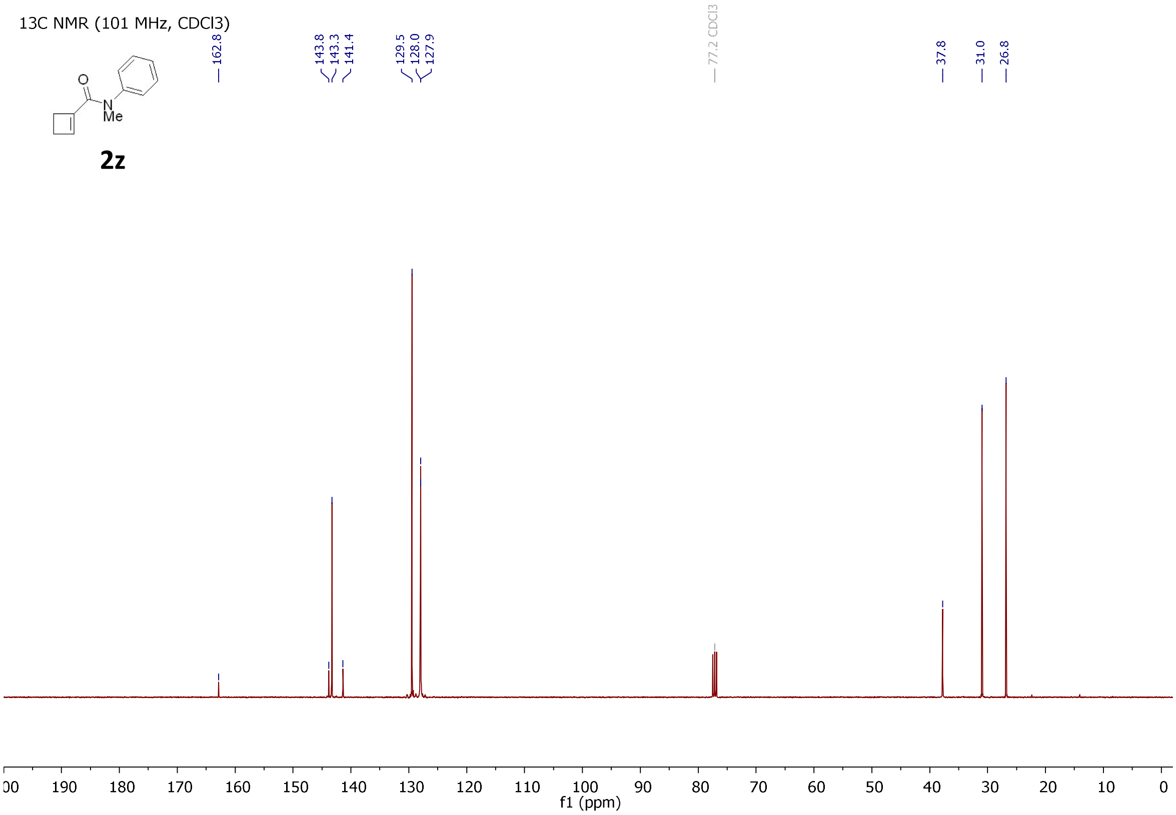
13C NMR (101 MHz, CDCl3)
O
N
Me
2z
162.8
143.8
143.3
141.4
129.5
128.0
127.9
77.2 CDCl3
37.8
31.0
26.8
f1 (ppm)

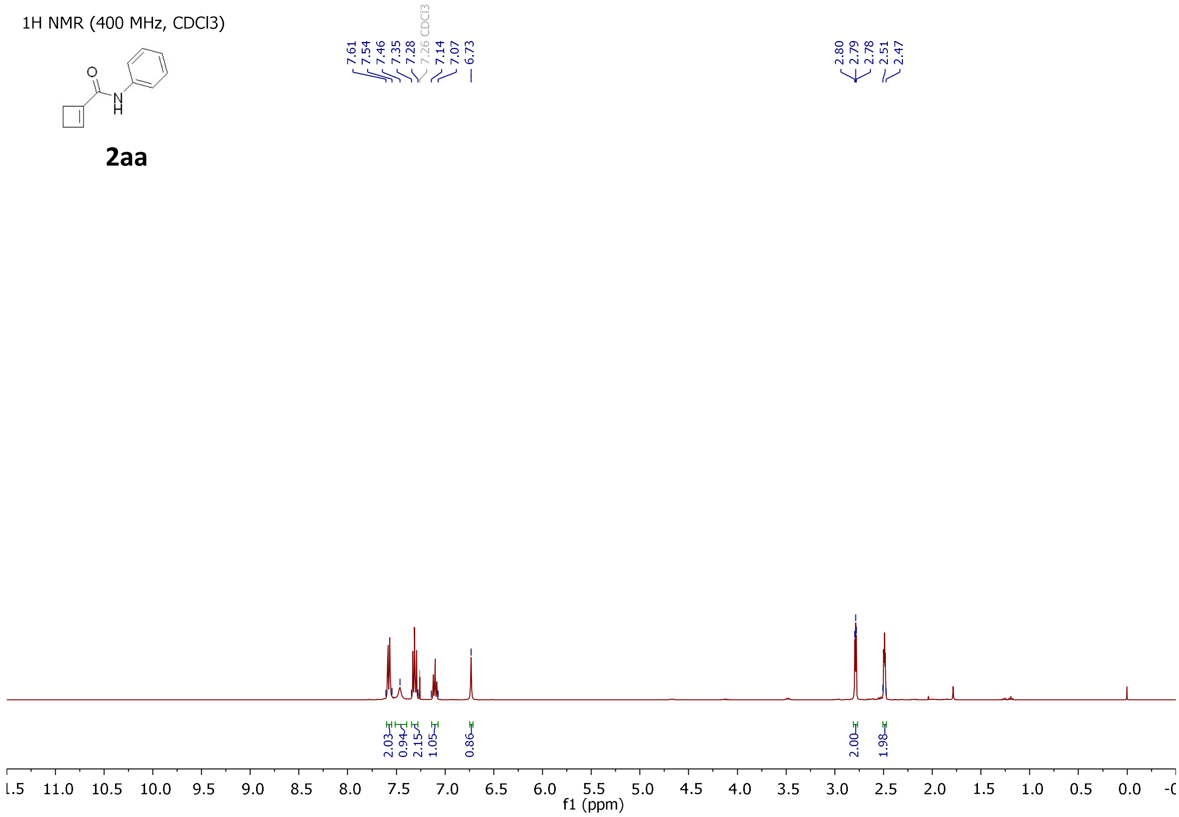
1H NMR (400 MHz, CDCl3)
2aa
7.61
7.54
7.46
7.35
7.28
7.26 CDCl3
7.14
7.07
6.73
2.80
2.79
2.78
2.51
2.47
2.03
0.94
2.15
1.05
0.86
2.00
1.98
f1 (ppm)

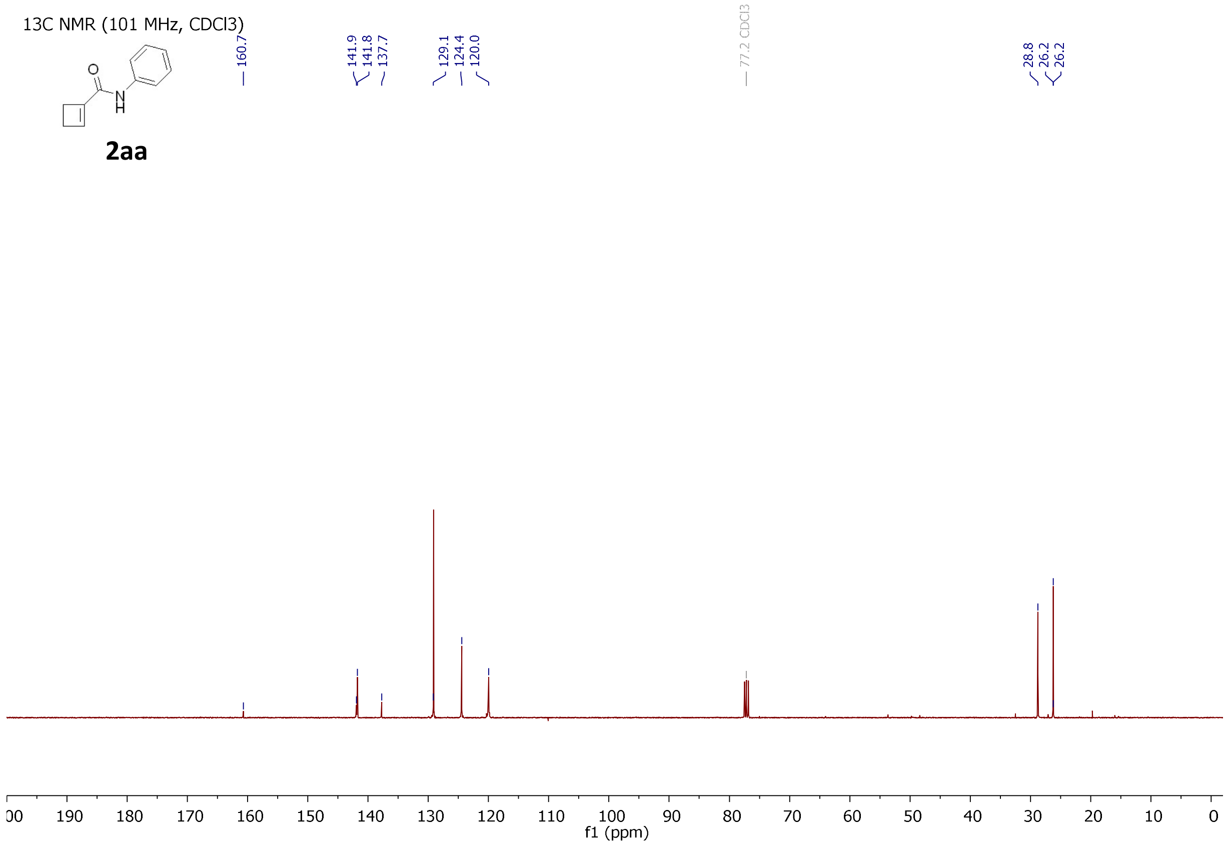
13C NMR (101 MHz, CDCl3)
2aa
160.7
141.9
141.8
137.7
129.1
124.4
120.0
77.2 CDCl3
28.8
26.2
26.2
f1 (ppm)

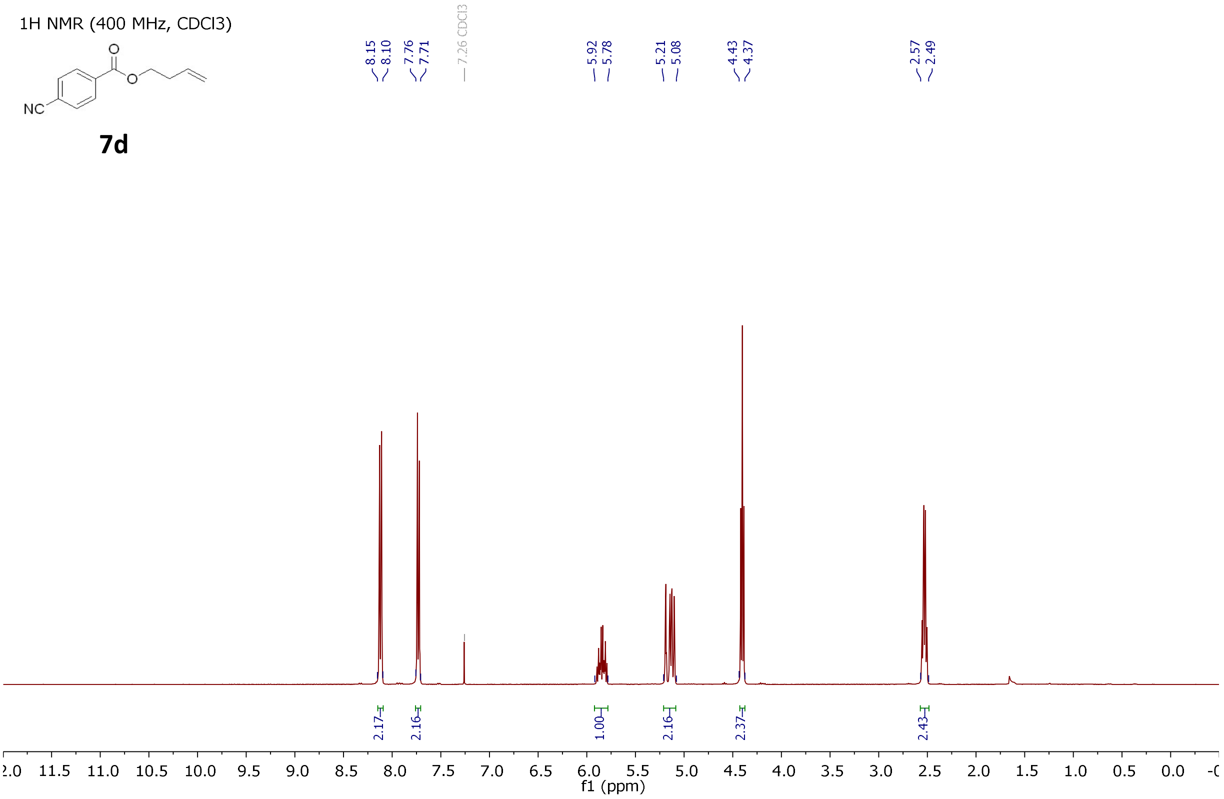
1H NMR (400 MHz, CDCl3)
7d
8.15
8.10
7.76
7.71
7.26 CDCl3
5.92
5.78
5.21
5.08
4.43
4.37
2.57
2.49
2.17
2.16
1.00
2.16
2.37
2.43
f1 (ppm)

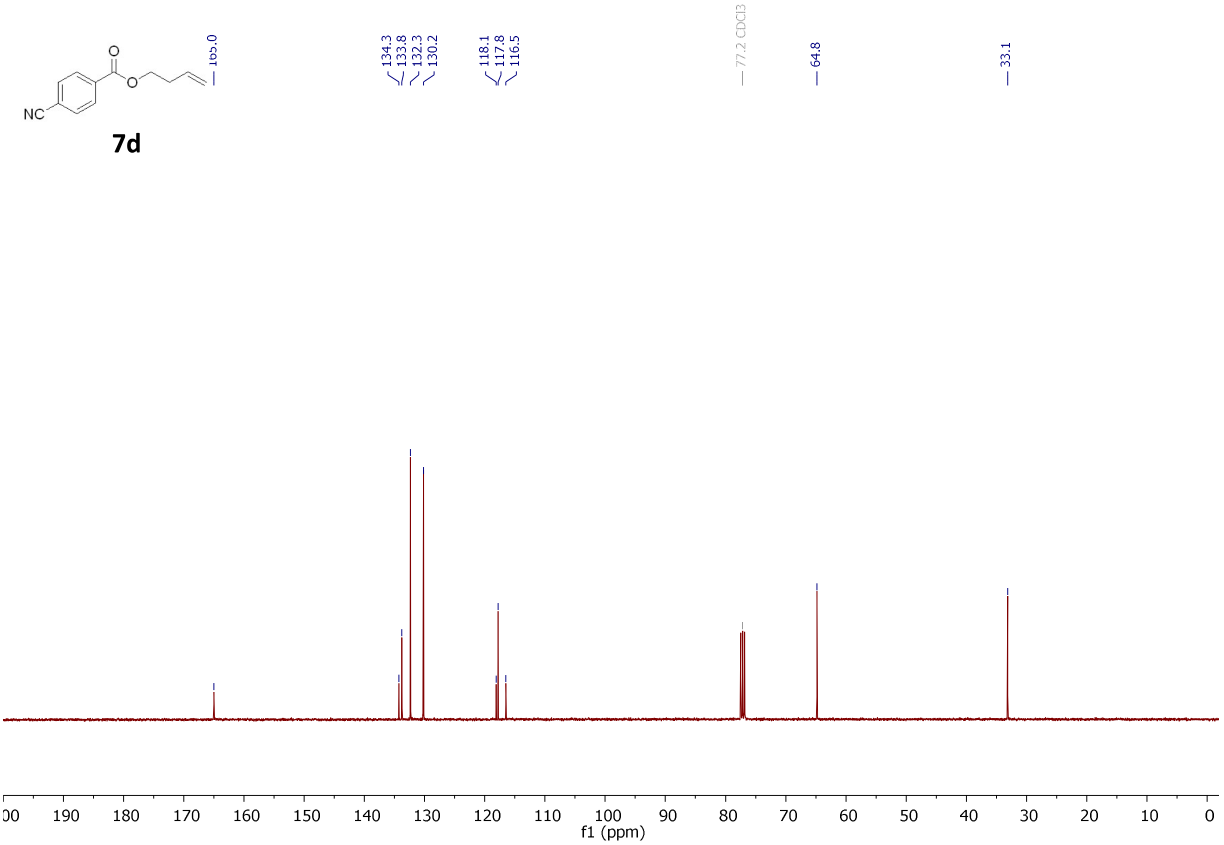
7d
134.3
133.8
132.3
130.2
118.1
117.8
116.5
77.2 CDCl3
64.8
33.1
f1 (ppm)

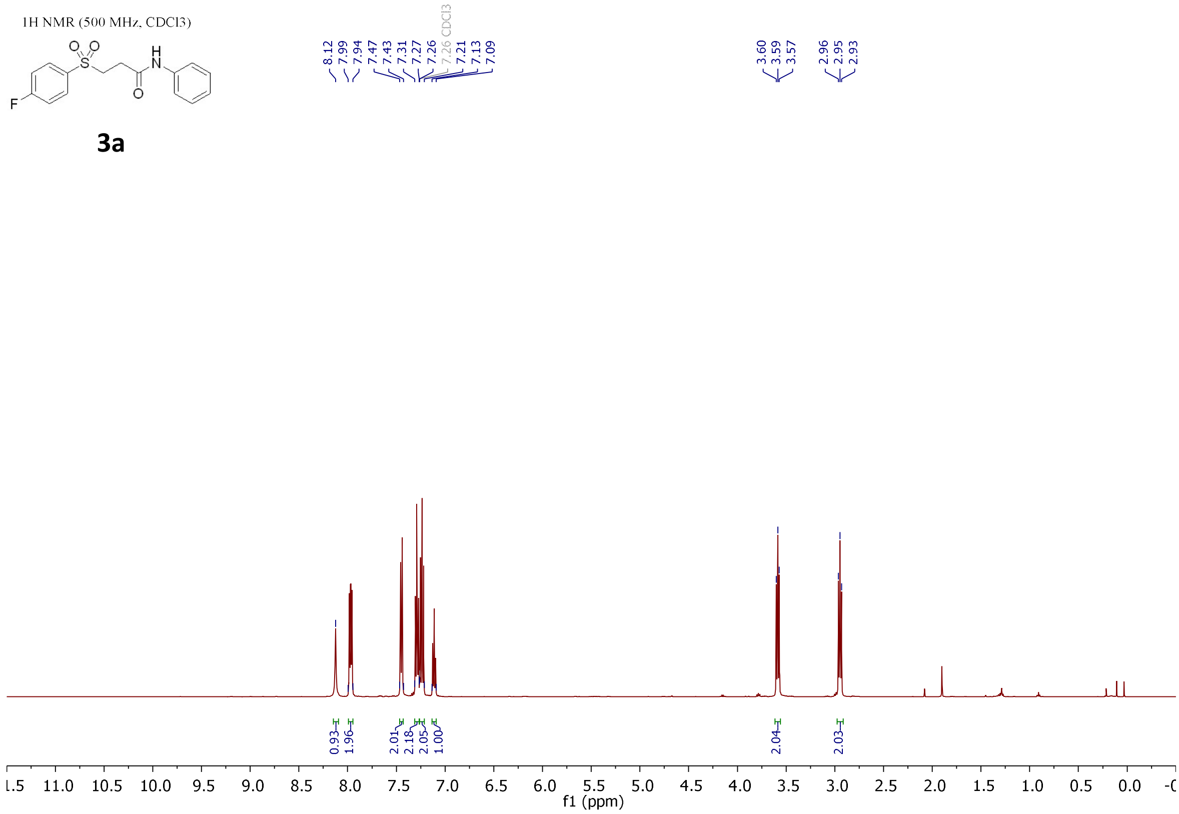
1H NMR (500 MHz, CDCl3)
F
O O
S
H
N
O
3a
8.12
7.99
7.94
7.47
7.43
7.31
7.27
7.26
7.26 CDCl3
7.21
7.13
7.09
3.60
3.59
3.57
2.96
2.95
2.93
0.93
1.96
2.01
2.18
2.05
1.00
2.04
2.03
f1 (ppm)

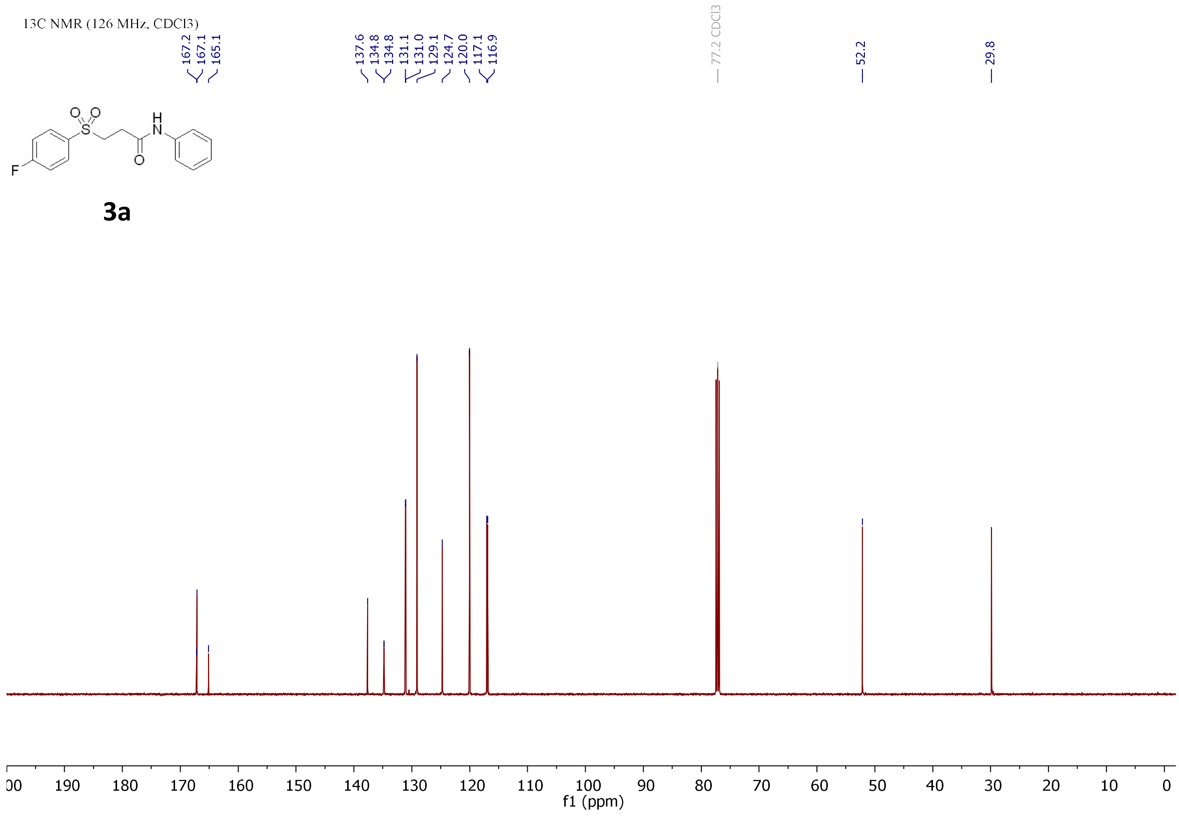
13C NMR (126 MHz, CDCl3)
167.2
167.1
165.1
137.6
134.8
134.8
131.1
131.0
129.1
124.7
120.0
117.1
116.9
77.2 CDCl3
52.2
29.8
F
O O
S
H
N
O
3a
f1 (ppm)

19F NMR (471 MHz, CDCl3)

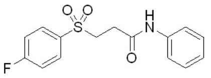

3a

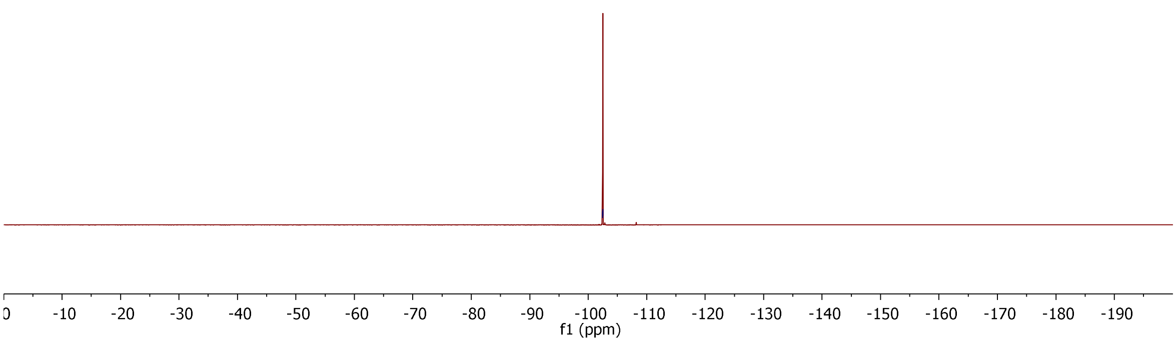

1H NMR (400 MHz, CDCl3)

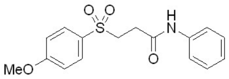

3b

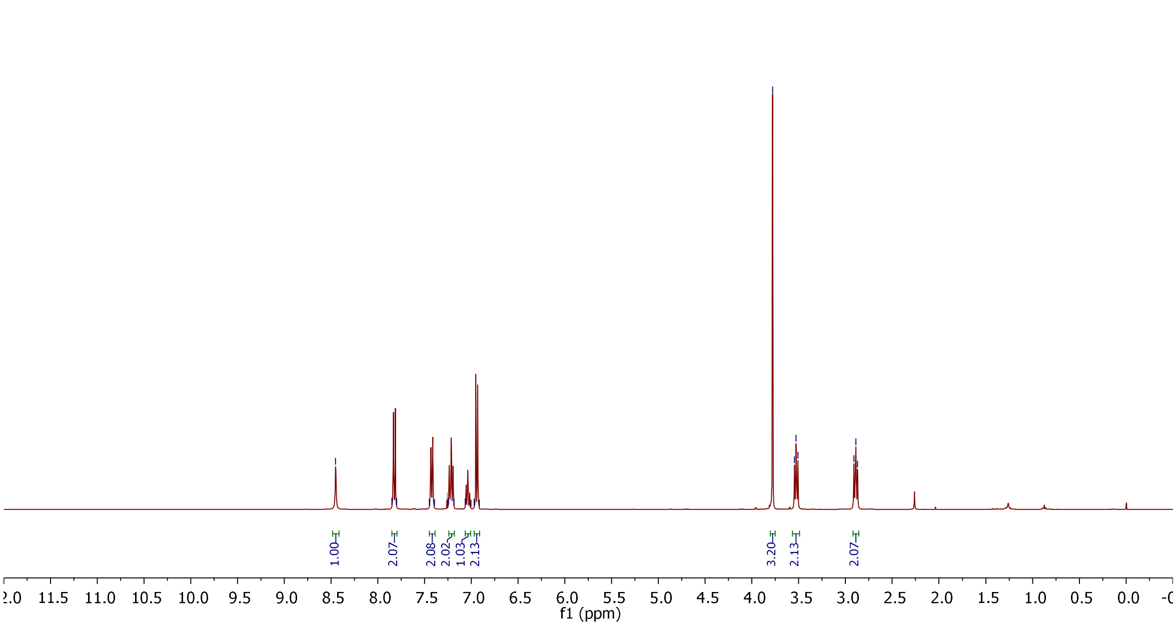

13C NMR (101 MHz, CDCl3)

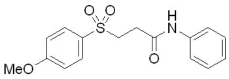

3b

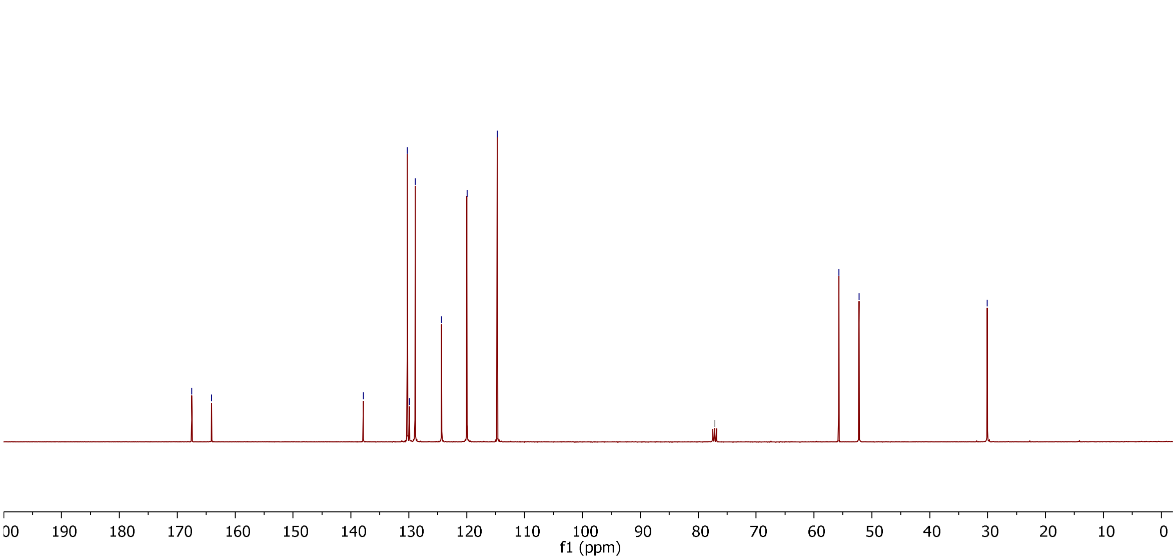

1H NMR (500 MHz, DMSO-d6)

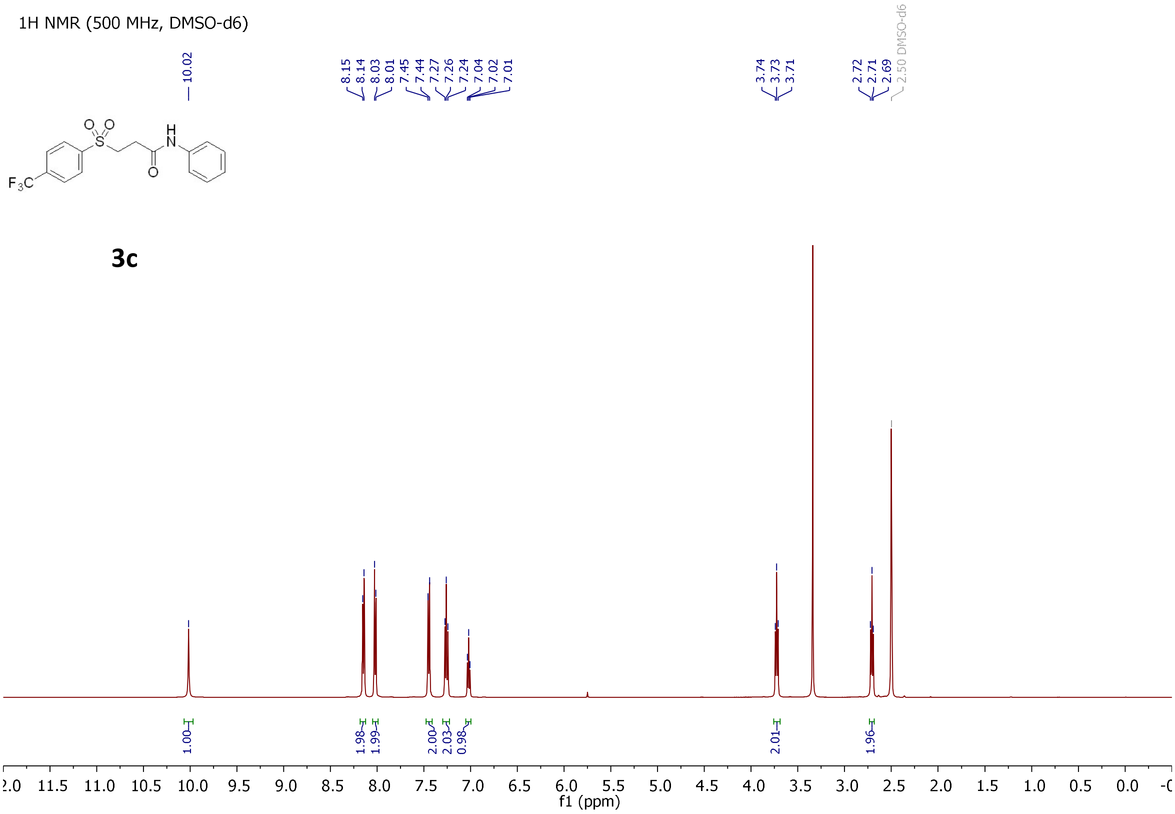

13C NMR (126 MHz, DMSO-d6)

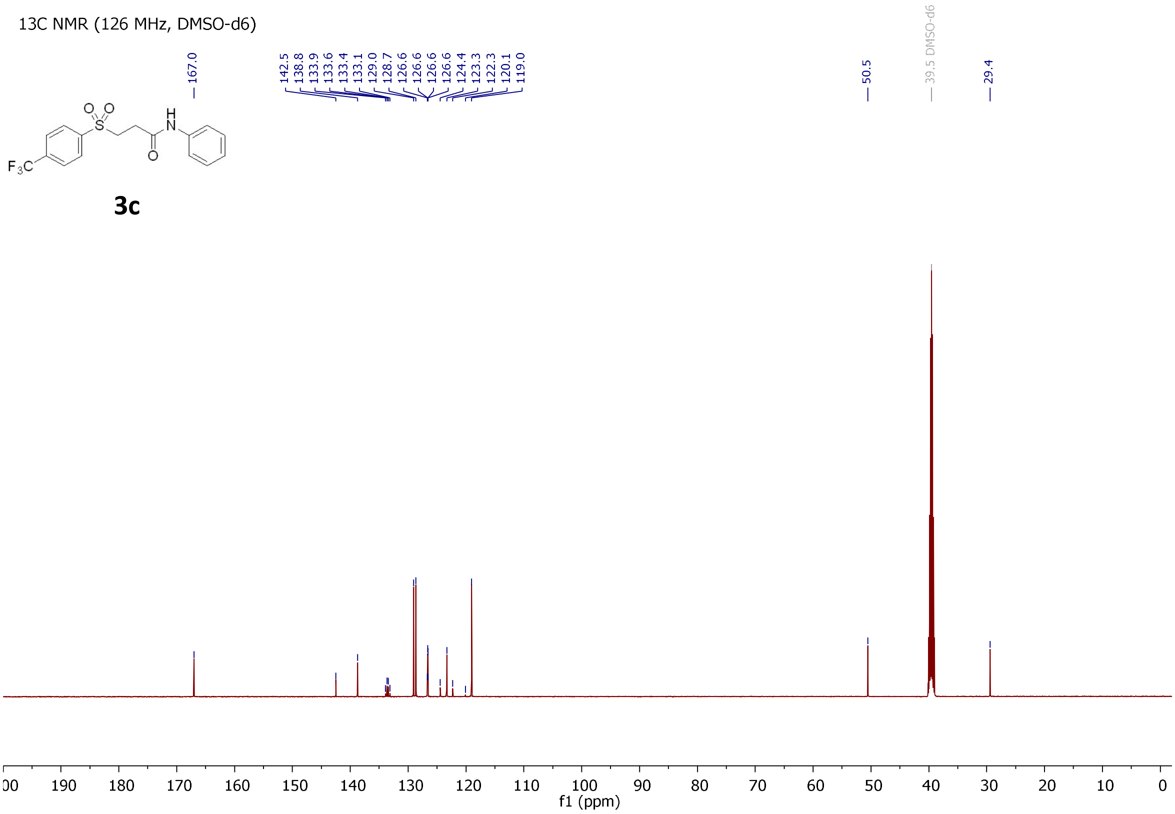

19F NMR (471 MHz, DMSO-d6)

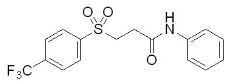

3c

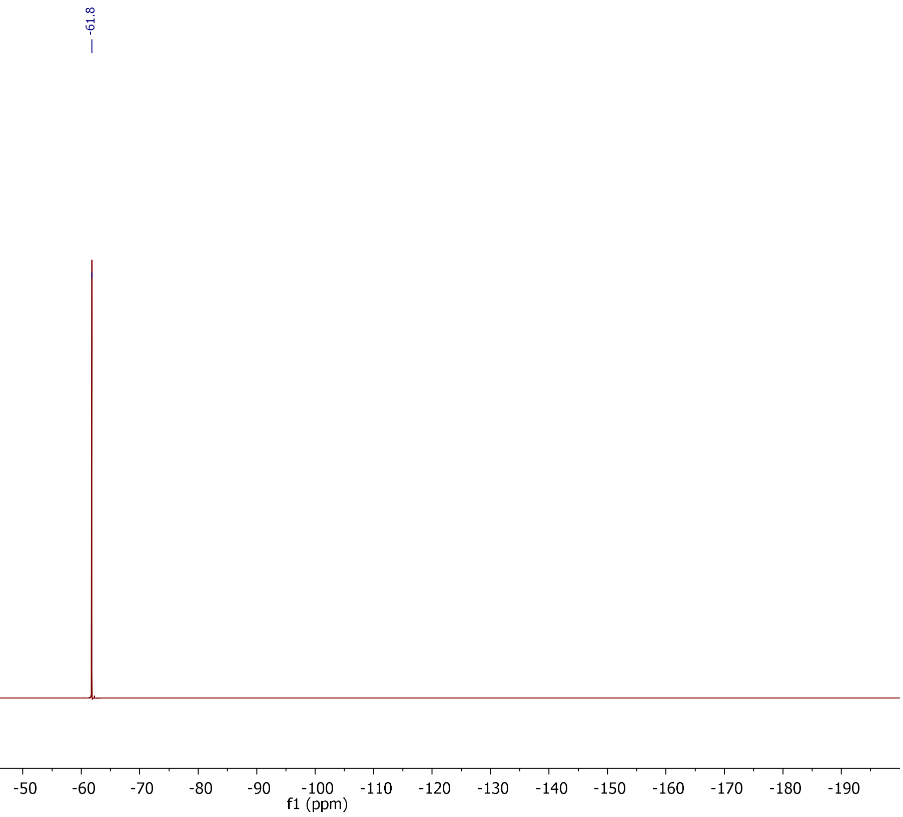

1H NMR (400 MHz, DMSO-d6)

3d

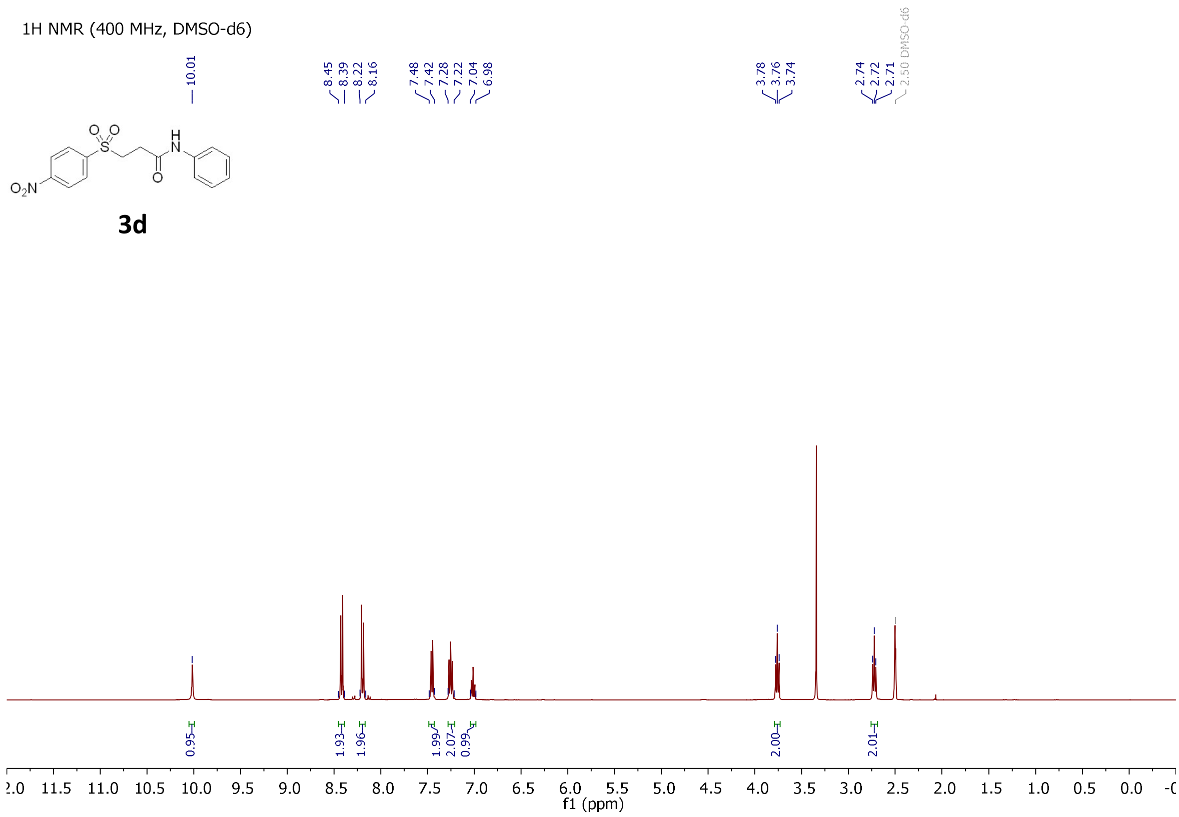

13C NMR (101 MHz, DMSO-d6)

3d

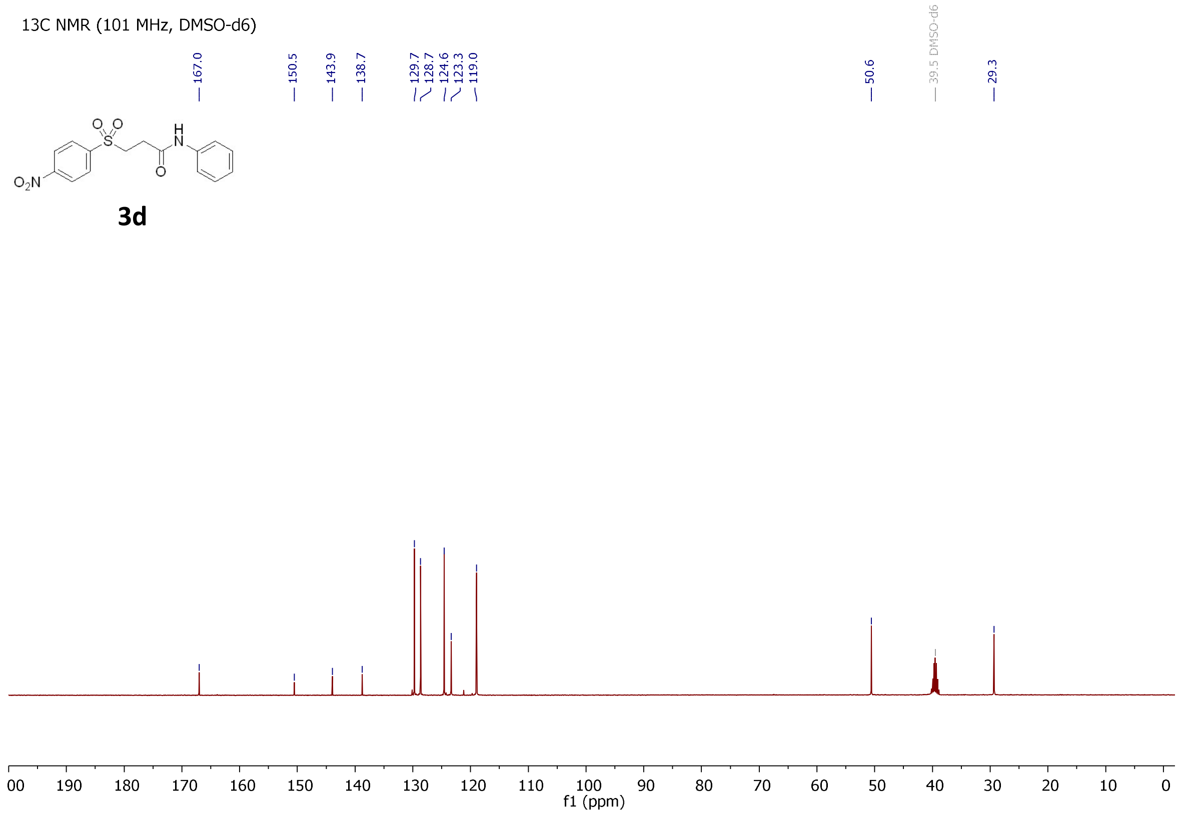

1H NMR (400 MHz, DMSO-d6)

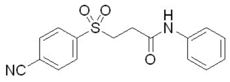

3e

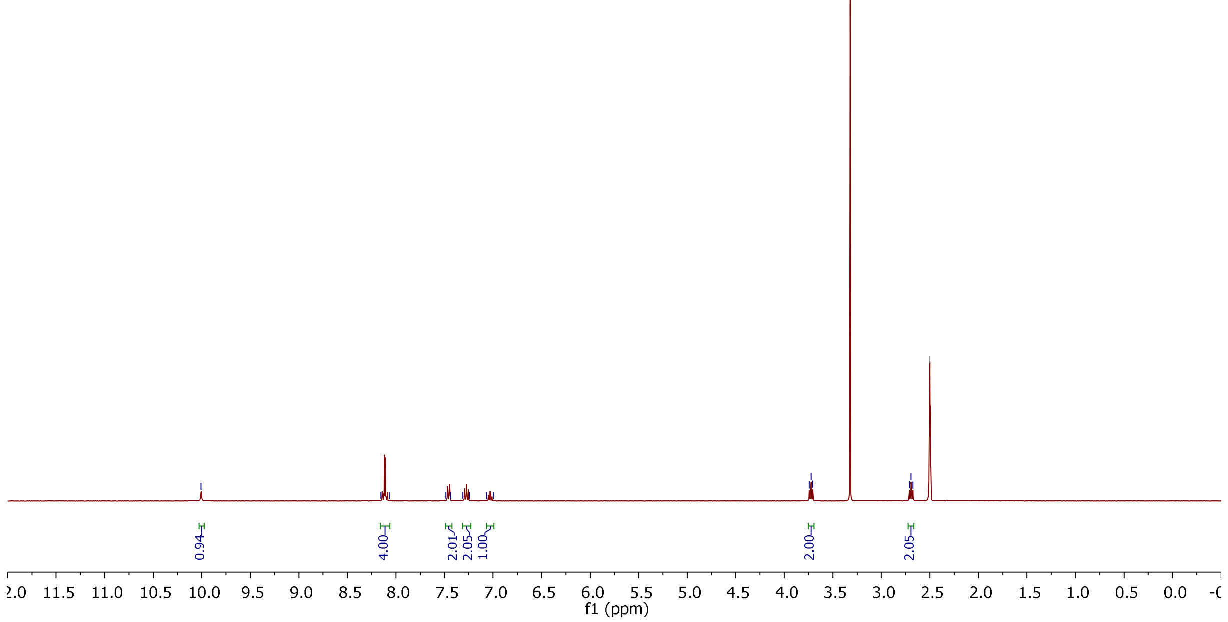

13C NMR (101 MHz, DMSO-d6)

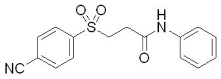

3e

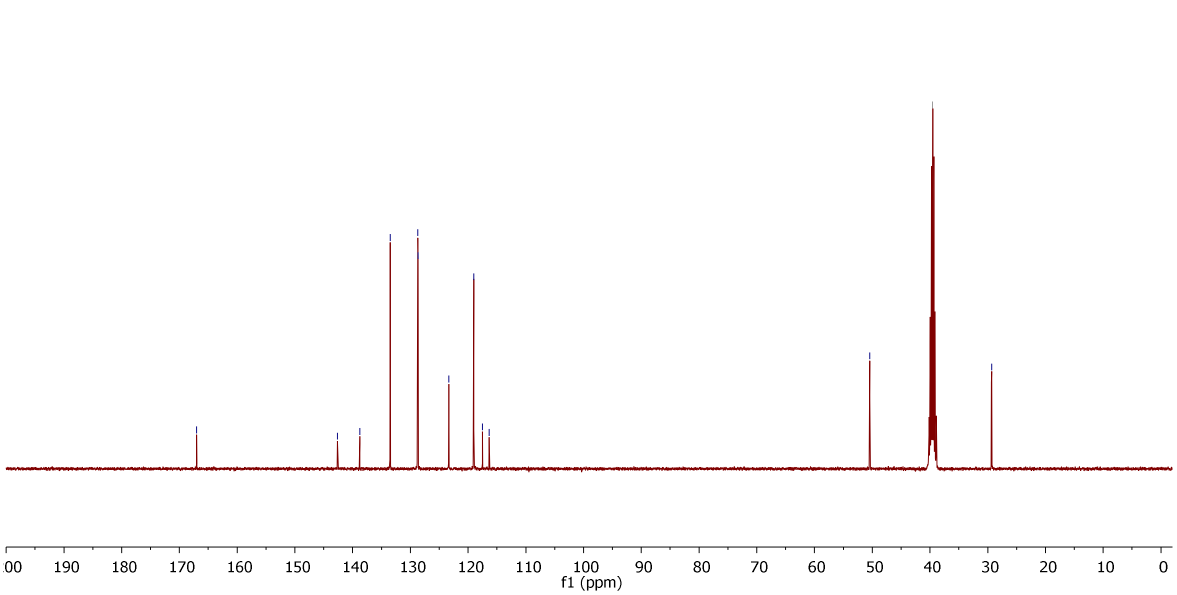

1H NMR (400 MHz, CDCl3)

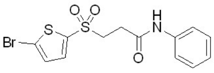

3f

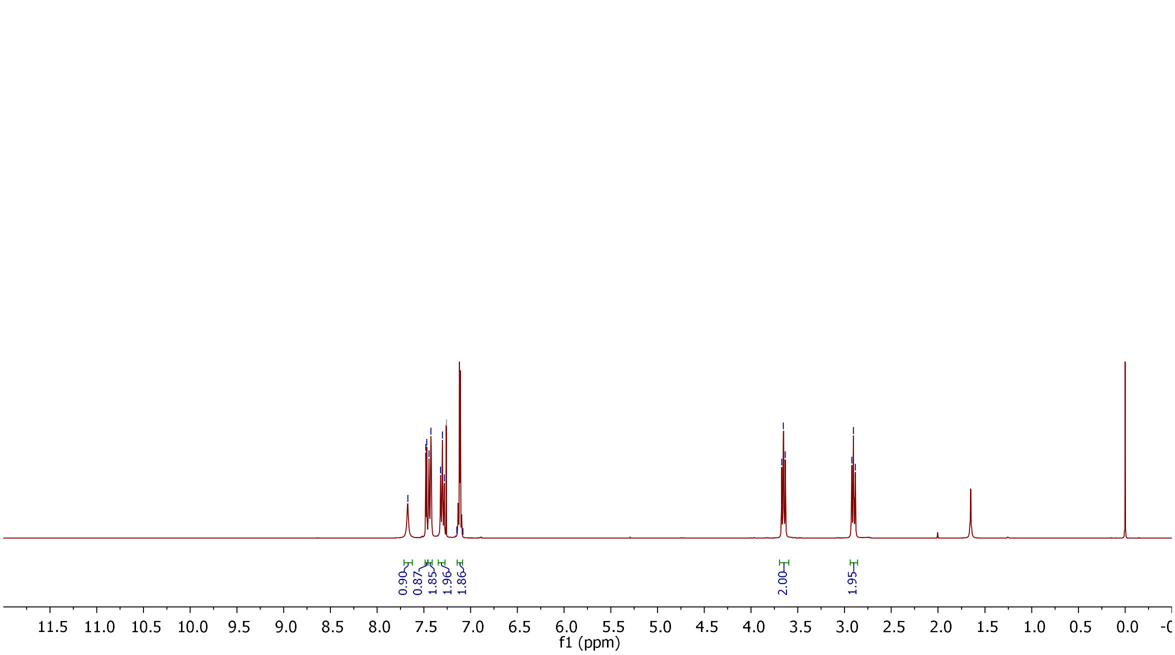

13C NMR (101 MHz, CDCl3)

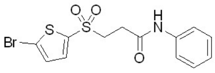

3f

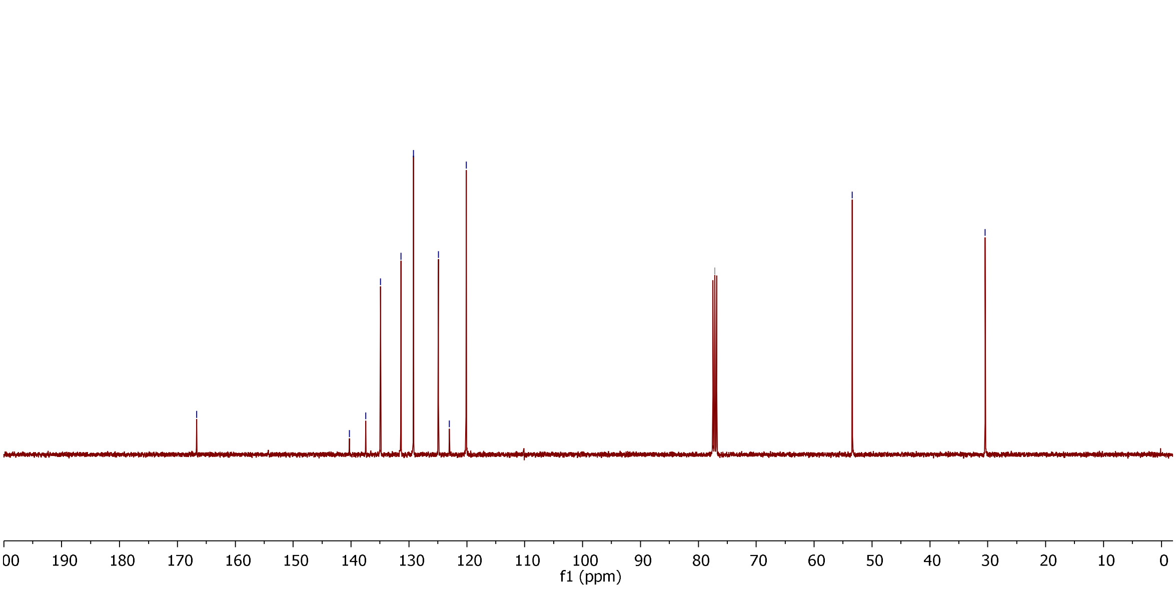

1H NMR (400 MHz, DMSO-d6)

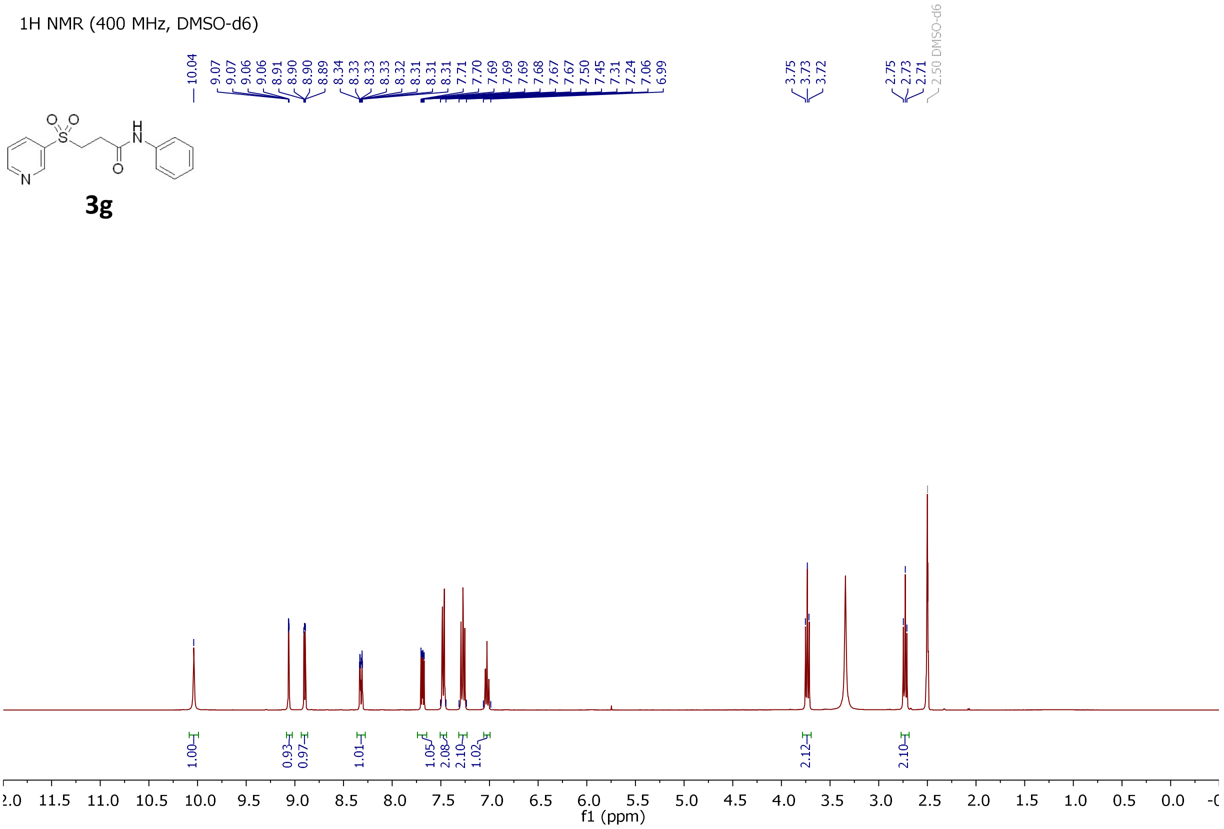

13C NMR (101 MHz, DMSO)-d6

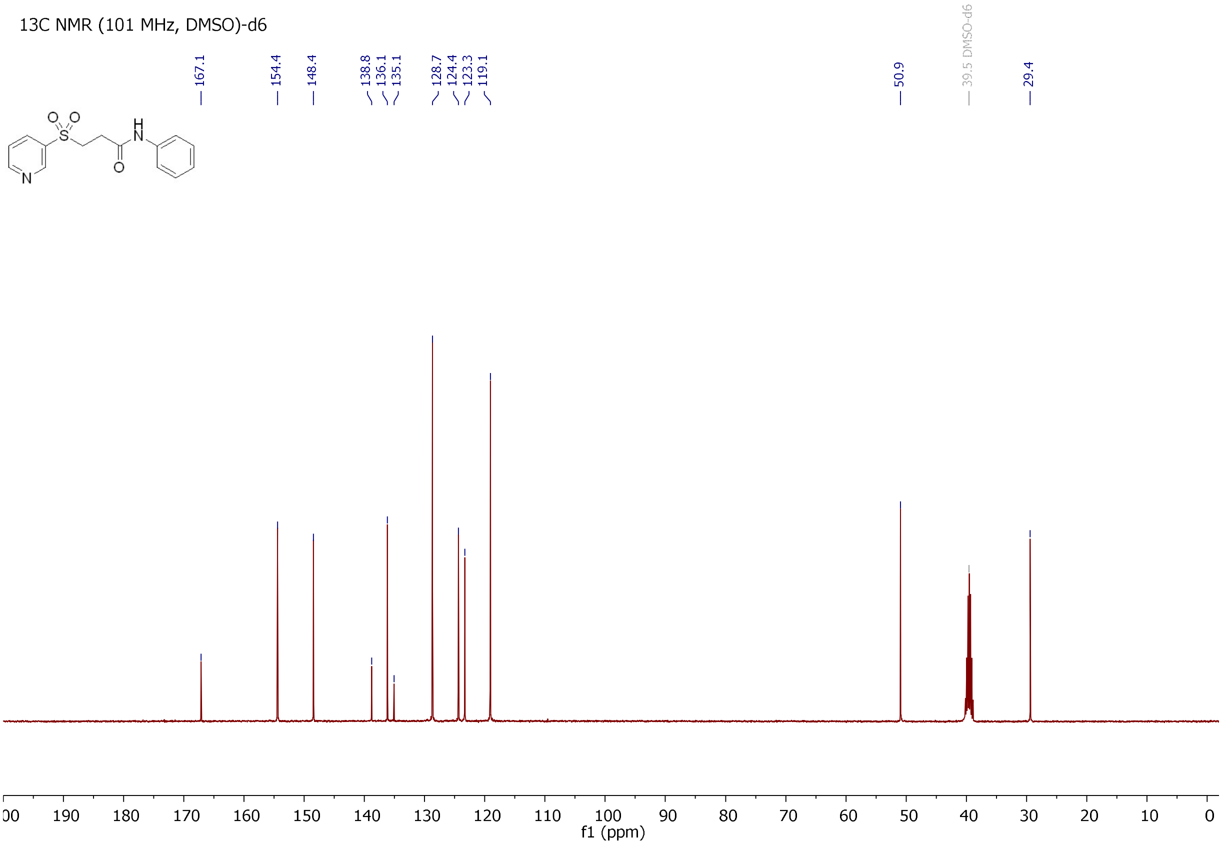

1H NMR (500 MHz, DMSO-d6)

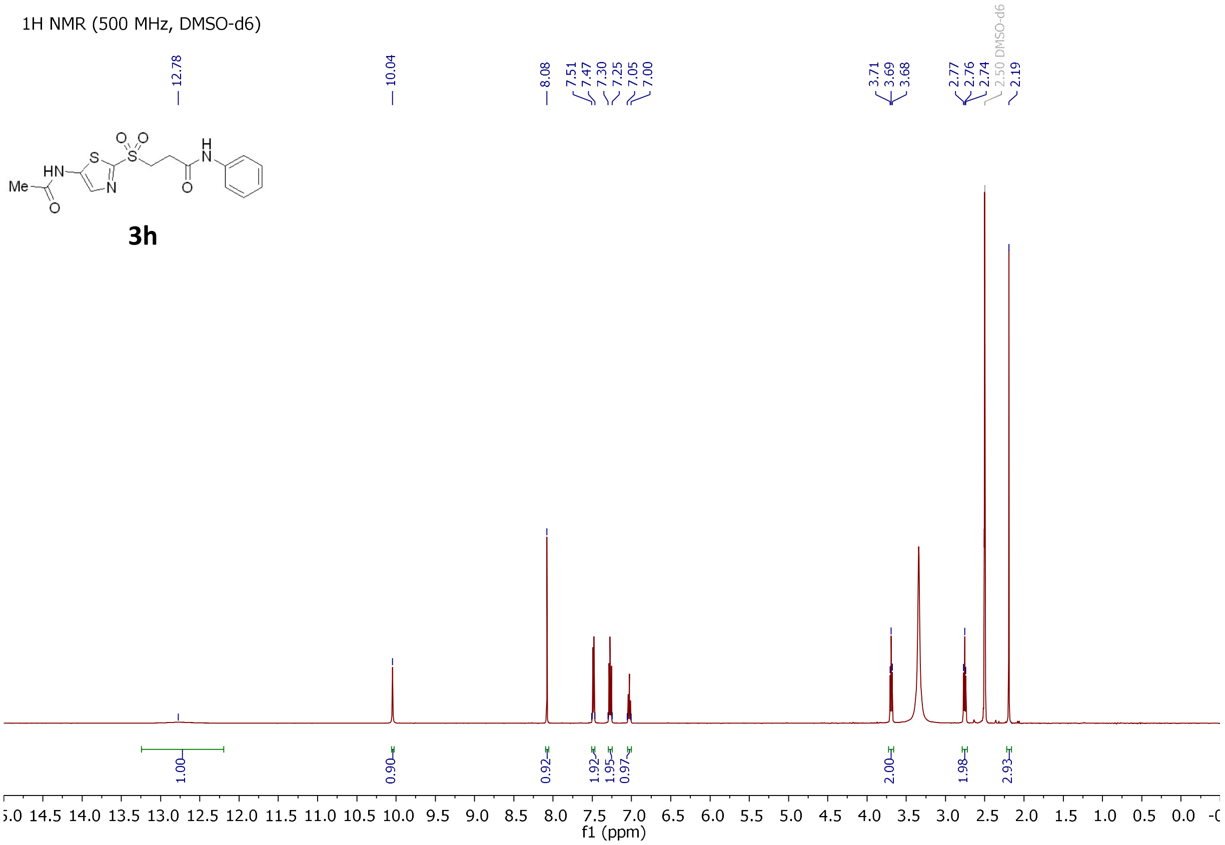

13C NMR (126 MHz, DMSO-d6)

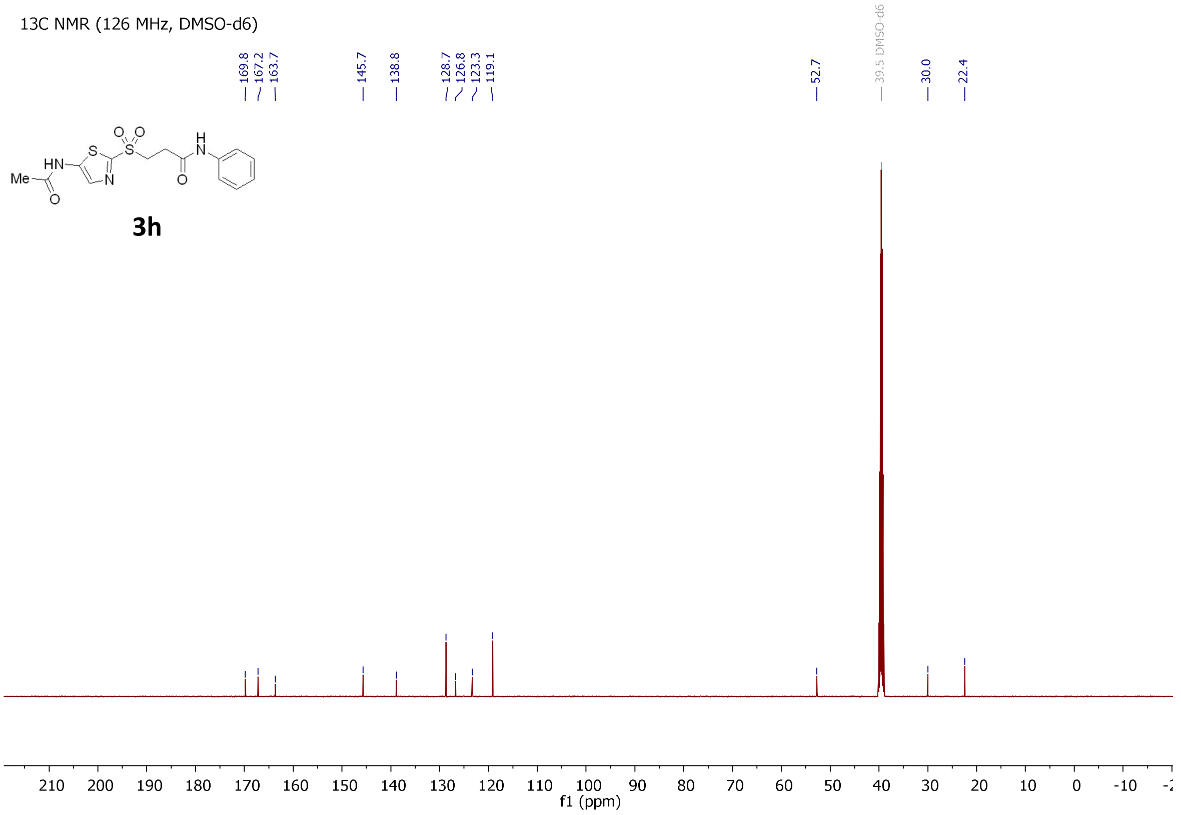

1H NMR (400 MHz, CDCl3)

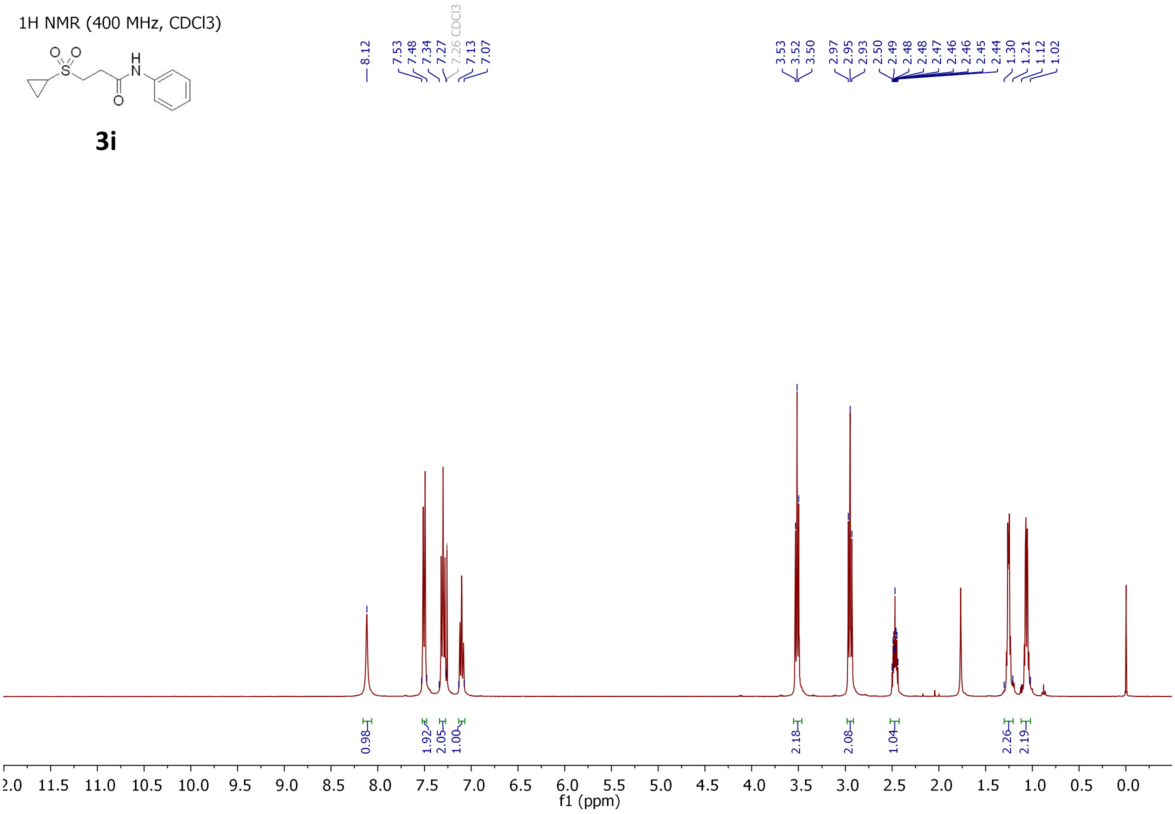

3i

13C NMR (101 MHz, CDCl3)

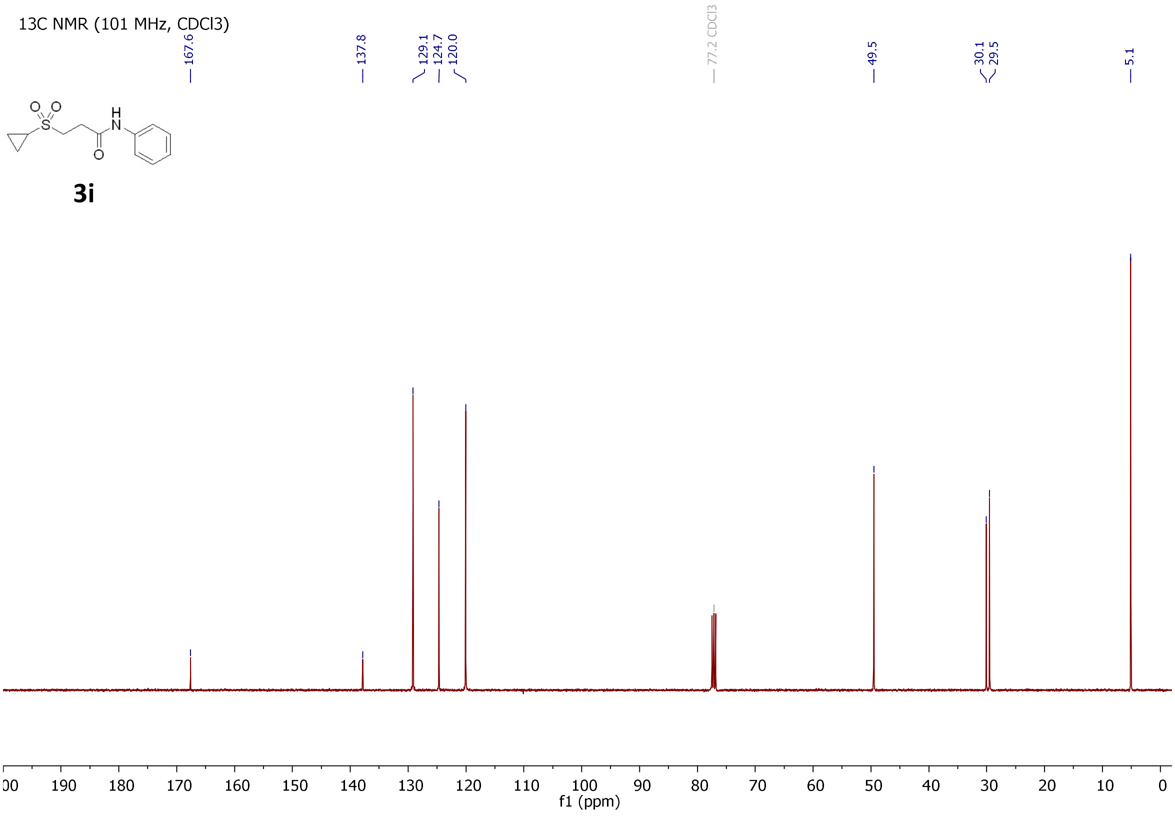

3i

1H NMR (400 MHz, CDCl3)

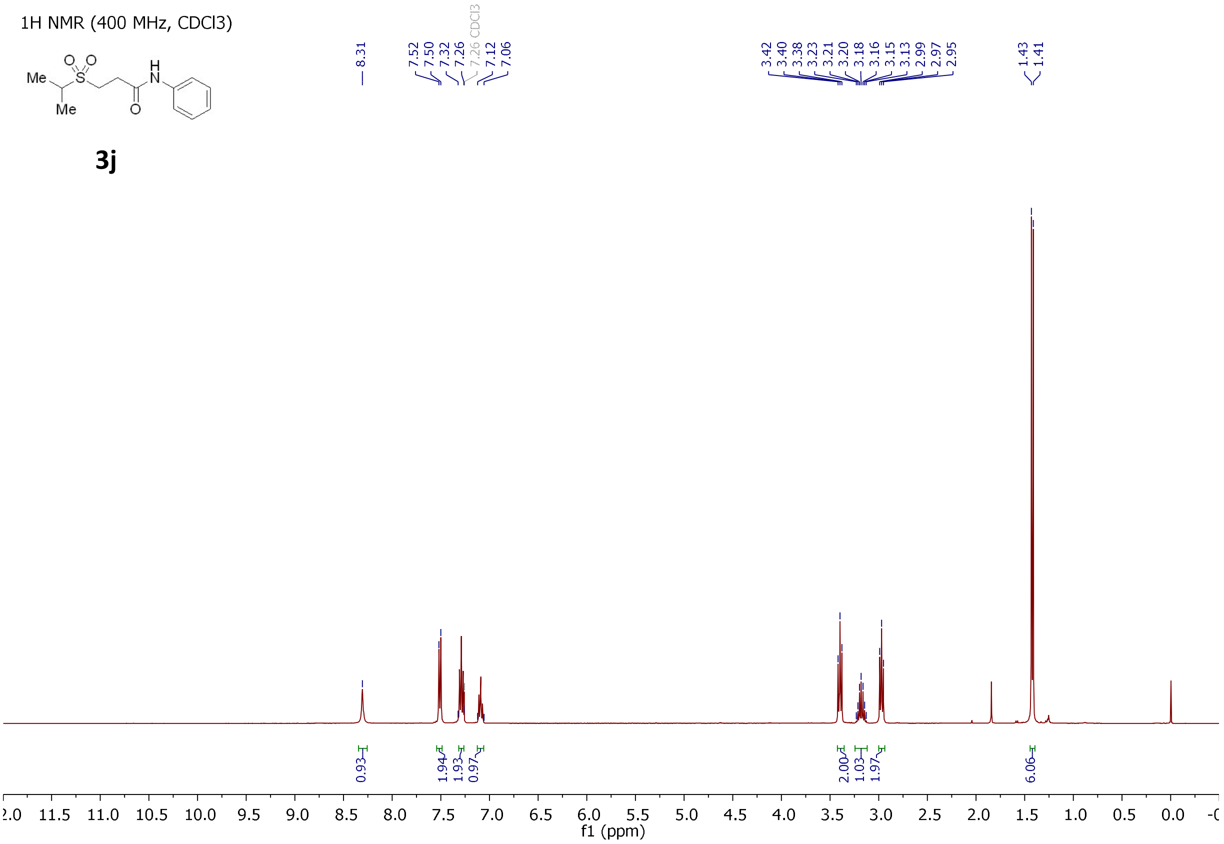

13C NMR (101 MHz, CDCl3)

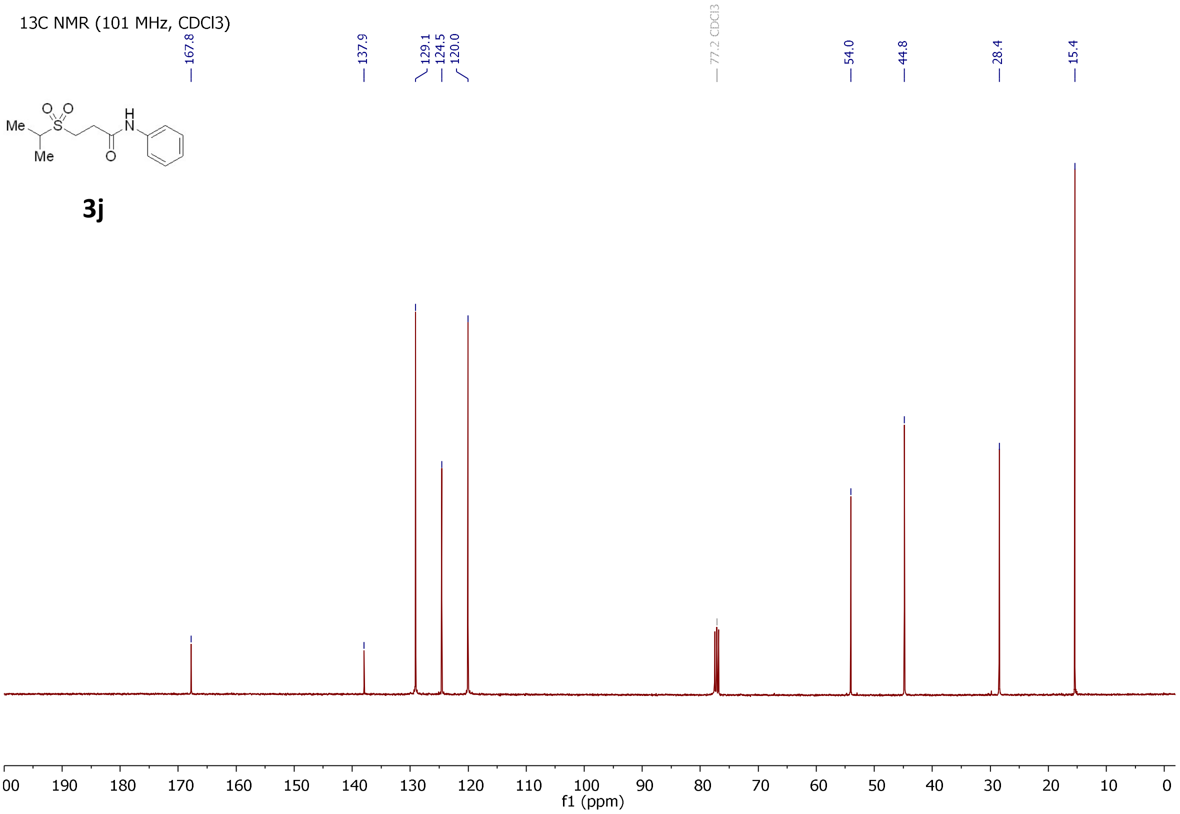

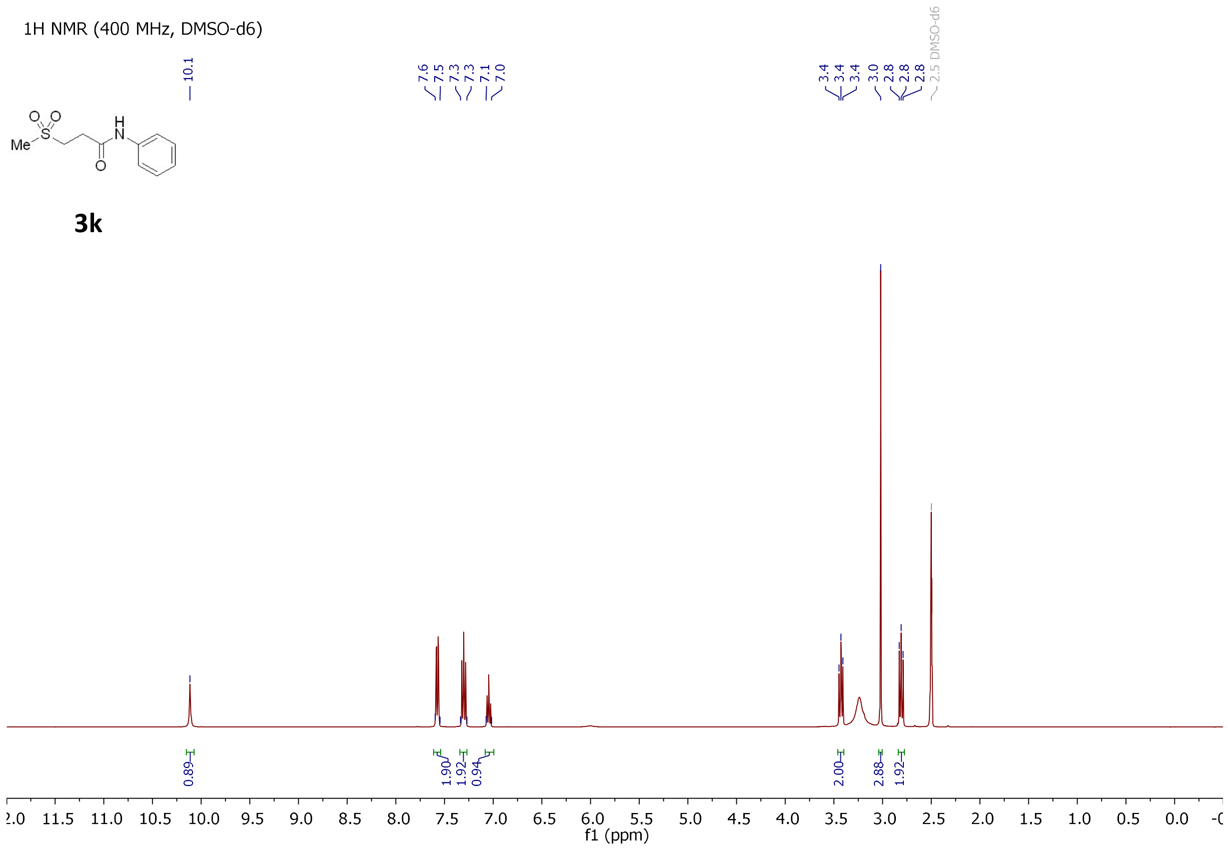
1H NMR (400 MHz, DMSO-d6)
10.1
7.6
7.5
7.3
7.3
7.1
7.0
3.4
3.4
3.4
3.0
2.8
2.8
2.8
2.5 DMSO-d6
Me
S
O
O
H
N
O
3k
0.89
1.90
1.92
0.94
2.00
2.88
1.92
f1 (ppm)

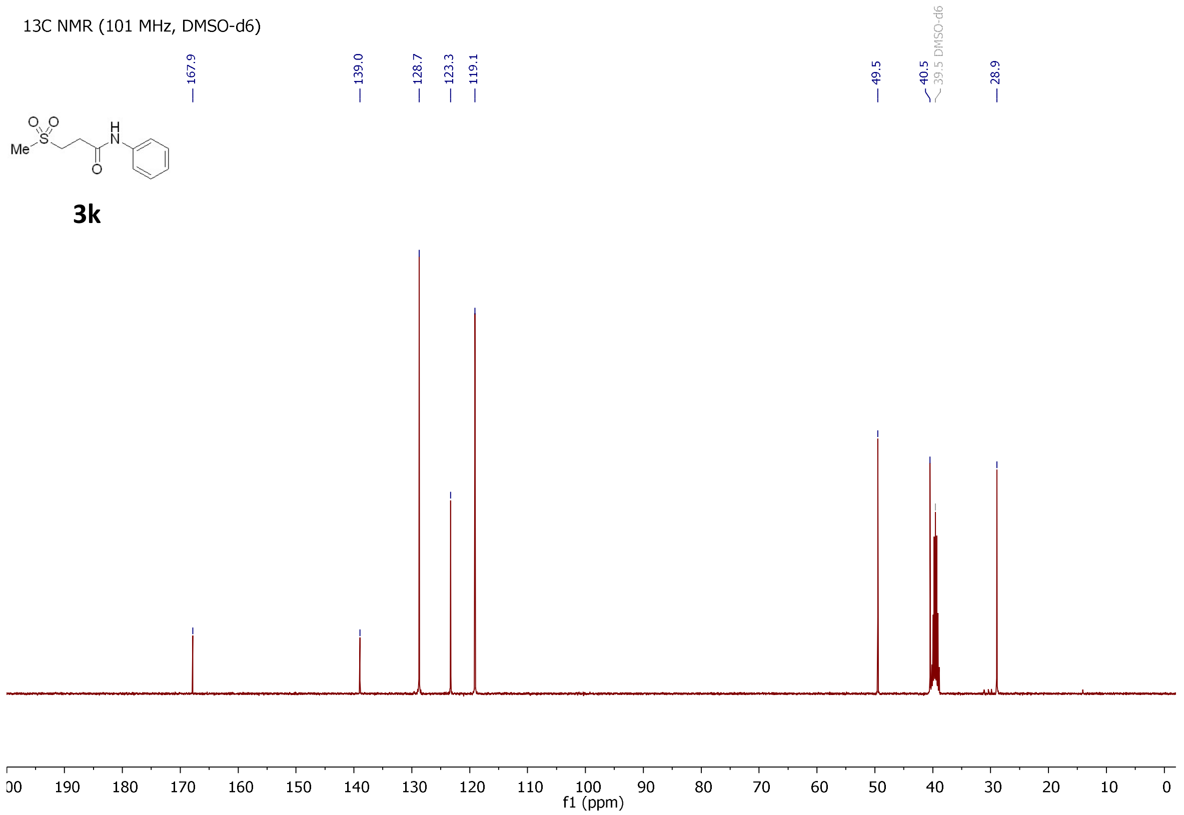
13C NMR (101 MHz, DMSO-d6)
167.9
139.0
128.7
123.3
119.1
49.5
40.5
39.5 DMSO-d6
28.9
Me
S
O
O
H
N
O
3k
f1 (ppm)

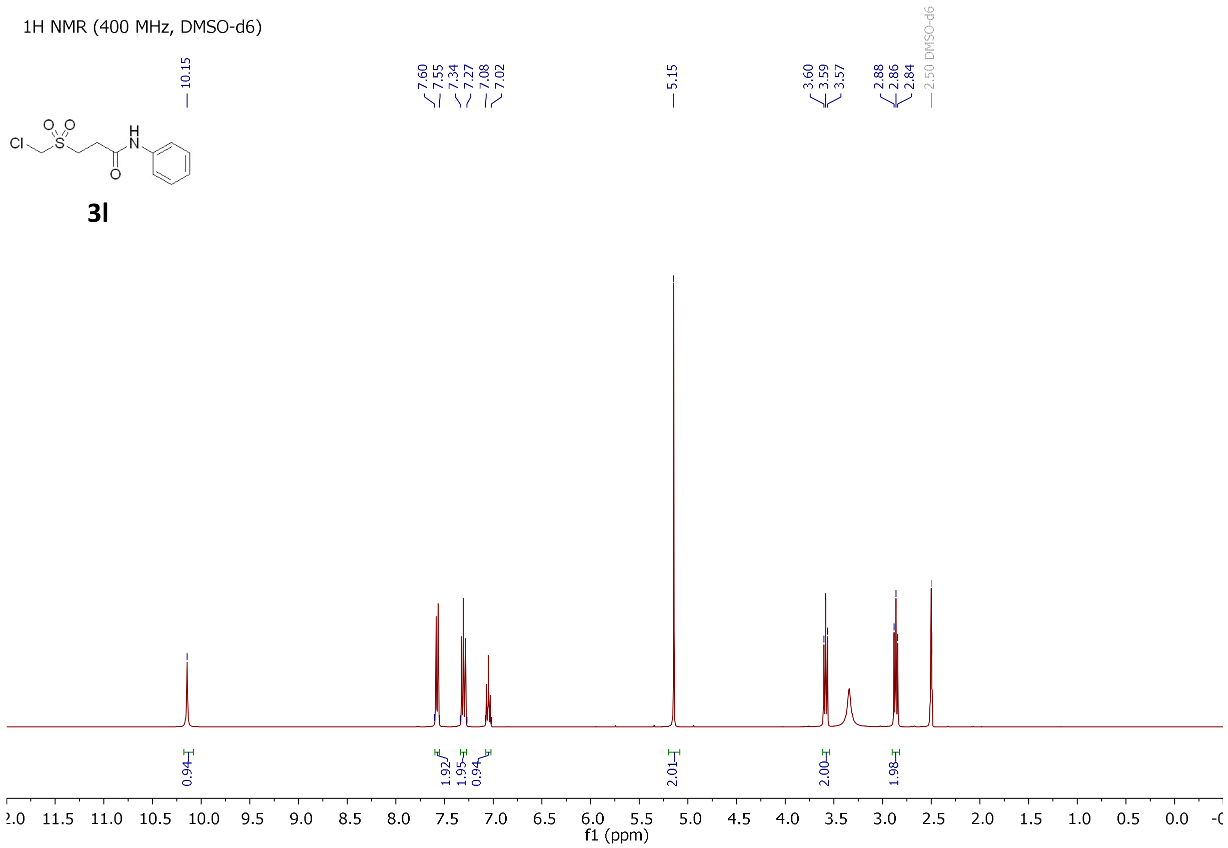
1H NMR (400 MHz, DMSO-d6)
10.15
7.60
7.55
7.34
7.27
7.08
7.02
5.15
3.60
3.59
3.57
2.88
2.86
2.84
2.50 DMSO-d6
Cl
S
O
O
H
N
O
3l
0.94
1.92
1.95
0.94
2.01
2.00
1.98
f1 (ppm)

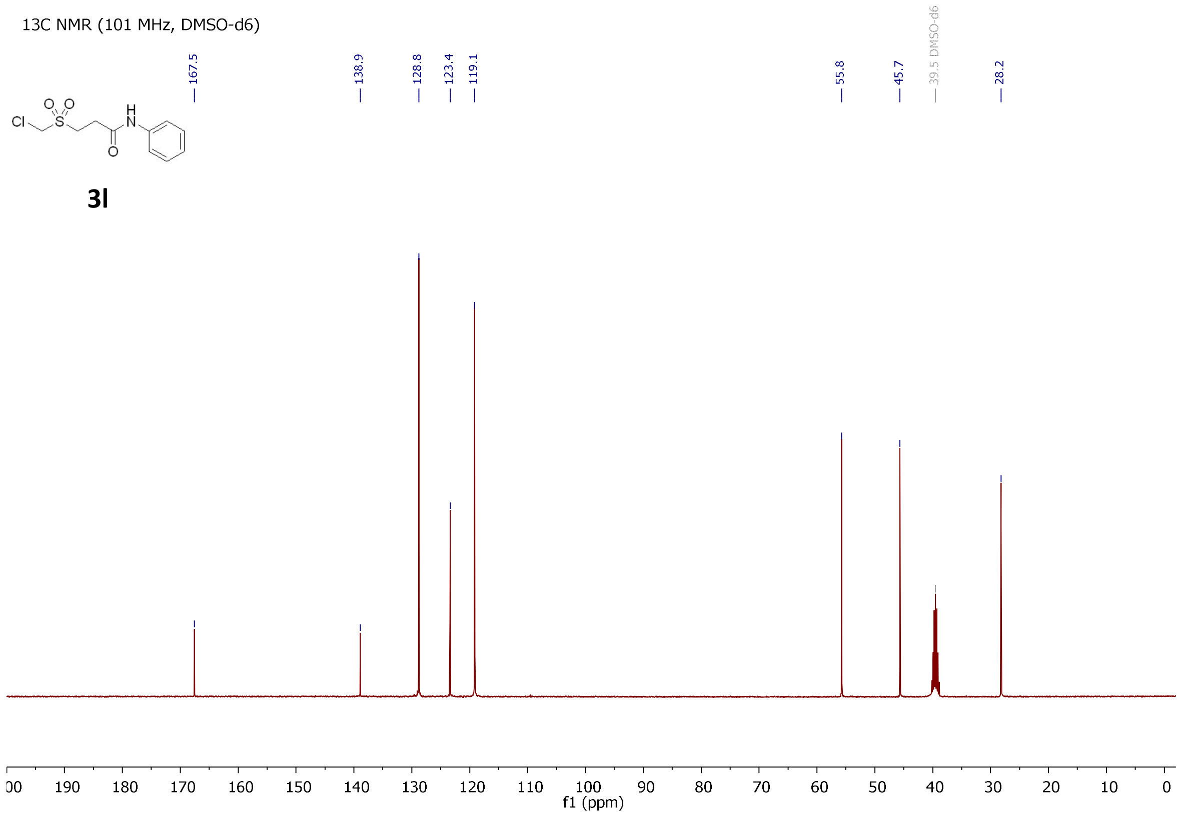
13C NMR (101 MHz, DMSO-d6)
167.5
138.9
128.8
123.4
119.1
55.8
45.7
39.5 DMSO-d6
28.2
Cl
S
O
O
H
N
O
3l
f1 (ppm)

1H NMR (400 MHz, DMSO-d6)

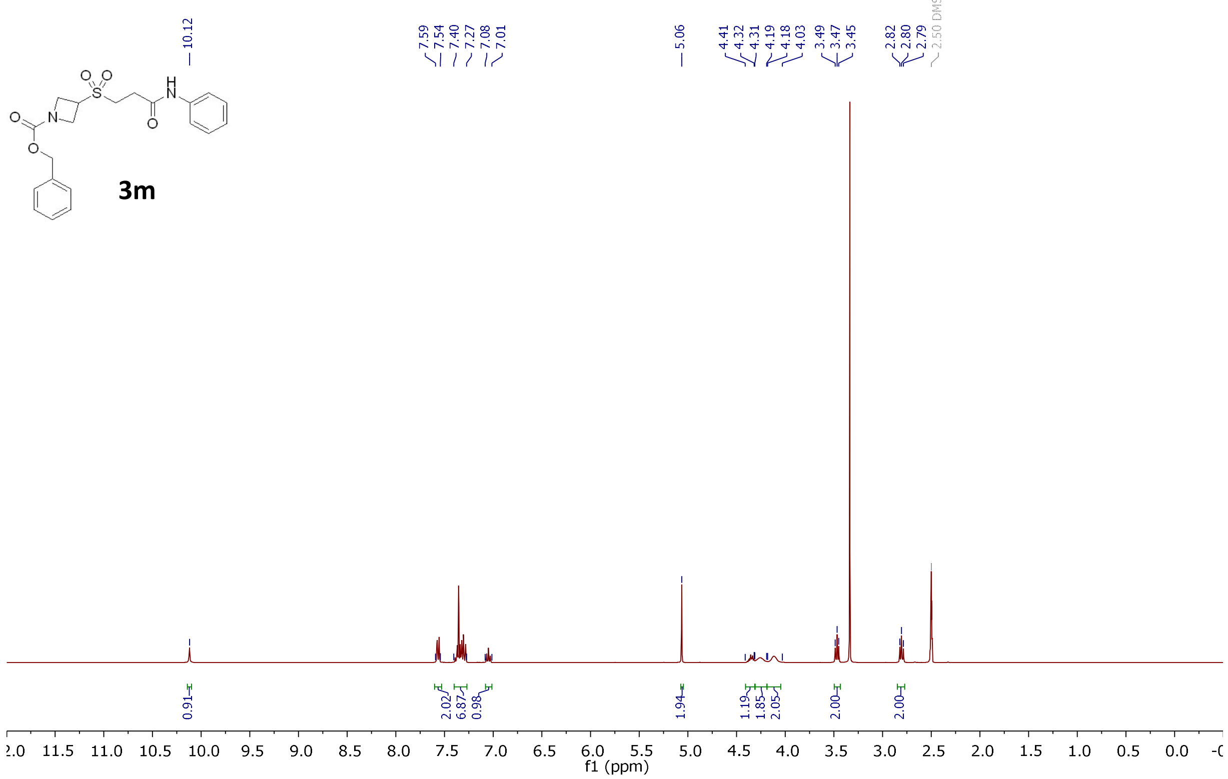

1H NMR (400 MHz, DMSO-d6)

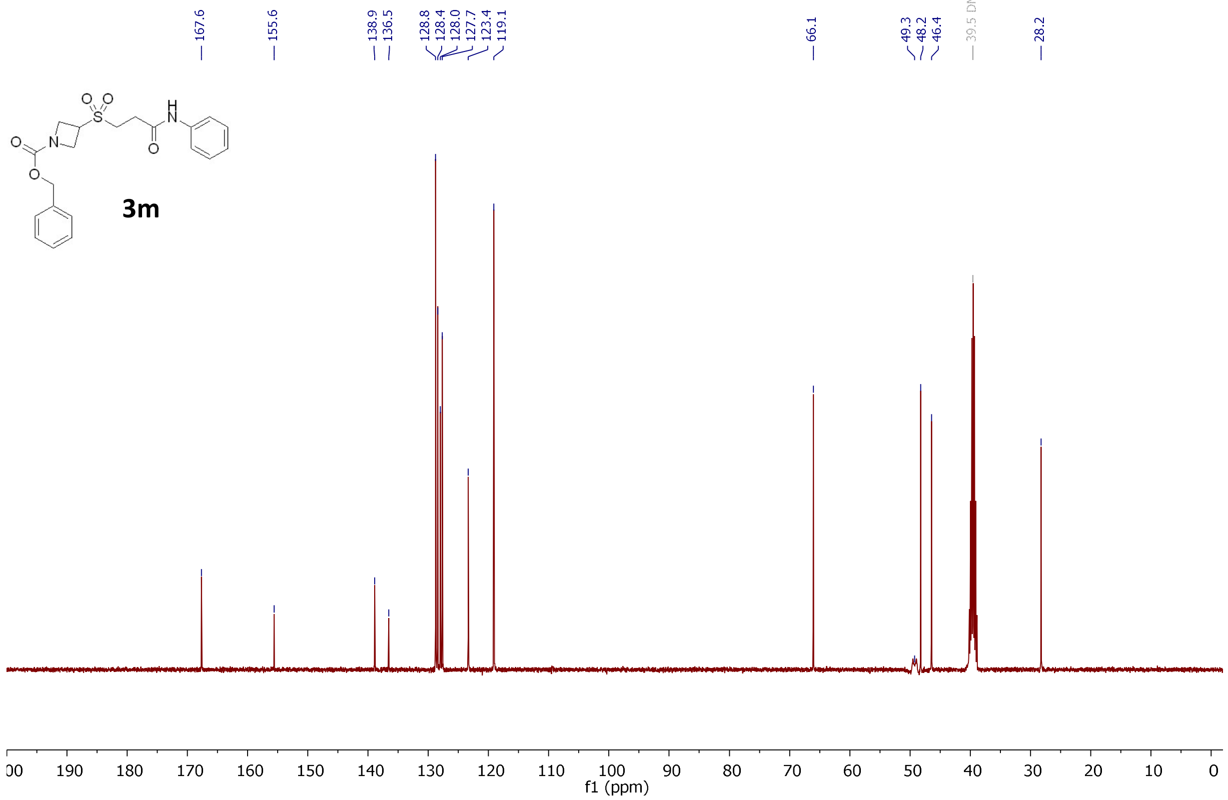

1H NMR (500 MHz, CDCl3)

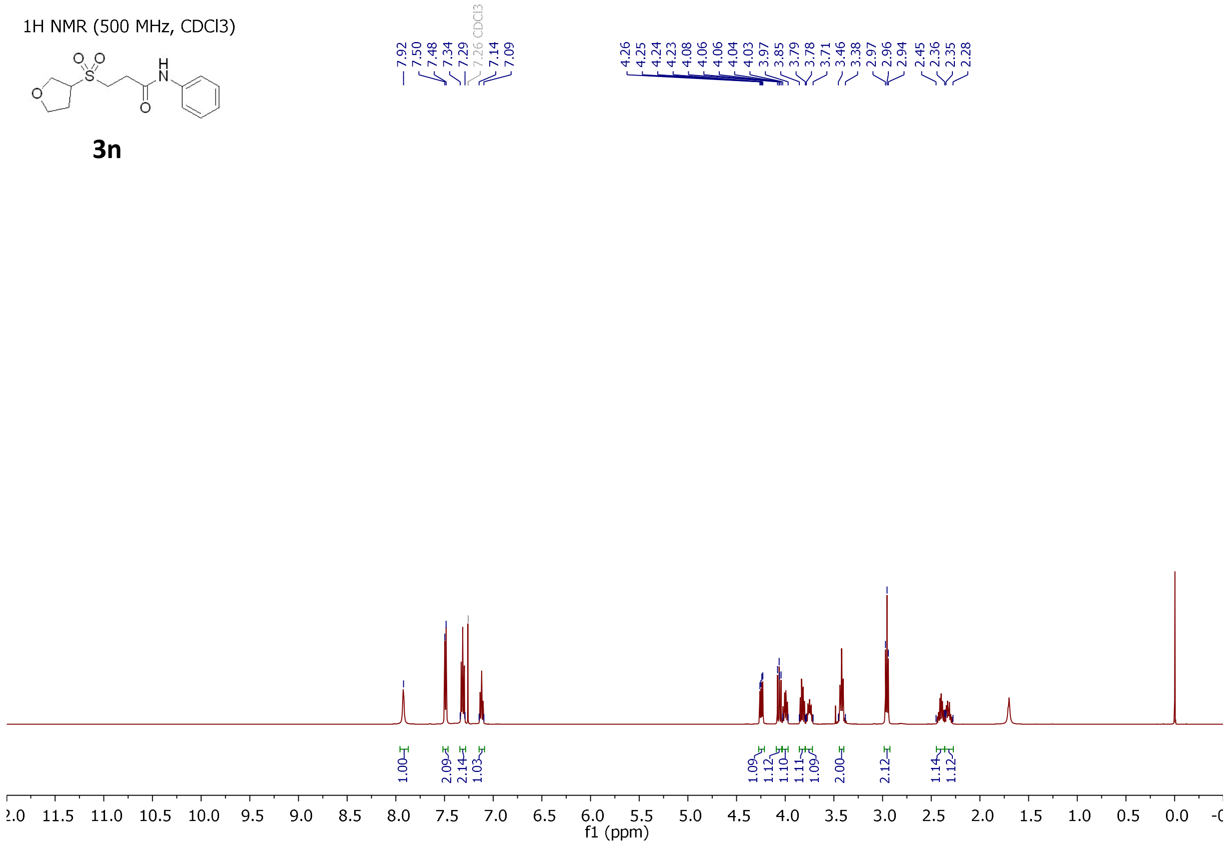

13C NMR (126 MHz, CDCl3)

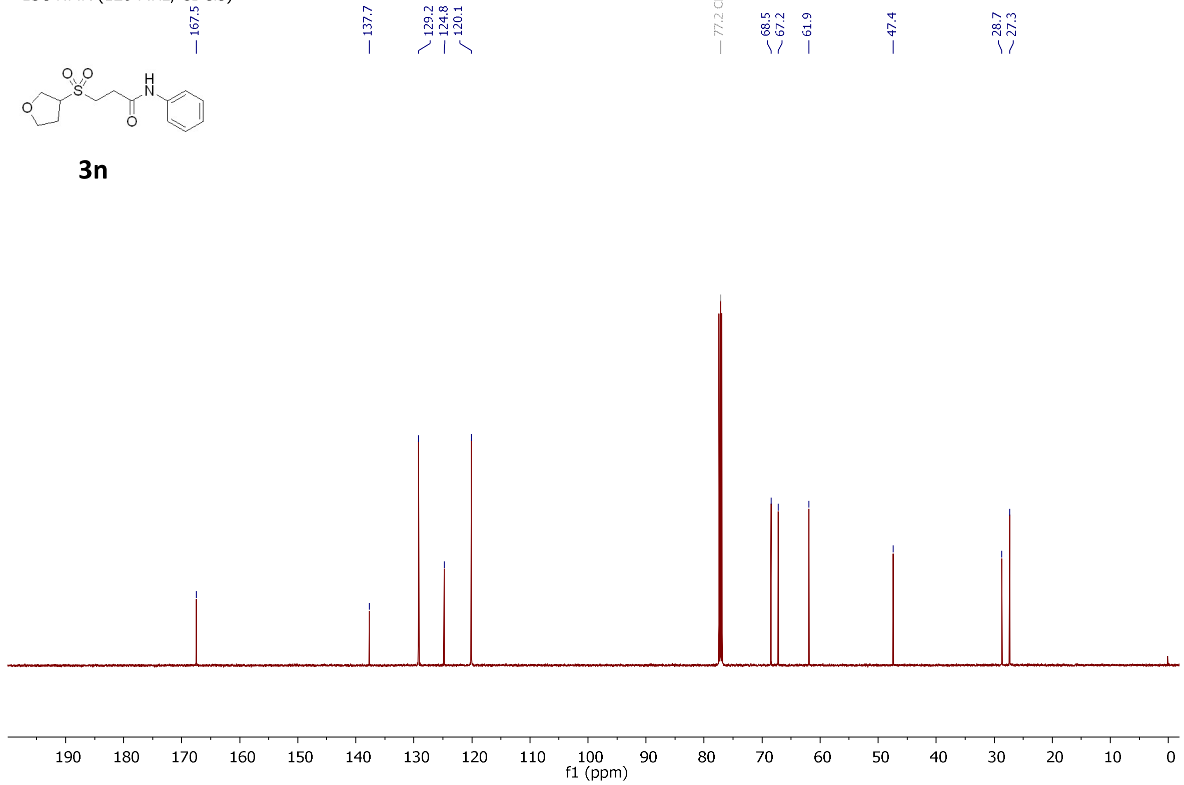

1H NMR (400 MHz, CDCl3)

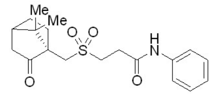

3o

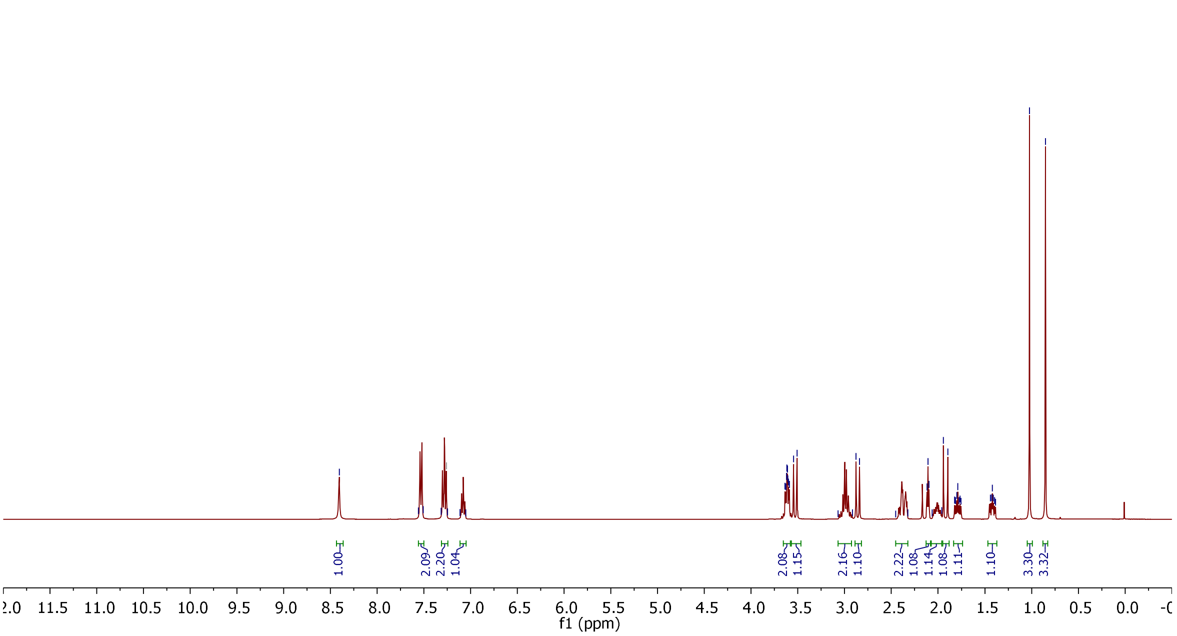

13C NMR (101 MHz, CDCl3)

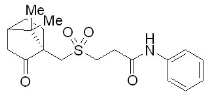

3o

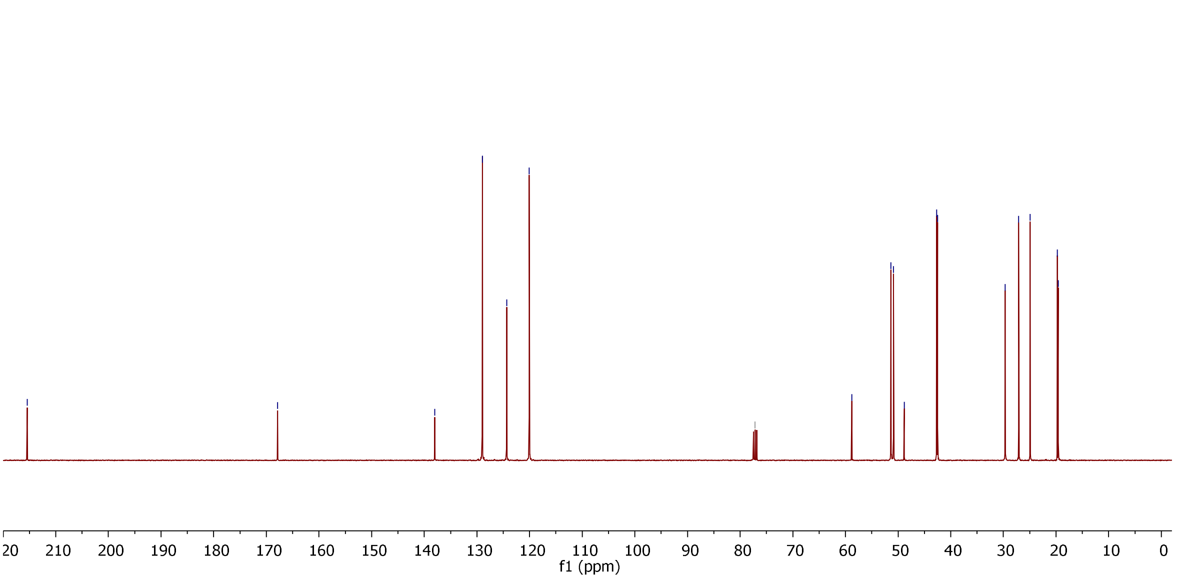

1H NMR (500 MHz, CDCl3)

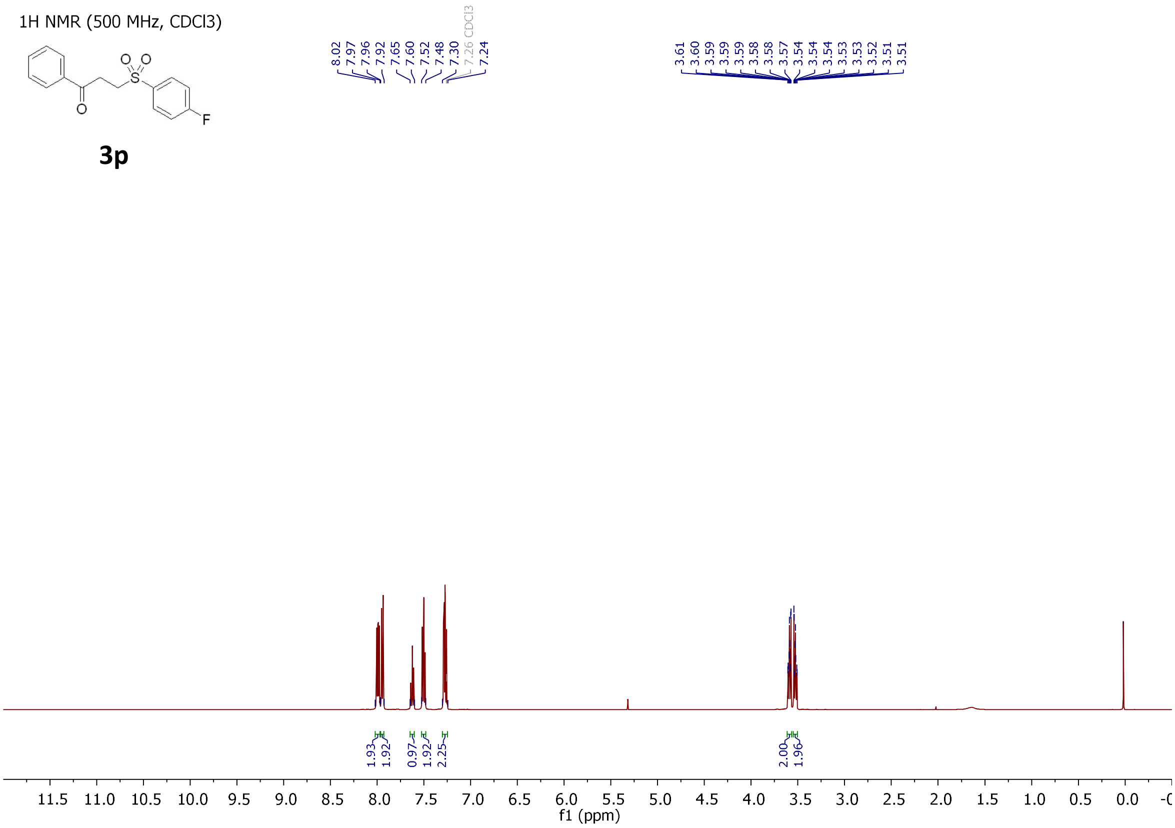

3p

13C NMR (126 MHz, CDCl3)

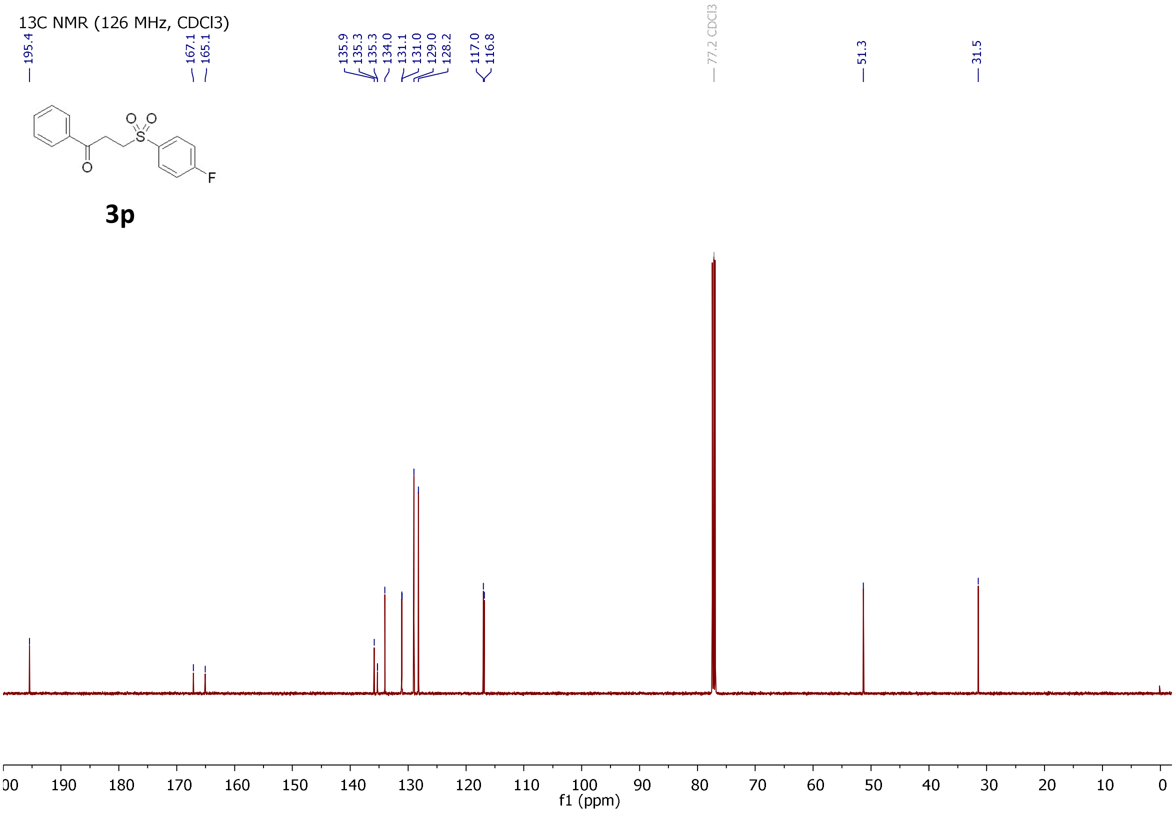

3p

19F NMR (471 MHz, CDCl3)

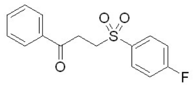

3p

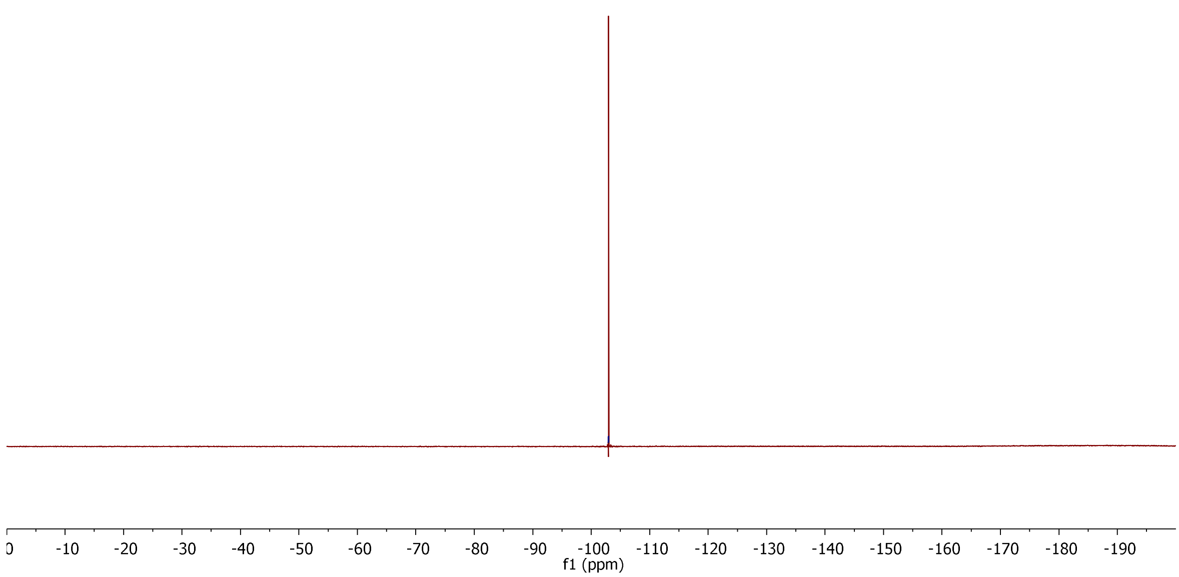

1H NMR (400 MHz, CDCl3)

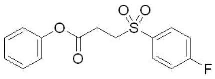

3q

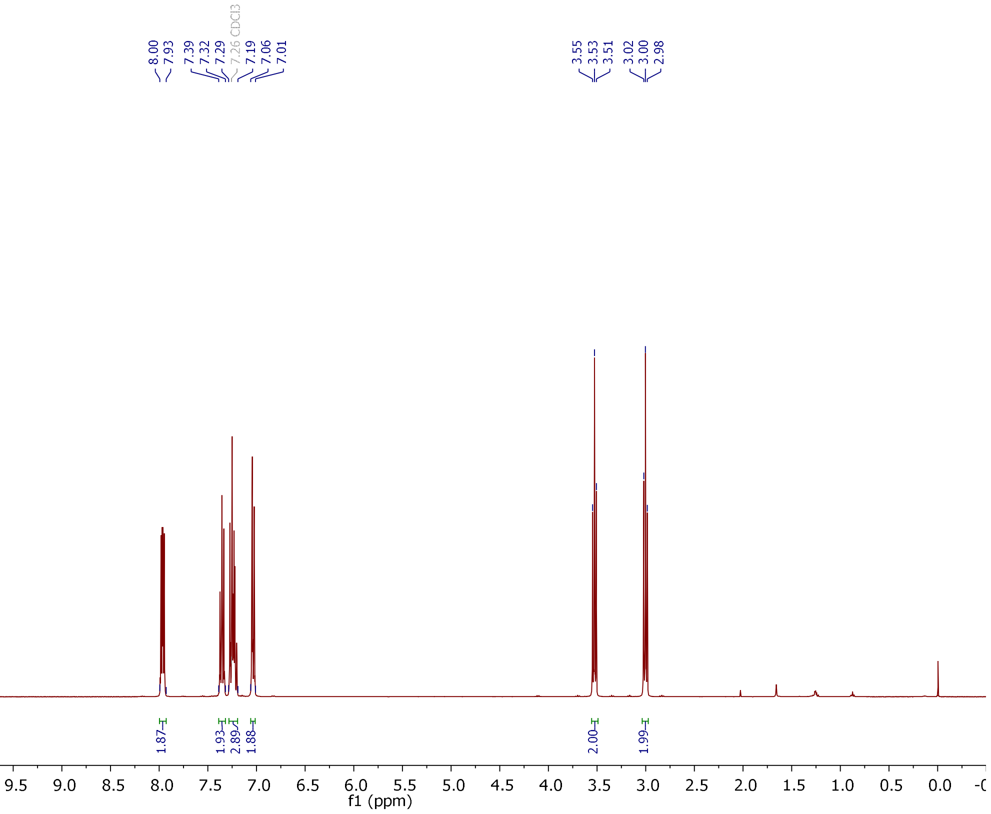

13C NMR (101 MHz, CDCl3)

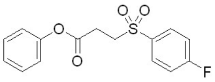

3q

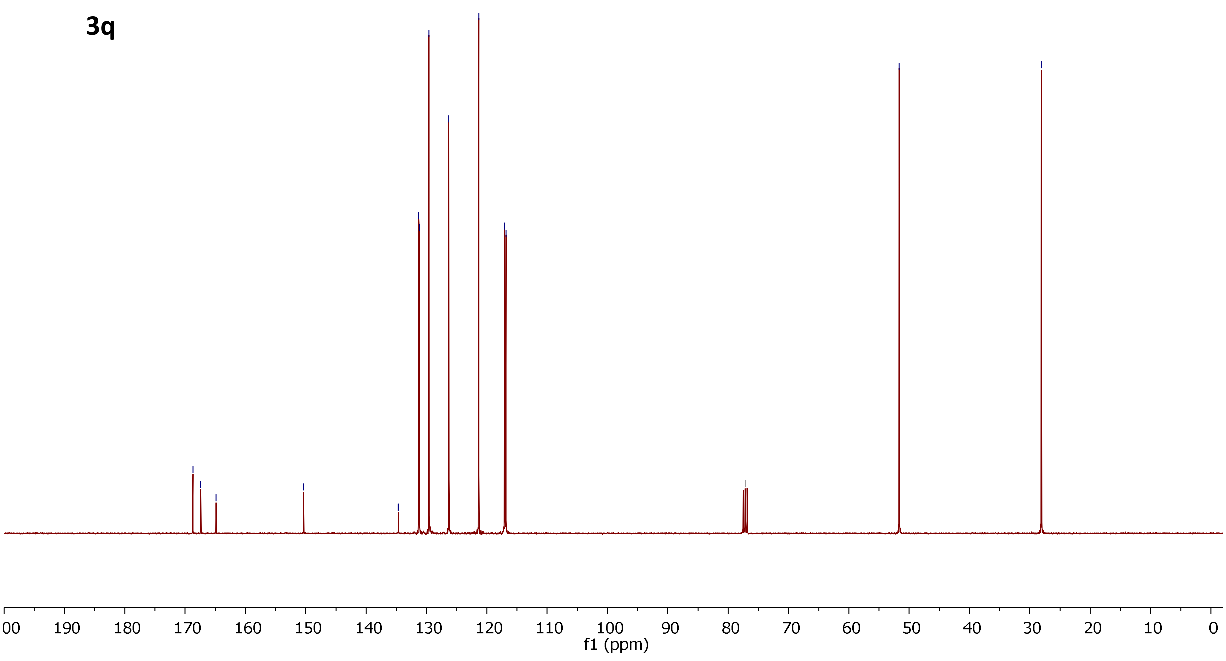

19F NMR (471 MHz, CDCl3)

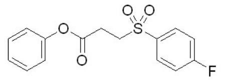

3q

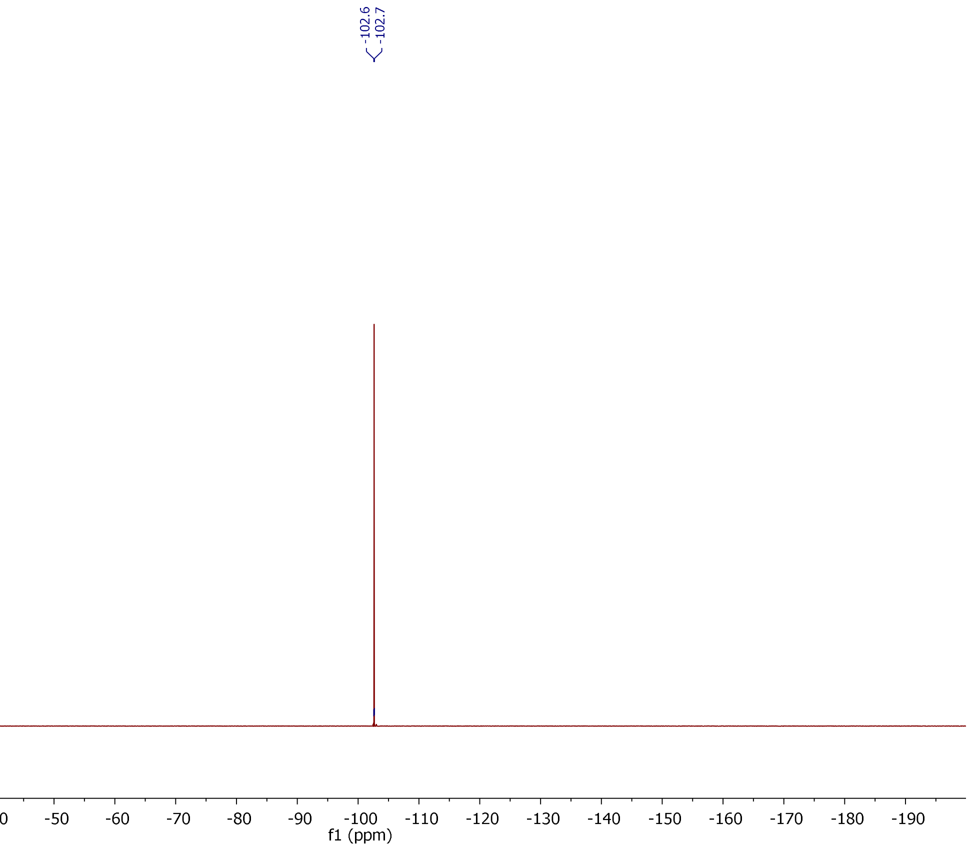

1H NMR (400 MHz, DMSO-d6)

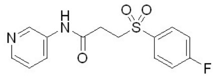

3r

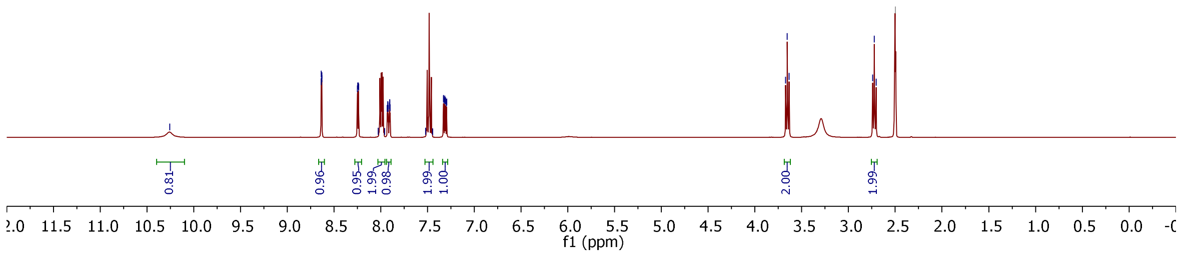

13C NMR (101 MHz, DMSO-d6)

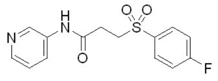

3r

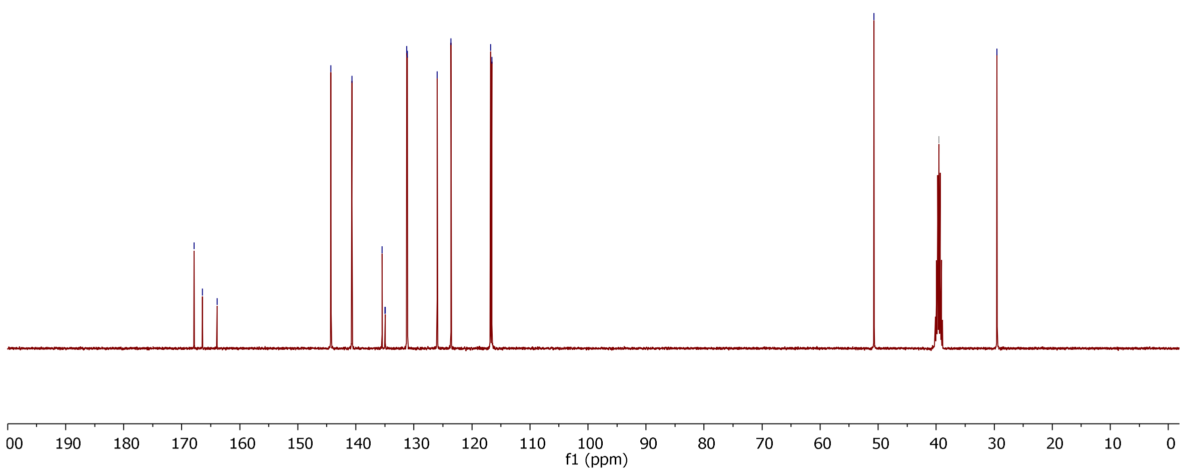

19F NMR (377 MHz, CDCl3)

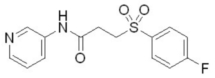

3r

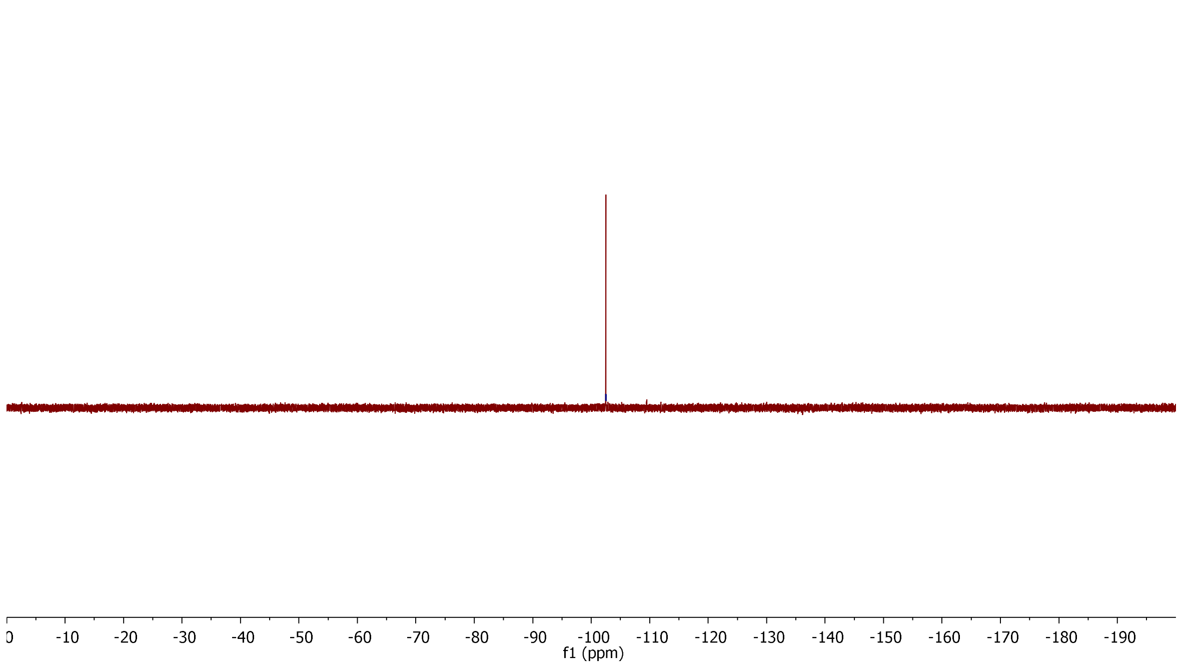

1H NMR (400 MHz, DMSO-d6)

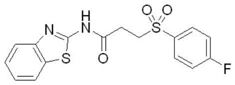

3s

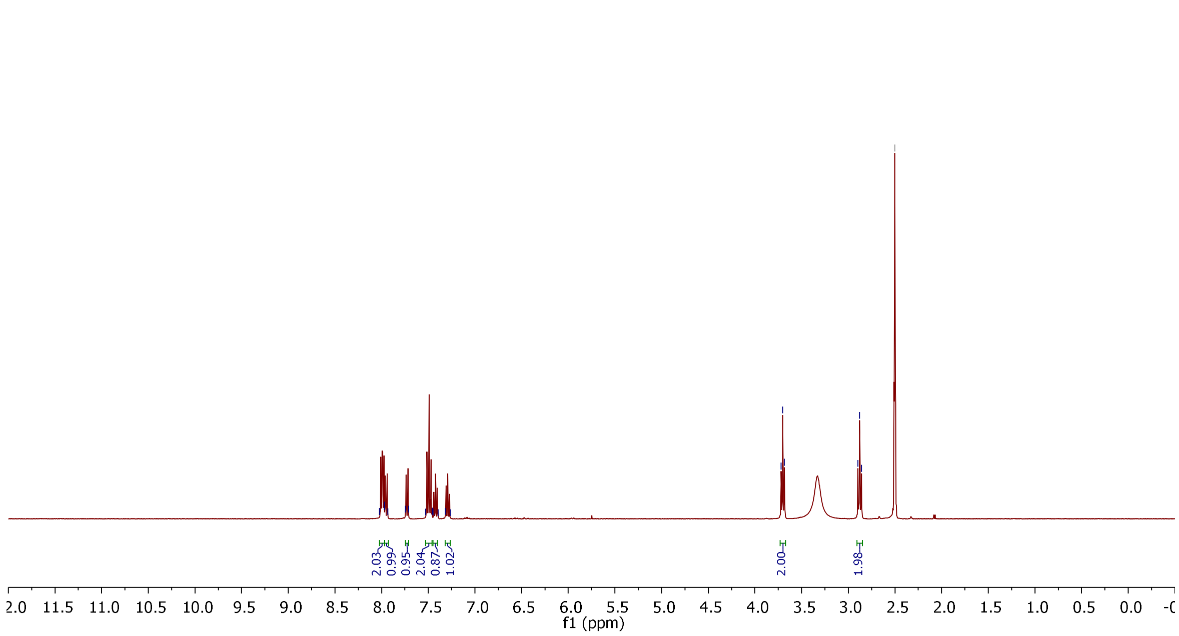

13C NMR (101 MHz, DMSO-d6)

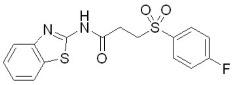

3s

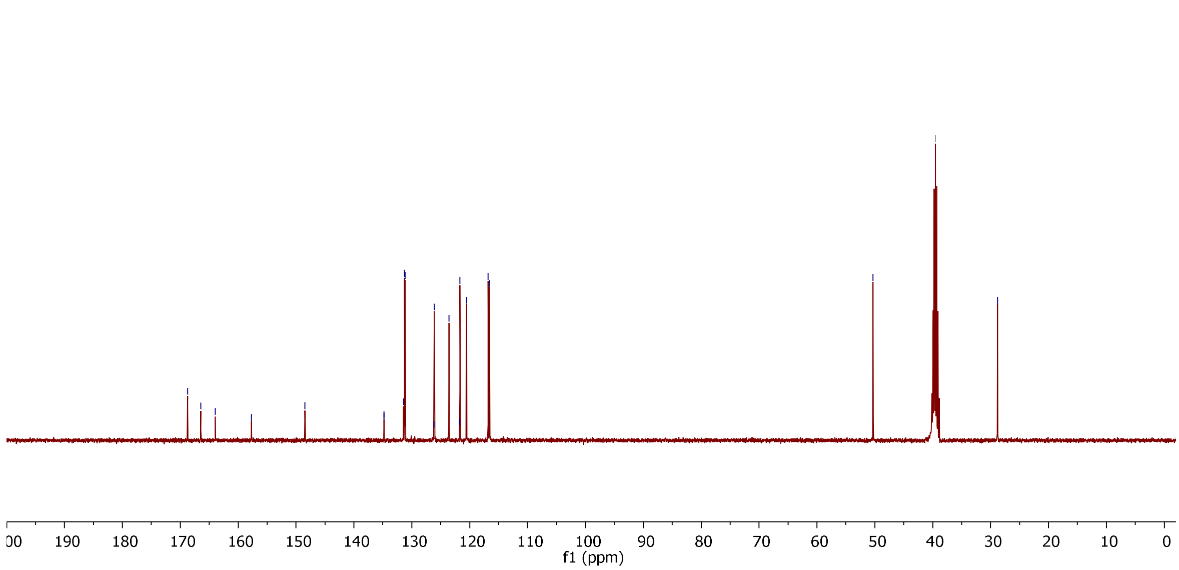

19F NMR (471 MHz, DMSO-d6)

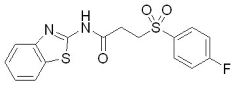

3s

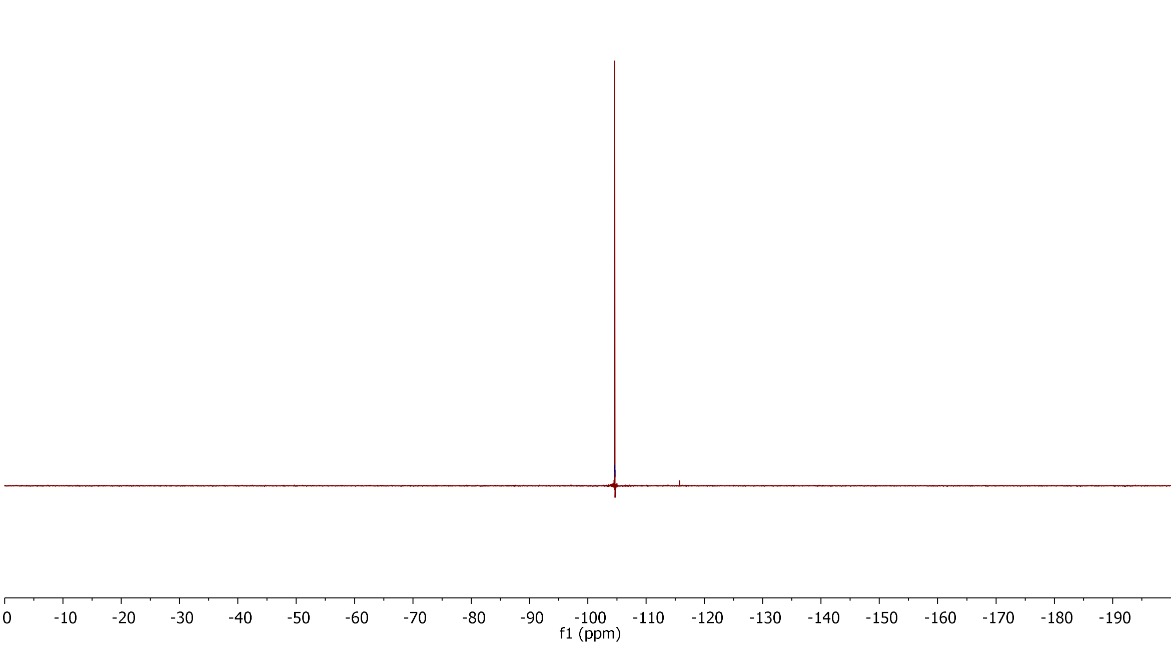

1H NMR (500 MHz, CDCl3)

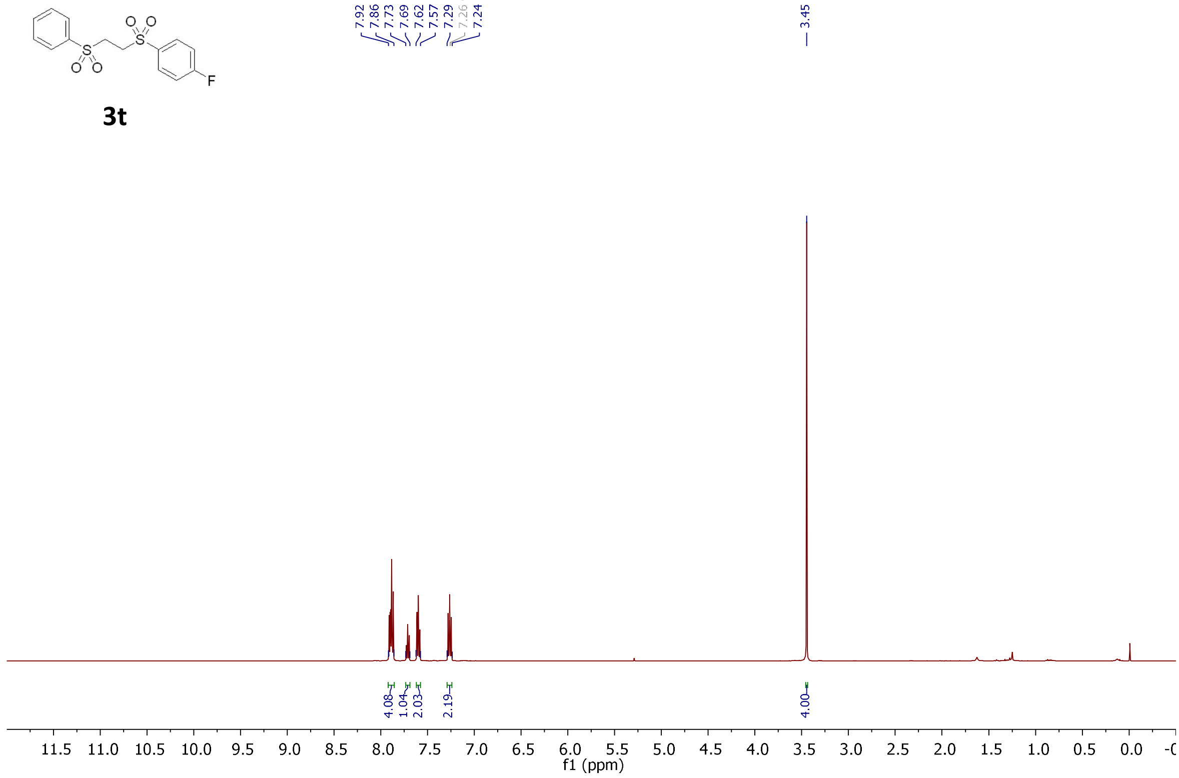

3t

13C NMR (126 MHz, CDCl3)

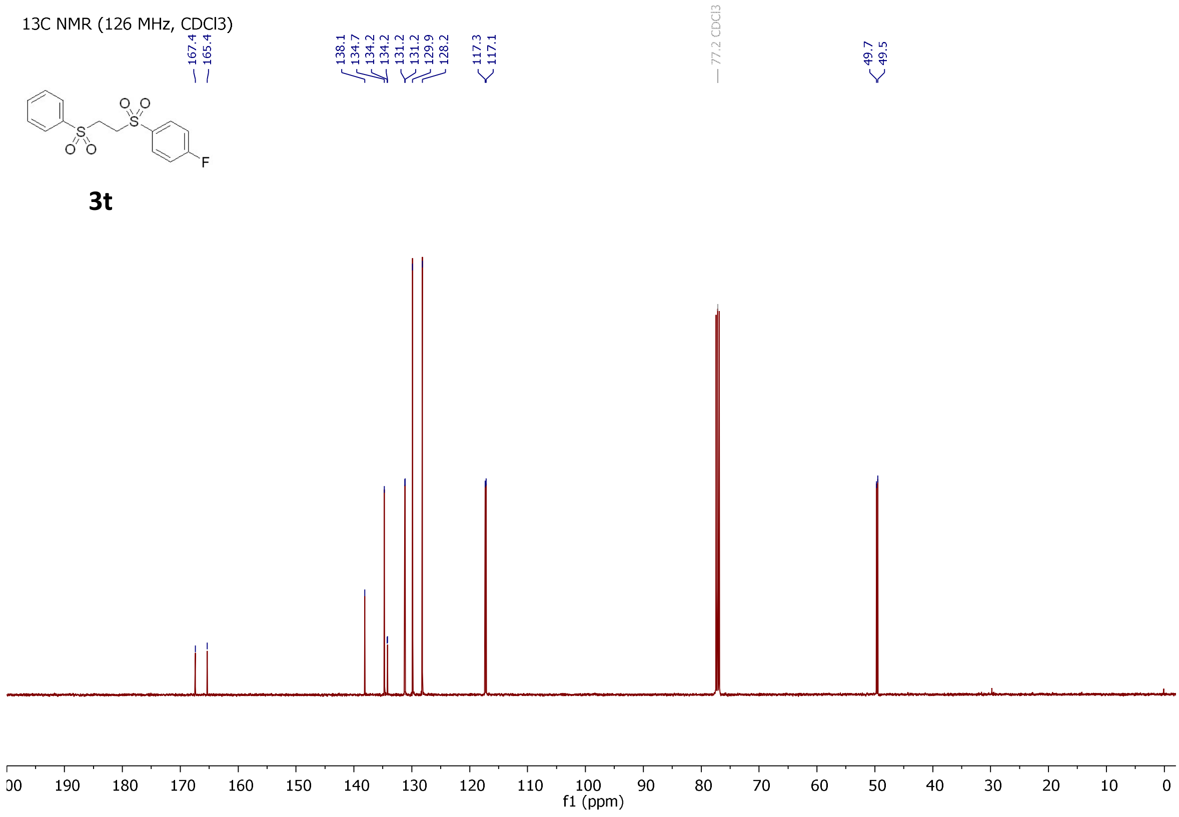

3t

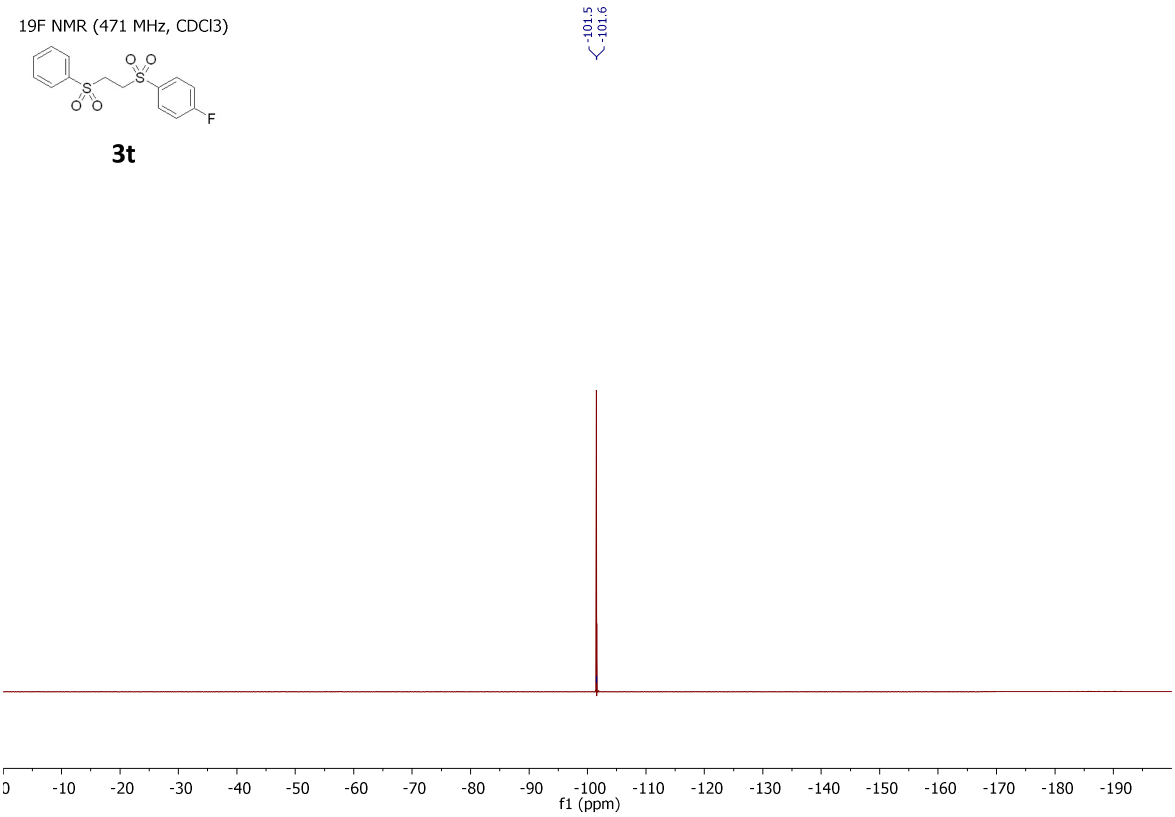
19F NMR (471 MHz, CDCl3)
3t
-101.5
-101.6
0
-10
-20
-30
-40
-50
-60
-70
-80
-90
-100
-110
-120
-130
-140
-150
-160
-170
-180
-190
f1 (ppm)

1H NMR (400 MHz, CDCl3)

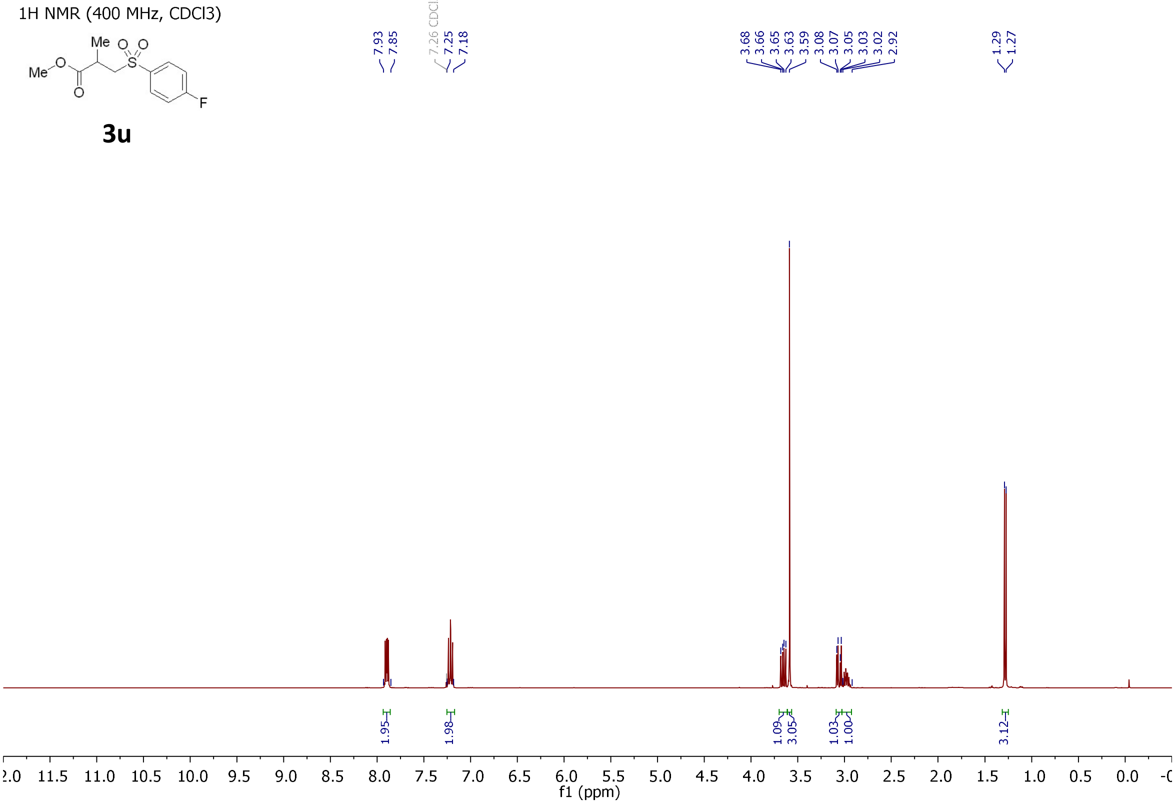

13C NMR (101 MHz, CDCl3)

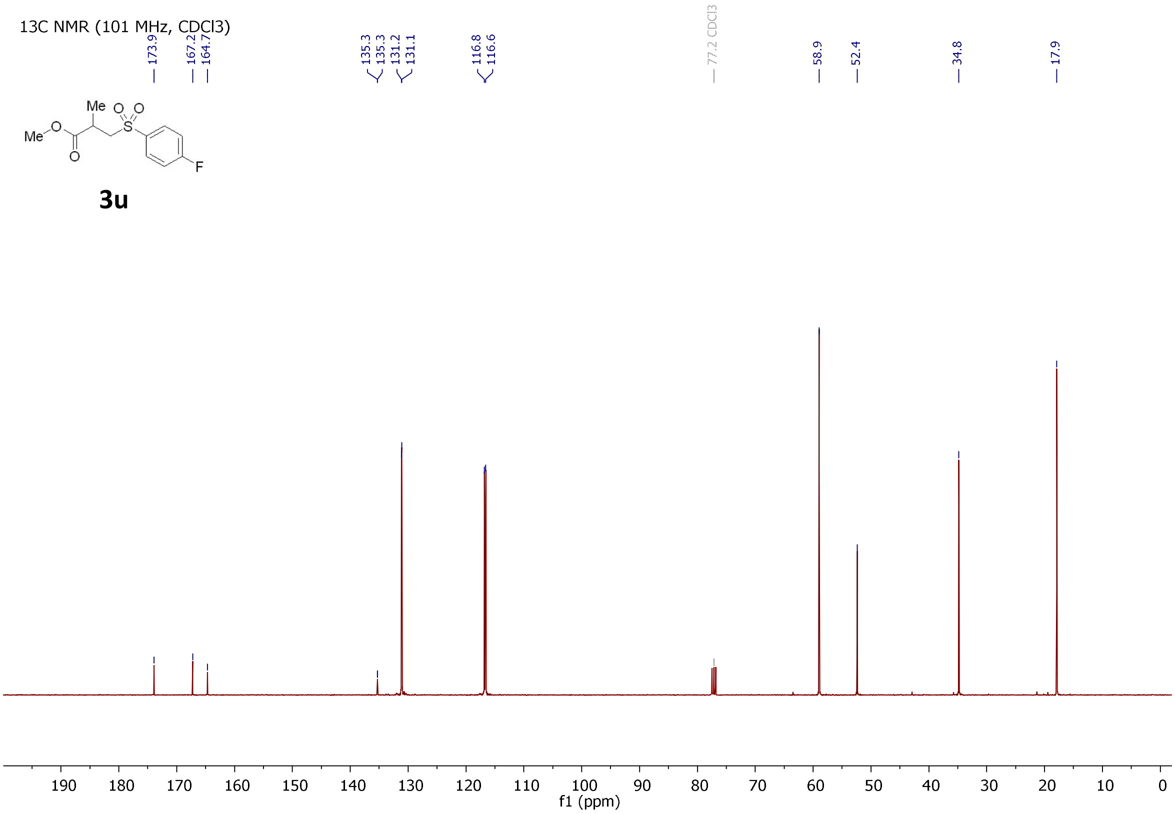

19F NMR (471 MHz, CDCl3)

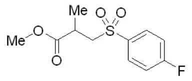

3u

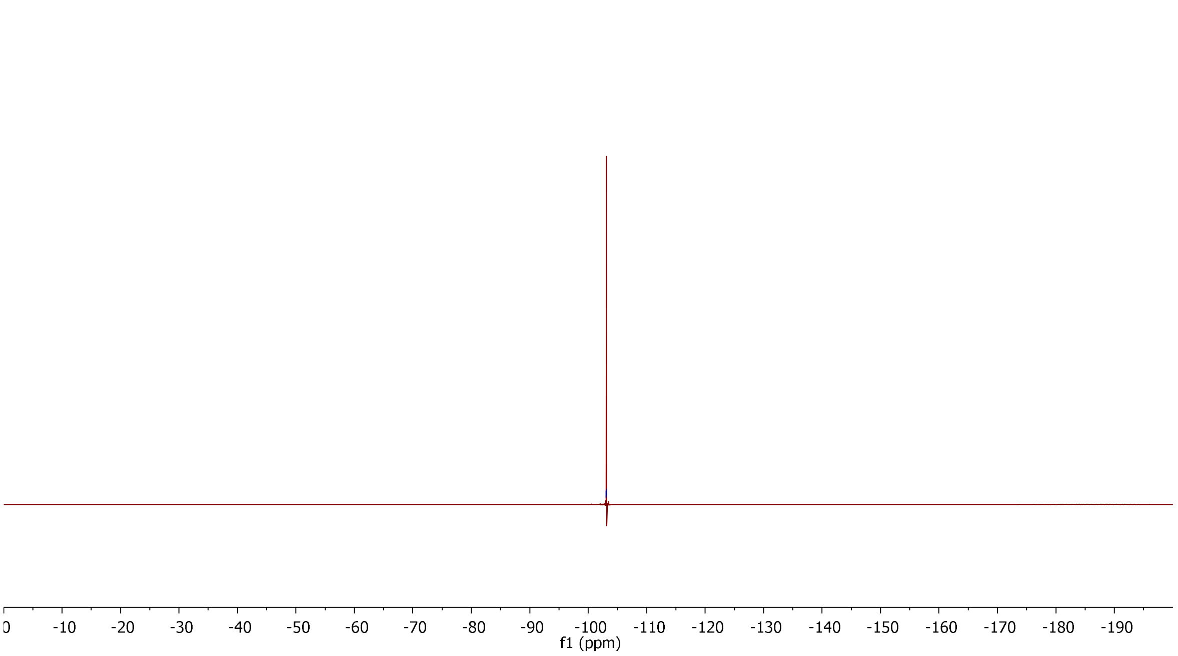

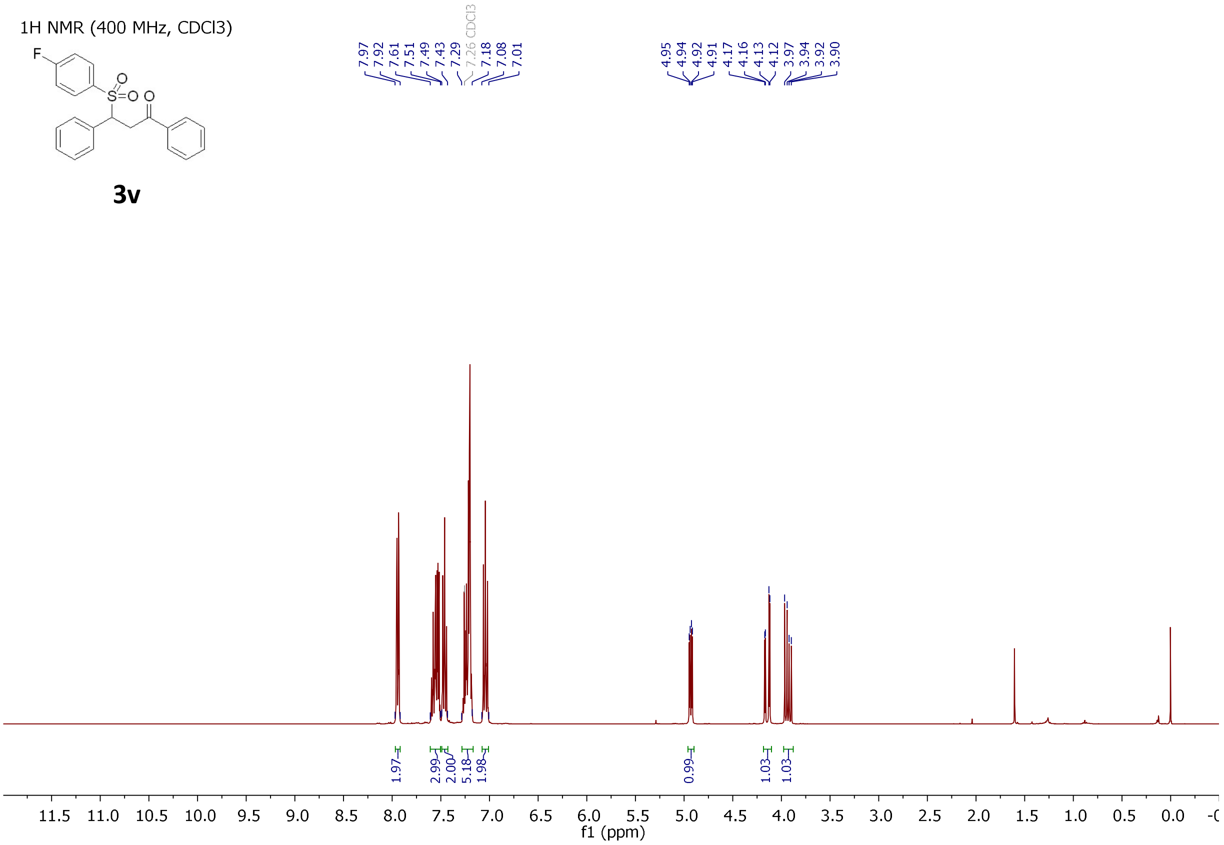
1H NMR (400 MHz, CDCl3)
F
O
S
O
O
3v
7.97
7.92
7.61
7.51
7.49
7.43
7.29
7.26 CDCl3
7.18
7.08
7.01
4.95
4.94
4.92
4.91
4.17
4.16
4.13
4.12
3.97
3.94
3.92
3.90
1.97
2.99
2.00
5.18
1.98
0.99
1.03
1.03
11.5 11.0 10.5 10.0 9.5 9.0 8.5 8.0 7.5 7.0 6.5 6.0 5.5 5.0 4.5 4.0 3.5 3.0 2.5 2.0 1.5 1.0 0.5 0.0
f1 (ppm)

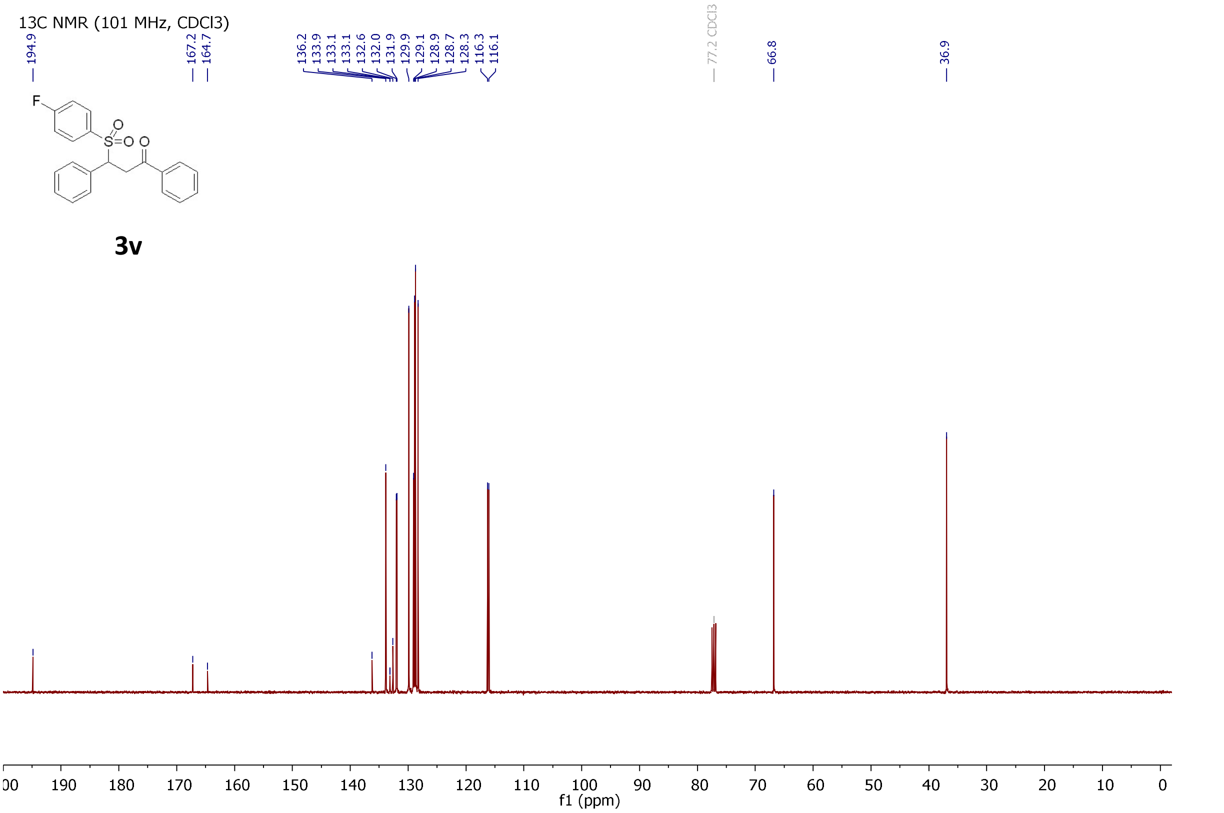
13C NMR (101 MHz, CDCl3)
194.9
167.2
164.7
136.2
133.9
133.1
132.6
132.0
131.9
129.9
129.1
128.9
128.7
128.3
116.3
116.1
77.2 CDCl3
66.8
36.9
F
O
S
O
O
3v
190 180 170 160 150 140 130 120 110 100 90 80 70 60 50 40 30 20 10 0
f1 (ppm)

19F NMR (471 MHz, CDCl3)

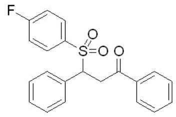

3v

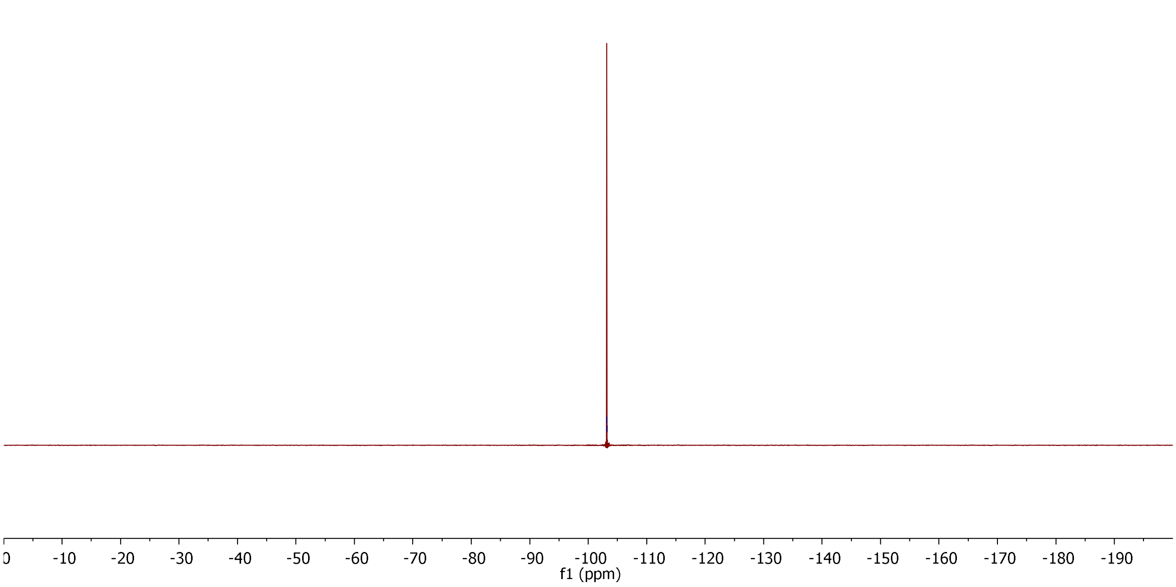

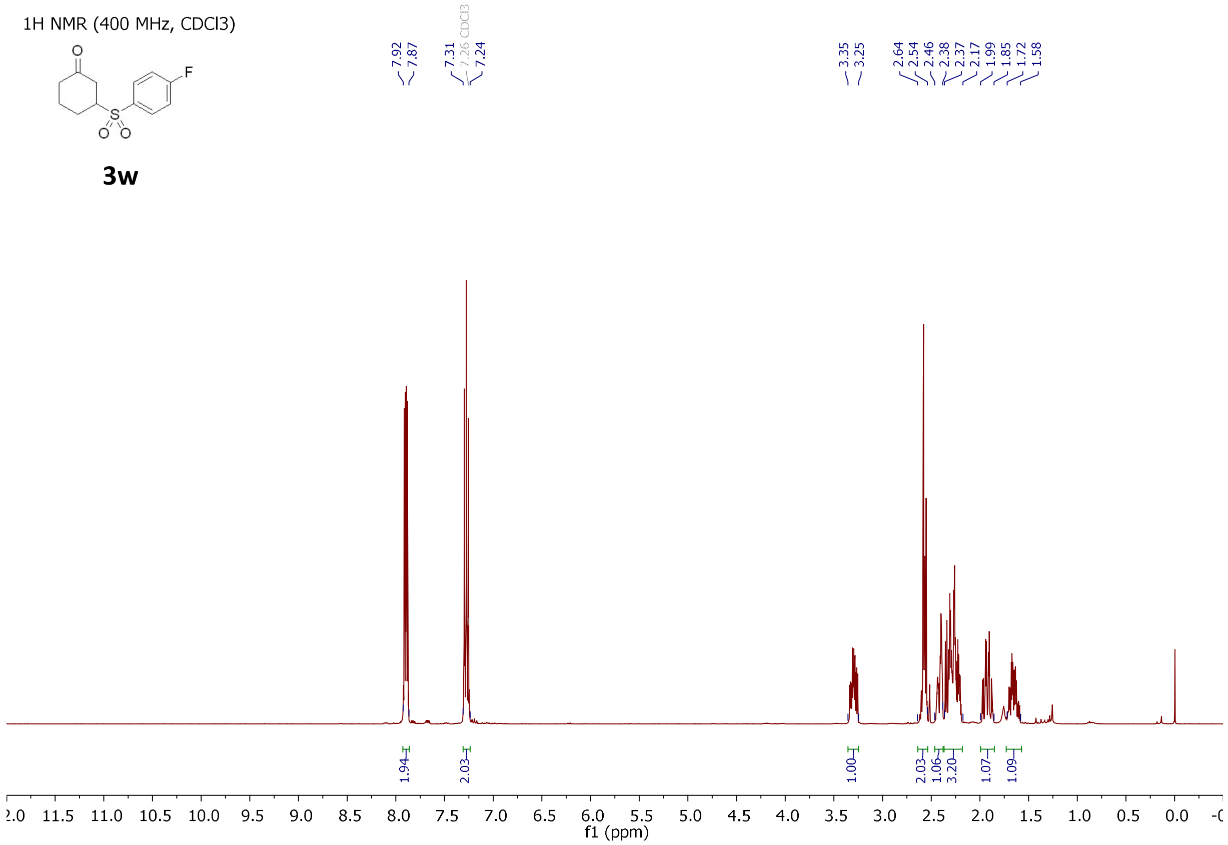
1H NMR (400 MHz, CDCl3)
3w
7.92
7.87
7.31
7.26 CDCl3
7.24
3.35
3.25
2.64
2.54
2.46
2.38
2.37
2.17
1.99
1.85
1.72
1.58
1.94
2.03
1.00
2.03
1.06
3.20
1.07
1.09
f1 (ppm)

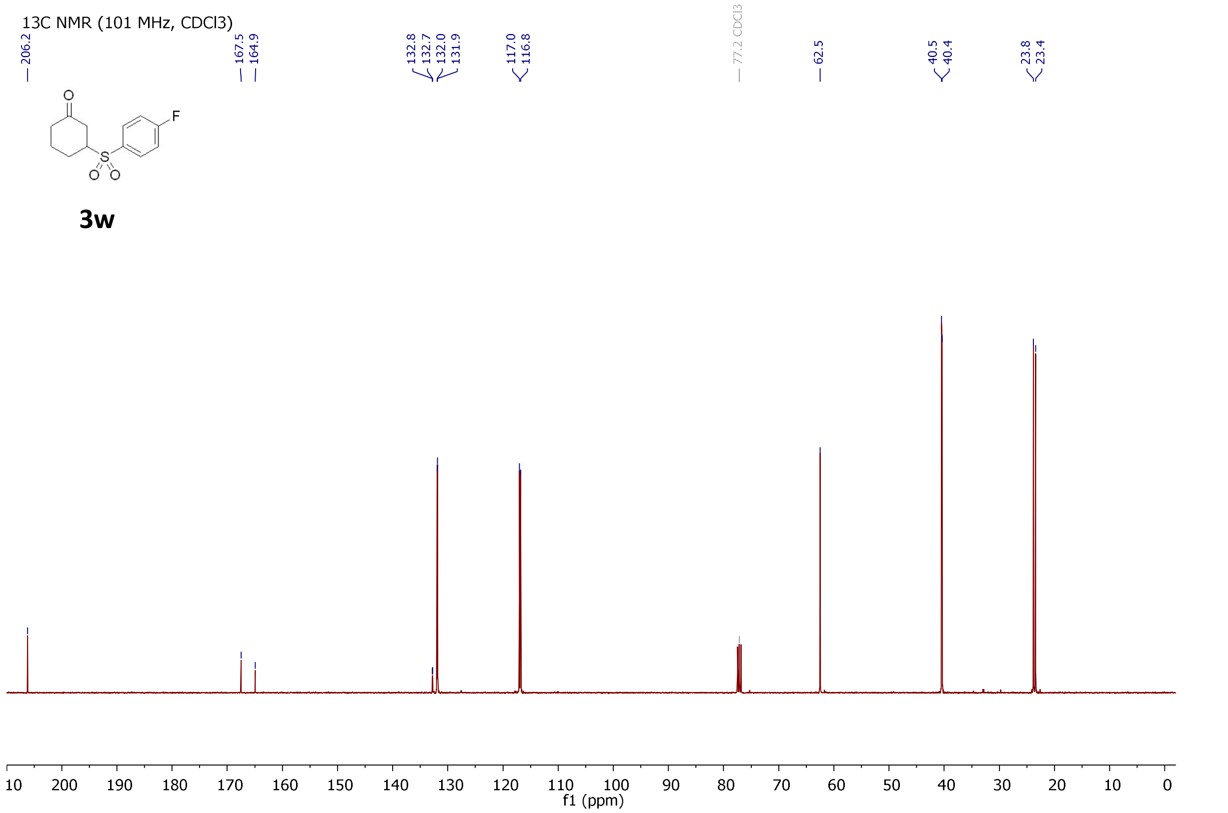
13C NMR (101 MHz, CDCl3)
3w
206.2
167.5
164.9
132.8
132.7
132.0
131.9
117.0
116.8
77.2 CDCl3
62.5
40.5
40.4
23.8
23.4
f1 (ppm)

19F NMR (471 MHz, CDCl3)

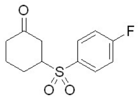

3w

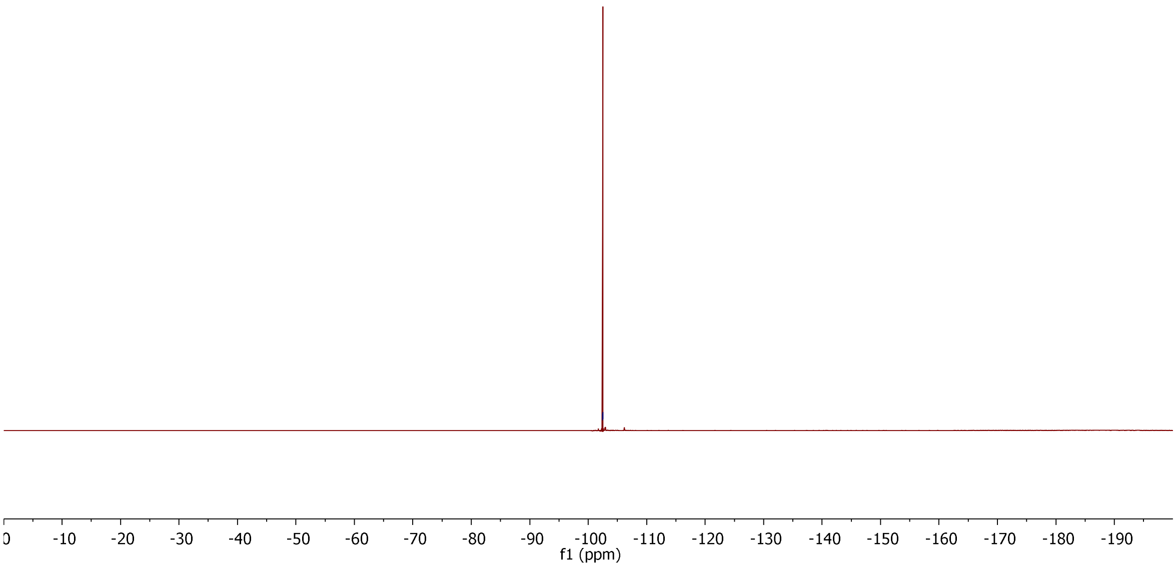

1H NMR (400 MHz, CDCl3)

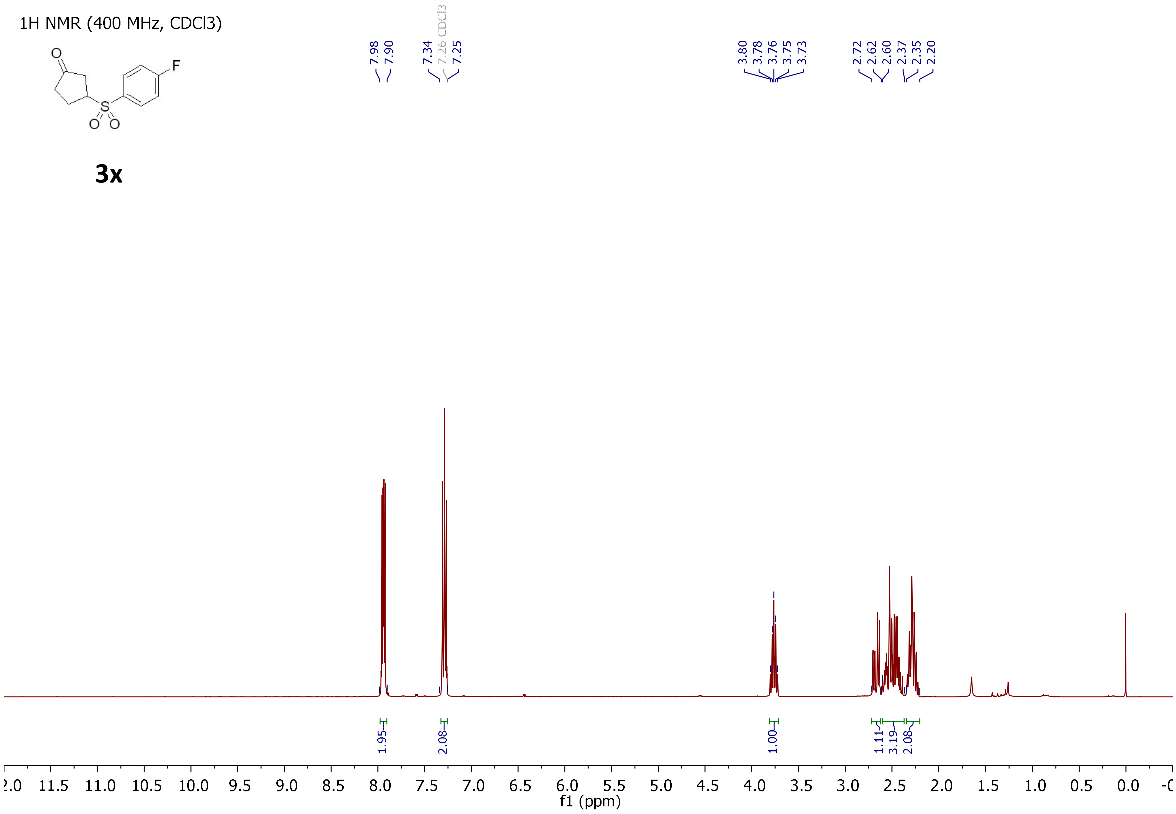

13C NMR (101 MHz, CDCl3)

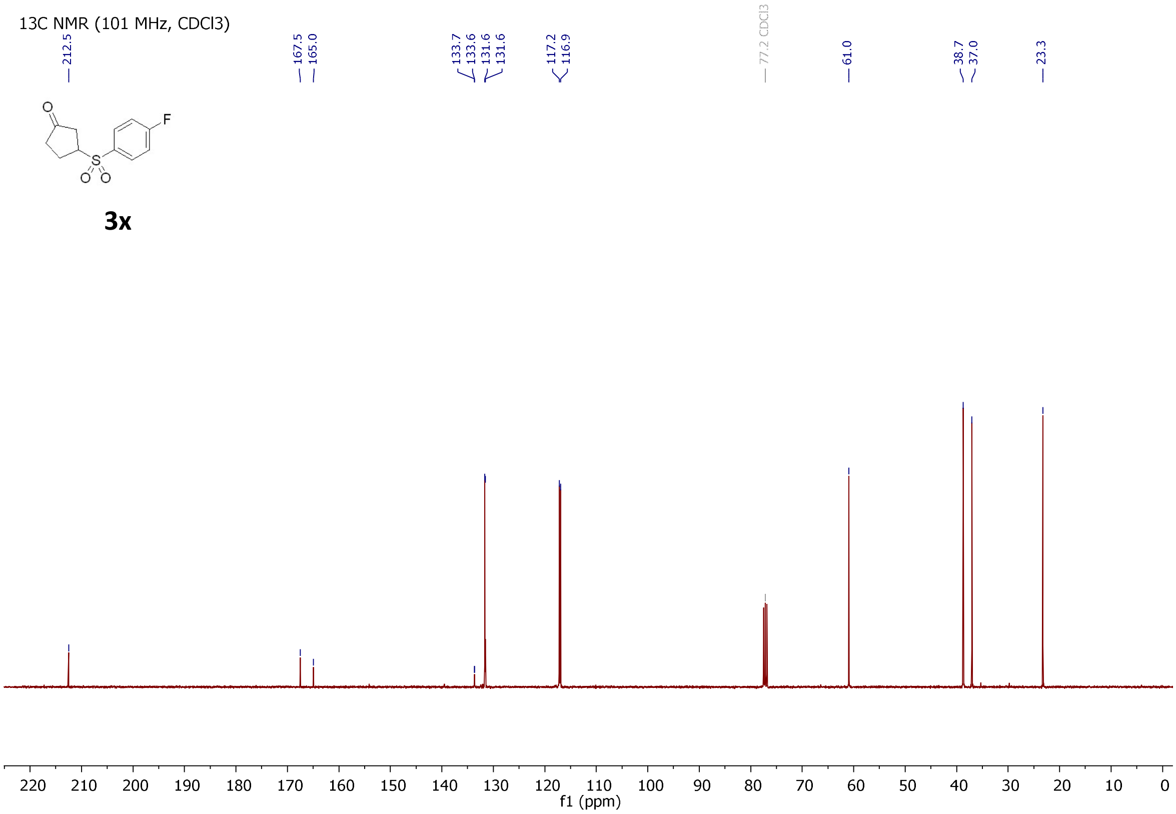

19F NMR (471 MHz, CDCl3)

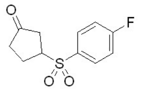

3x

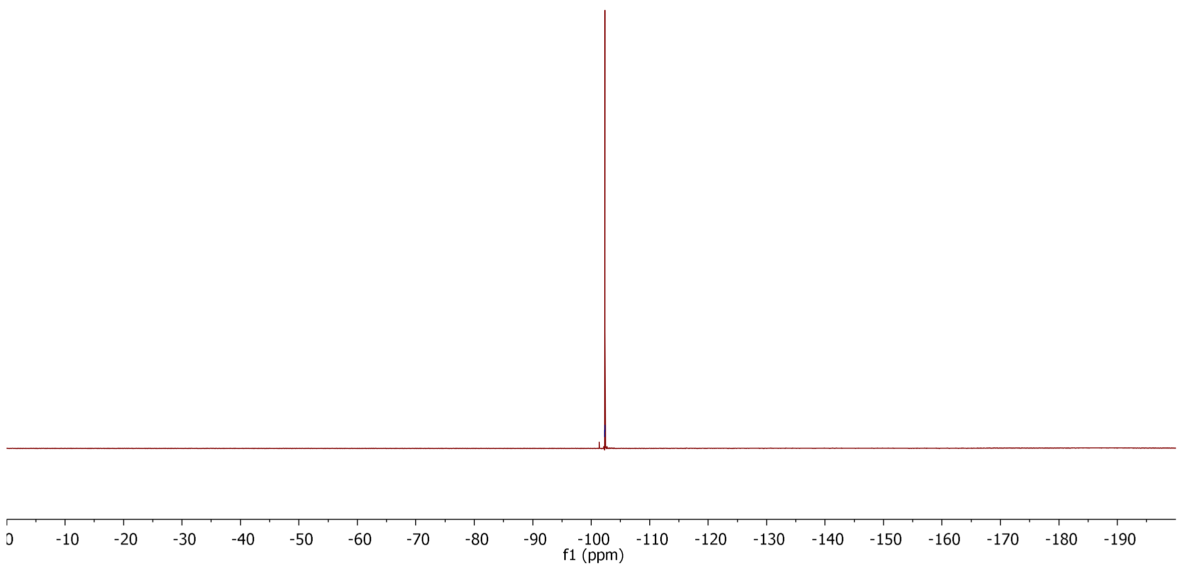

1H NMR (500 MHz, CDCl3)

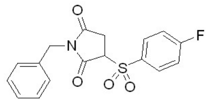

3y

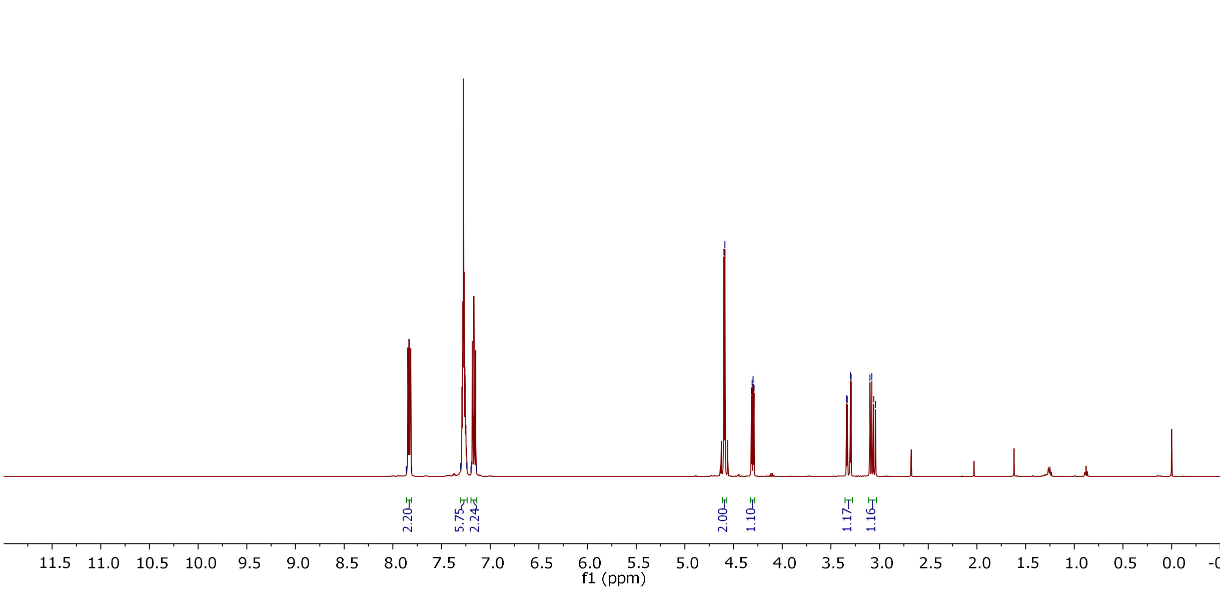

13C NMR (101 MHz, CDCl3)

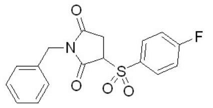

3y

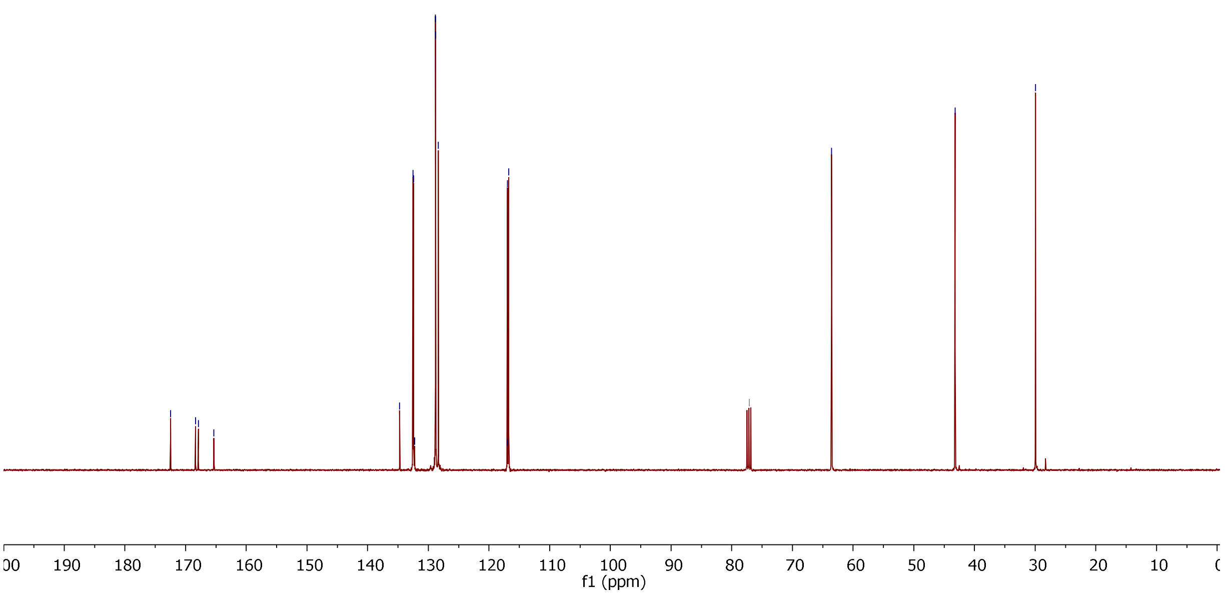

19F NMR (471 MHz, CDCl3)

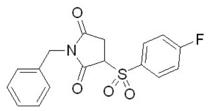

3y

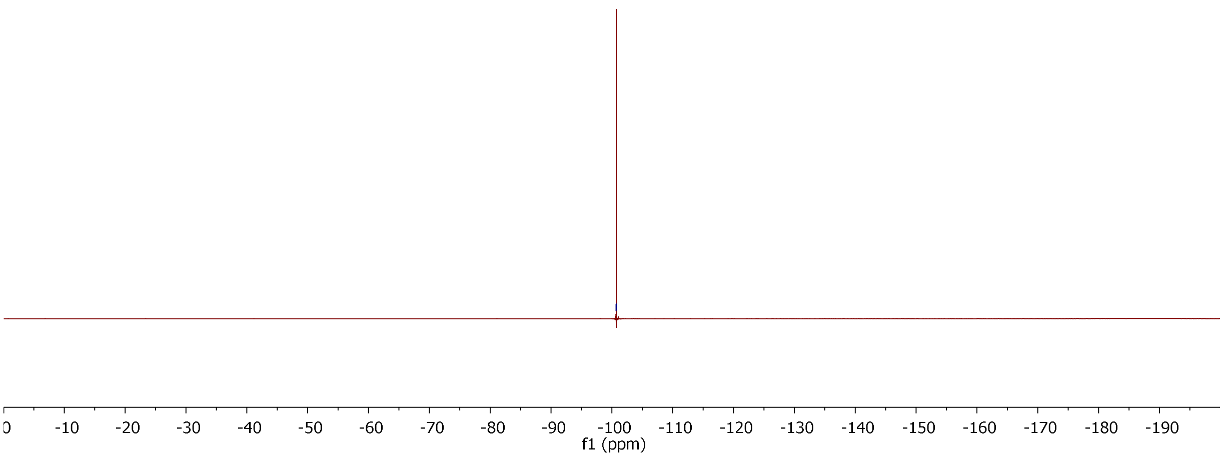

1H NMR (500 MHz, DMSO-d6)

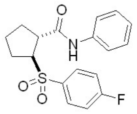

***trans*-3z**

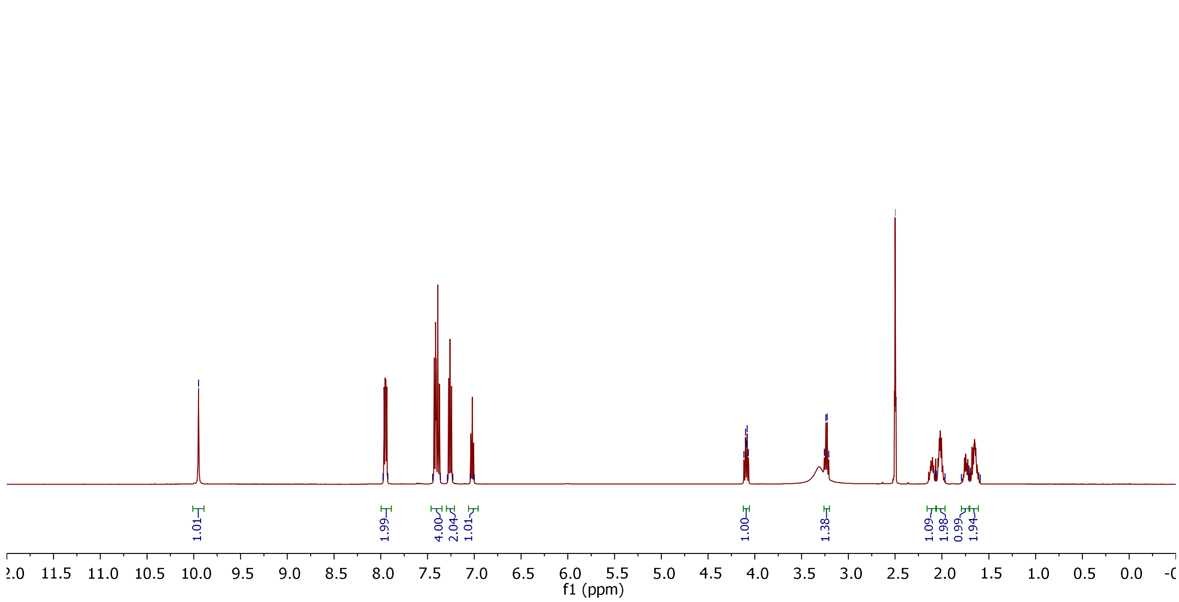

13C NMR (126 MHz, DMSO-d6)

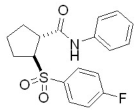

***trans*-3z**

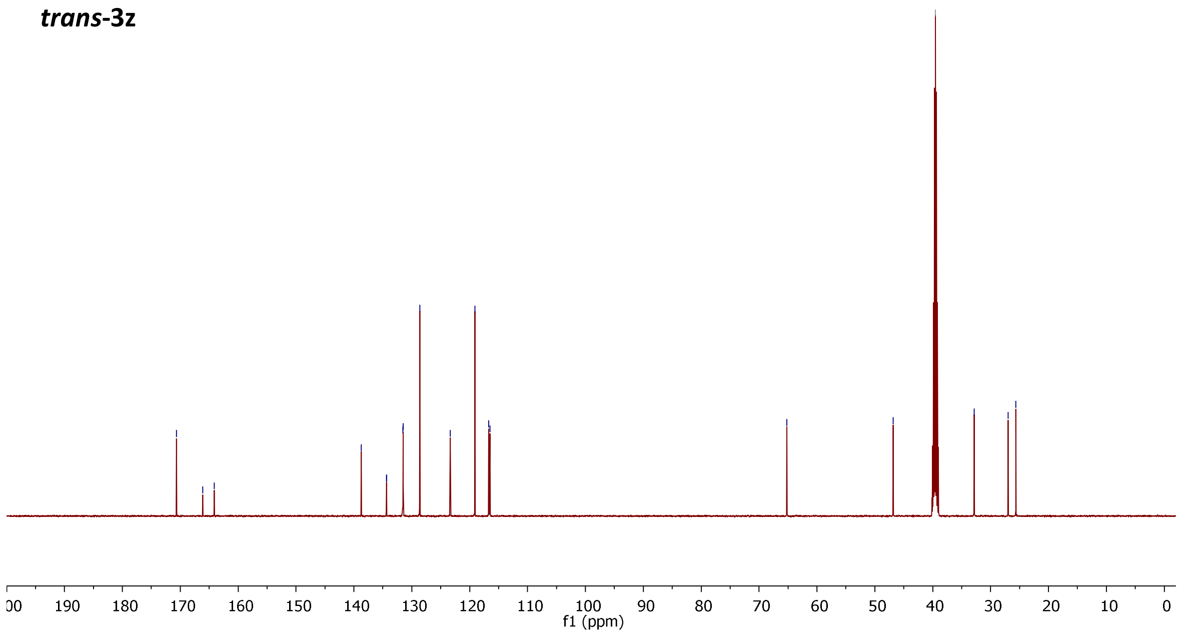

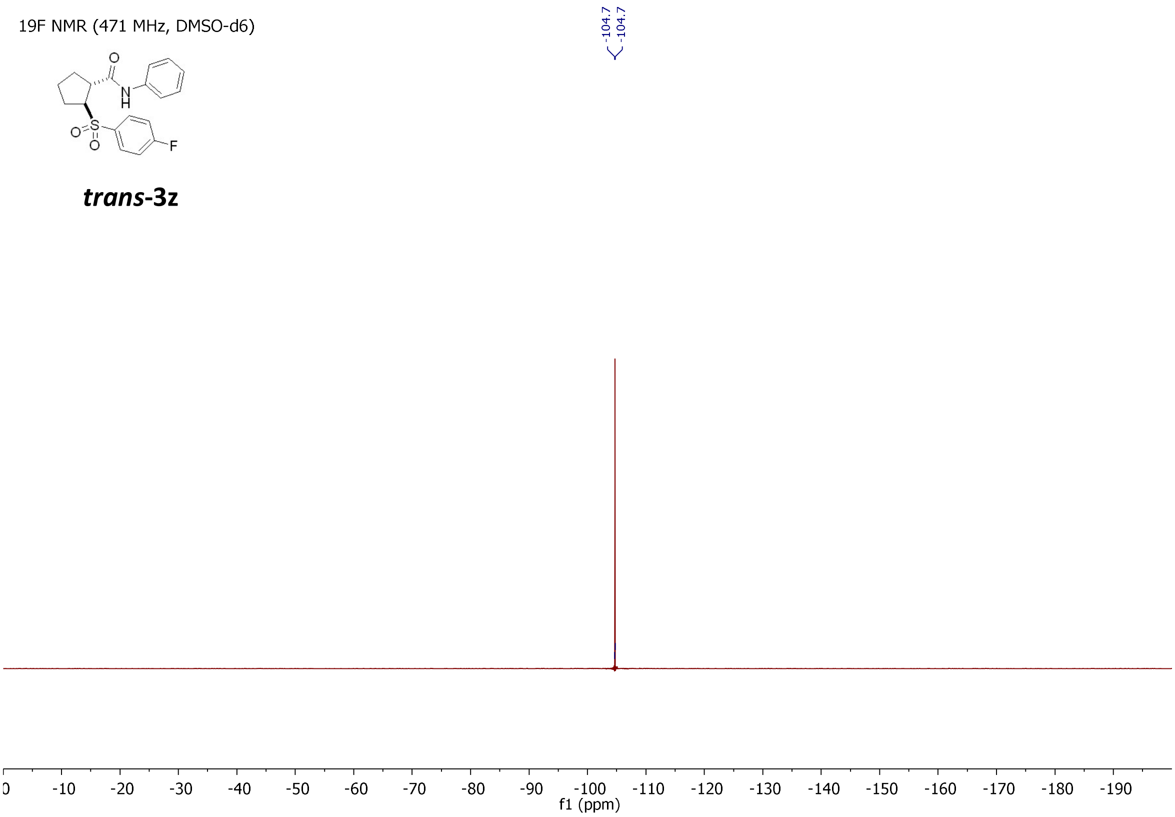
19F NMR (471 MHz, DMSO-d6)
trans-3z
-104.7
-104.7
f1 (ppm)

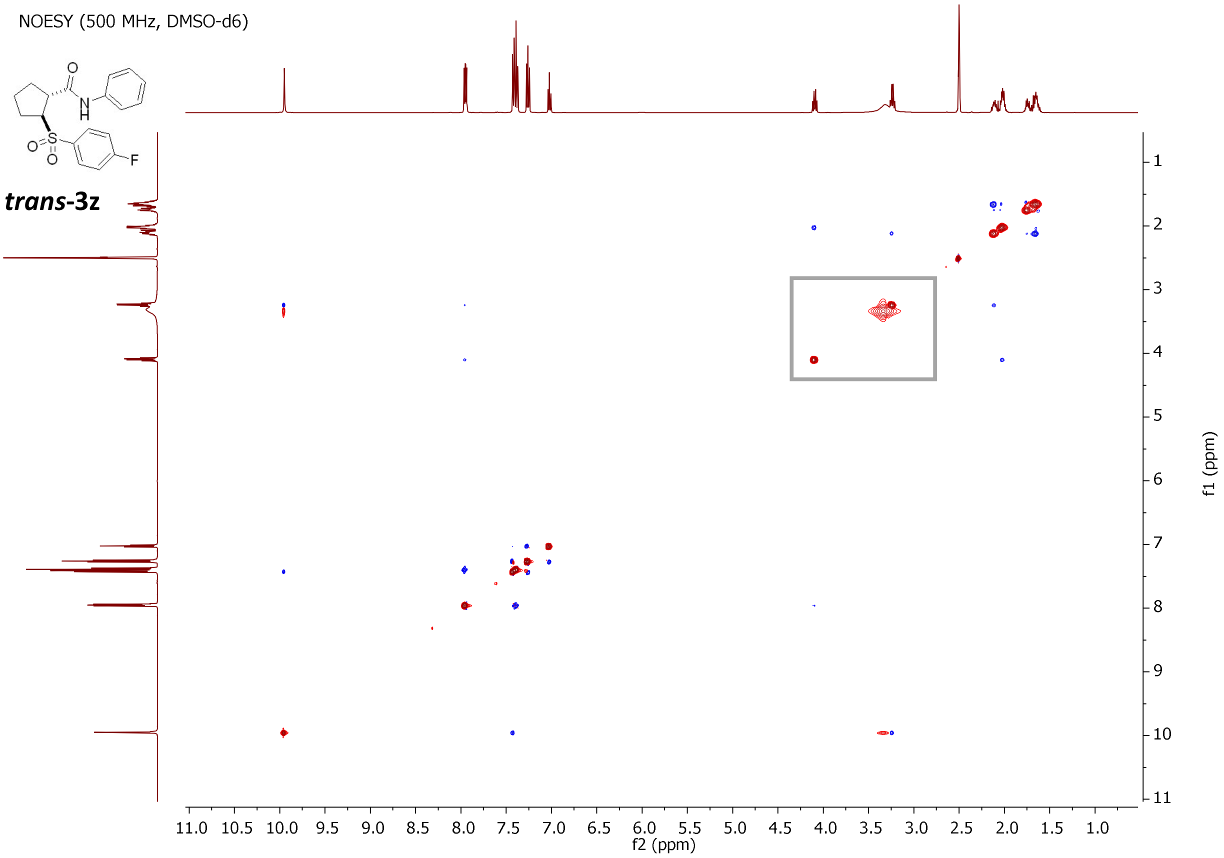
NOESY (500 MHz, DMSO-d6)
trans-3z
f2 (ppm)
f1 (ppm)

1H NMR (400 MHz, DMSO-d6)

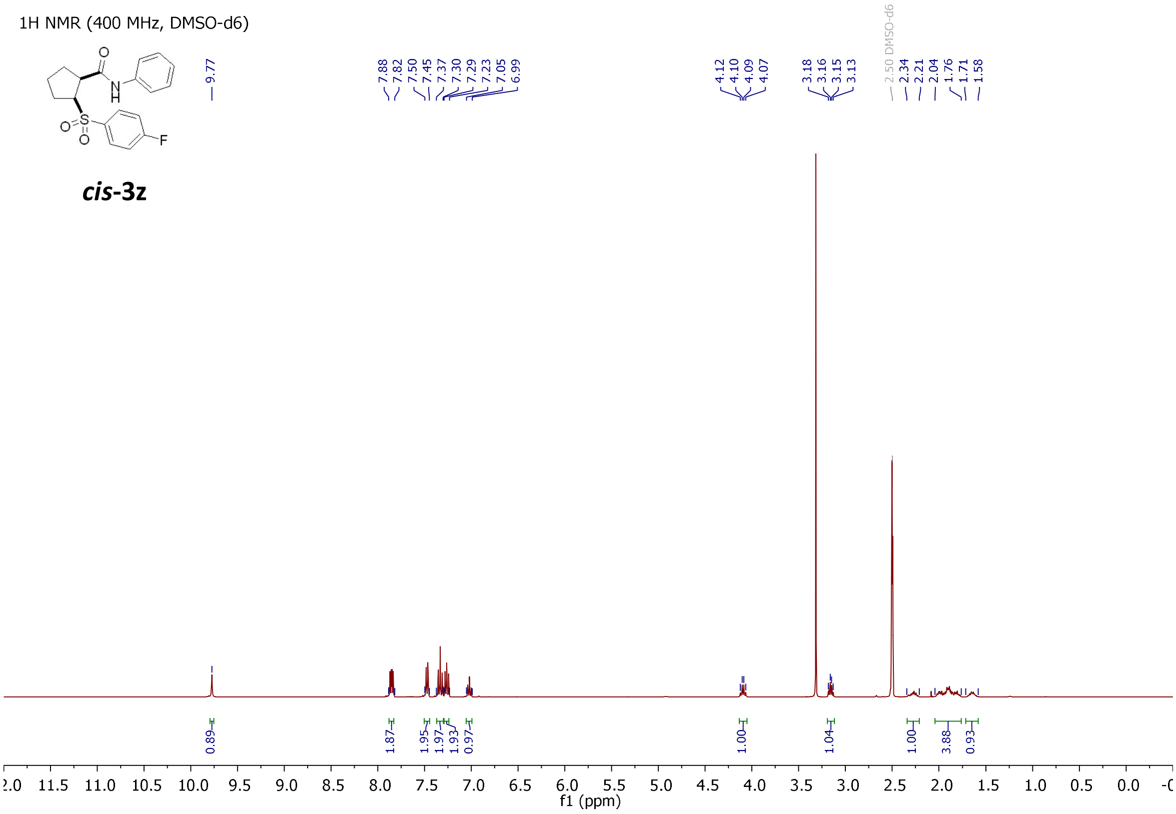

13C NMR (101 MHz, DMSO-d6)

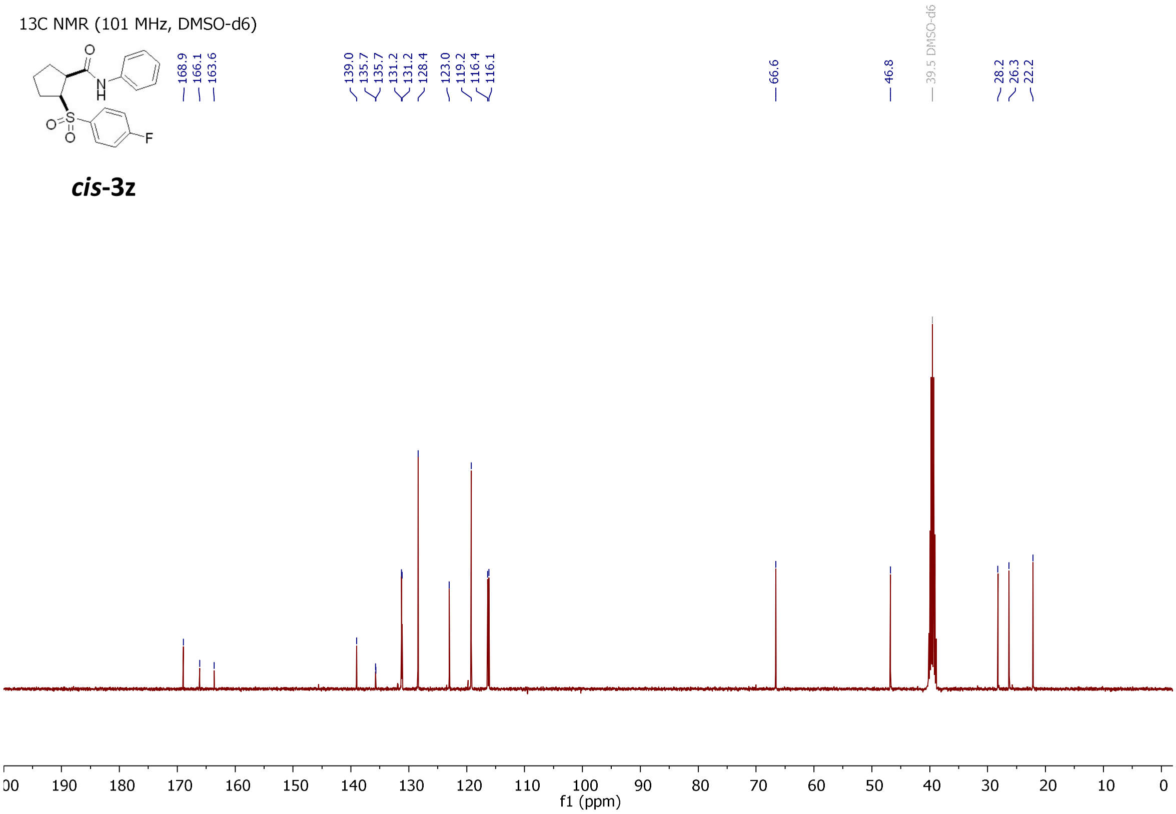

19F NMR (471 MHz, DMSO-d6)

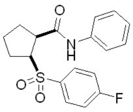

cis-3z

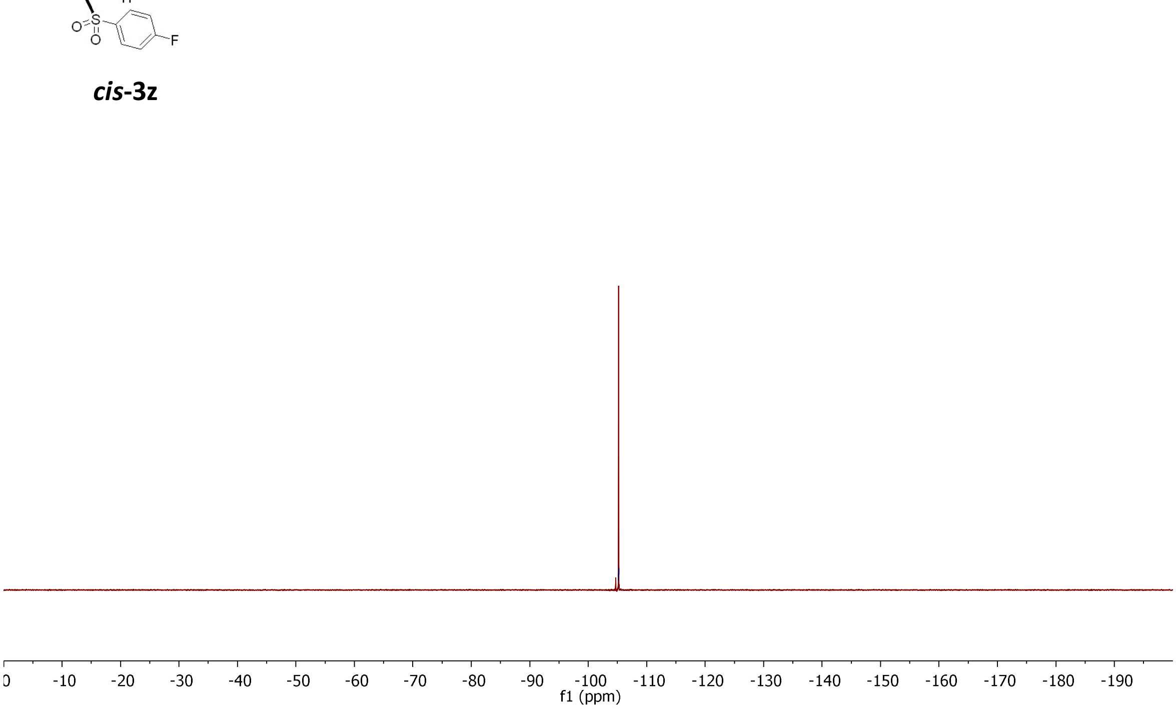

1H NMR (400 MHz, DMSO)

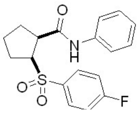

cis-3z

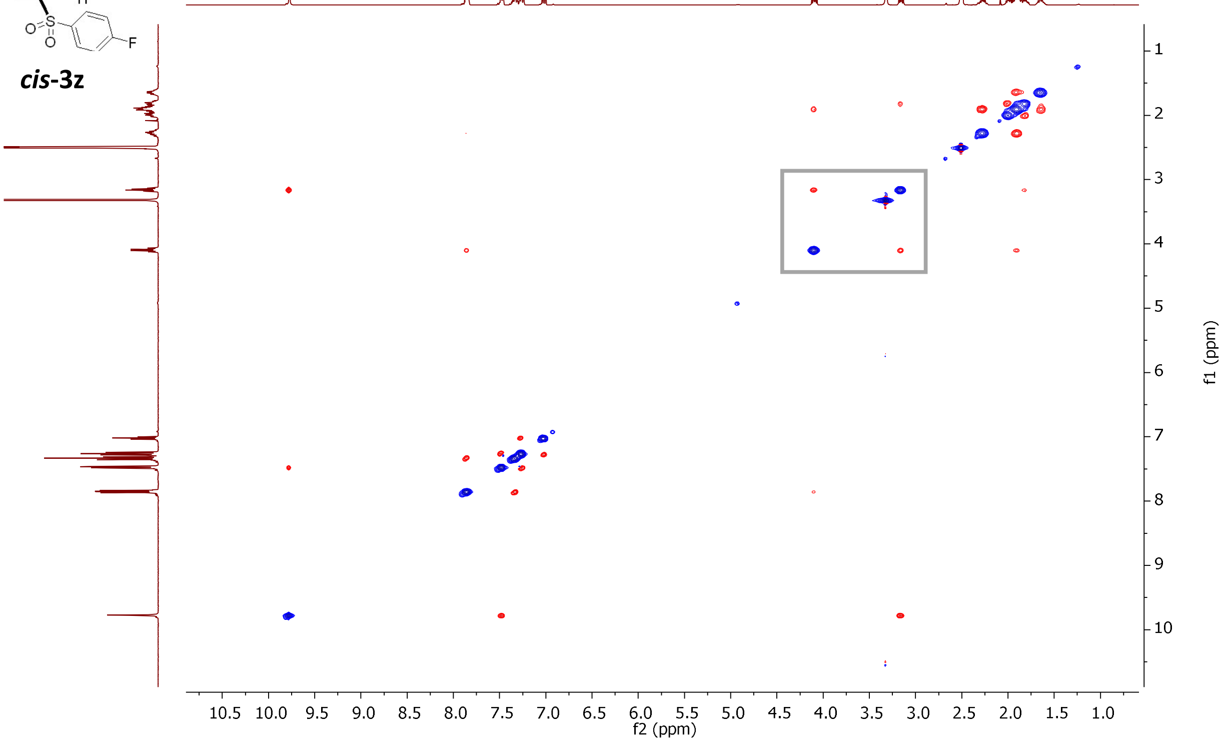

1H NMR (500 MHz, DMSO-d6)

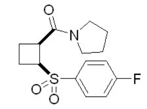

3aa

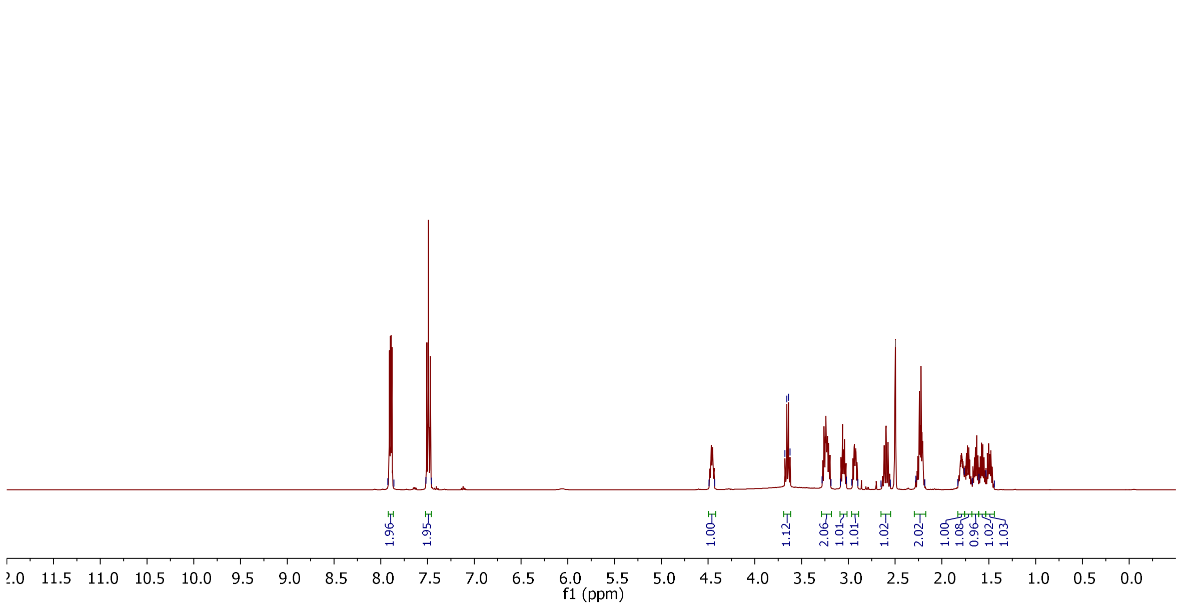

13C NMR (126 MHz, DMSO-d6)

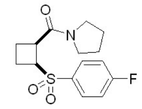

3aa

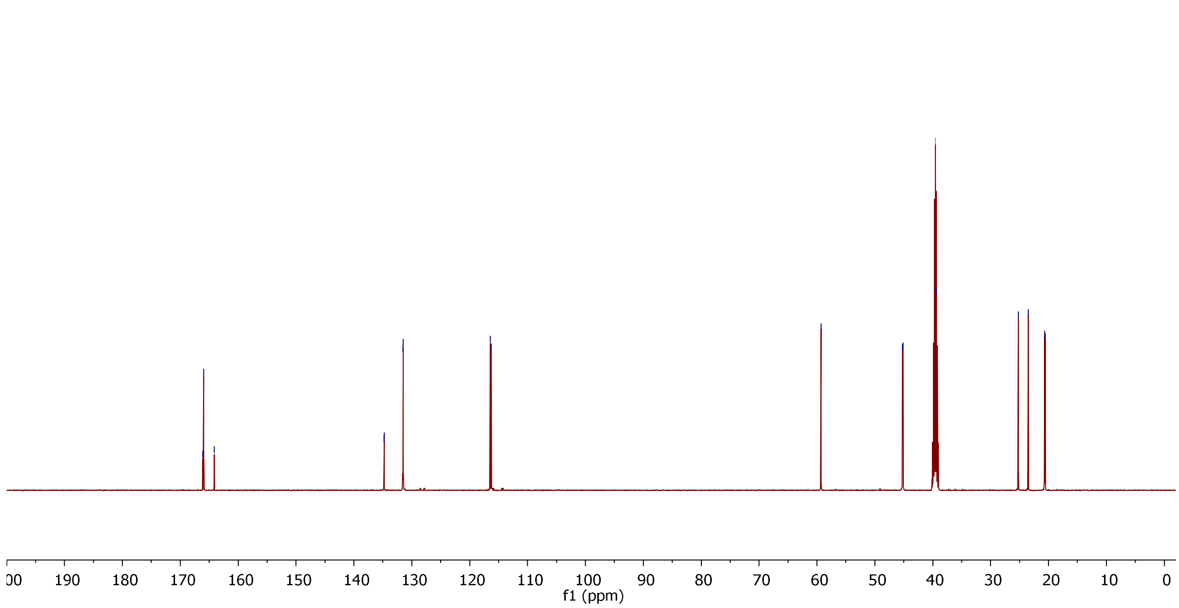

19F NMR (471 MHz, DMSO-d6)

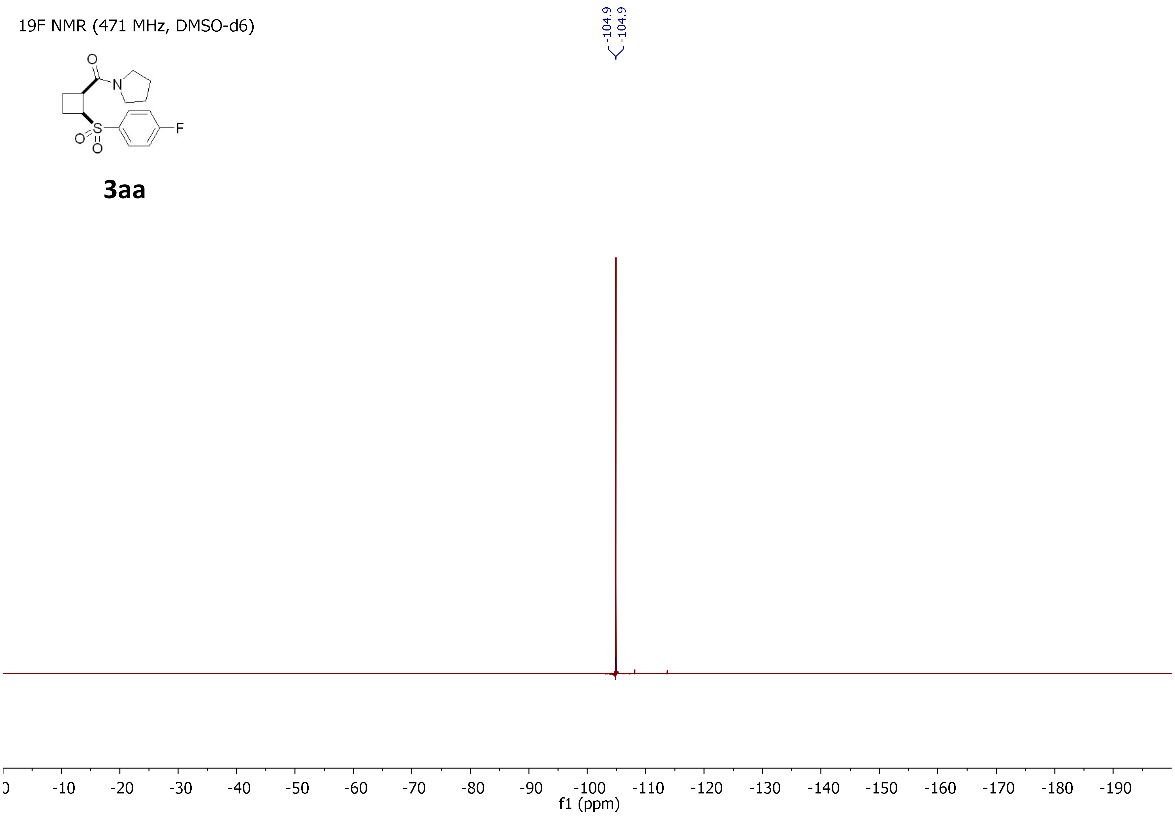

NOESY (500 MHz, DMSO-d6)

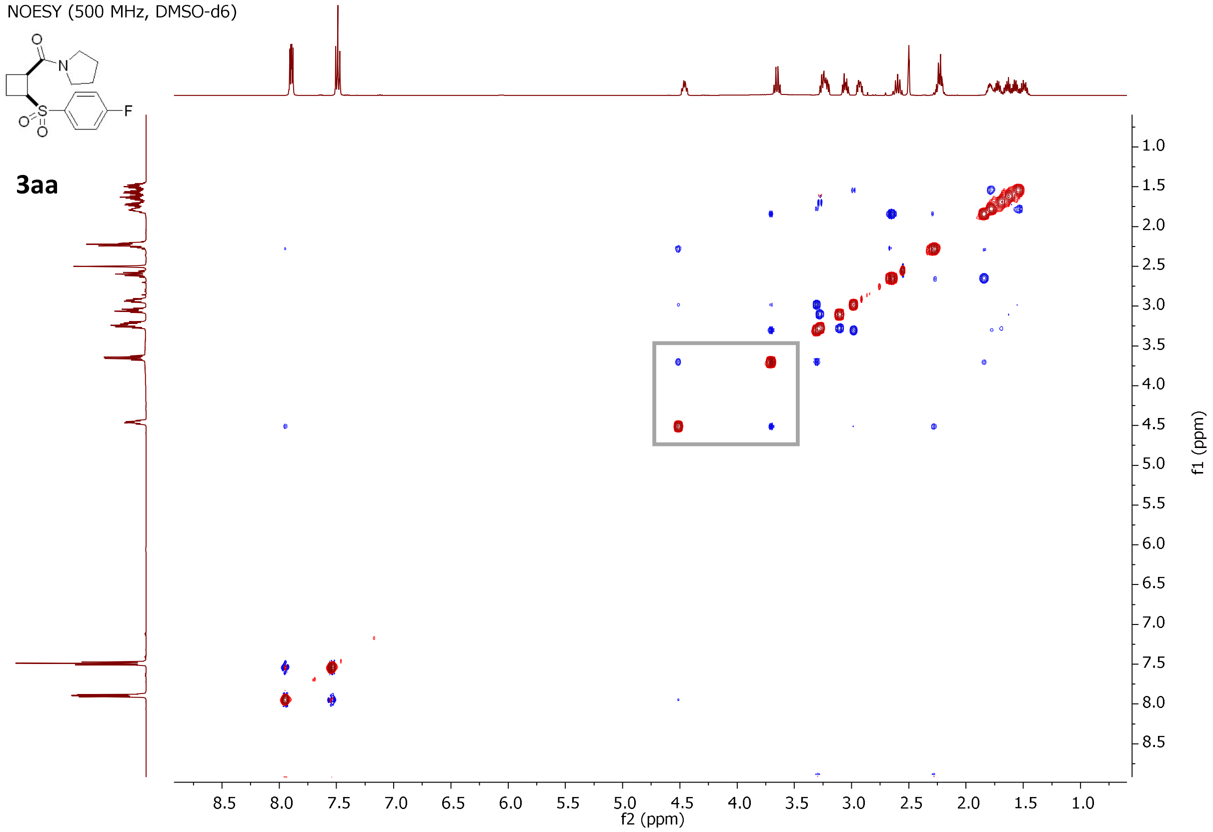

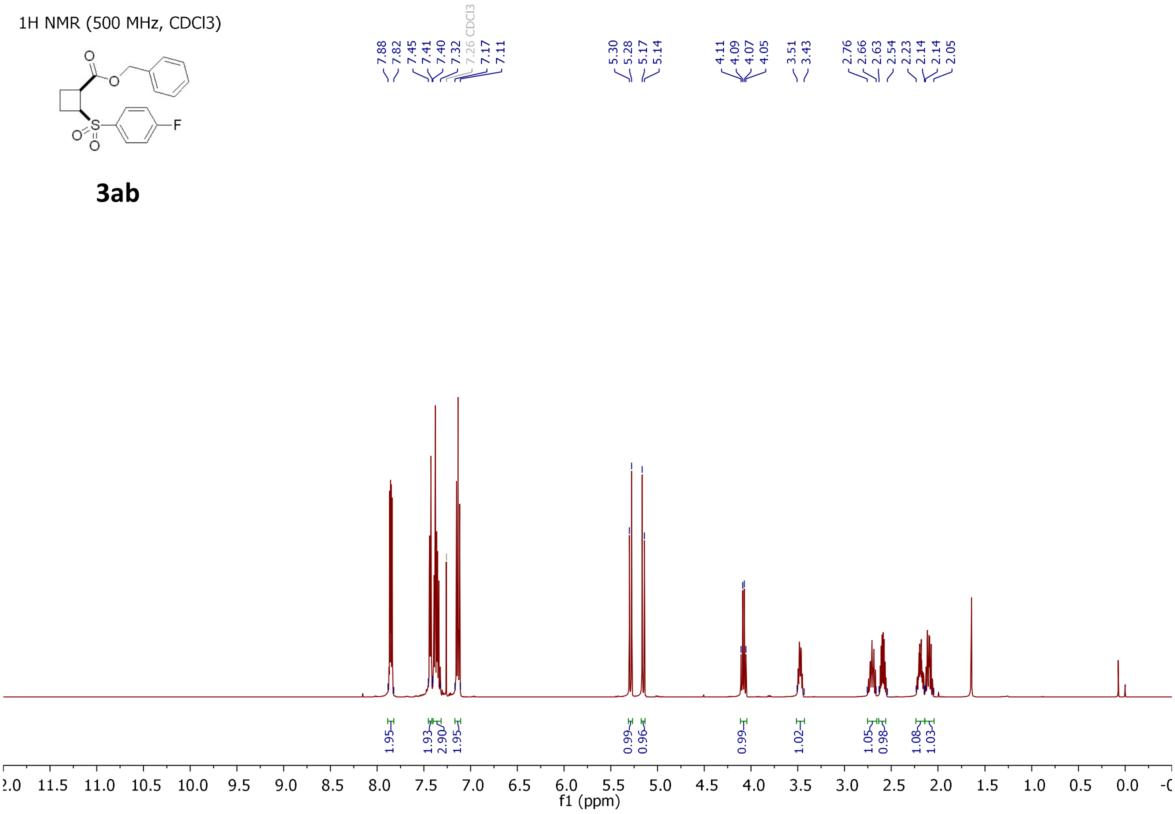
1H NMR (500 MHz, CDCl3)
3ab
7.88
7.82
7.45
7.41
7.40
7.32
7.26 CDCl3
7.17
7.11
5.30
5.28
5.17
5.14
4.11
4.09
4.07
4.05
3.51
3.43
2.76
2.66
2.63
2.54
2.23
2.14
2.14
2.05
1.95
1.93
2.90
1.95
0.99
0.96
0.99
1.02
1.05
0.98
1.08
1.03
f1 (ppm)

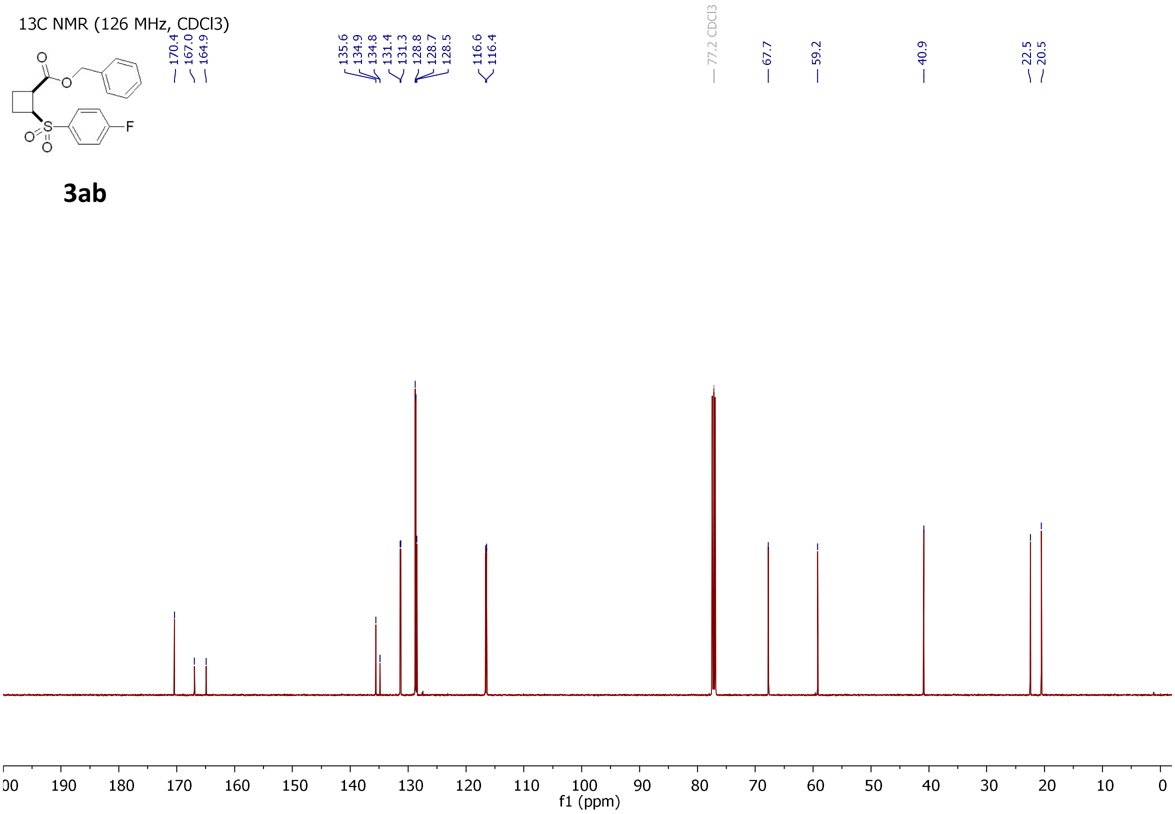
13C NMR (126 MHz, CDCl3)
3ab
170.4
167.0
164.9
135.6
134.9
134.8
131.4
131.3
128.8
128.7
128.5
116.6
116.4
77.2 CDCl3
67.7
59.2
40.9
22.5
20.5
f1 (ppm)

19F NMR (471 MHz, CDCl3)

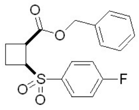

3ab

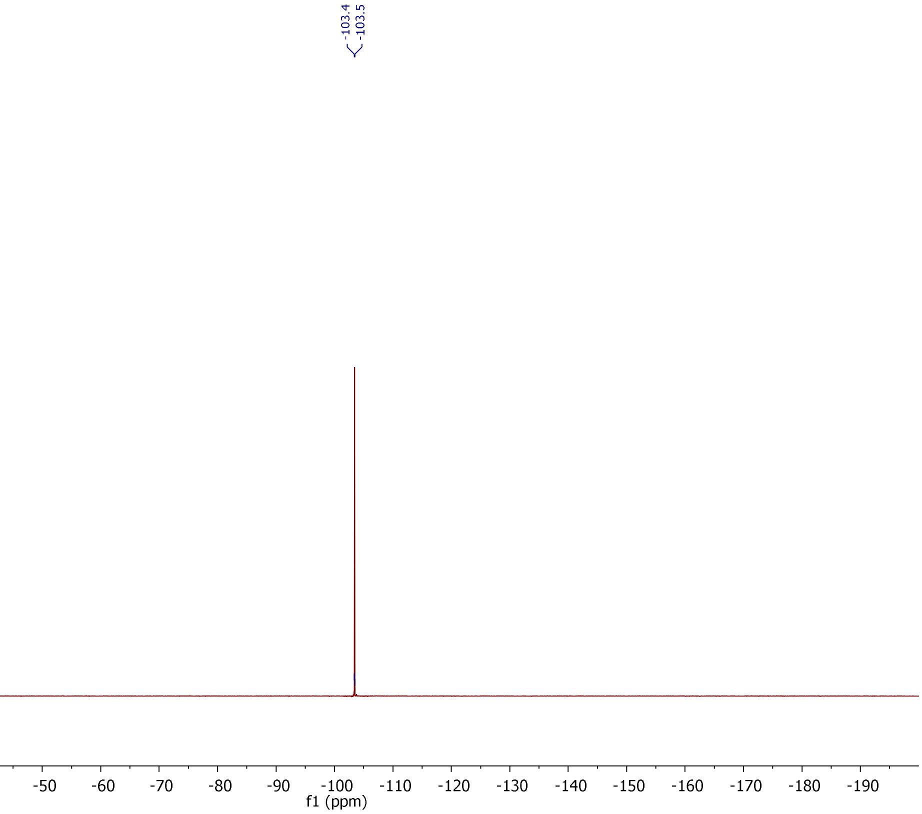

NOESY (500 MHz, CDCl3)

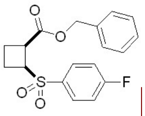

3ab

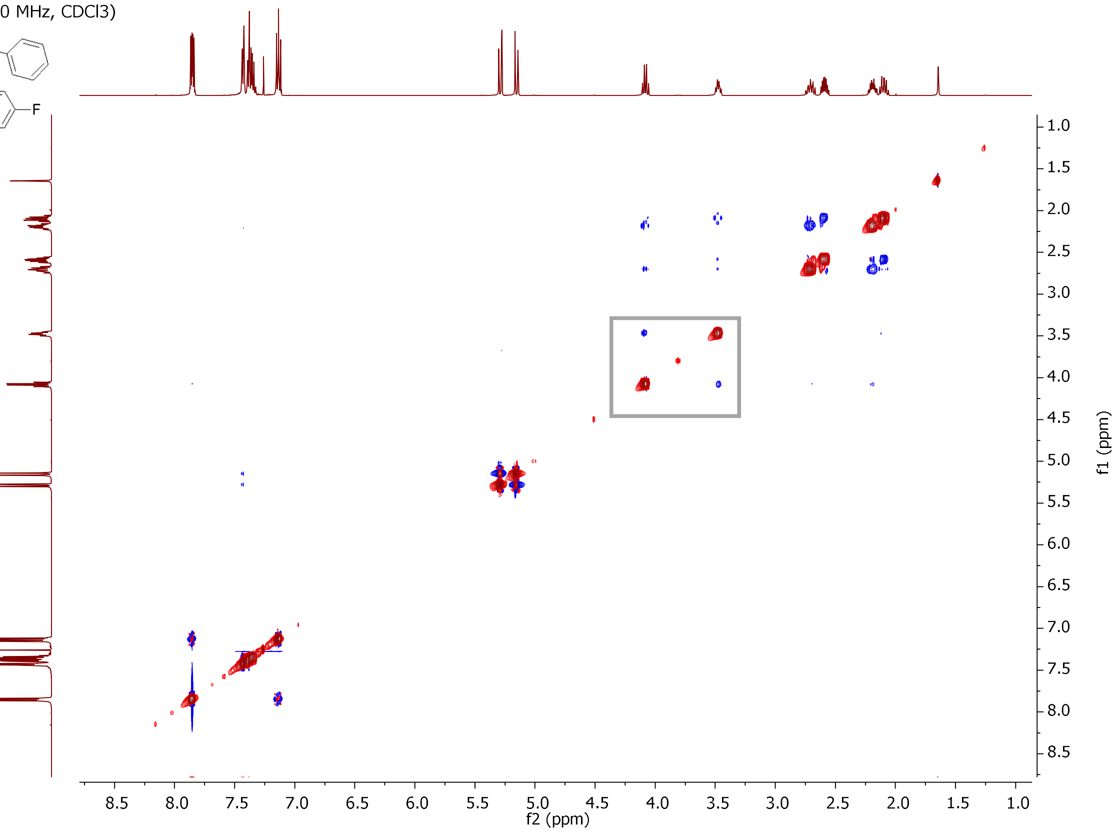

1H NMR (500 MHz, CDCl3)

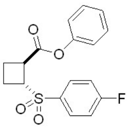

***trans*-3ac**

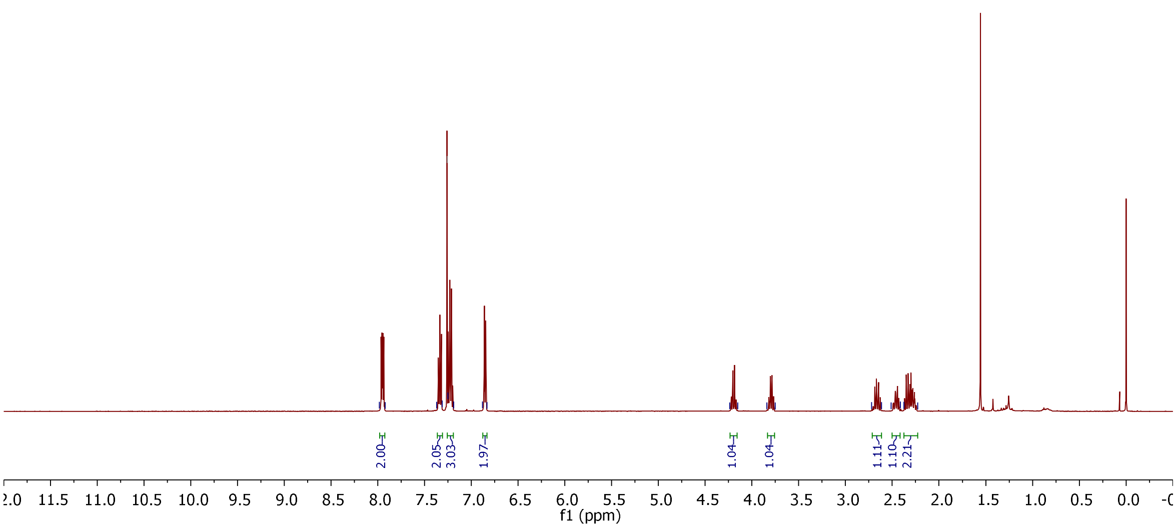

13C NMR (126 MHz, CDCl3)

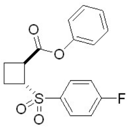

***trans*-3ac**

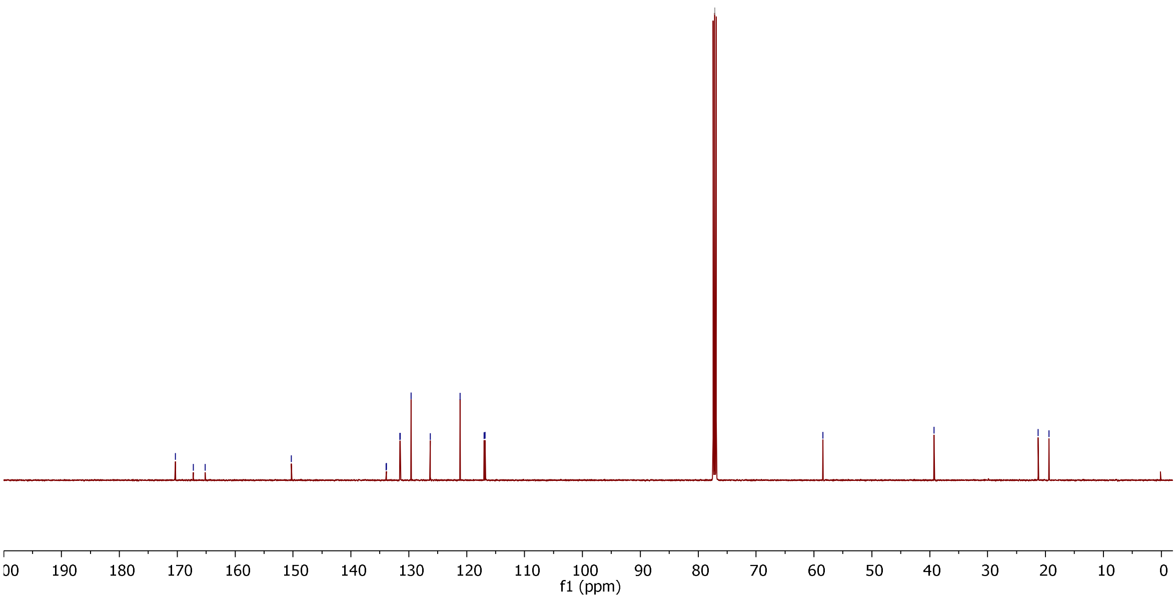

19F NMR (471 MHz, CDCl3)

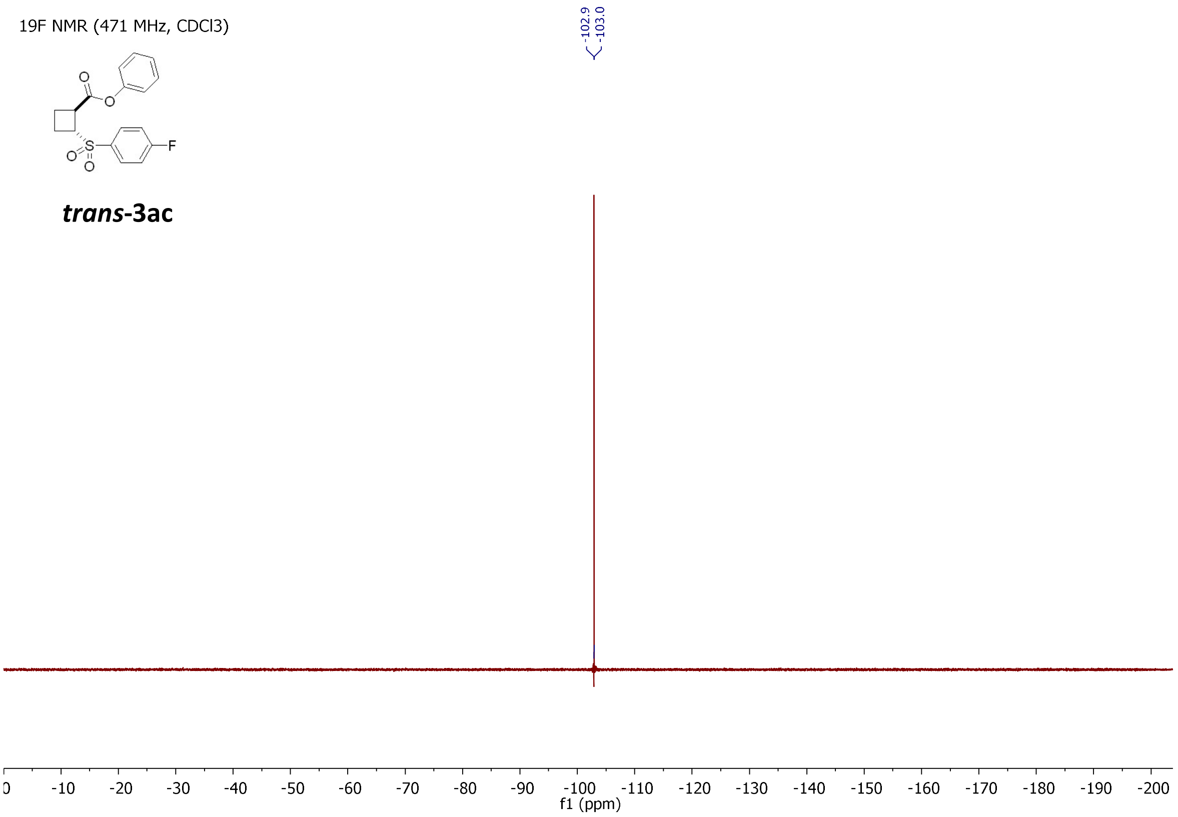

NOESY (500 MHz, CDCl3)

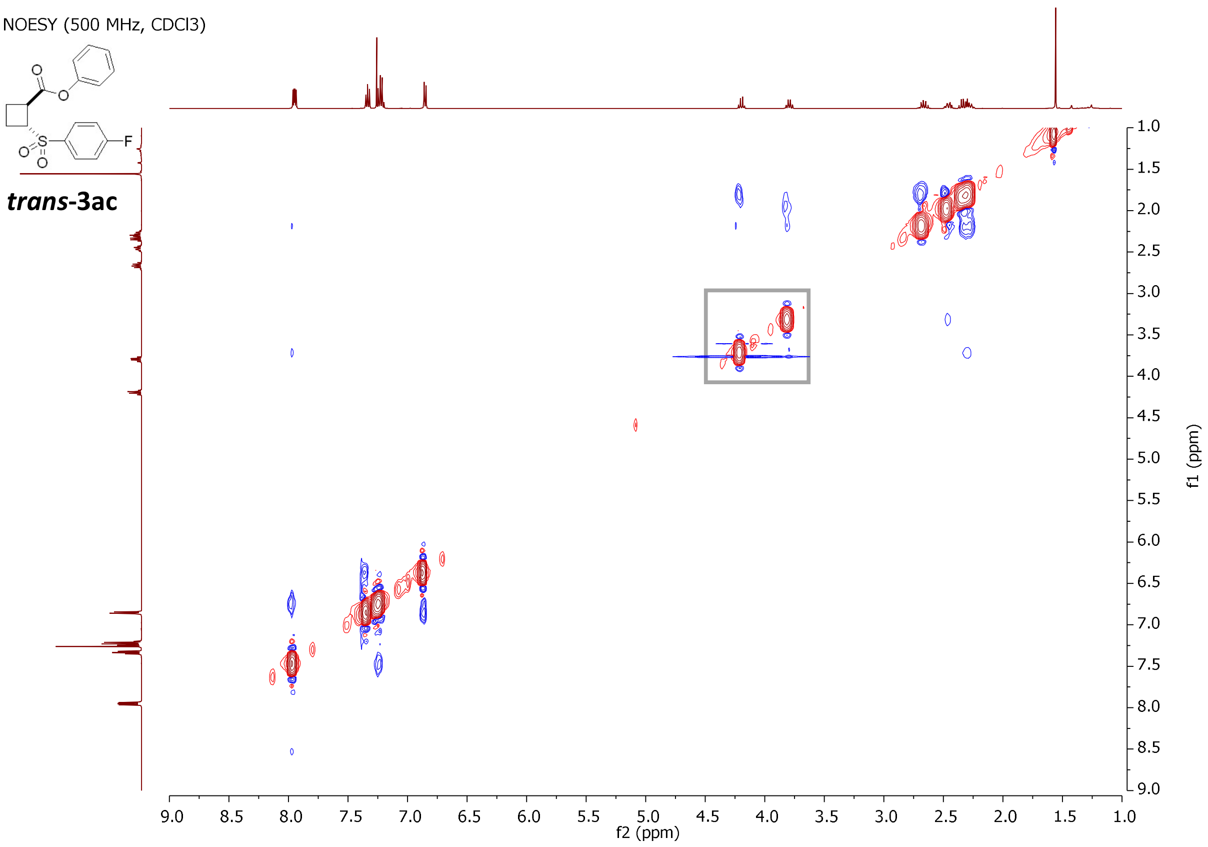

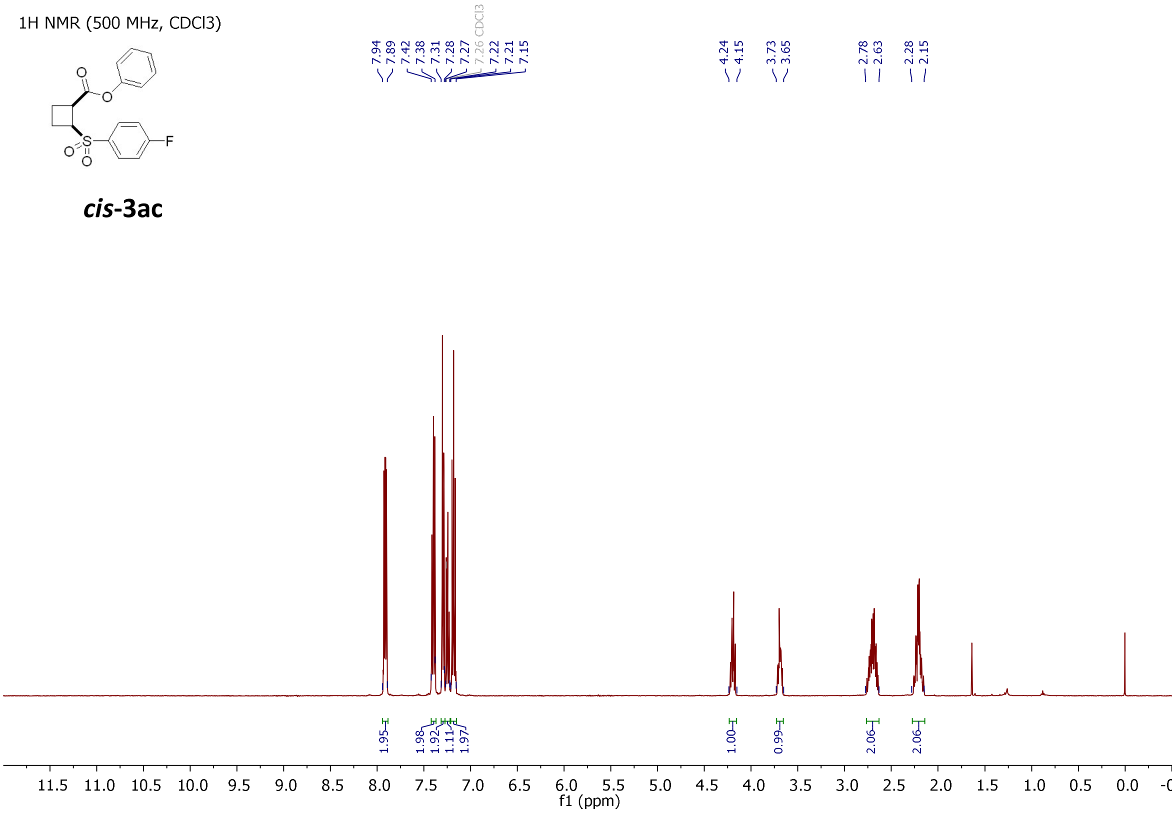
1H NMR (500 MHz, CDCl3)
cis-3ac
7.94
7.89
7.42
7.38
7.31
7.28
7.27
7.26 CDCl3
7.22
7.21
7.15
4.24
4.15
3.73
3.65
2.78
2.63
2.28
2.15
1.95
1.98
1.92
1.11
1.97
1.00
0.99
2.06
2.06
f1 (ppm)

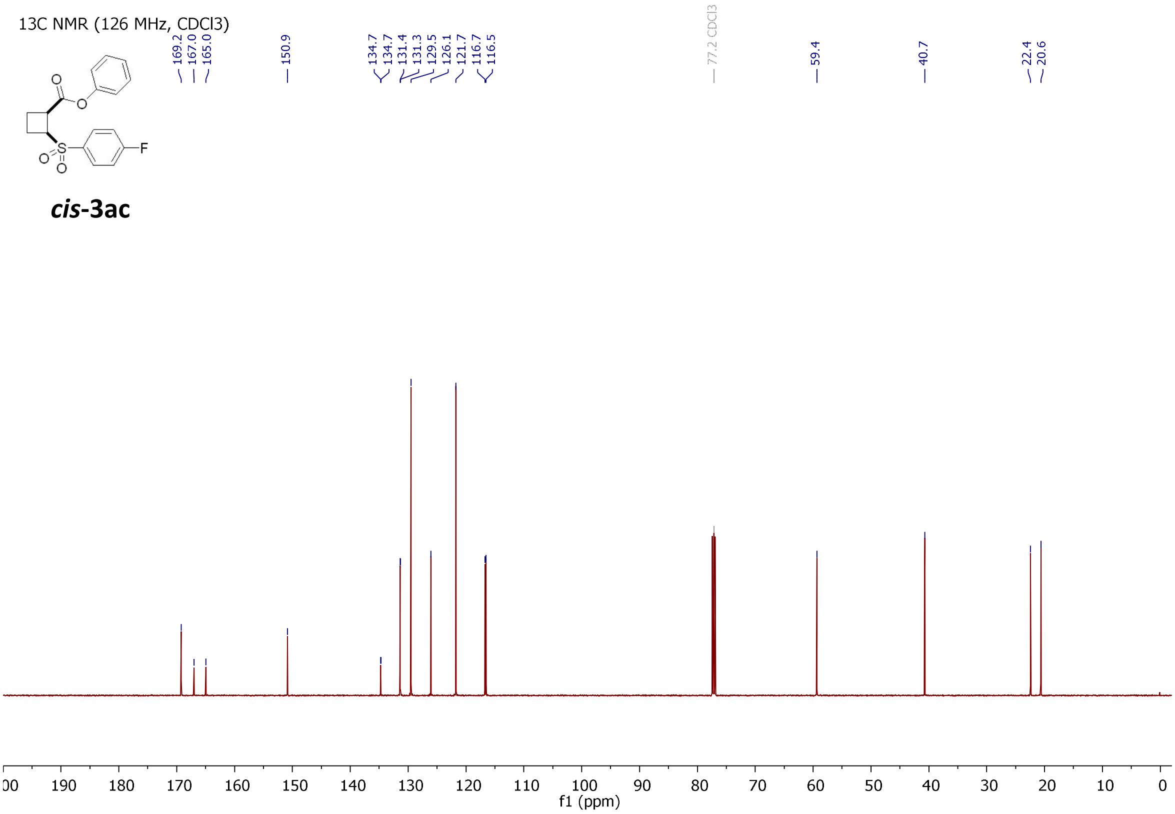
13C NMR (126 MHz, CDCl3)
cis-3ac
169.2
167.0
165.0
150.9
134.7
134.7
131.4
131.3
129.5
126.1
121.7
116.7
116.5
77.2 CDCl3
59.4
40.7
22.4
20.6
f1 (ppm)

19F NMR (471 MHz, CDCl3)

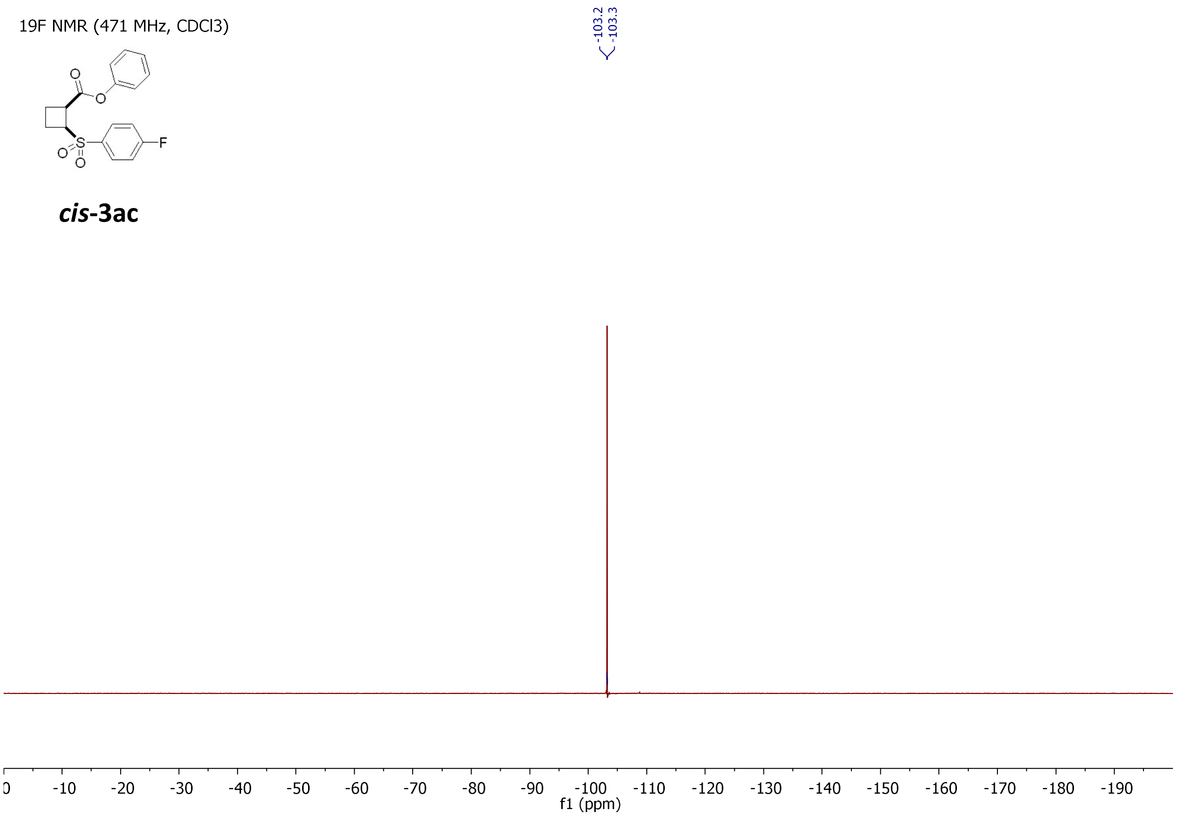

NOESY (500 MHz, CDCl3)

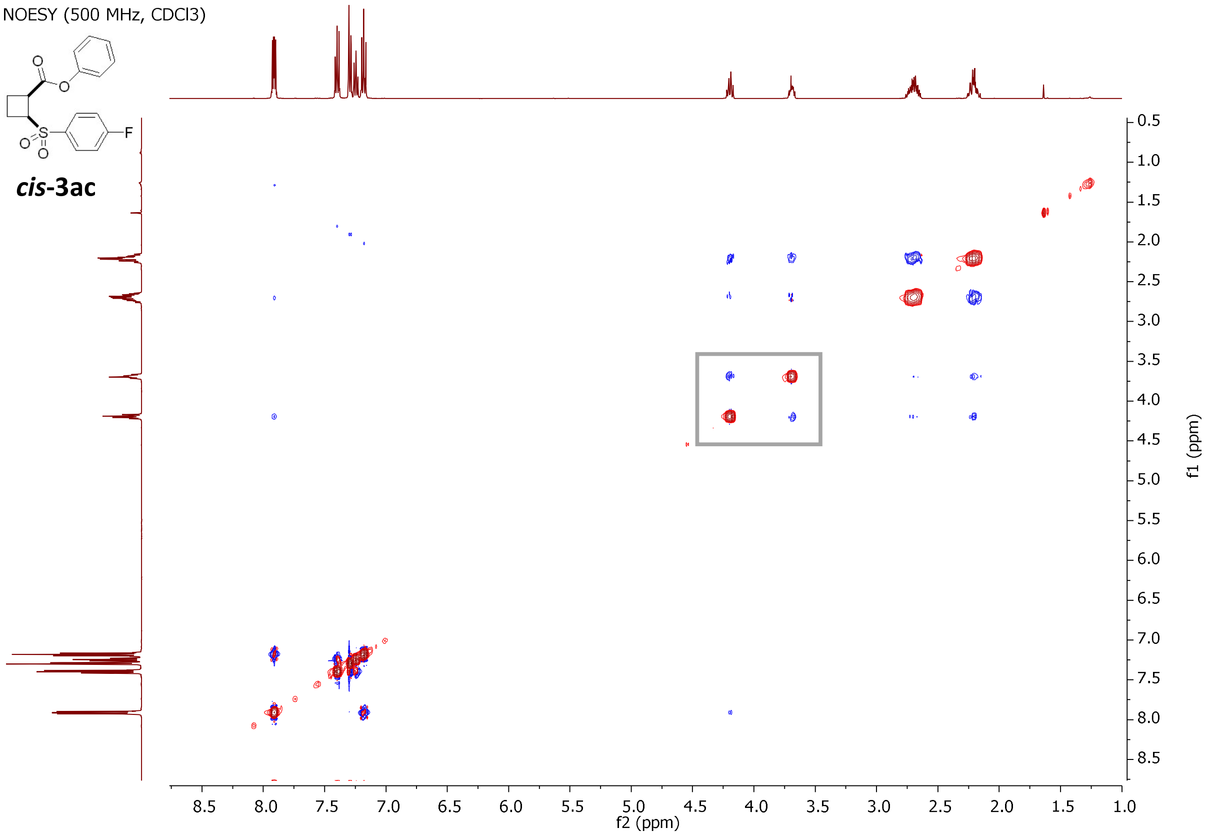

1H NMR (400 MHz, CDCl3)

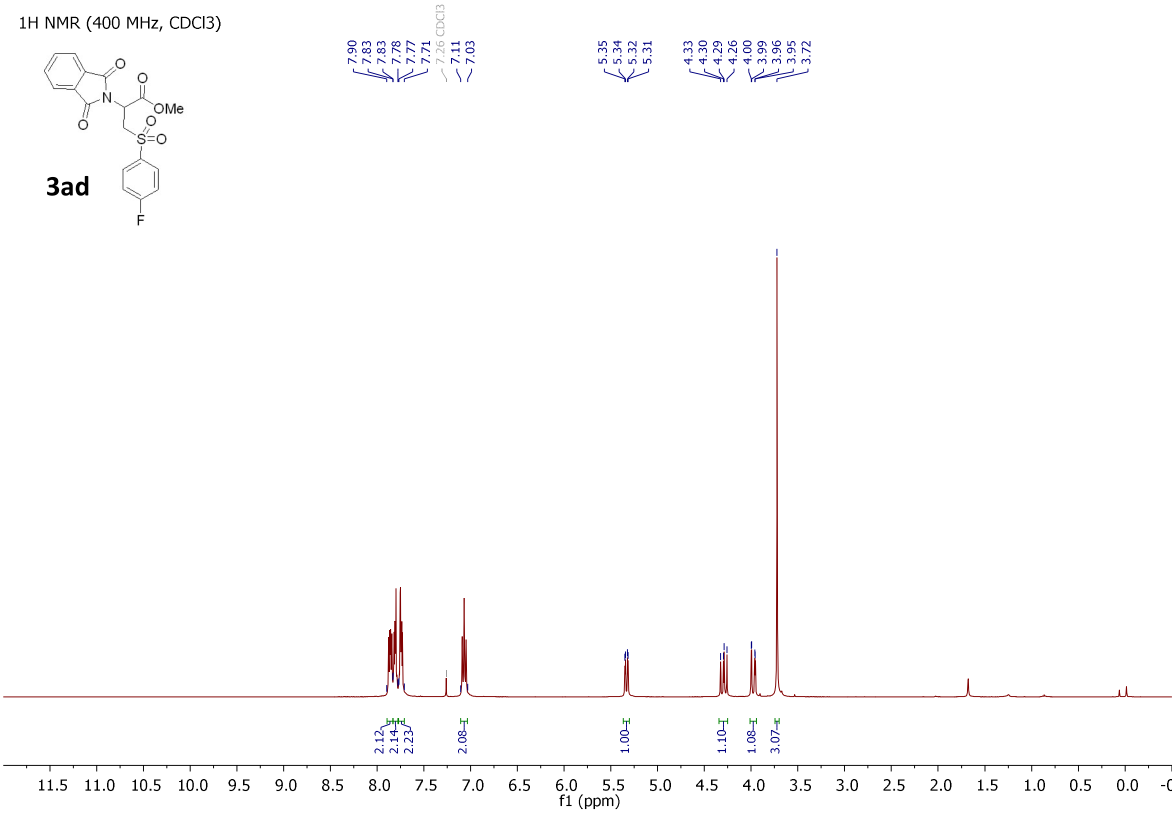

13C NMR (101 MHz, CDCl3)

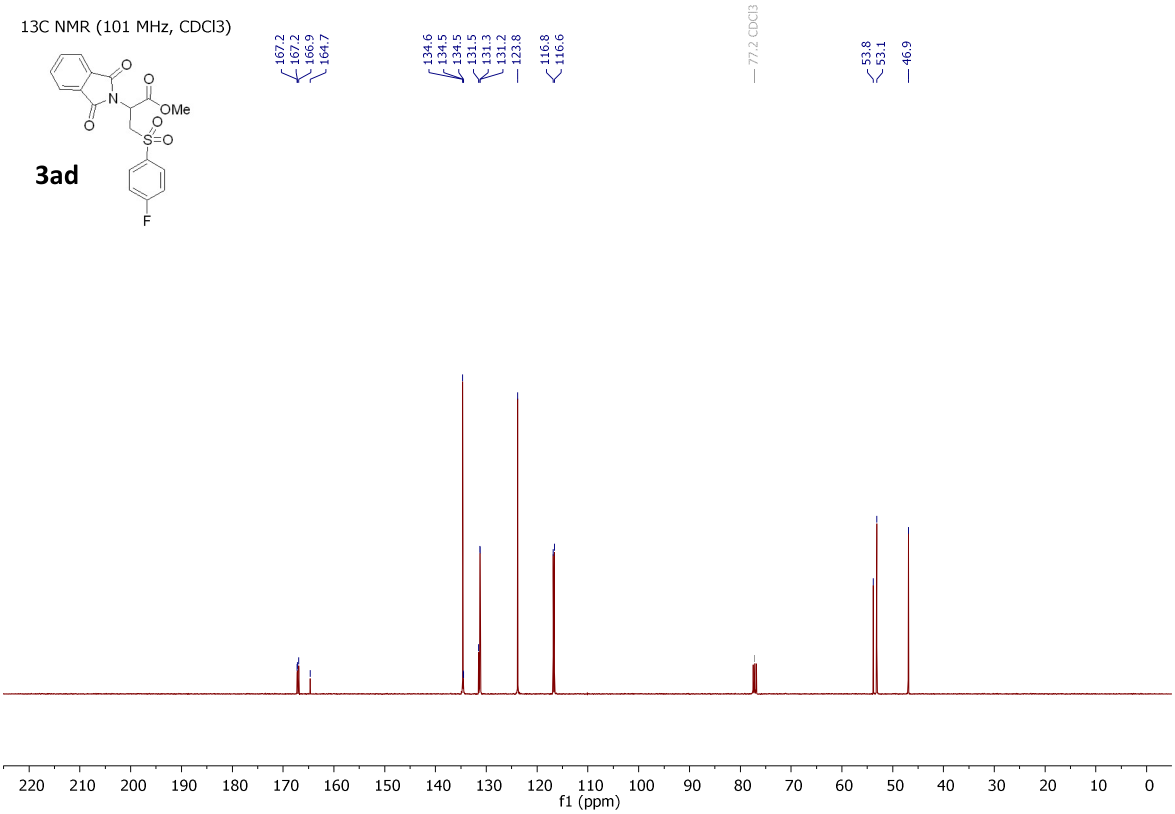

19F NMR (471 MHz, CDCl3)

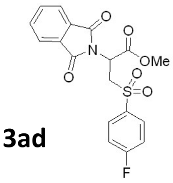

3ad

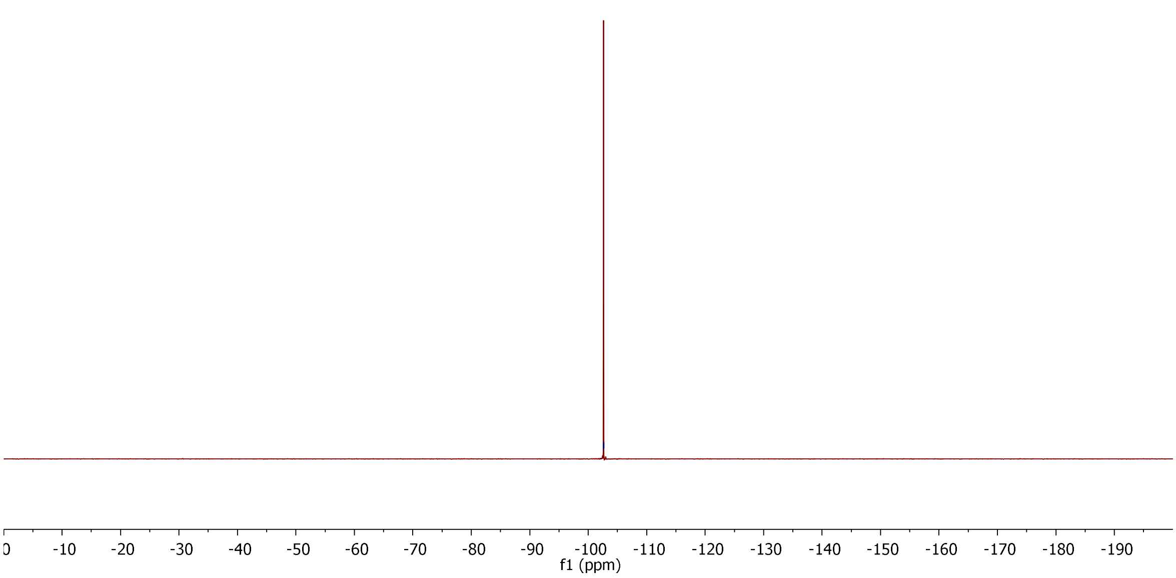

1H NMR (400 MHz, CDCl3)

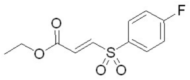

(*E*)-3ae

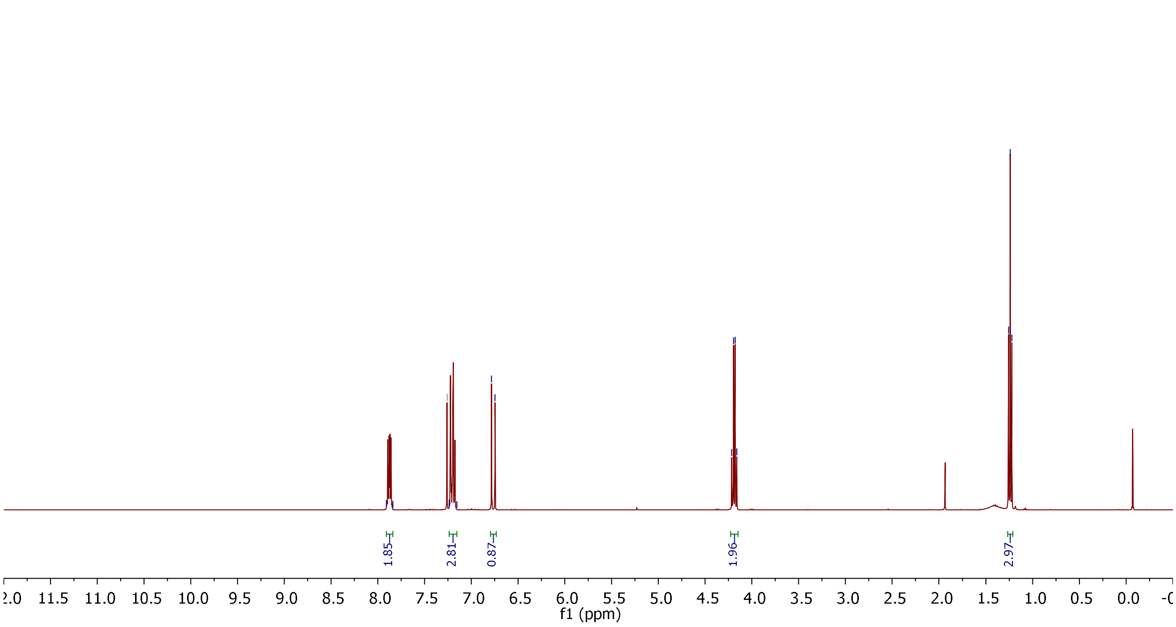

13C NMR (101 MHz, CDCl3)

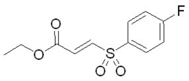

(*E*)-3ae

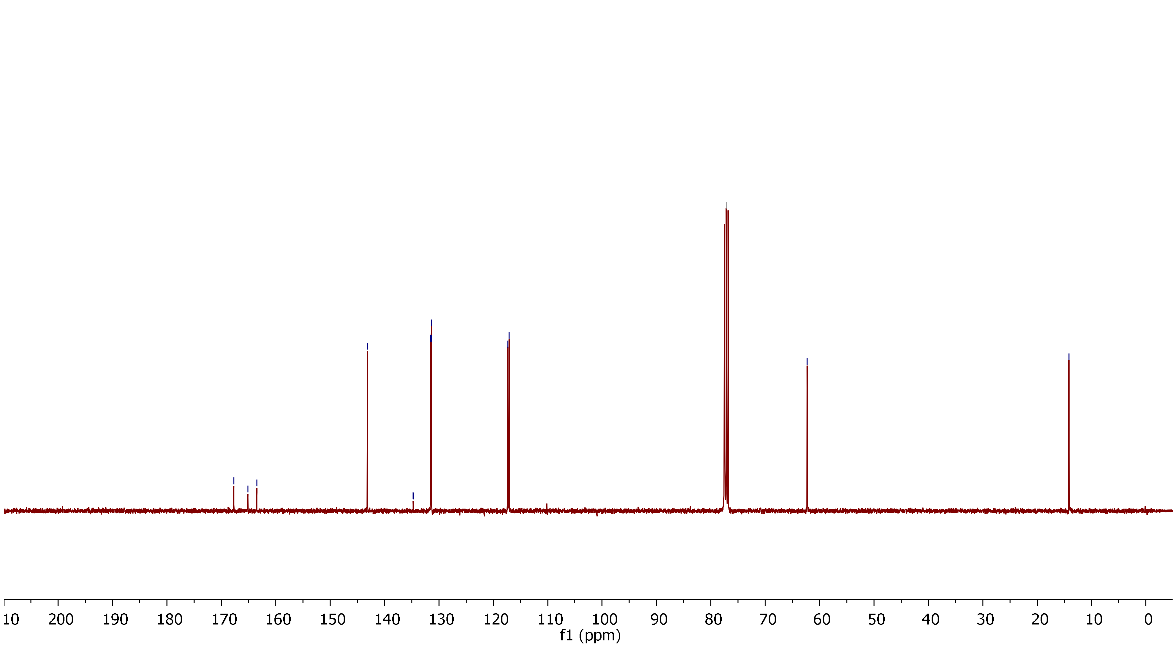

19F NMR (471 MHz, CDCl3)

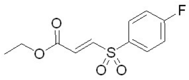

(*E*)-3ae

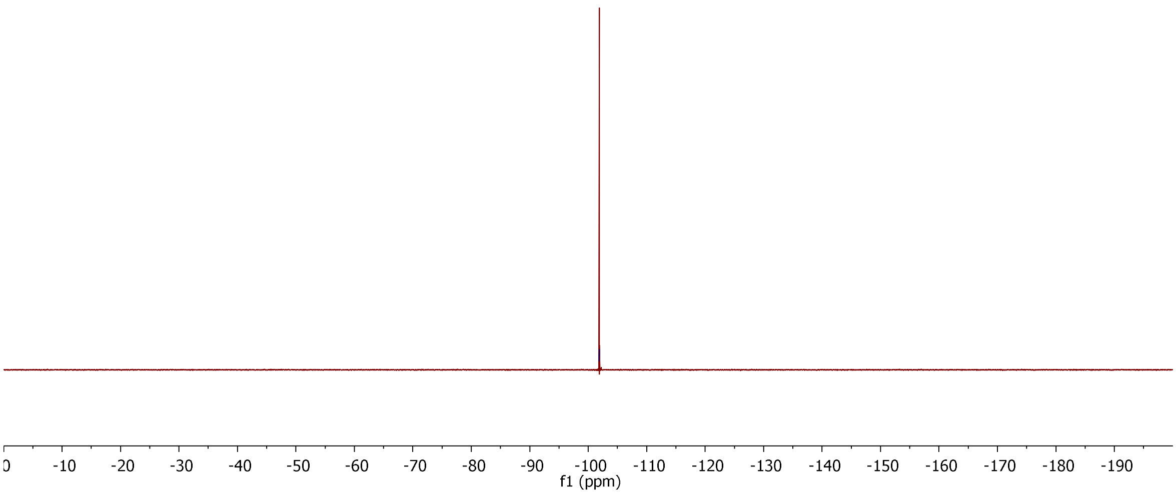

1H NMR (400 MHz, CDCl3)

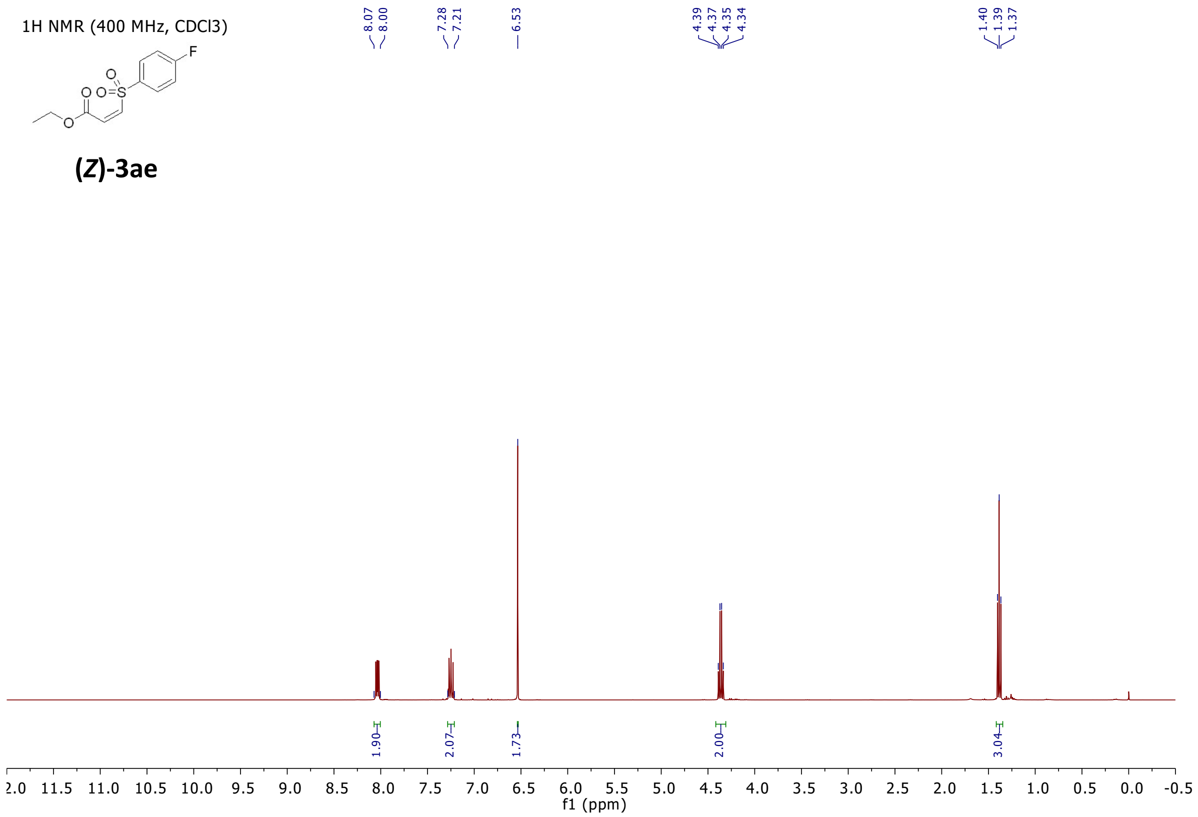

(*Z*)-3ae

13C NMR (101 MHz, CDCl3)

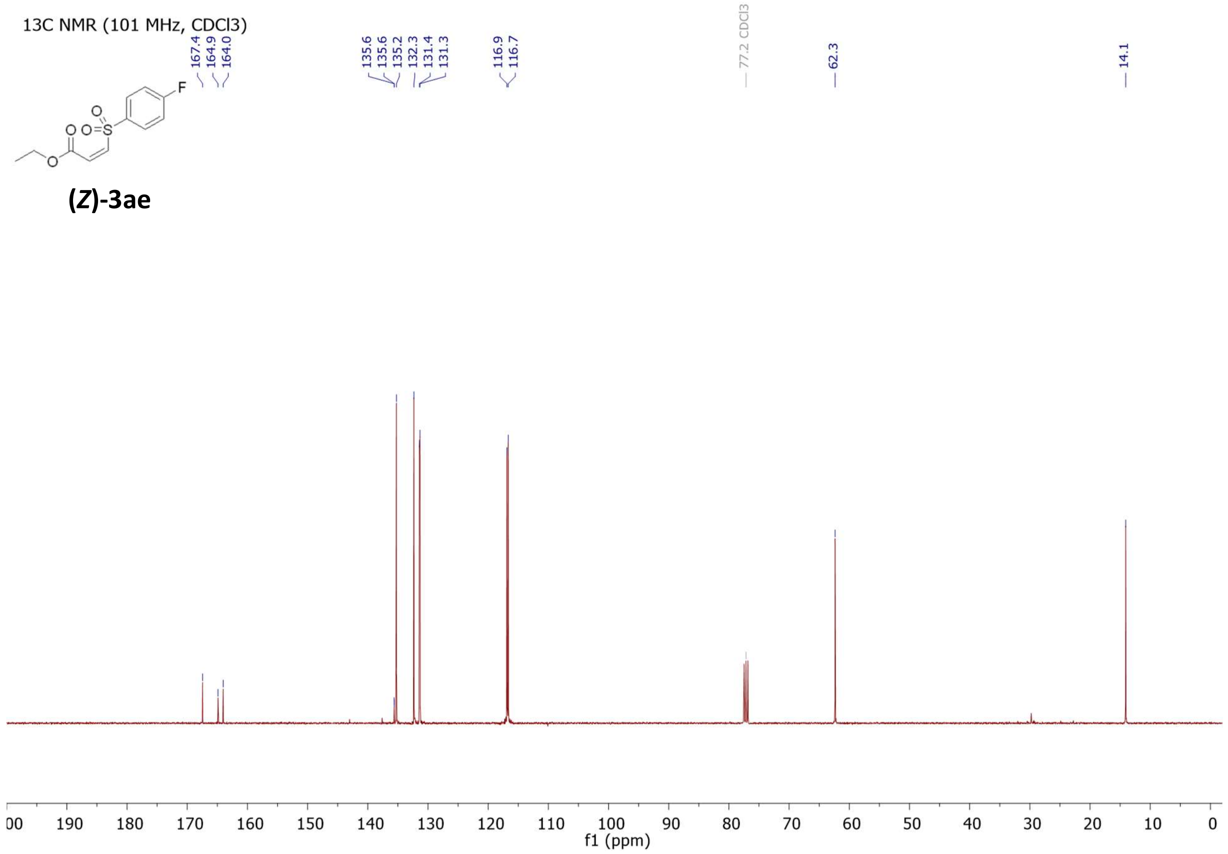

(*Z*)-3ae

19F NMR (471 MHz, CDCl3)

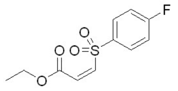

(*Z*)-3ae

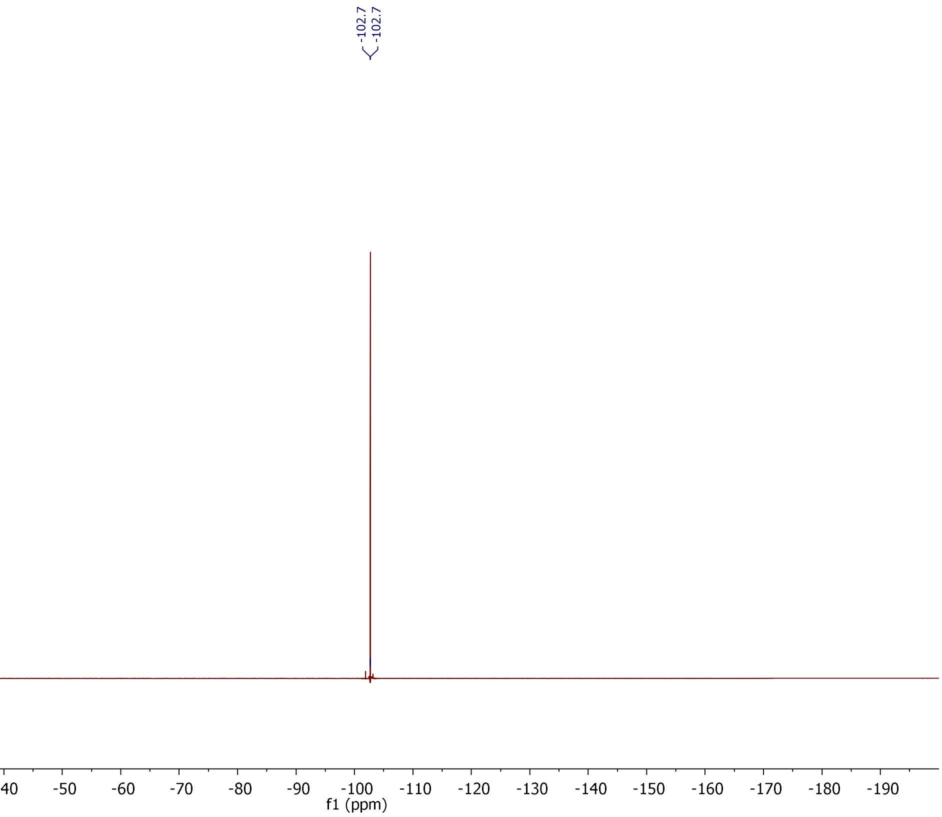

1H NMR (400 MHz, DMSO-d6)

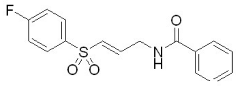

(*E*)-3af

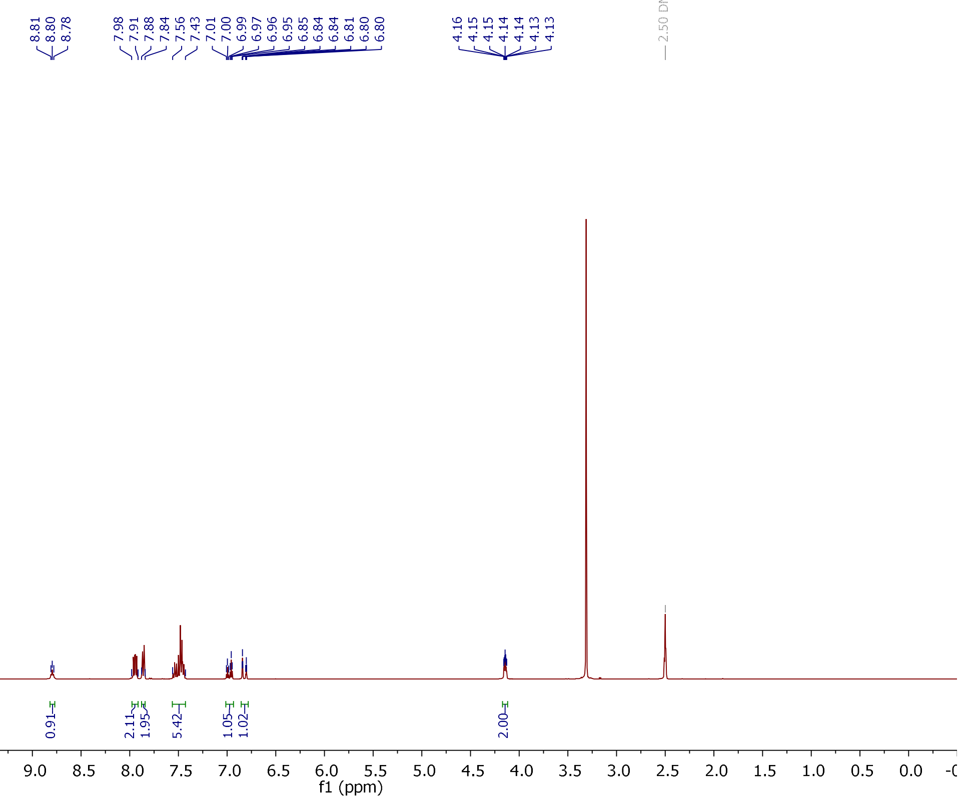

13C NMR (101 MHz, CDCl3)

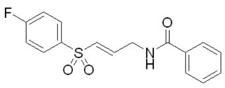

(*E*)-3af

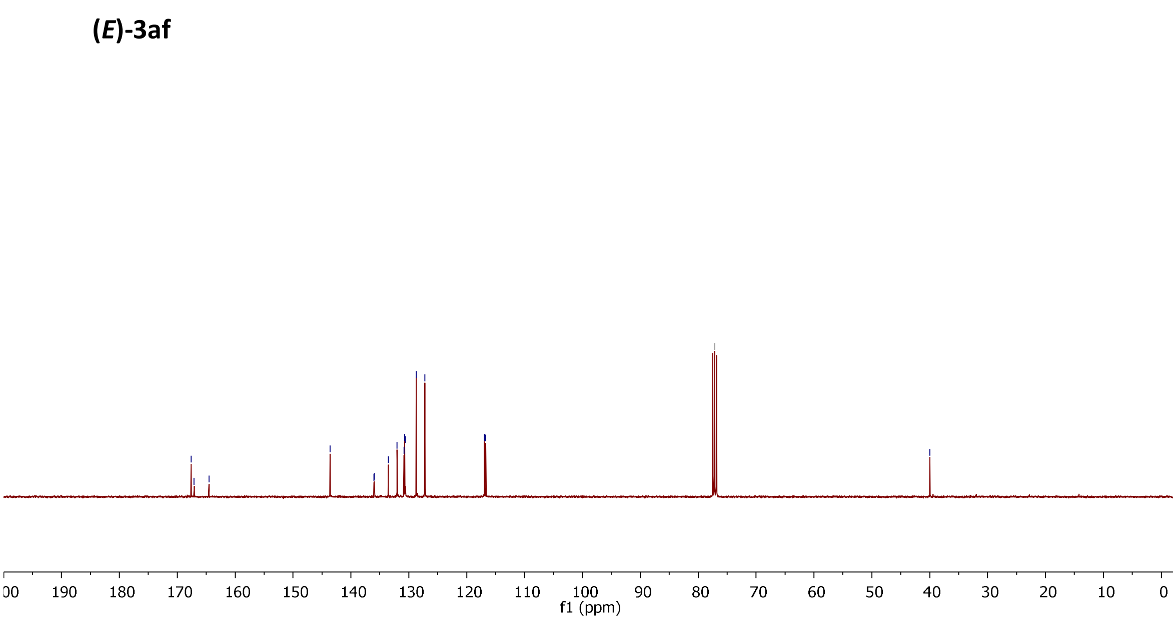

19F NMR (377 MHz, DMSO-d6)

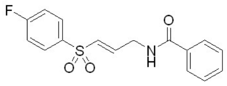

(*E*)-3af

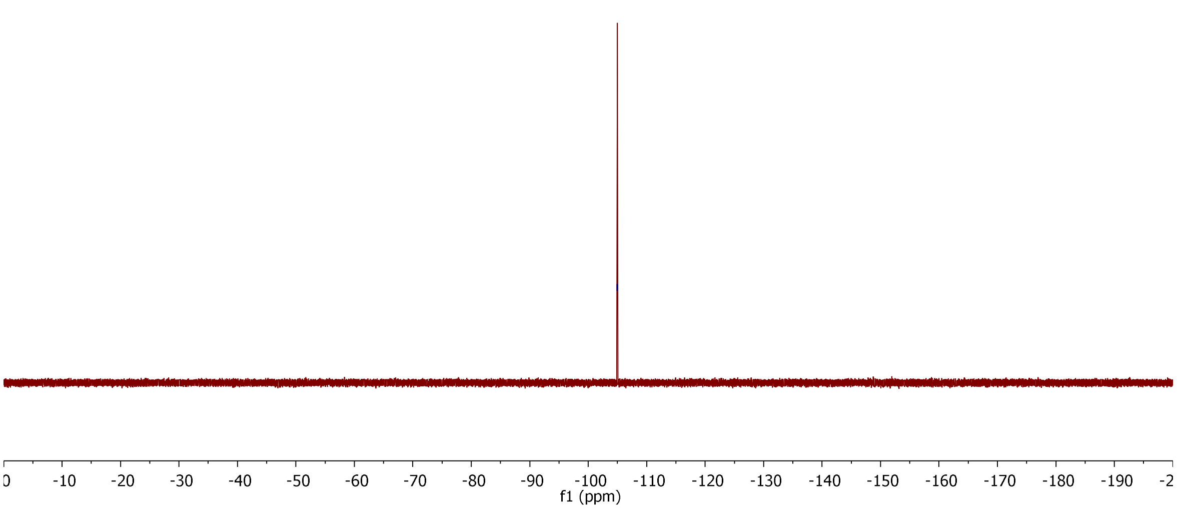

1H NMR (400 MHz, DMSO-d6)

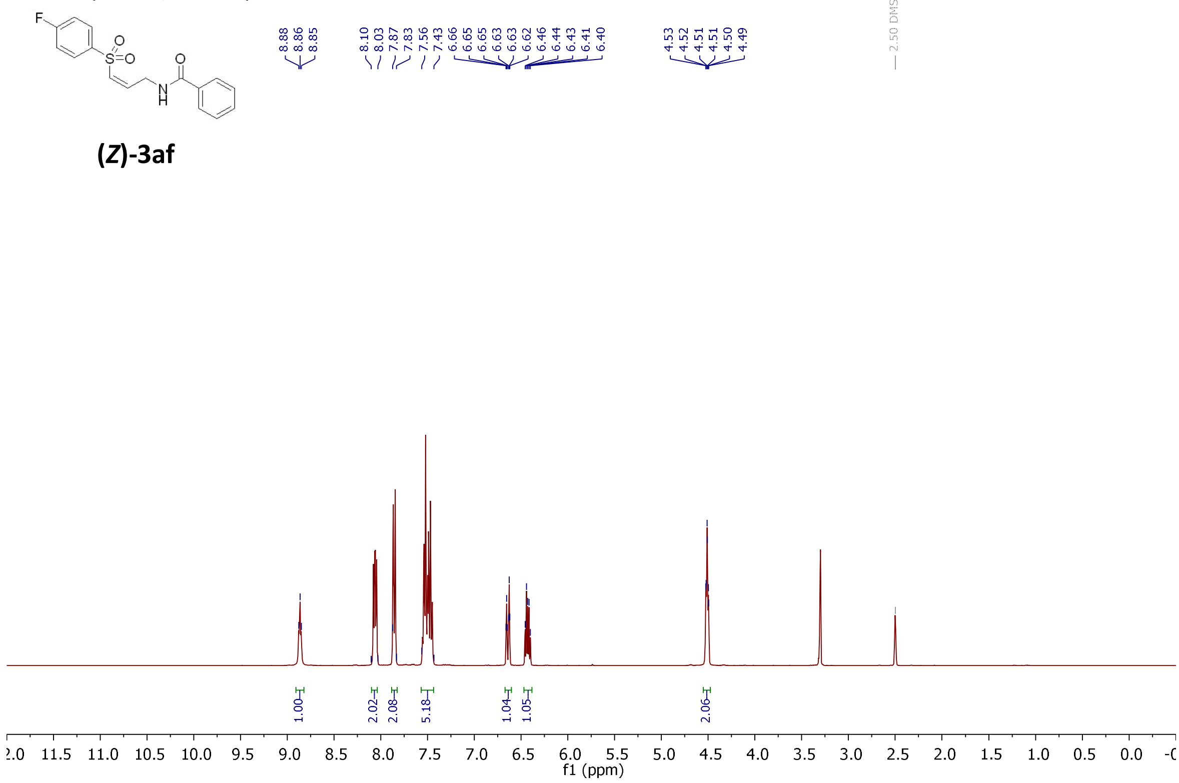

13C NMR (101 MHz, DMSO-d6)

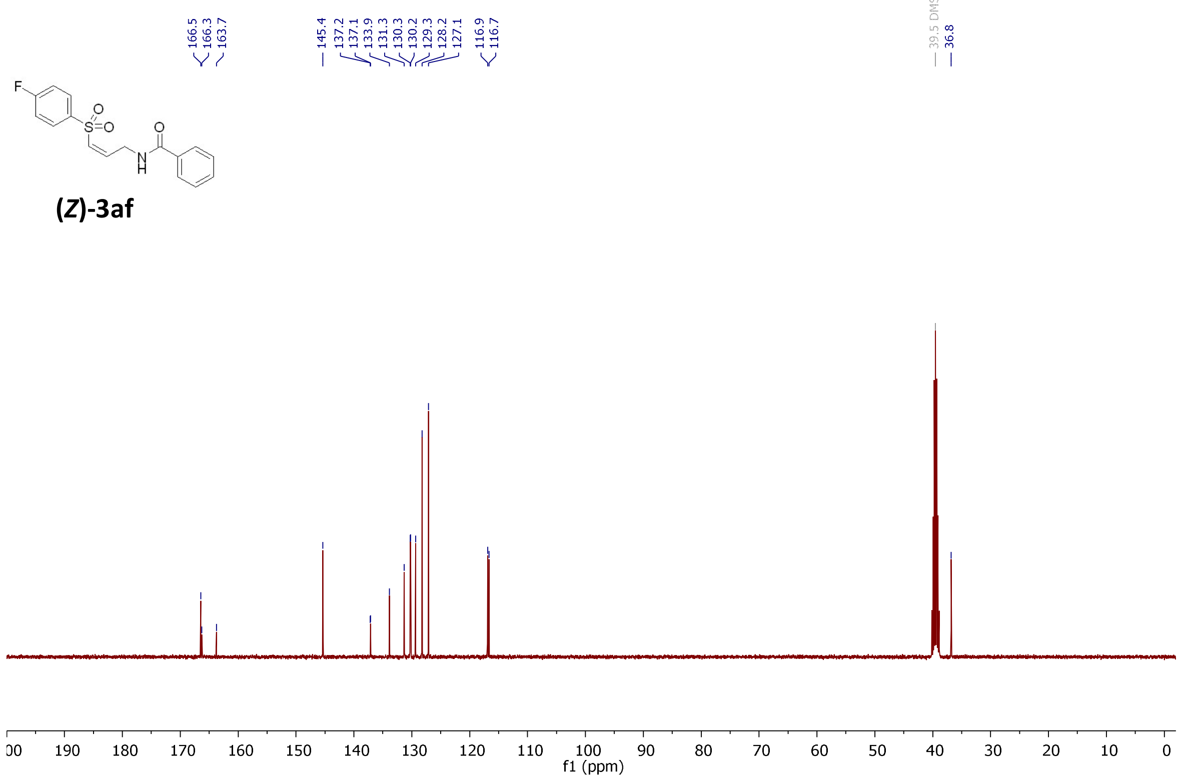

19F NMR (377 MHz, DMSO-d6)

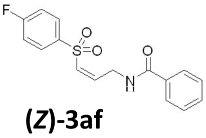

(*Z*)-3af

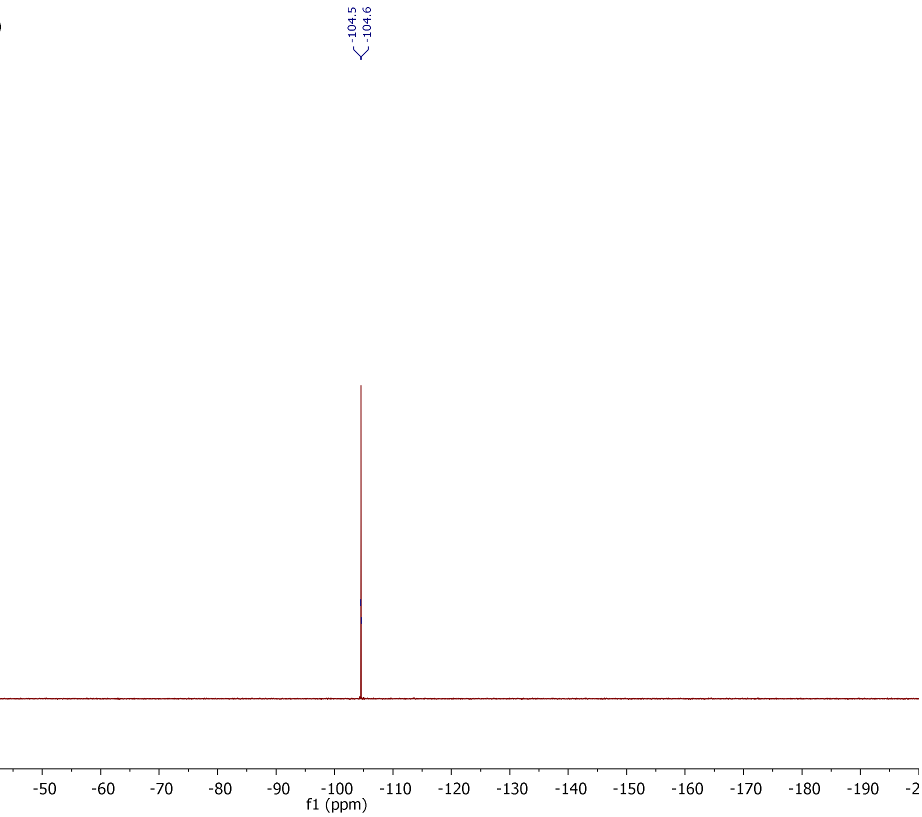

1H NMR (500 MHz, CDCl3)

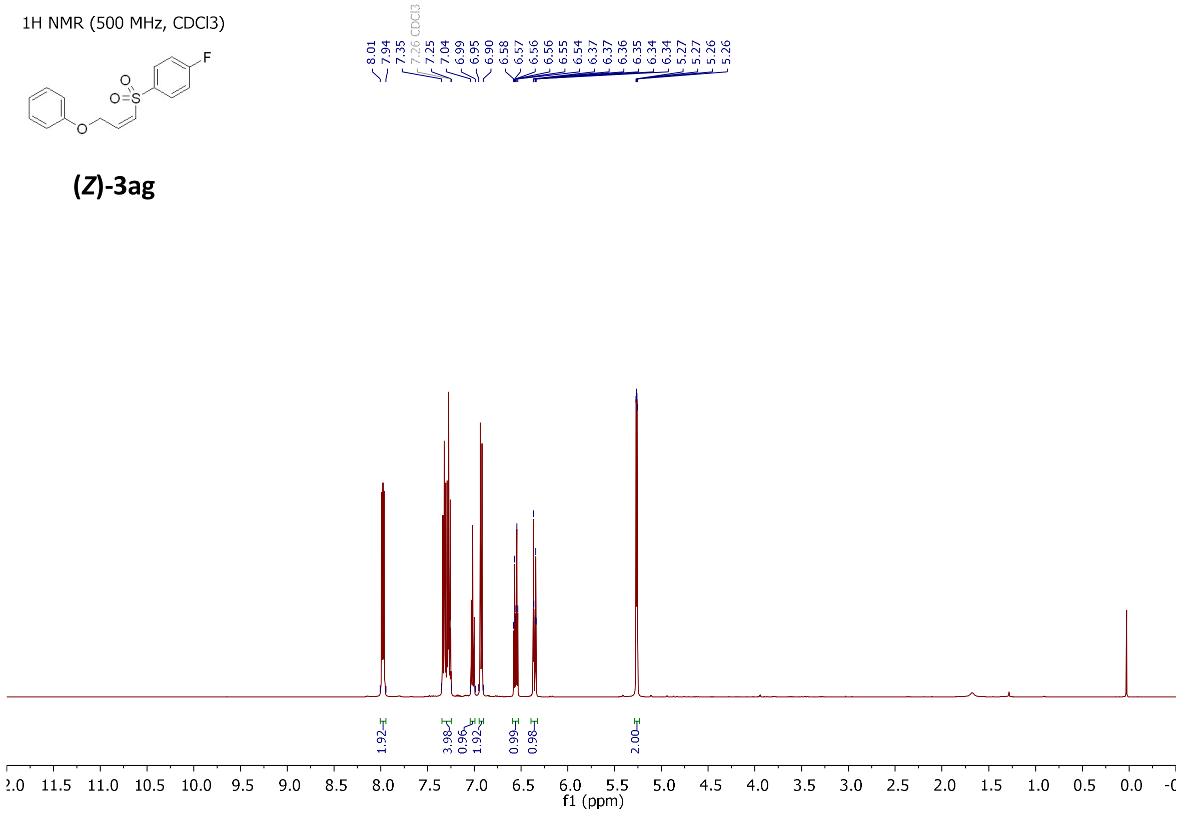

(*Z*)-3ag

13C NMR (126 MHz, CDCl3)

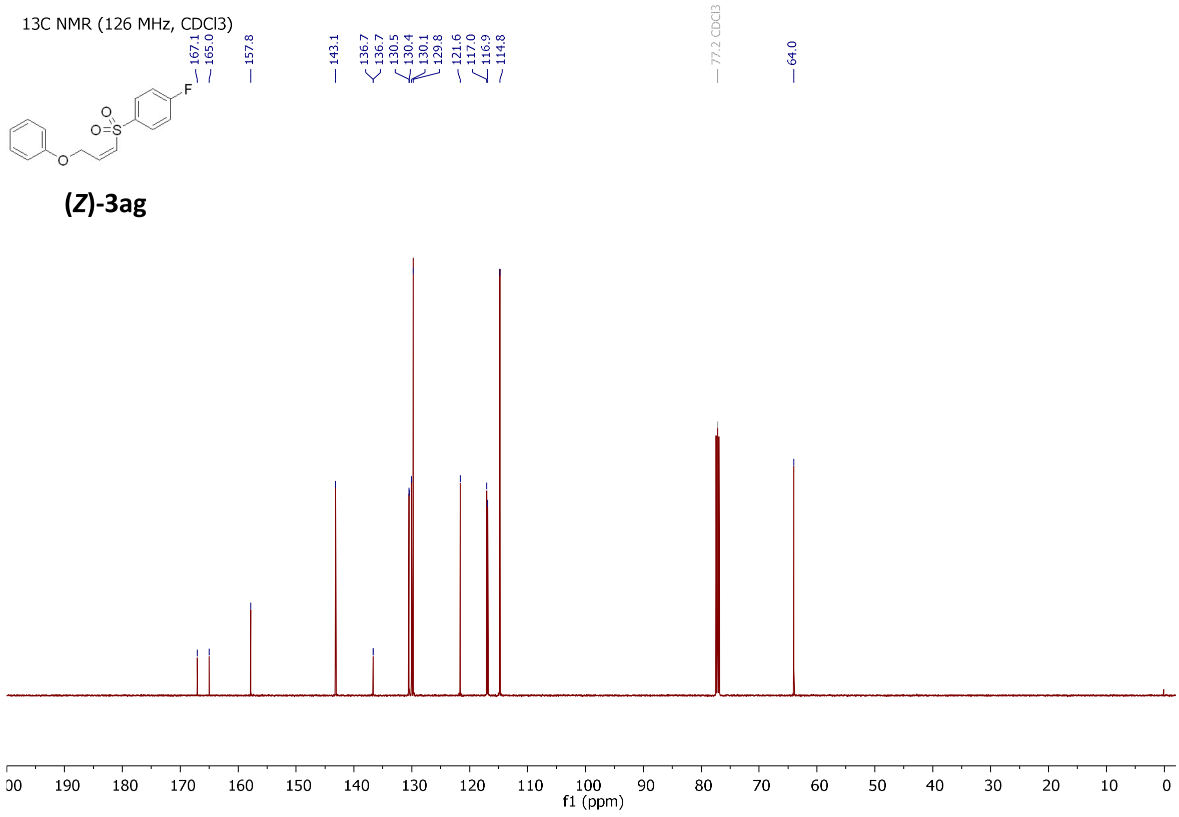

(*Z*)-3ag

19F NMR (377 MHz, CDCl3)

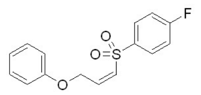

(*Z*)-3ag

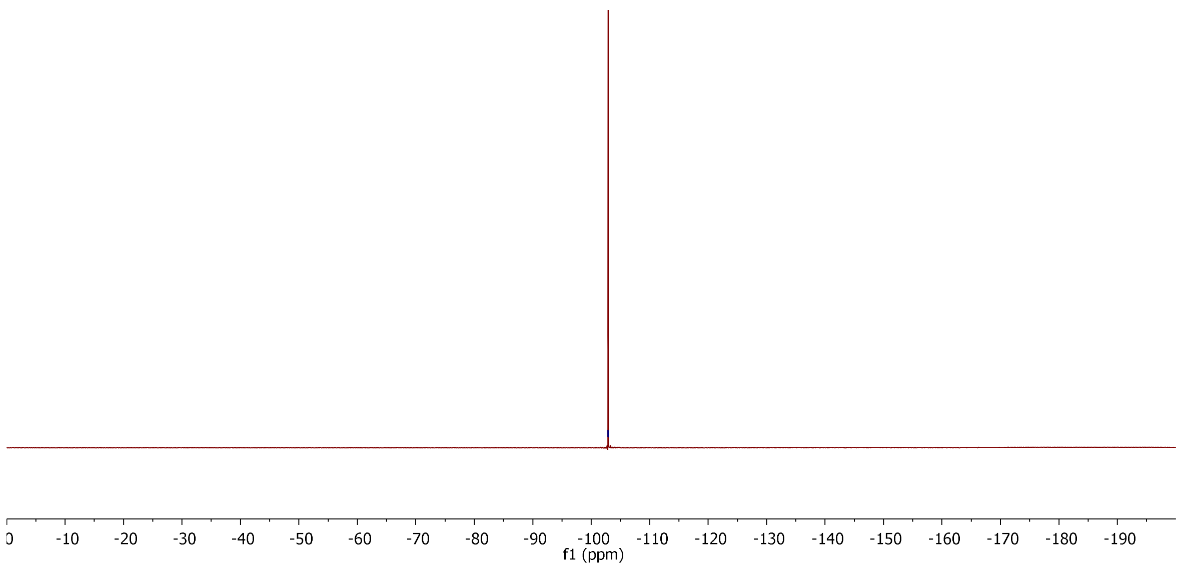

1H NMR (500 MHz, CDCl3)

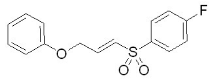

(*E*)-3ag

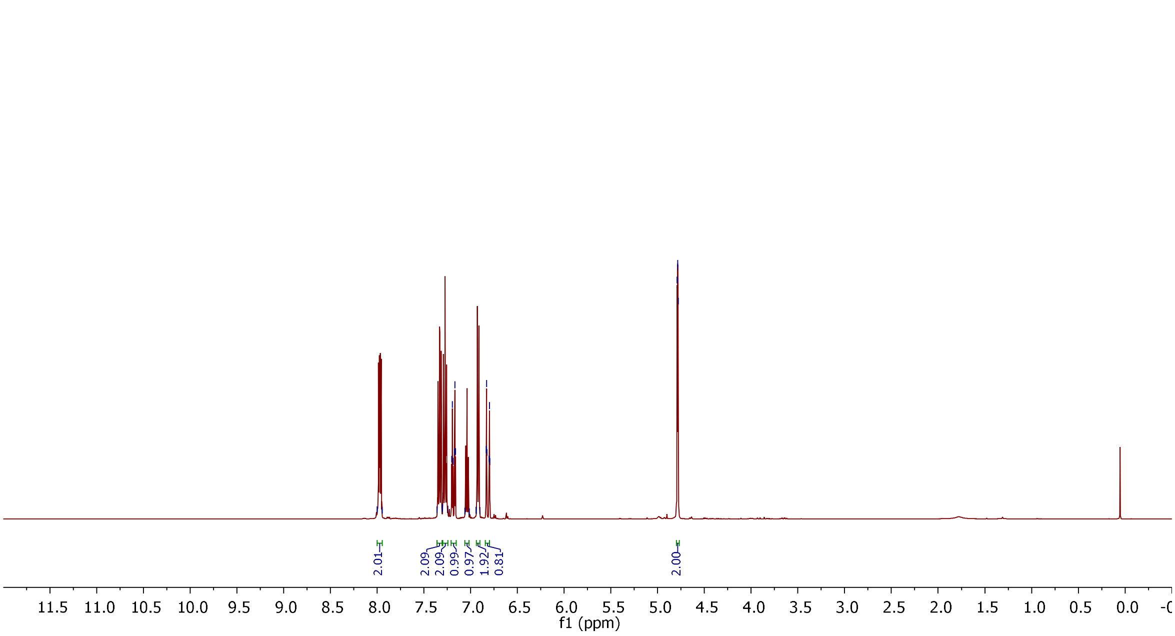

13C NMR (126 MHz, CDCl3)

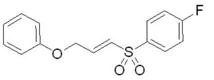

(*E*)-3ag

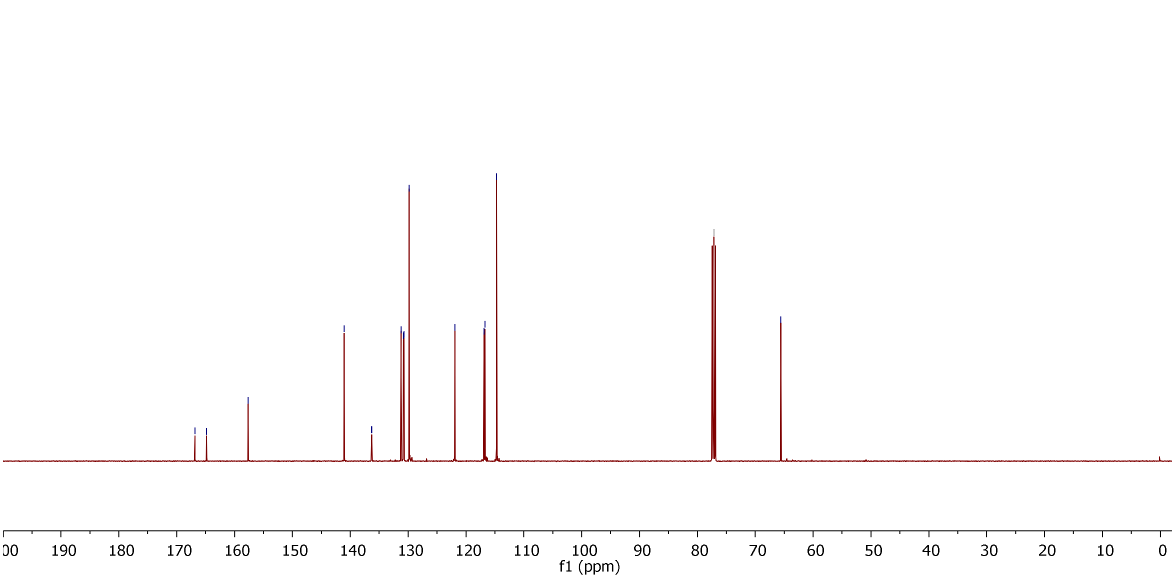

19F NMR (471 MHz, CDCl3)

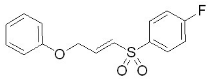

(*E*)-3ag

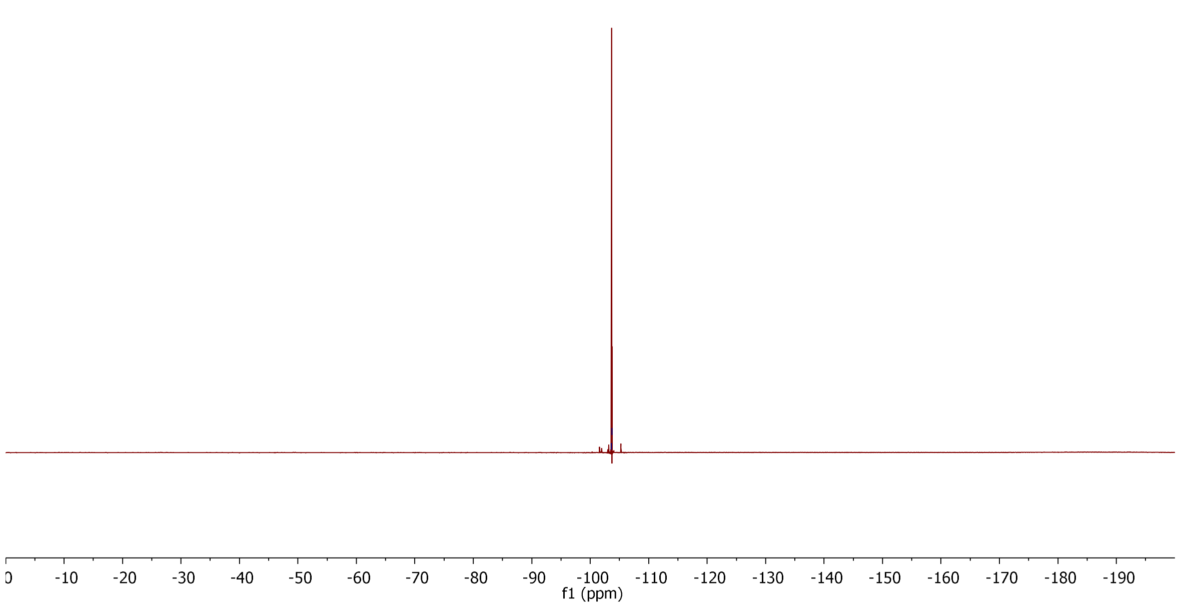

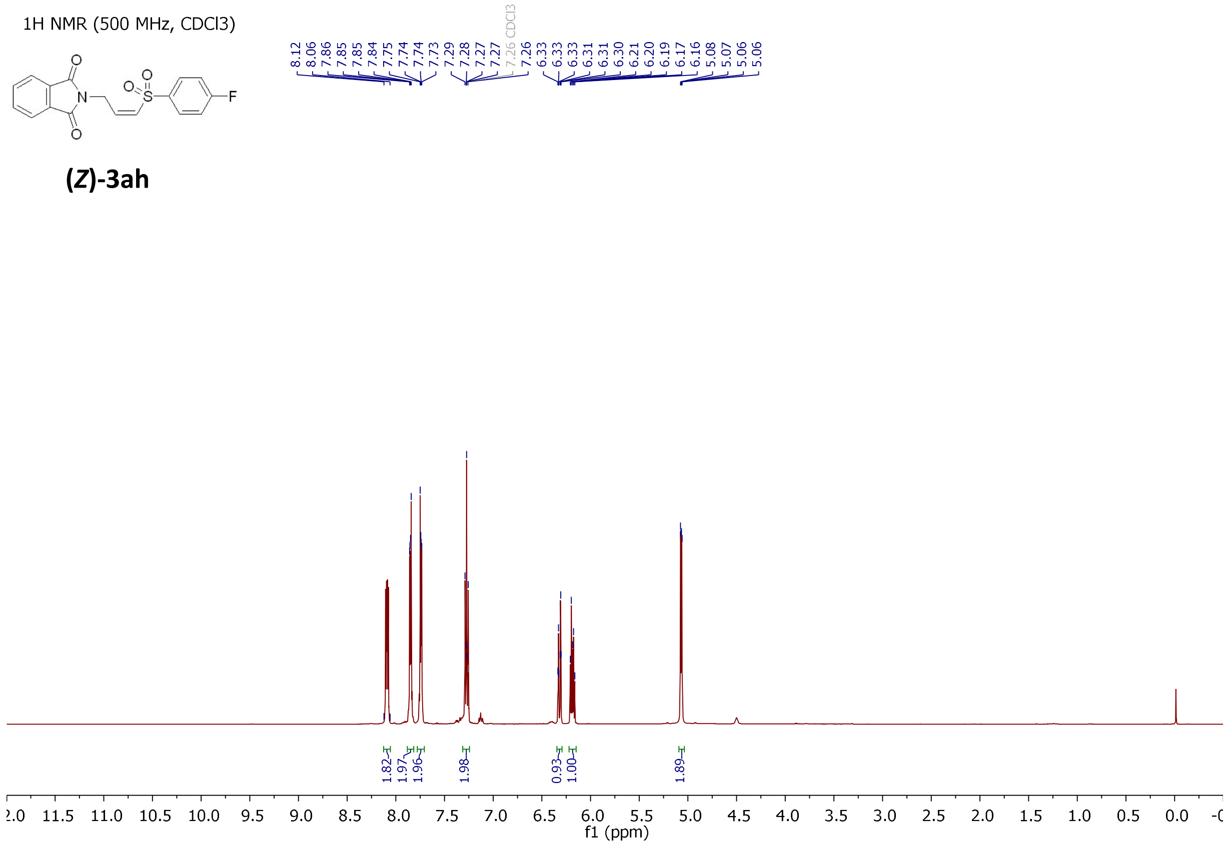

1H NMR (500 MHz, CDCl3)
O
O
N
O
O
S
F
8.12
8.06
7.86
7.85
7.85
7.84
7.75
7.74
7.74
7.73
7.29
7.28
7.27
7.27
7.26
7.26 CDCl3
6.33
6.33
6.33
6.31
6.31
6.30
6.21
6.20
6.19
6.17
6.16
5.08
5.07
5.06
5.06
1.82
1.97
1.96
1.98
0.93
1.00
1.89
2.0 11.5 11.0 10.5 10.0 9.5 9.0 8.5 8.0 7.5 7.0 6.5 6.0 5.5 5.0 4.5 4.0 3.5 3.0 2.5 2.0 1.5 1.0 0.5 0.0 -0
f1 (ppm)

(*Z*)-3ah

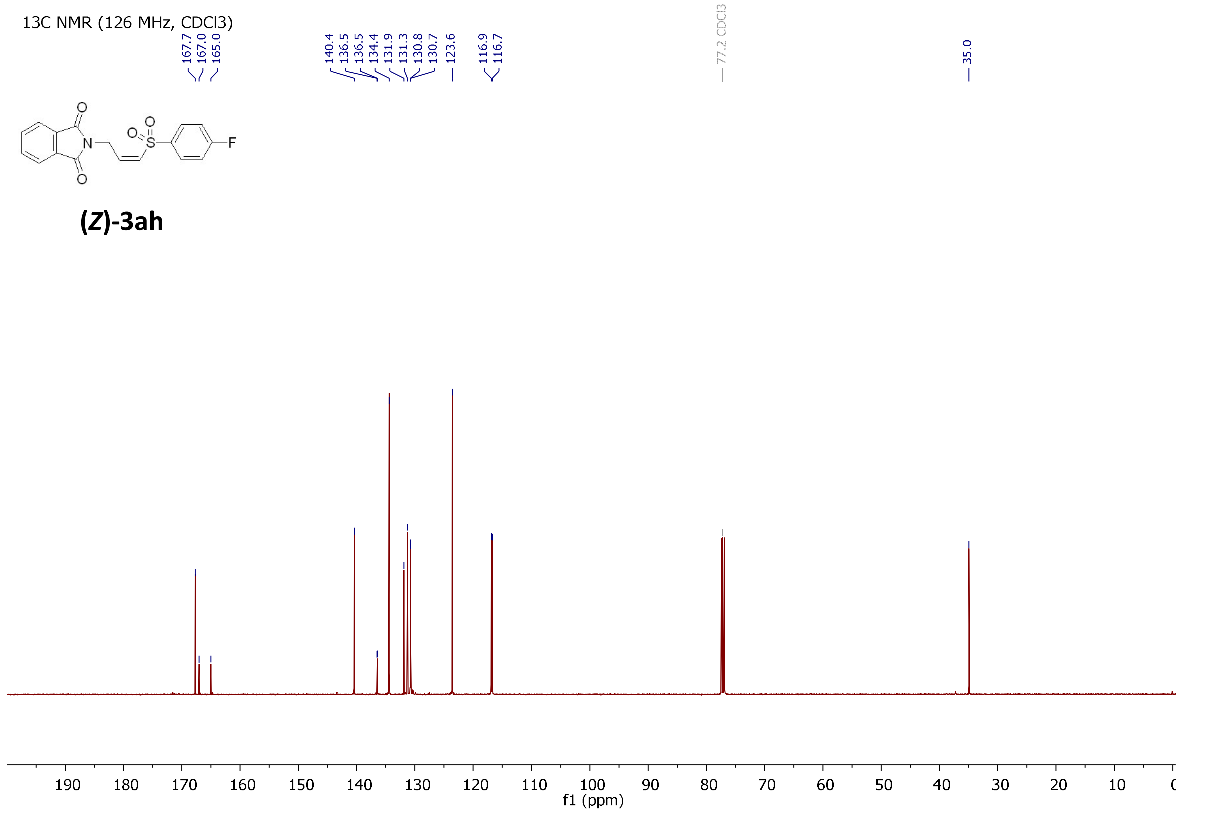

13C NMR (126 MHz, CDCl3)
167.7
167.0
165.0
140.4
136.5
136.5
134.4
131.9
131.3
130.8
130.7
123.6
116.9
116.7
77.2 CDCl3
35.0
O
O
N
O
O
S
F
190 180 170 160 150 140 130 120 110 100 90 80 70 60 50 40 30 20 10 0
f1 (ppm)

(*Z*)-3ah

19F NMR (471 MHz, CDCl3)

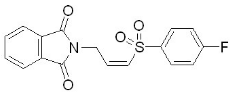

(*Z*)-3ah

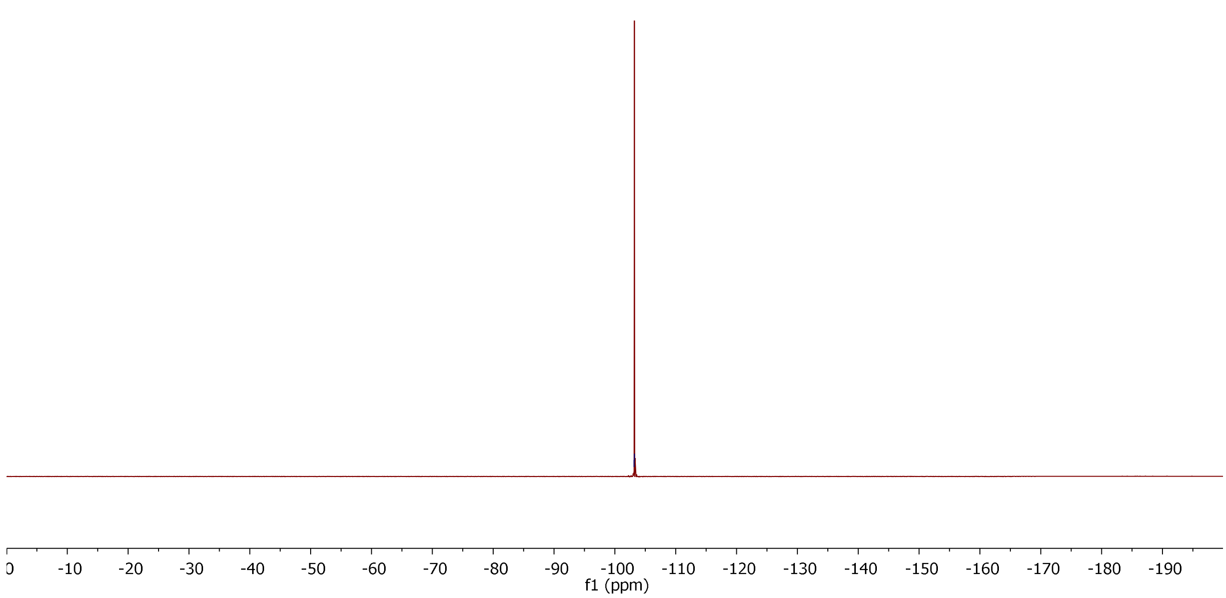

1H NMR (500 MHz, CDCl3)

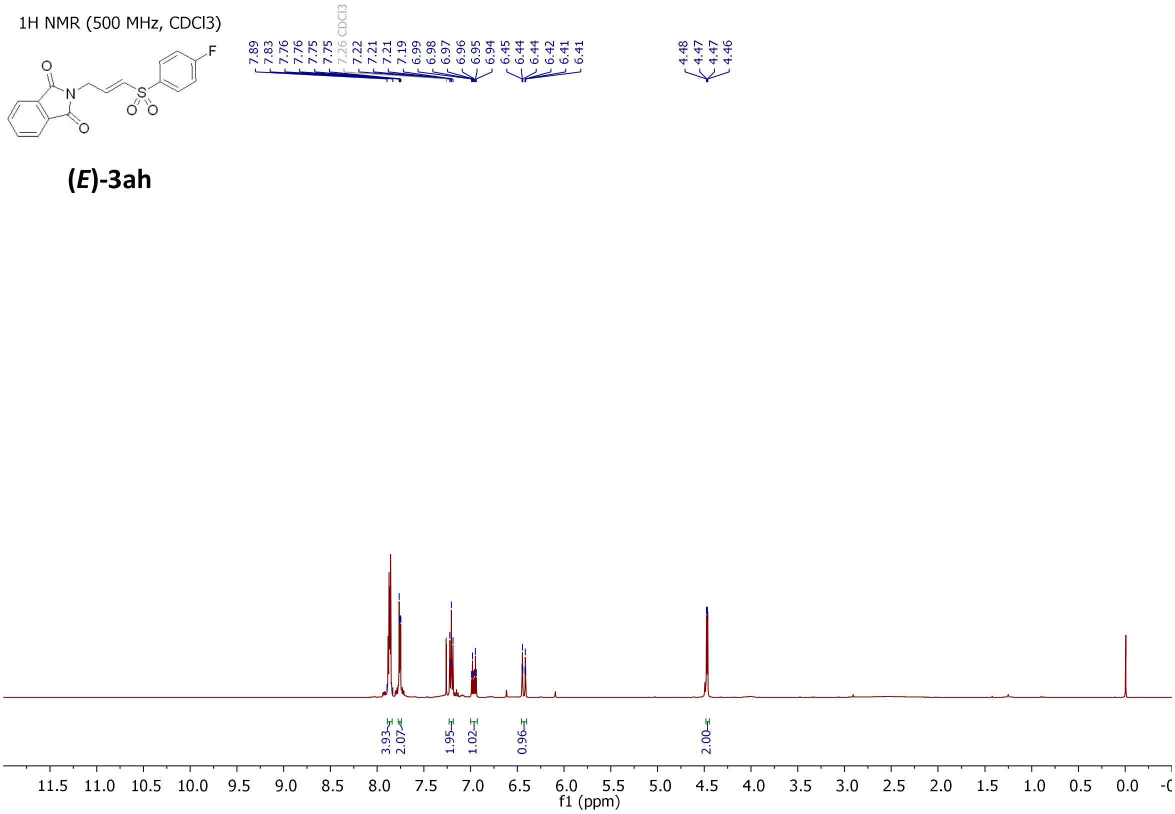

(*E*)-3ah

13C NMR (126 MHz, CDCl3)

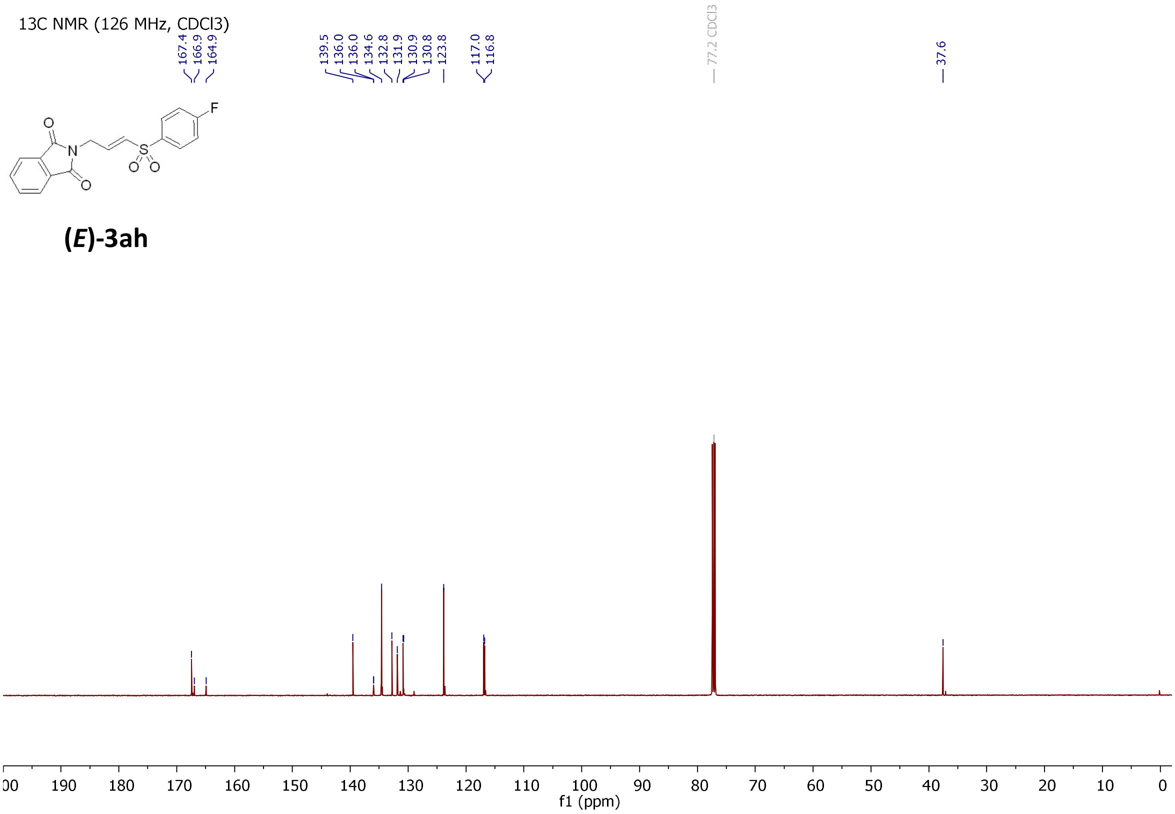

(*E*)-3ah

19F NMR (471 MHz, CDCl3)

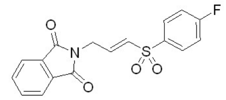

(*E*)-3ah

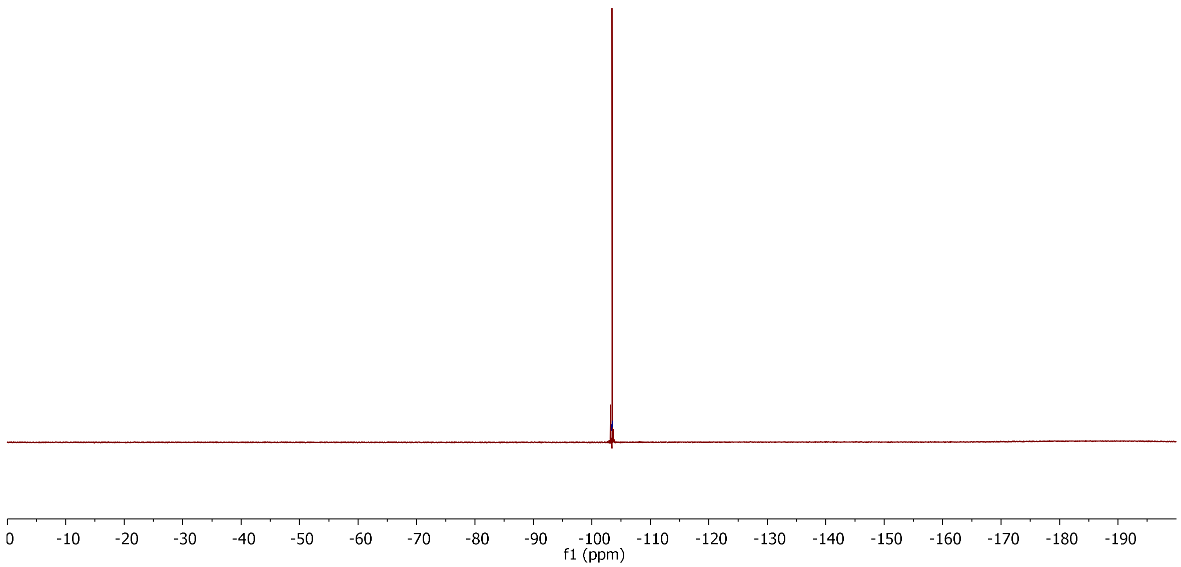

1H NMR (500 MHz, CDCl3)

3ai

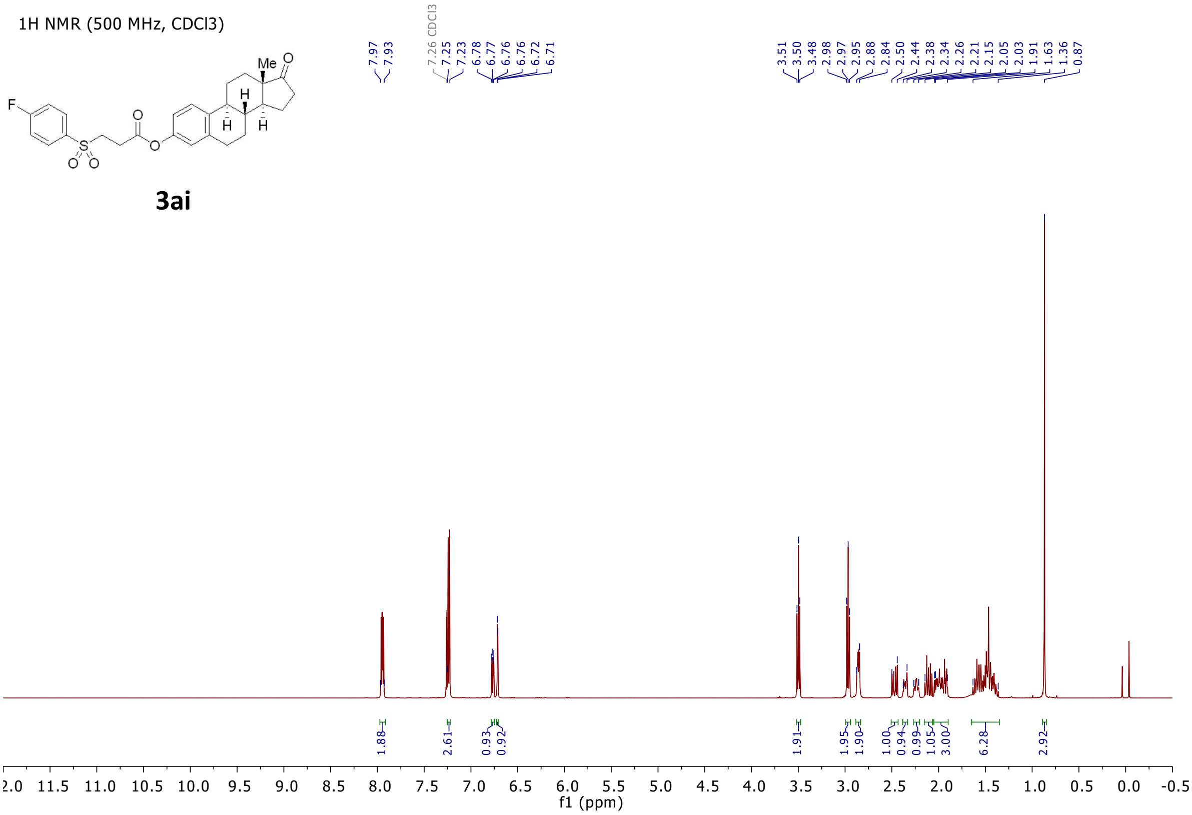

13C NMR (126 MHz, CDCl3)

3ai

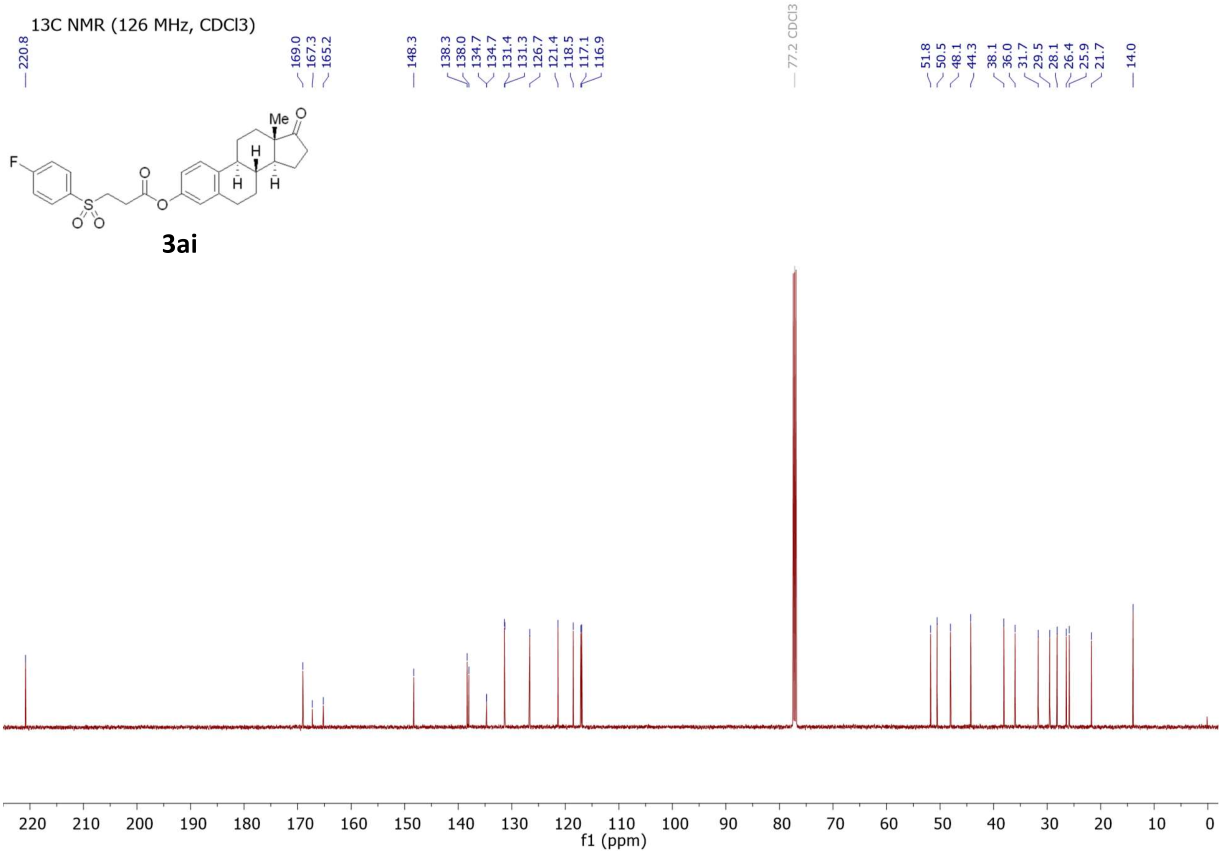

19F NMR (471 MHz, CDCl3)

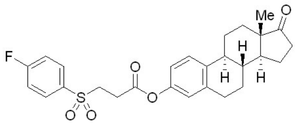

3ai

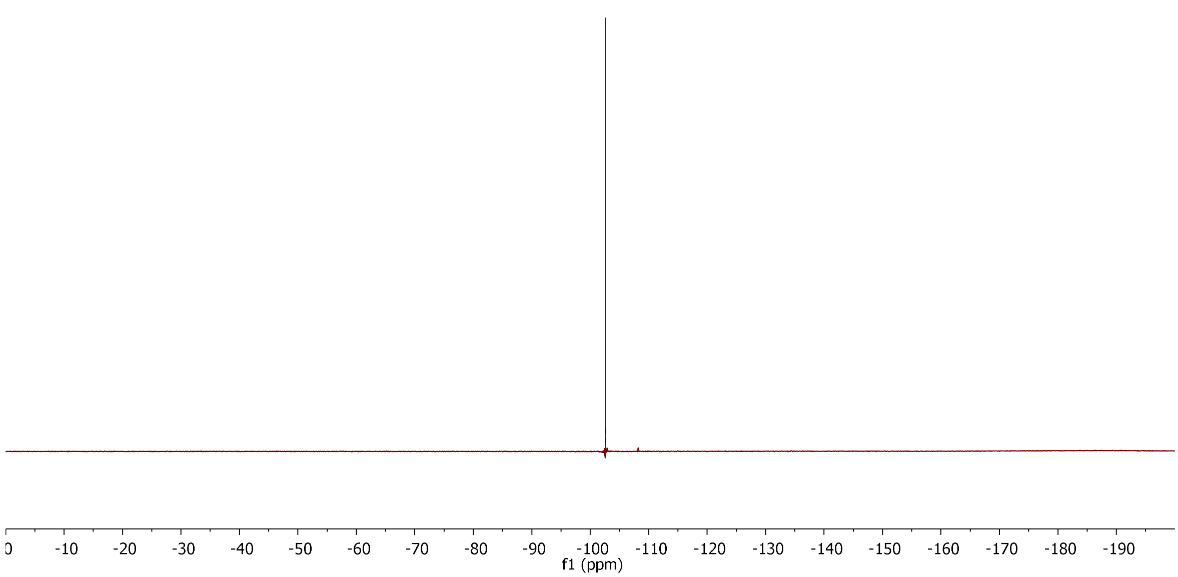

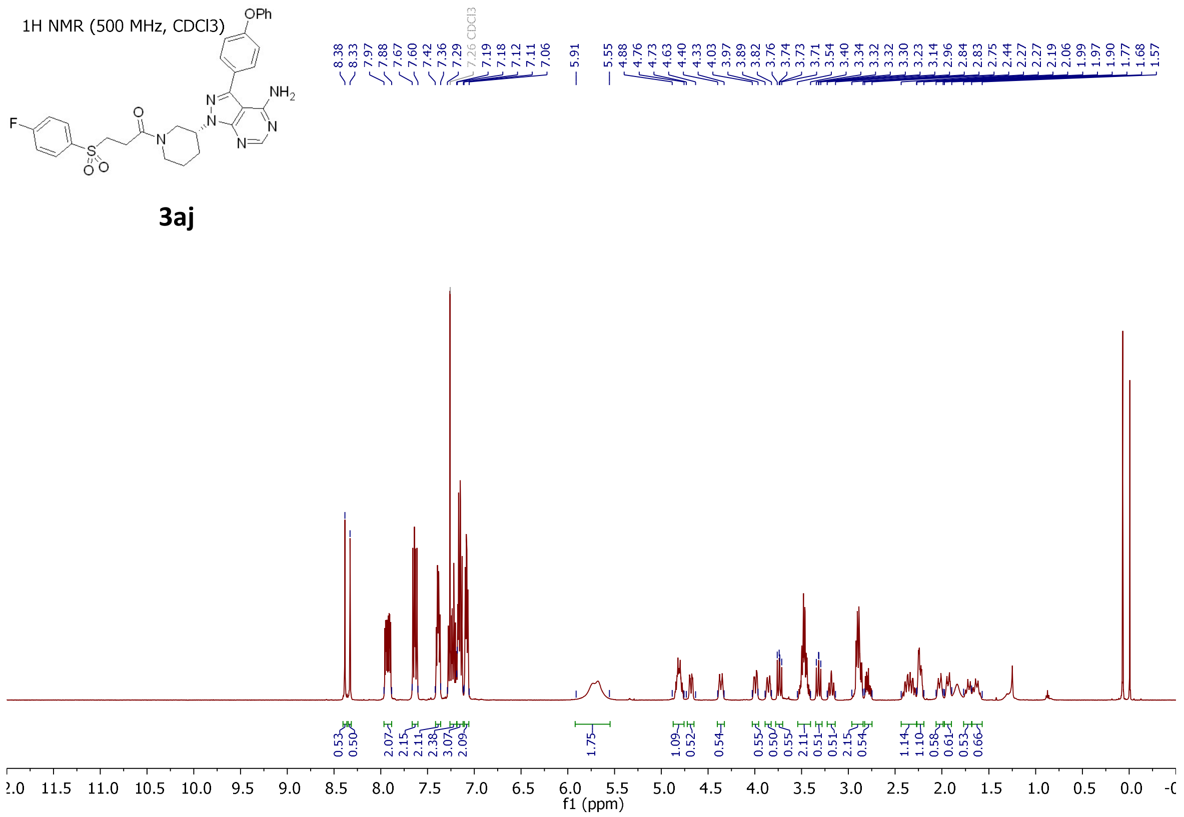

3aj

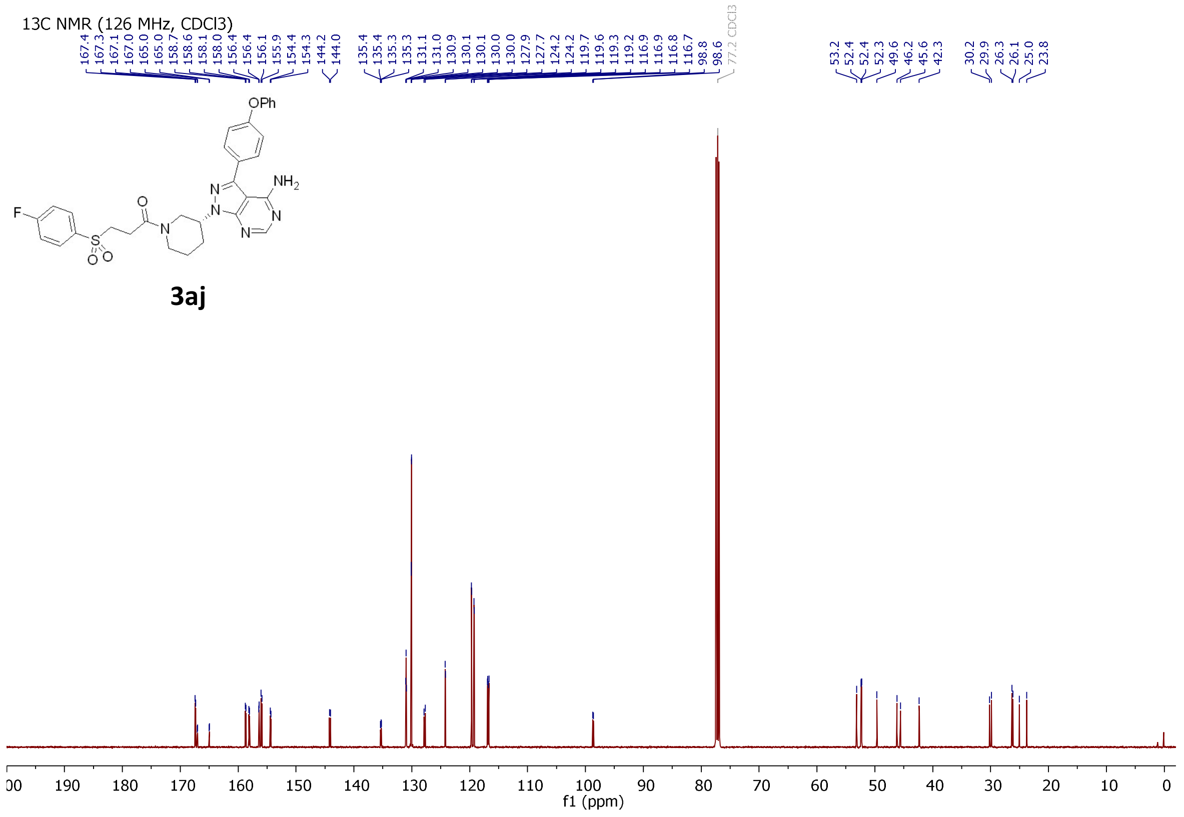

3aj

19F NMR (471 MHz, CDCl3)

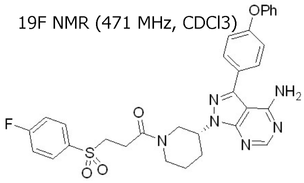

3aj

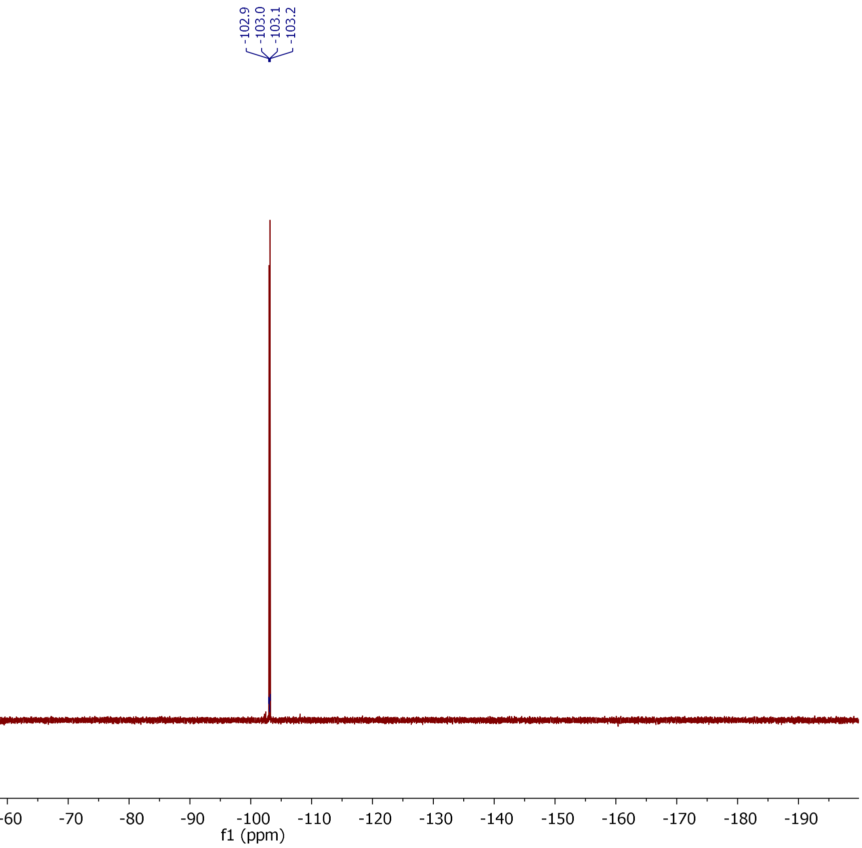

1H NMR (500 MHz, CDCl3)

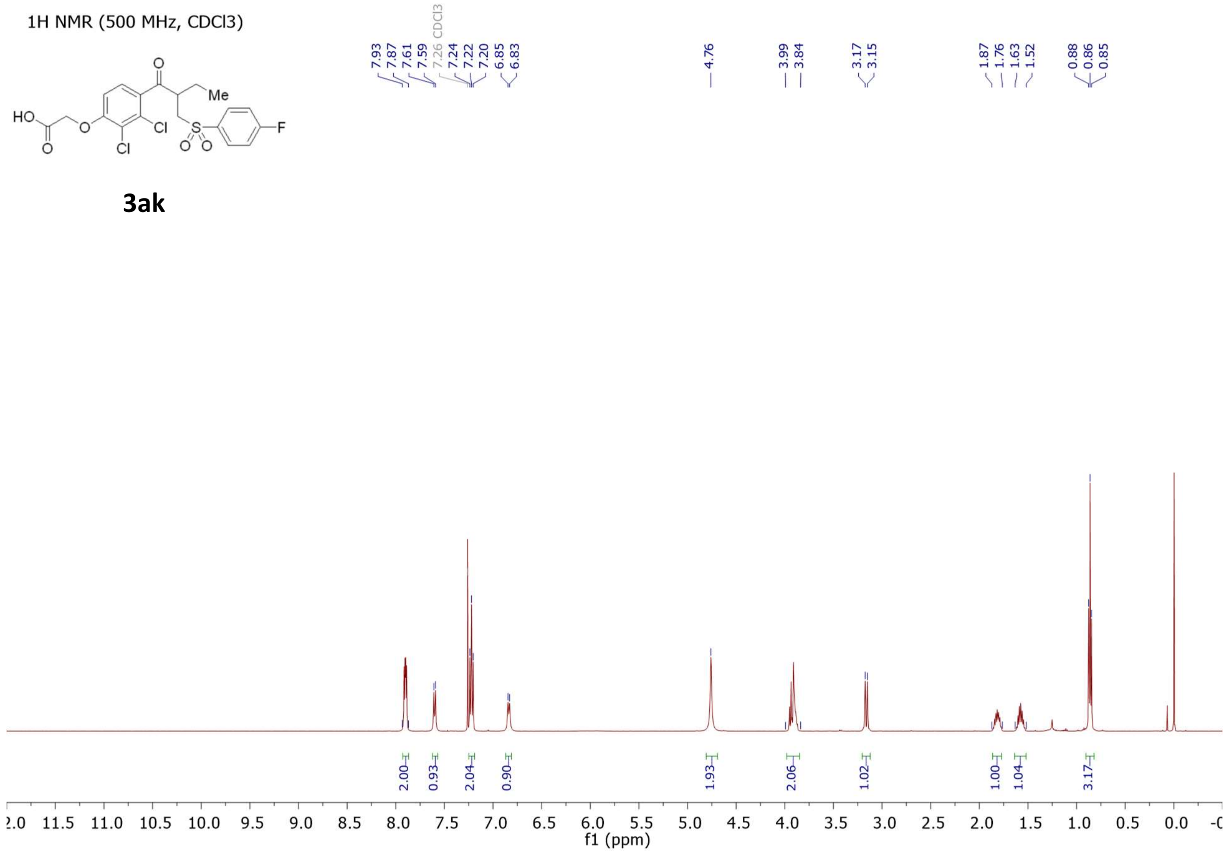

3ak

13C NMR (101 MHz, CDCl3)

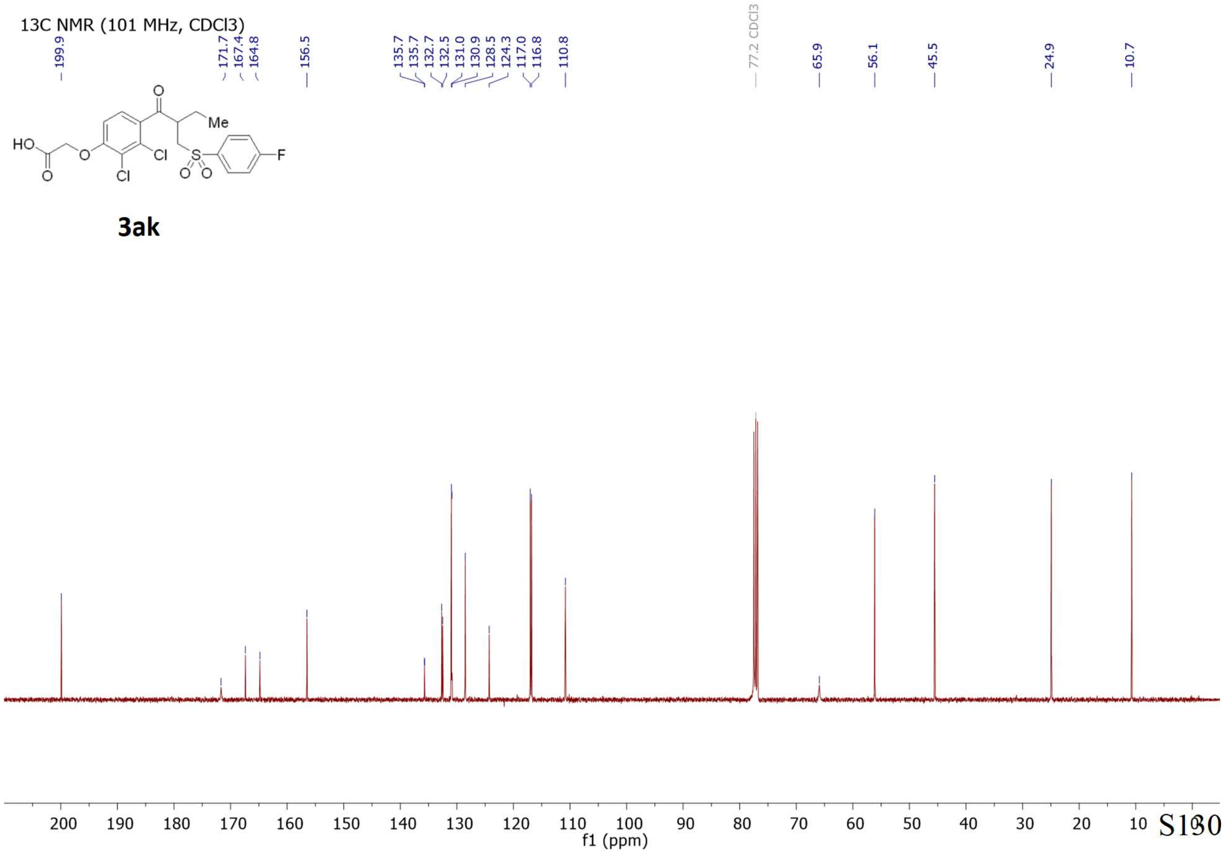

3ak

19F NMR (471 MHz, CDCl3)

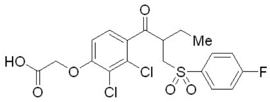

3ak

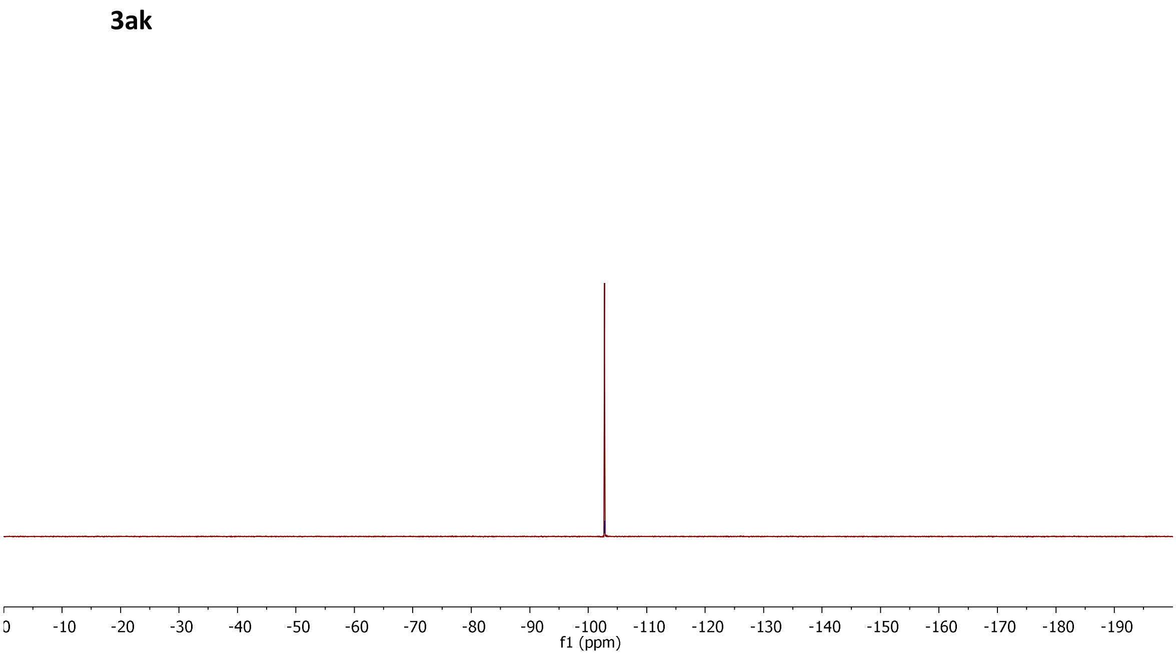

1H NMR (400 MHz, CDCl3)

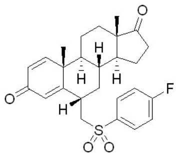

3al

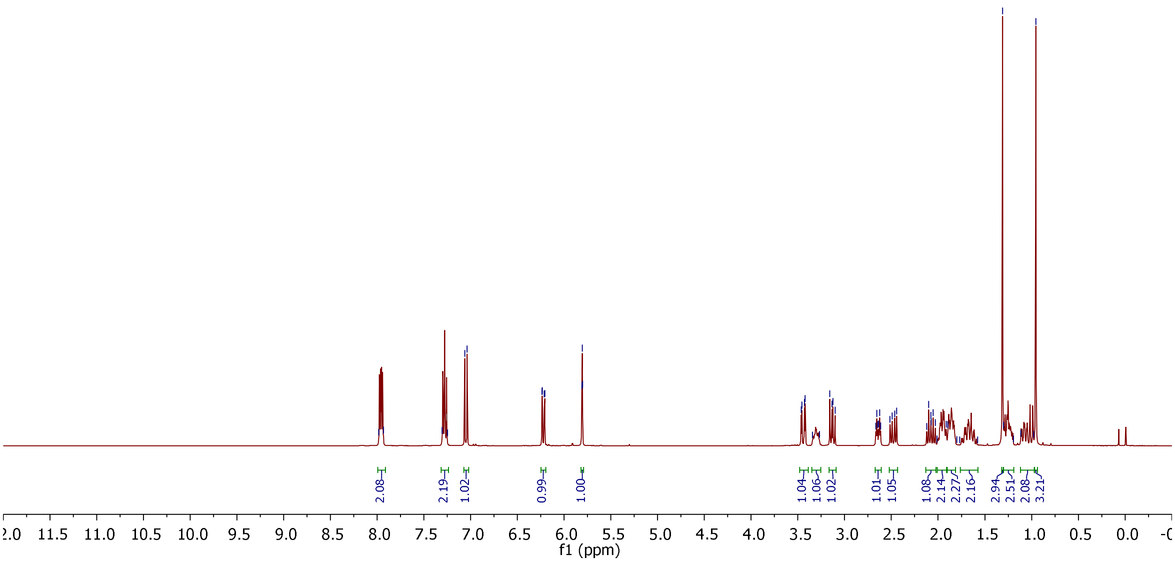

13C NMR (126 MHz, CDCl3)

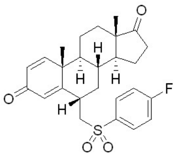

3al

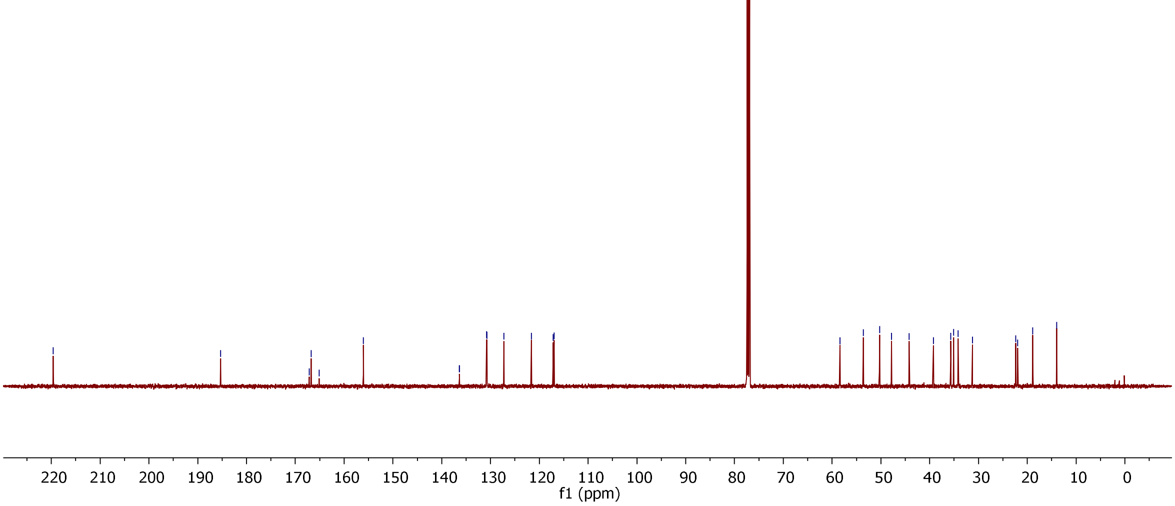

19F NMR (471 MHz, CDCl3)

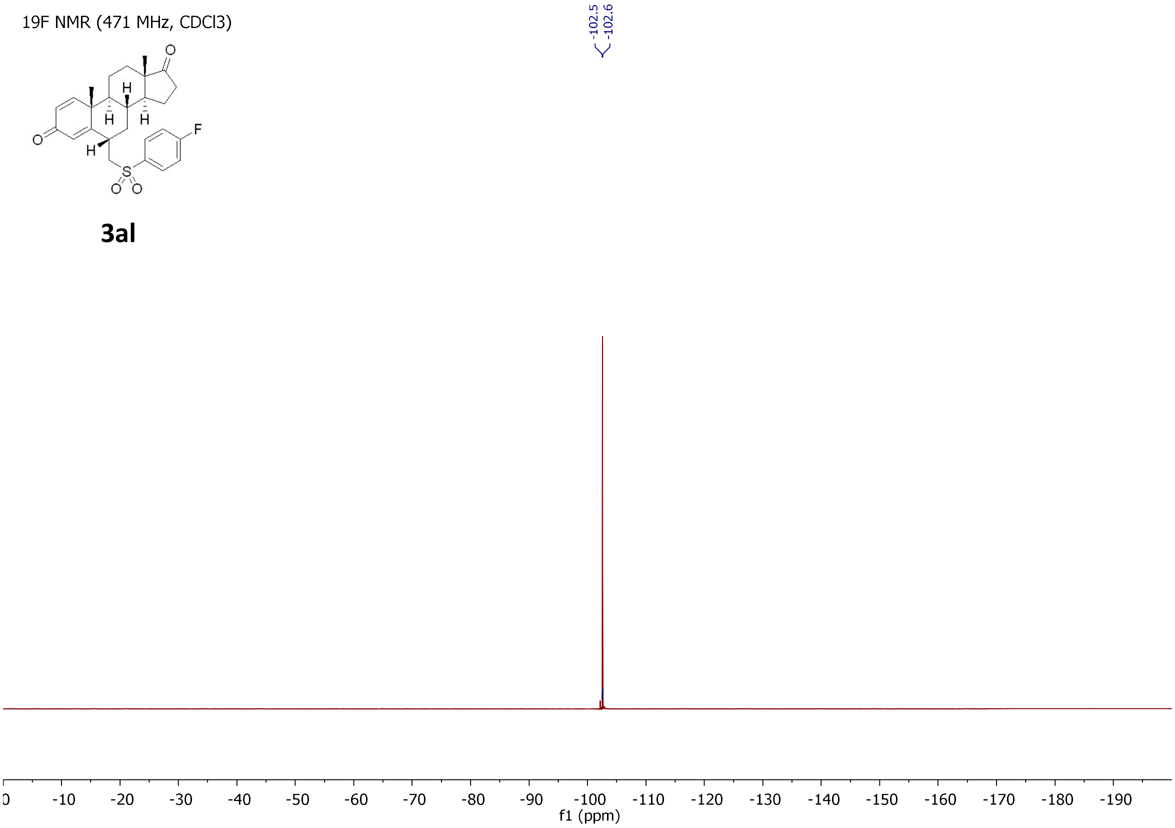

3al

NOESY (400 MHz, CDCl3)

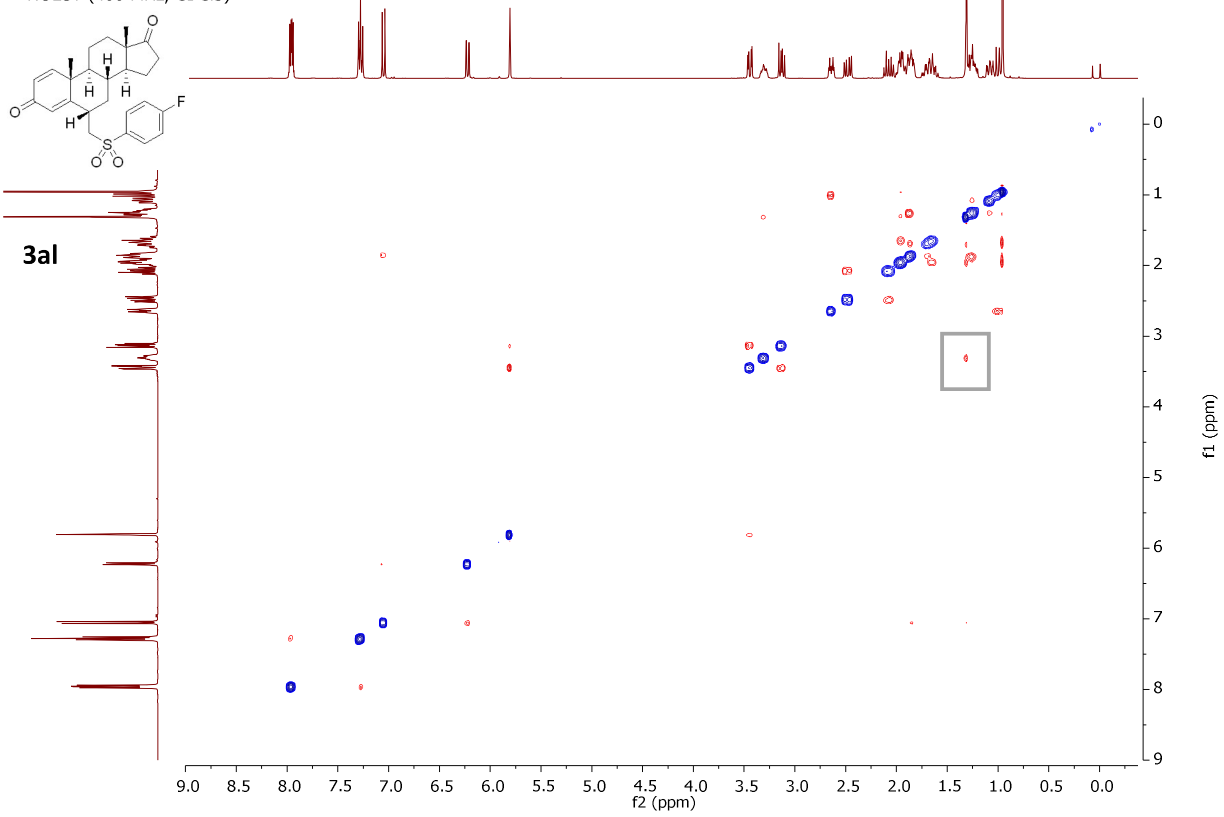

3al

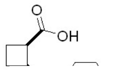

1H NMR (400 MHz, DMSO-d6)

3am

3am

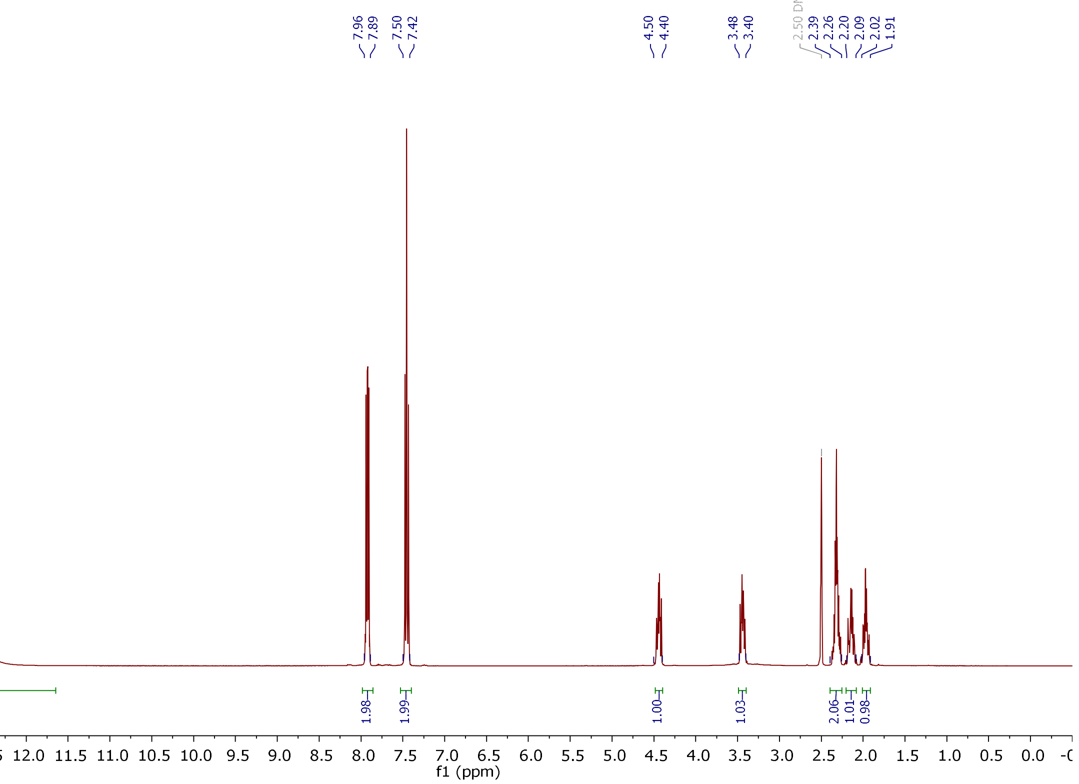

13C NMR (101 MHz, DMSO)

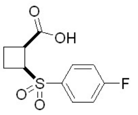

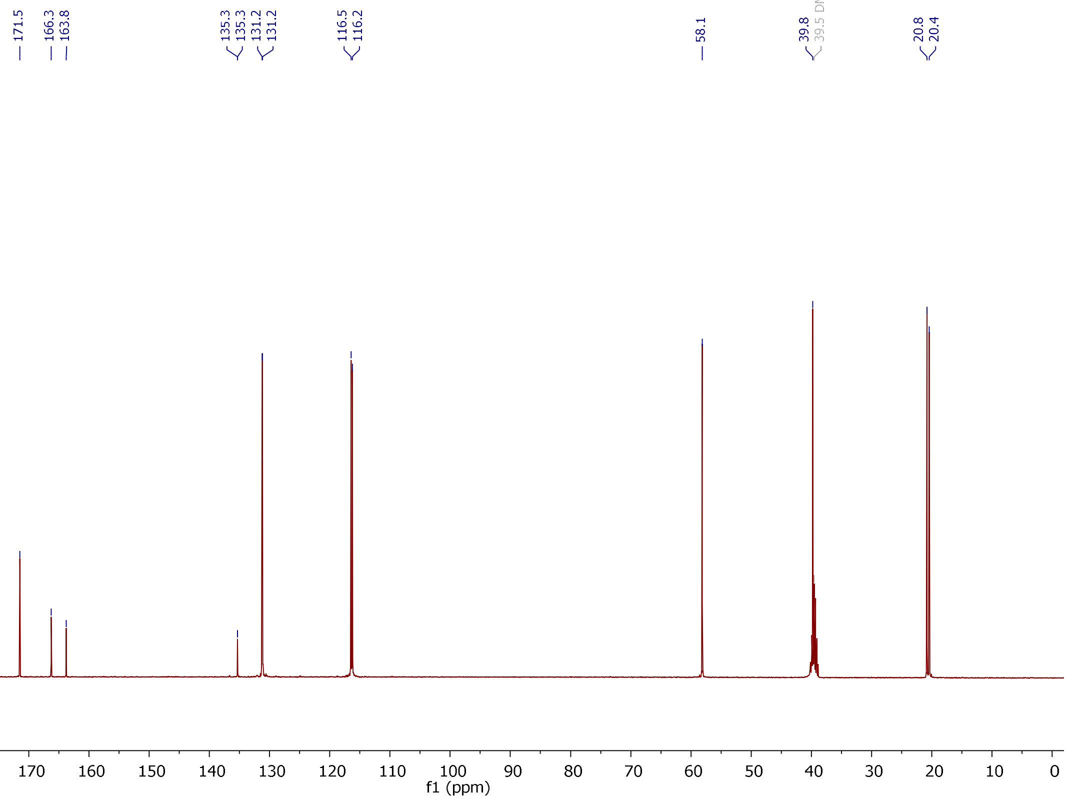

19F NMR (471 MHz, DMSO-d6)

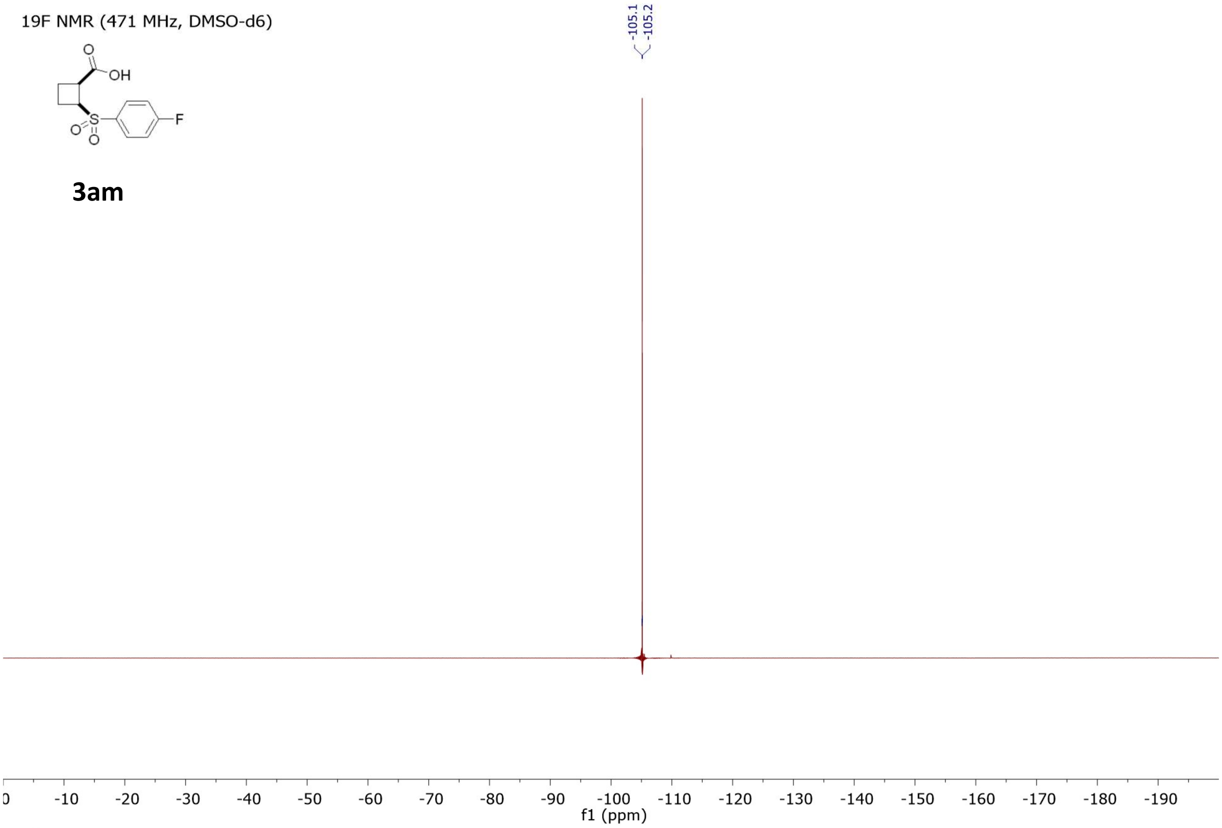

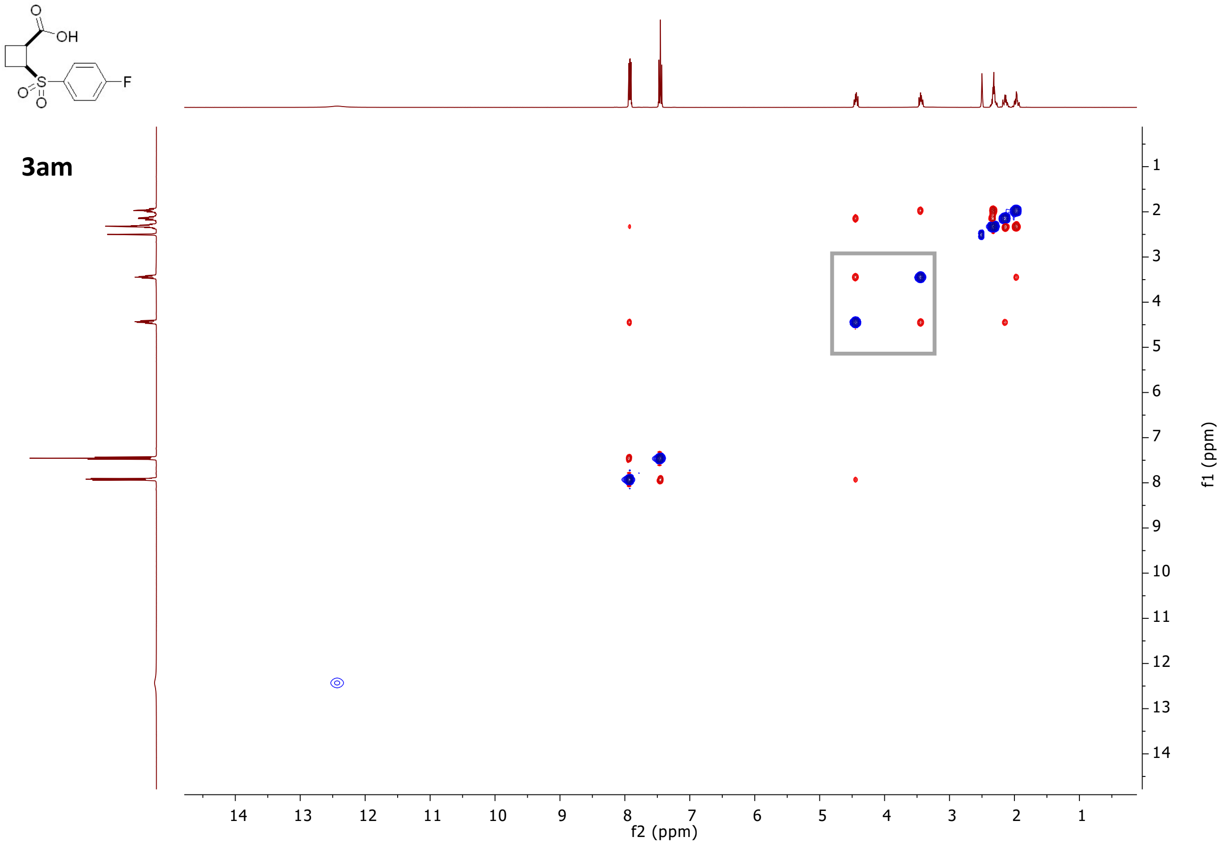

1H NMR (400 MHz, CDCl3)

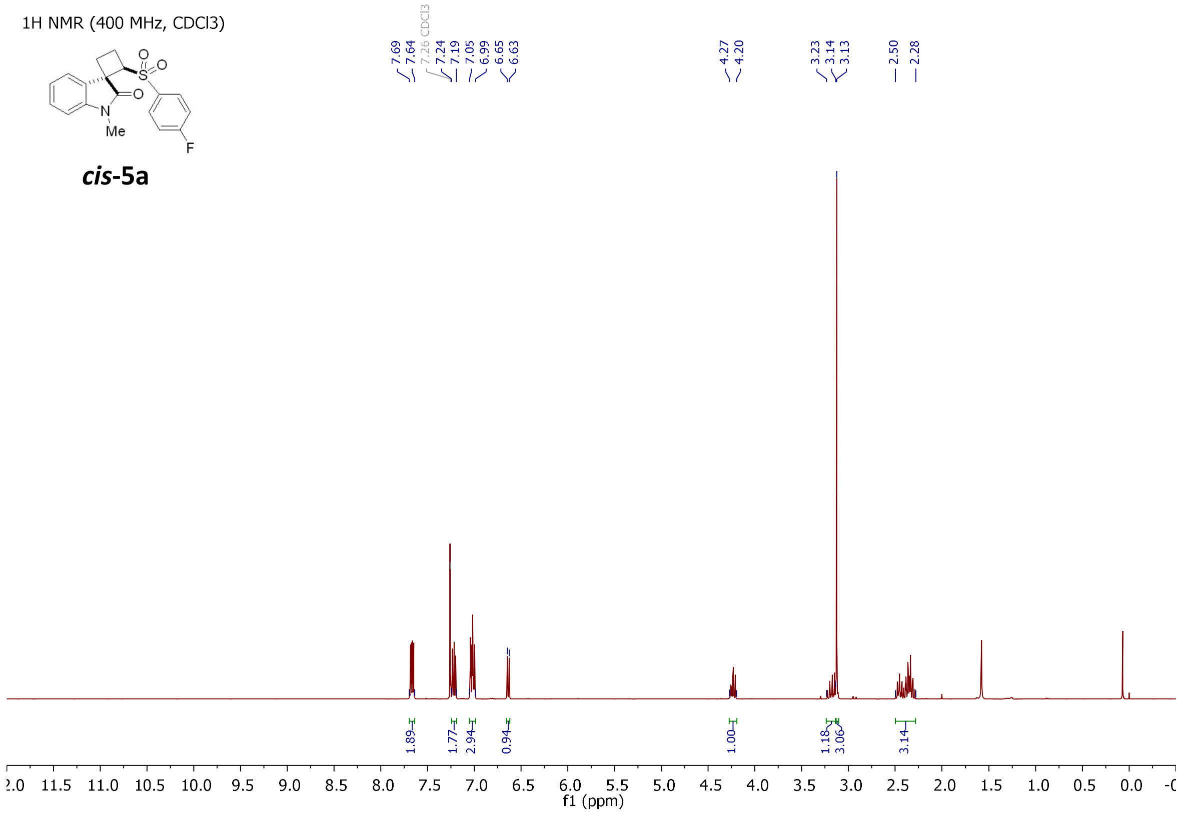

13C NMR (101 MHz, CDCl3)

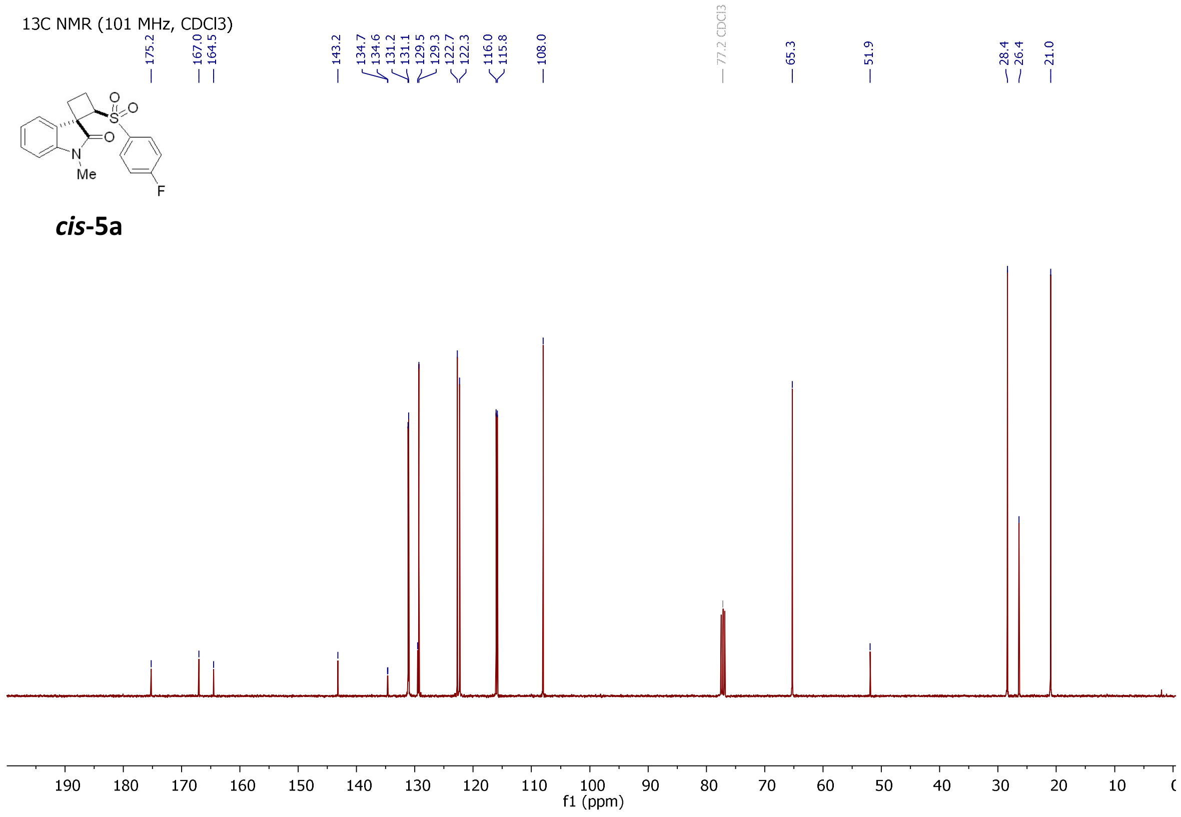

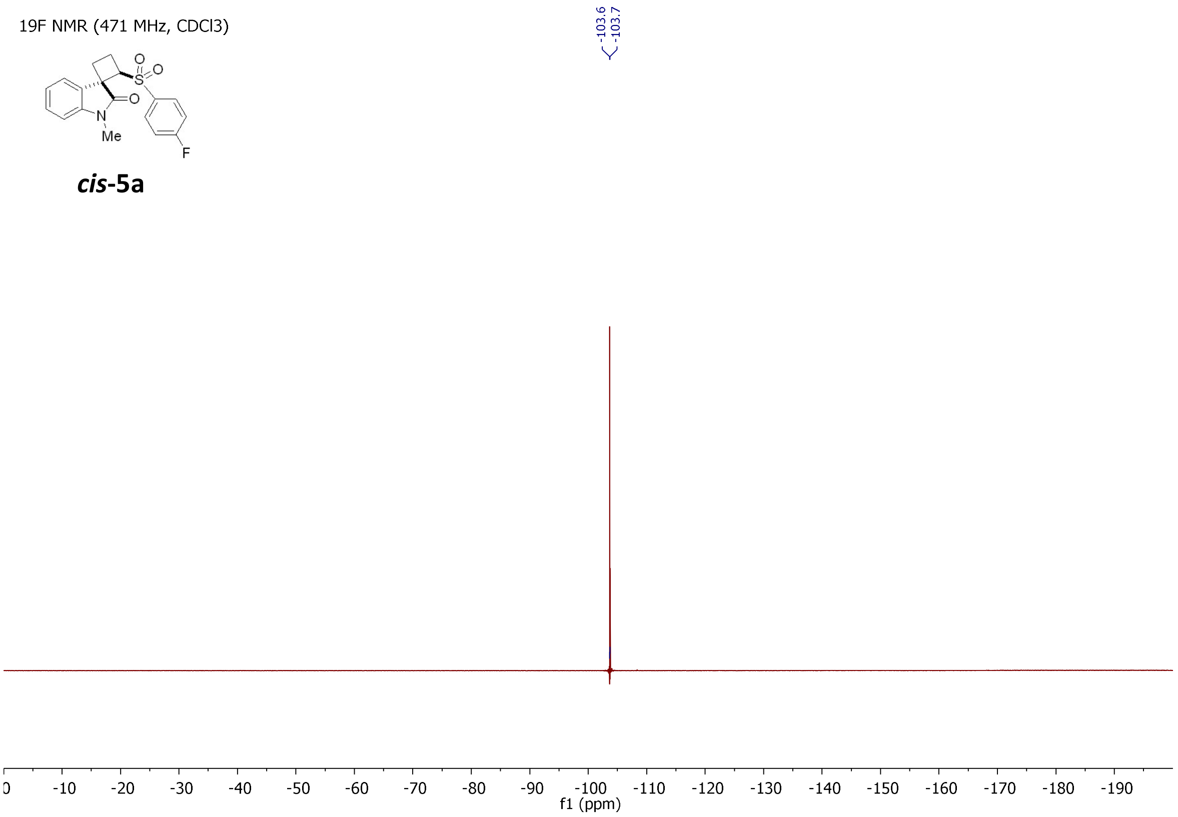
19F NMR (471 MHz, CDCl3)
cis-5a
-103.6
-103.7
f1 (ppm)

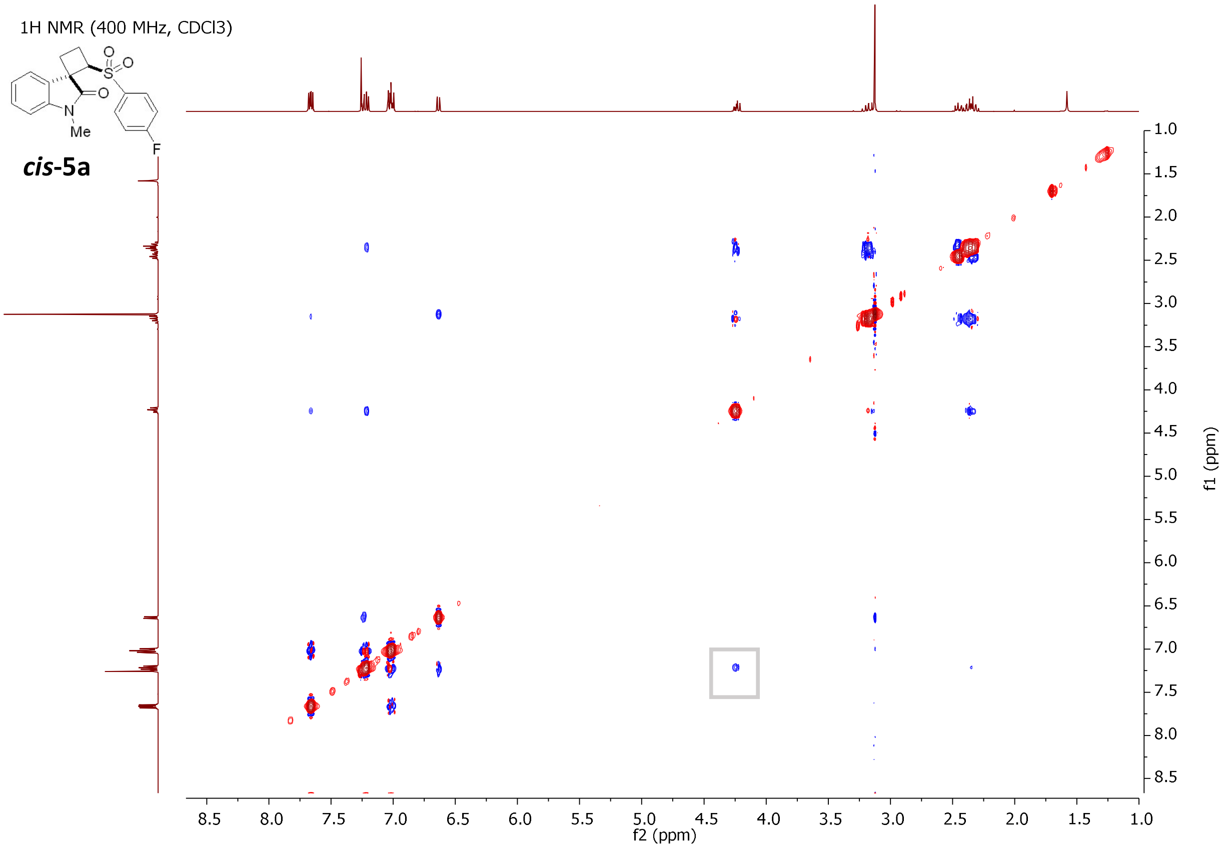
1H NMR (400 MHz, CDCl3)
cis-5a
f2 (ppm)
f1 (ppm)

1H NMR (400 MHz, CDCl3)

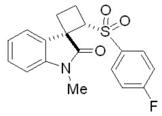

***trans*-5a**

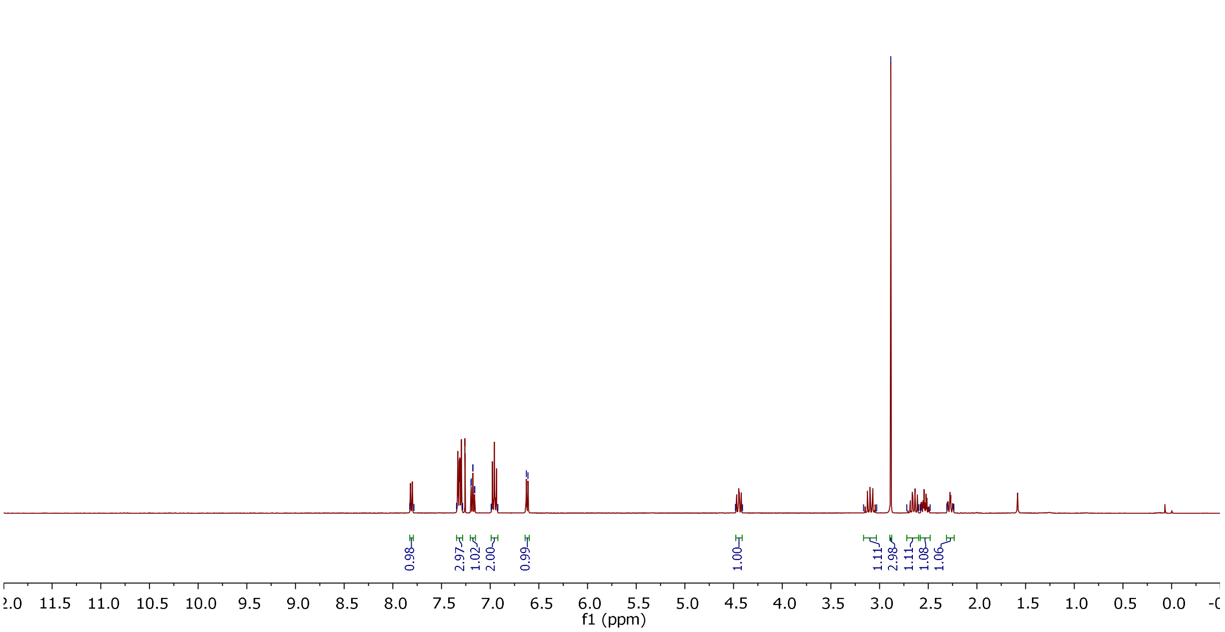

13C NMR (101 MHz, CDCl3)

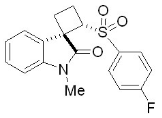

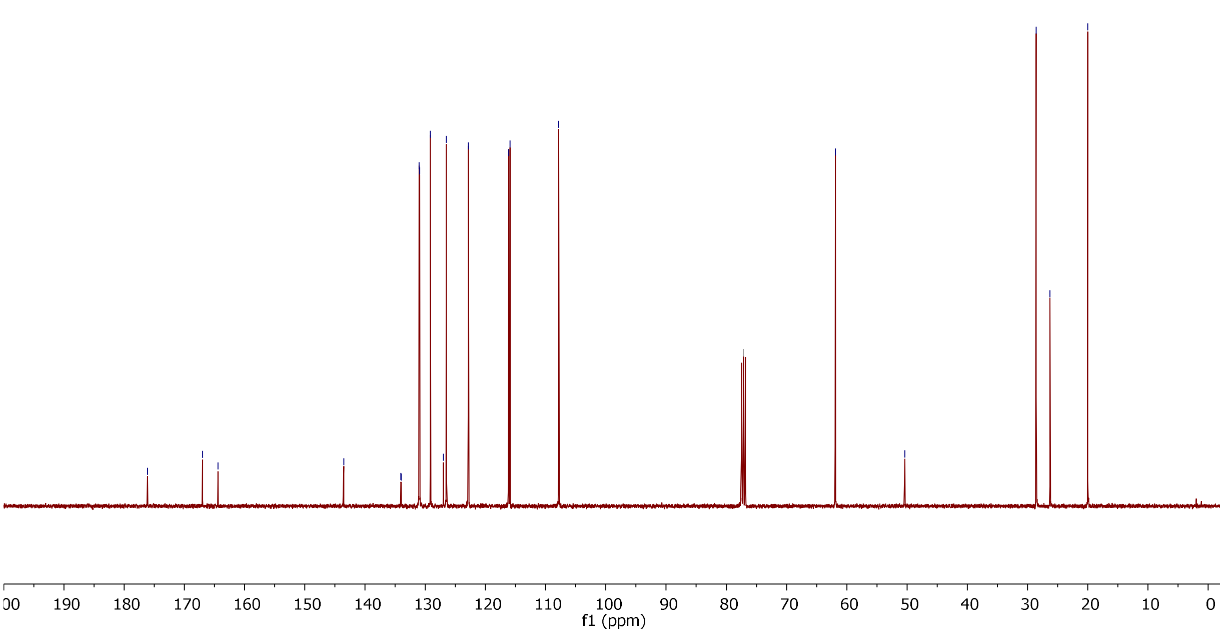

19F NMR (471 MHz, CDCl3)

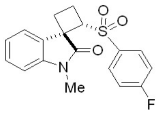

trans-5a

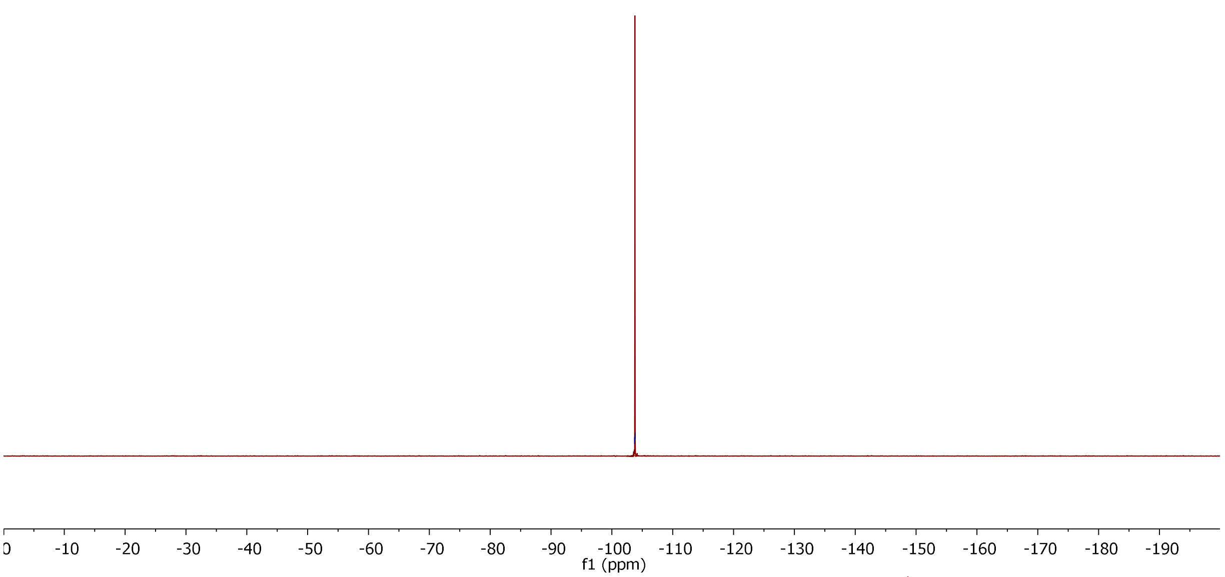

NOESY (400 MHz, CDCl3)

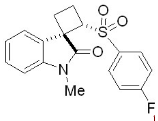

trans-5a

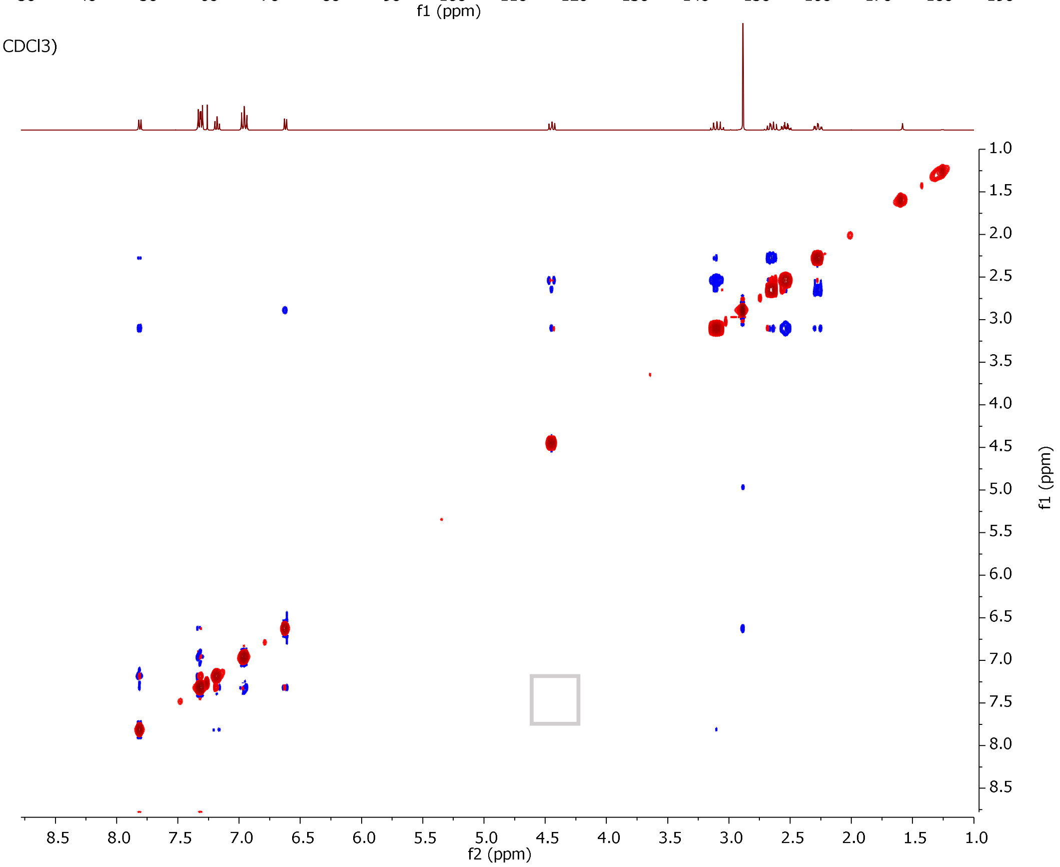

1H NMR (400 MHz, CDCl3)

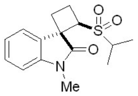

***cis*-5b**

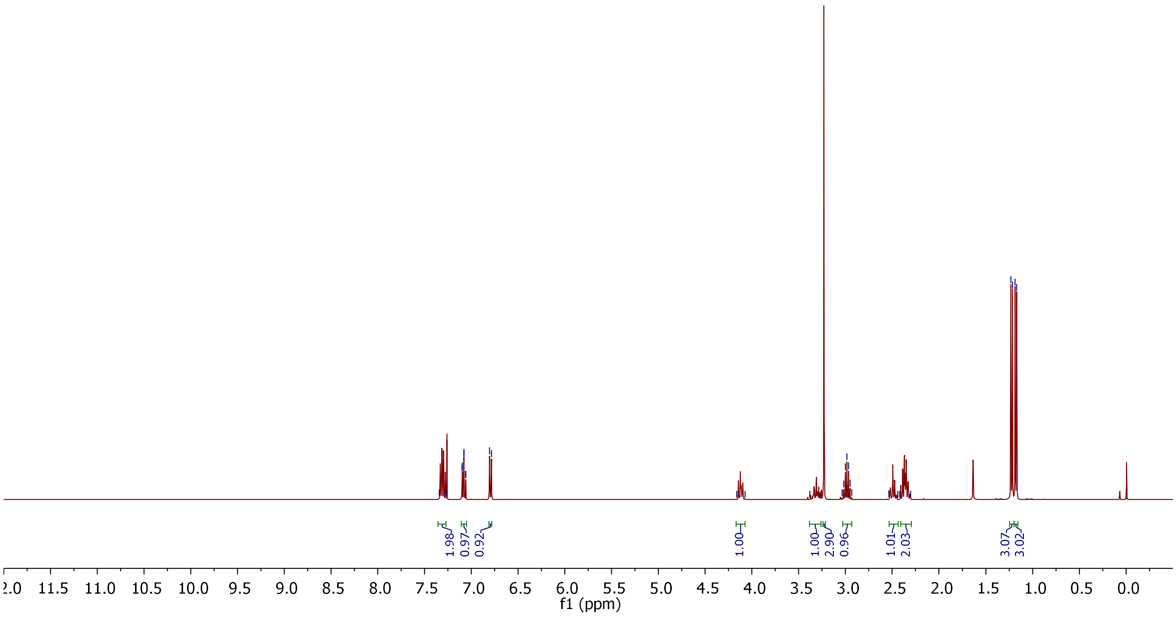

13C NMR (101 MHz, CDCl3)

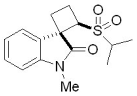

***cis*-5b**

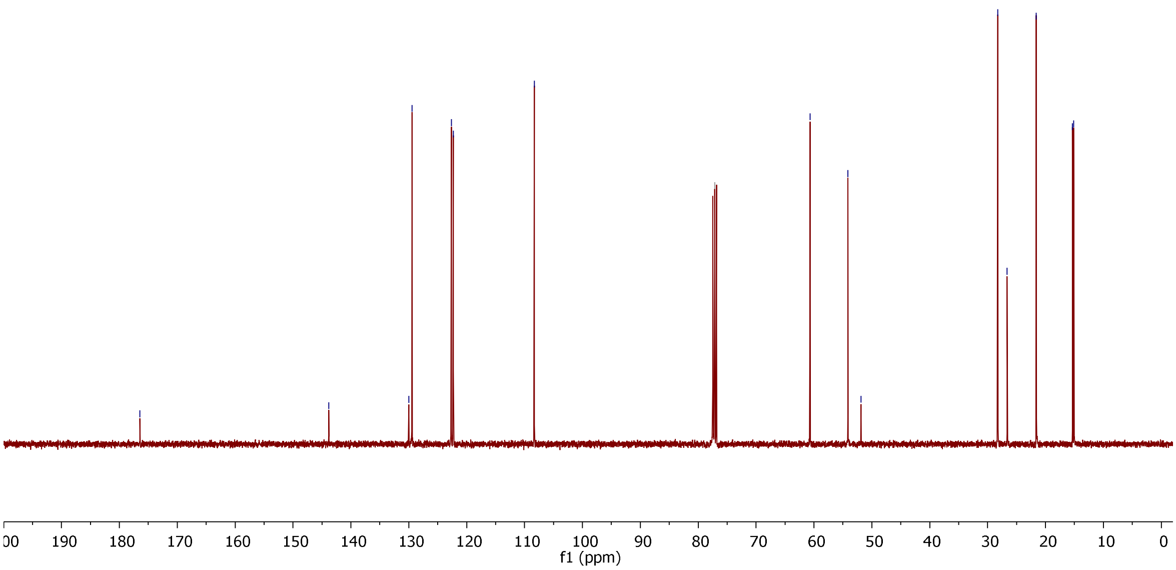

1H NMR (400 MHz, CDCl3)

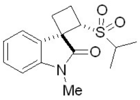

cis-**5b**

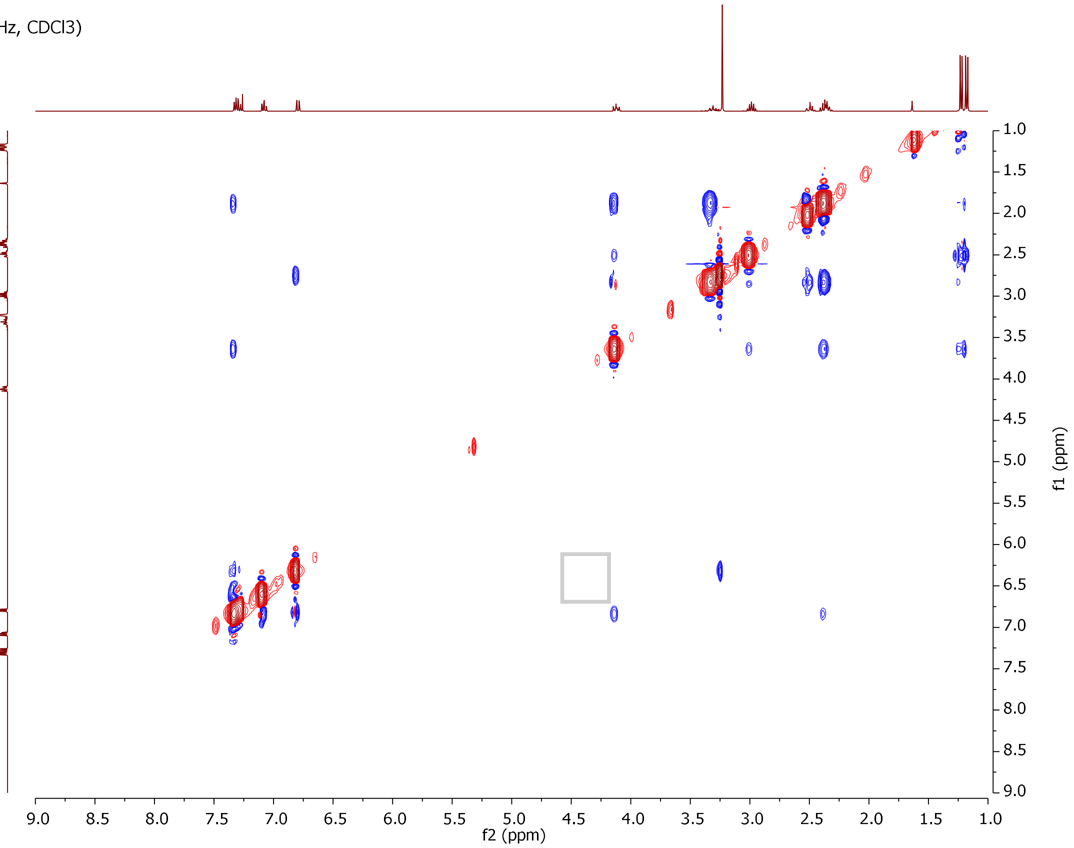

1H NMR (400 MHz, CDCl3)

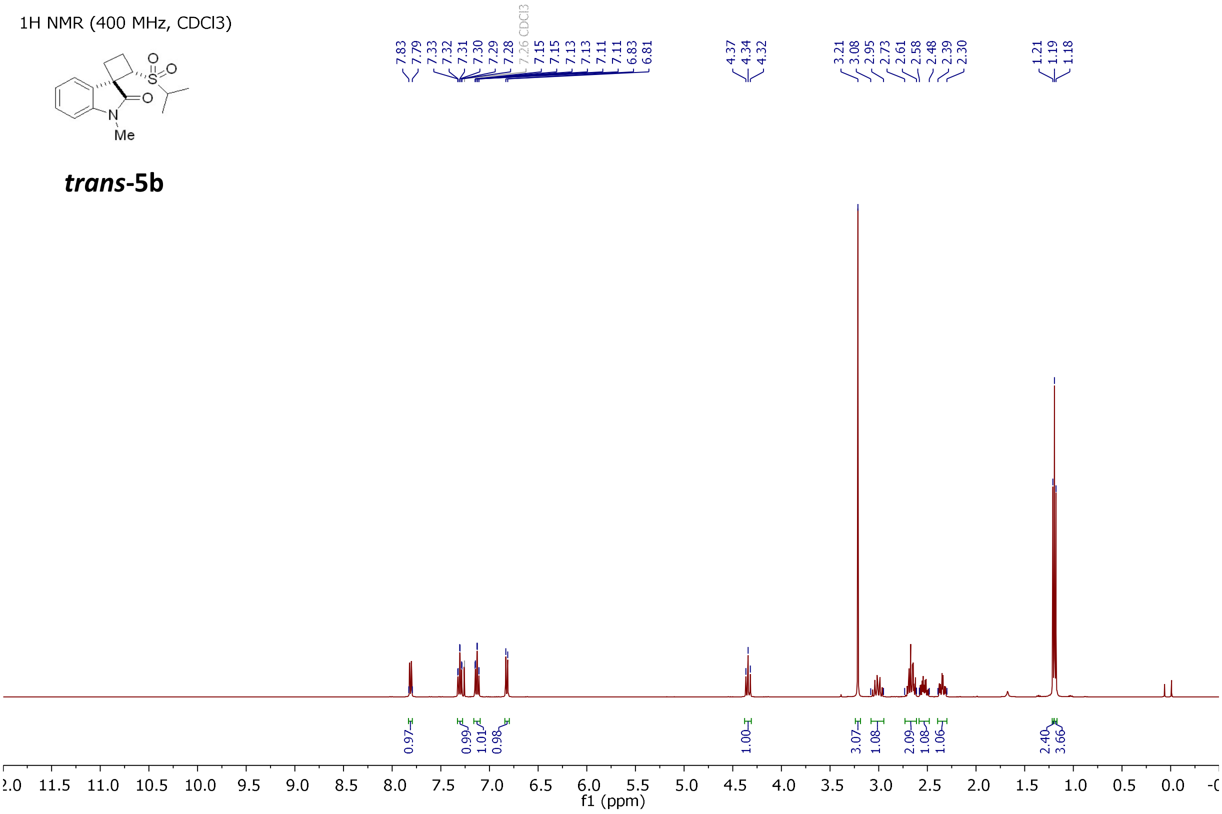

***trans*-5b**

13C NMR (101 MHz, CDCl3)

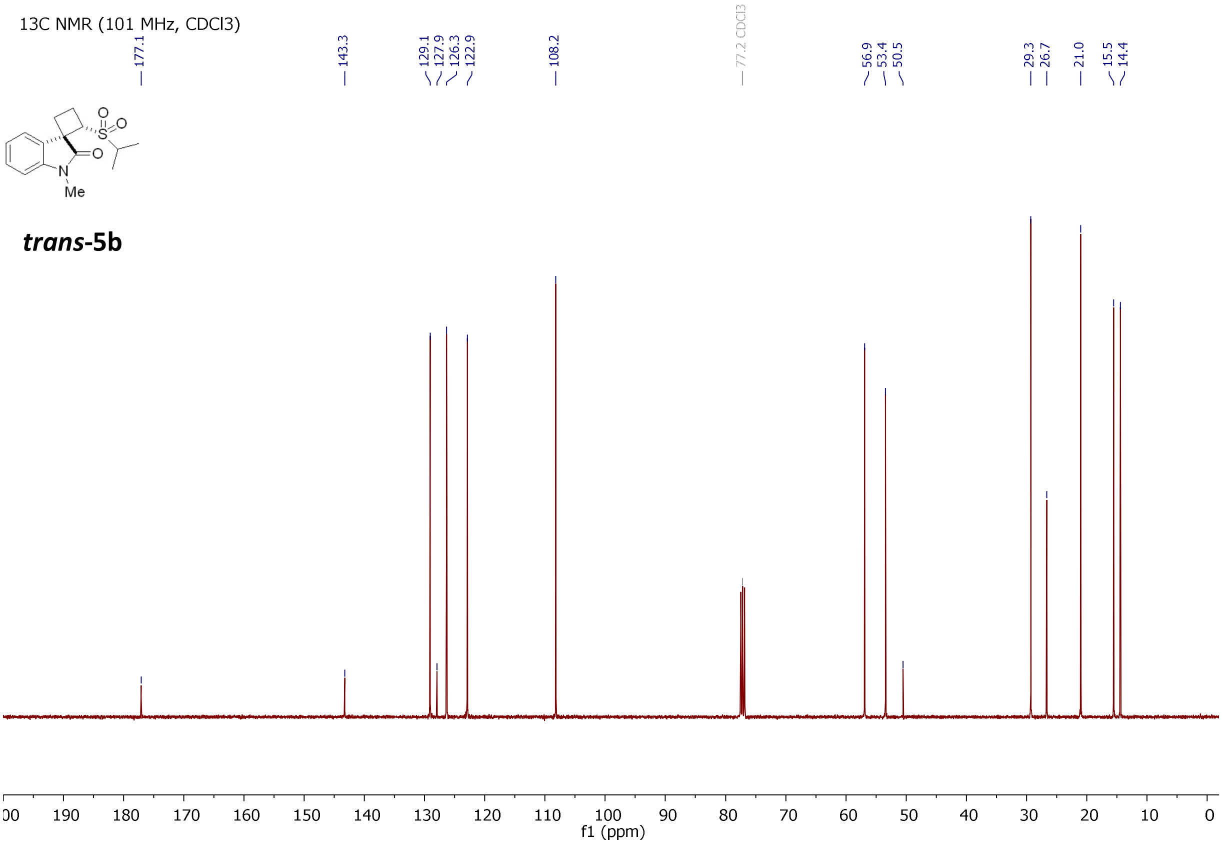

***trans*-5b**

1H NMR (400 MHz, CDCl3)

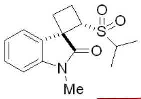

***trans*-5b**

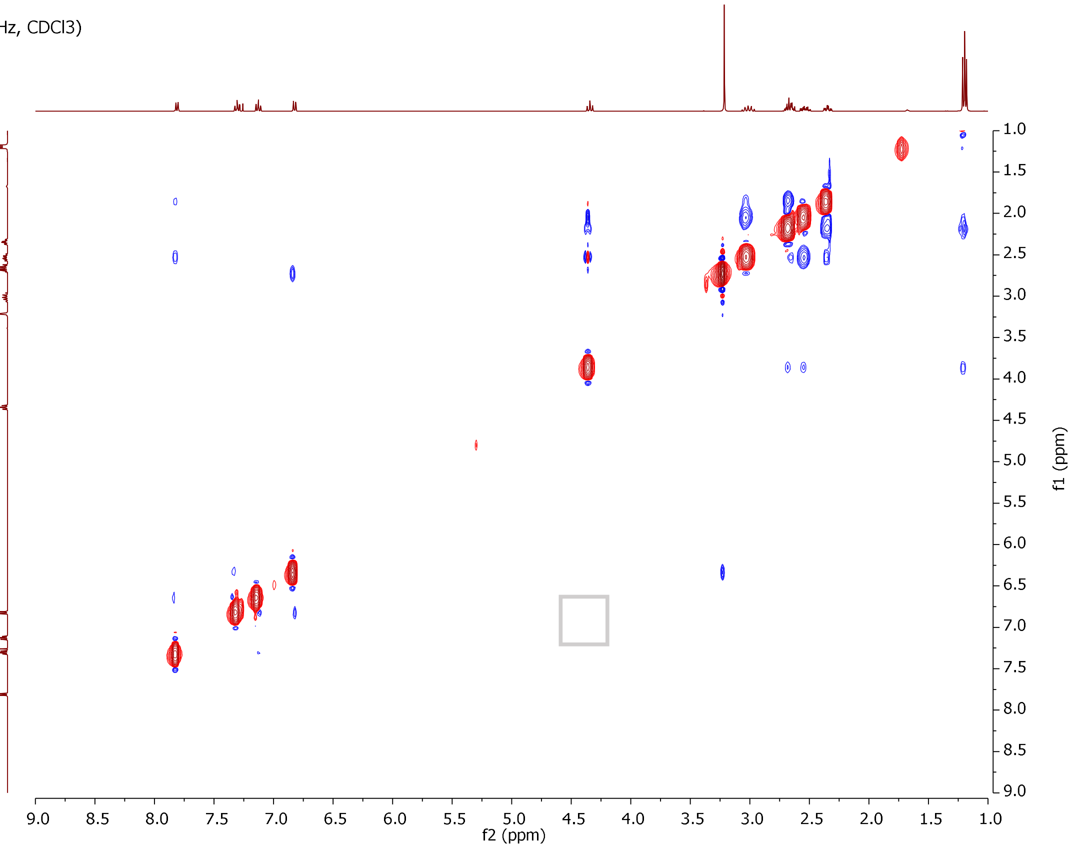

1H NMR (500 MHz, CDCl3)

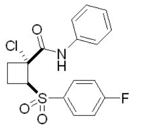

6

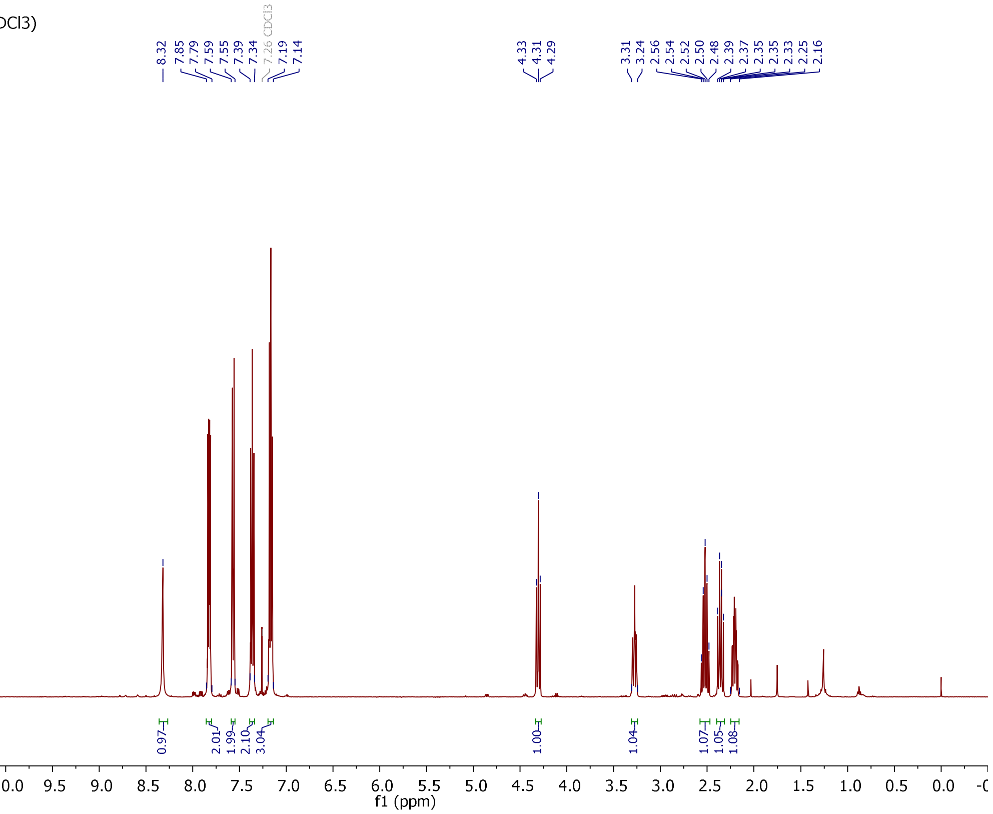

13C NMR (126 MHz, CDCl3)

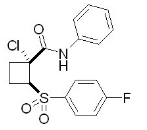

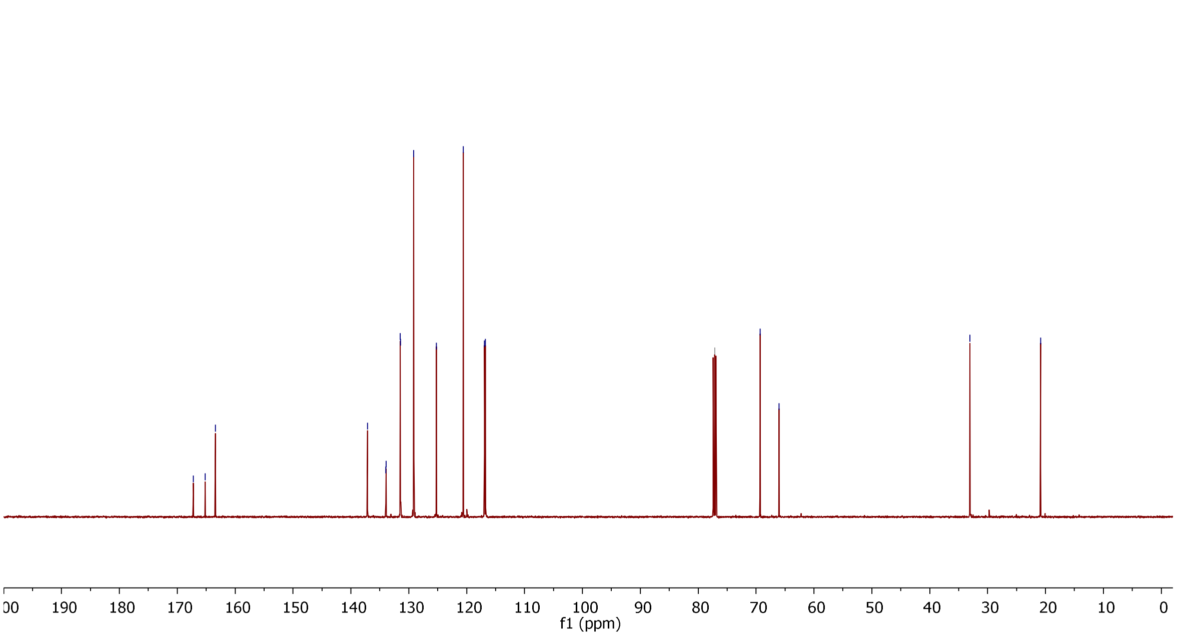

19F NMR (471 MHz, CDCl3)

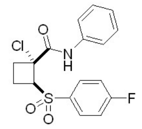

6

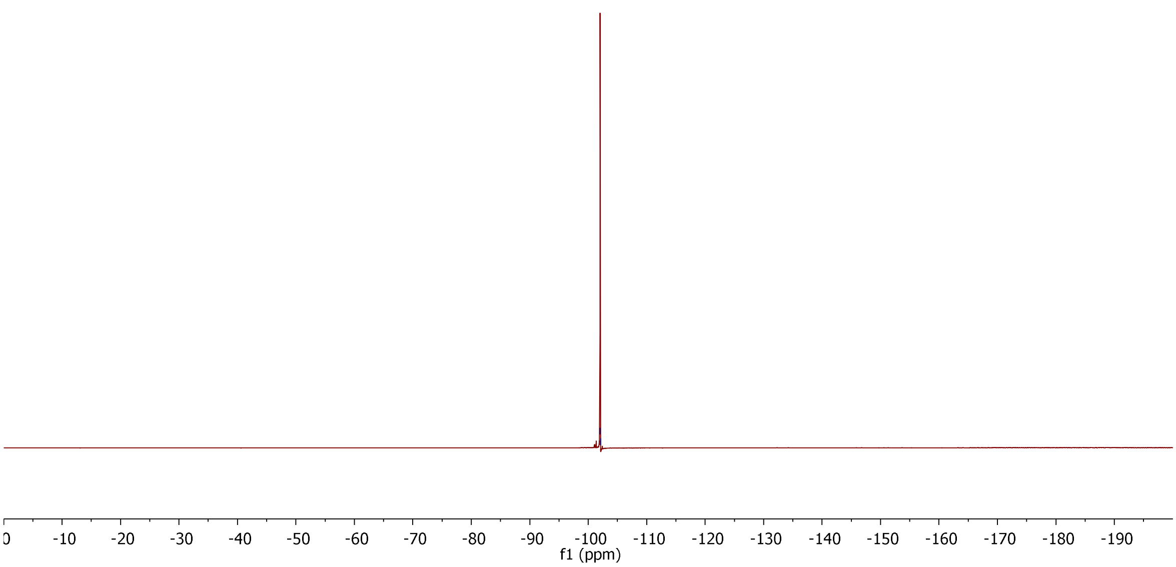

1H NMR (400 MHz, CDCl3)

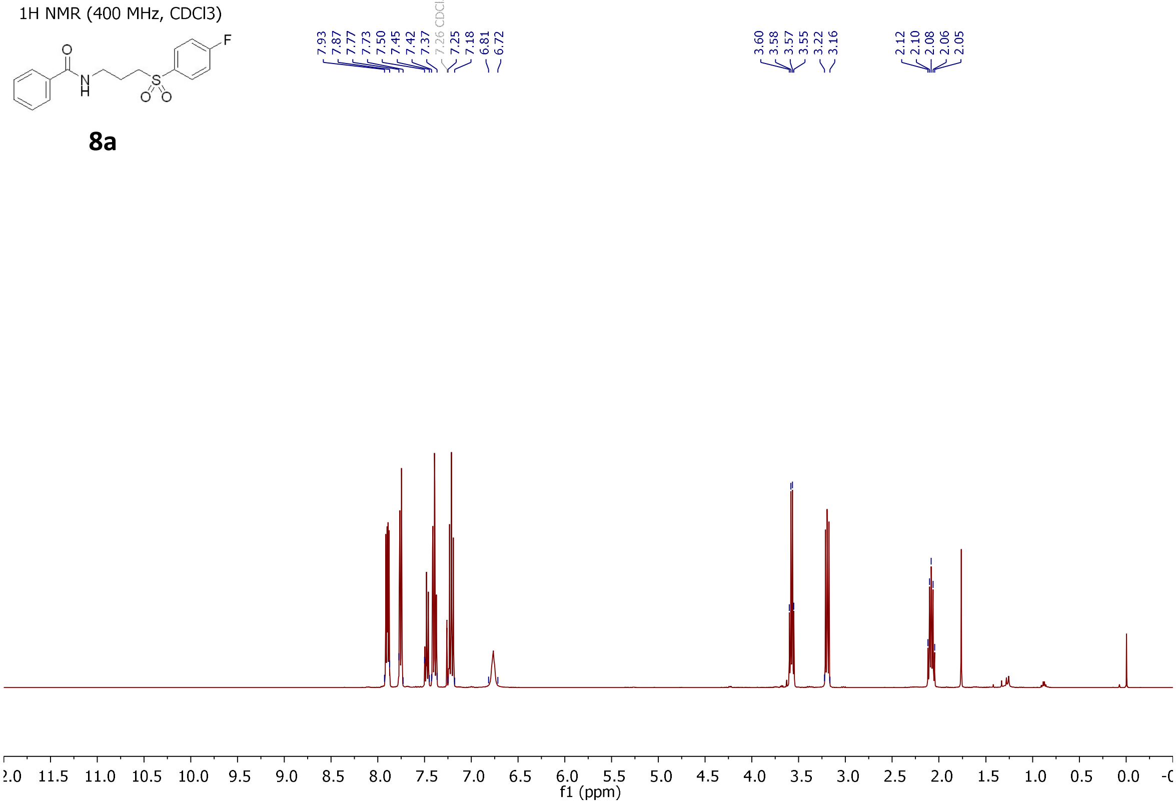

13C NMR (101 MHz, CDCl3)

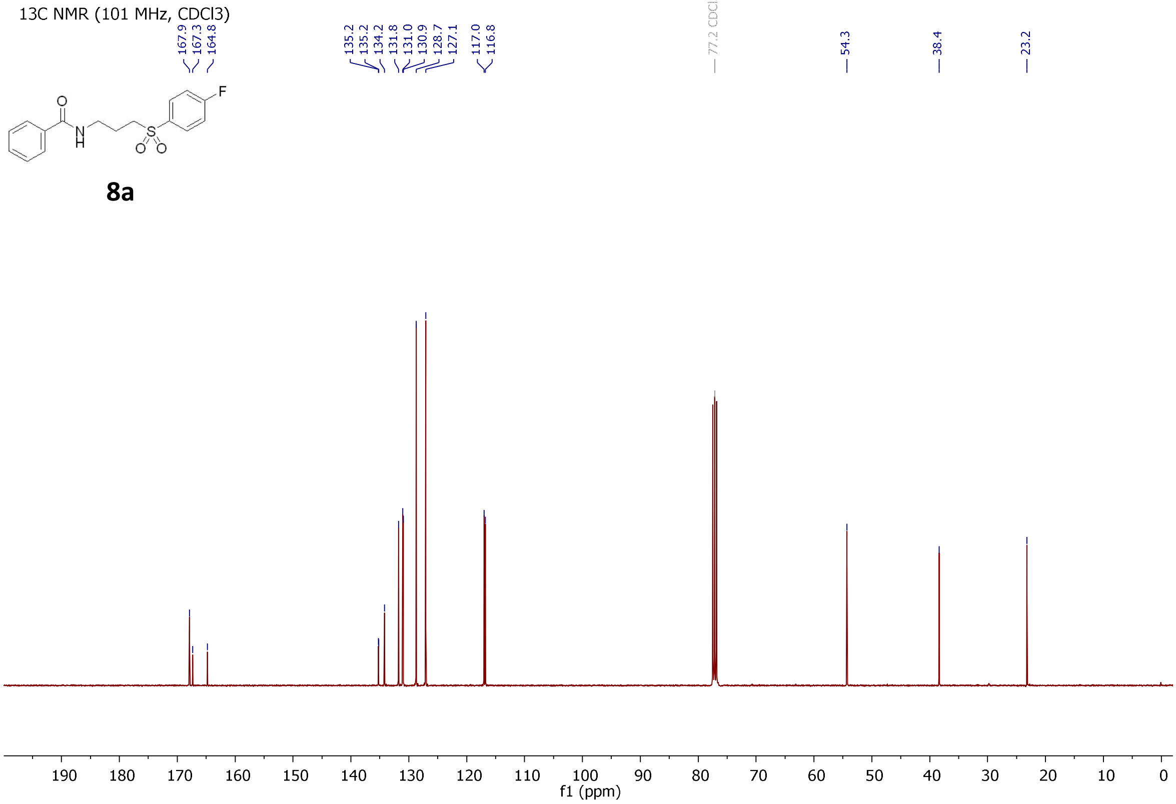

19F NMR (377 MHz, CDCl3)

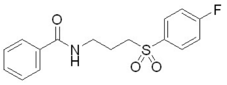

8a

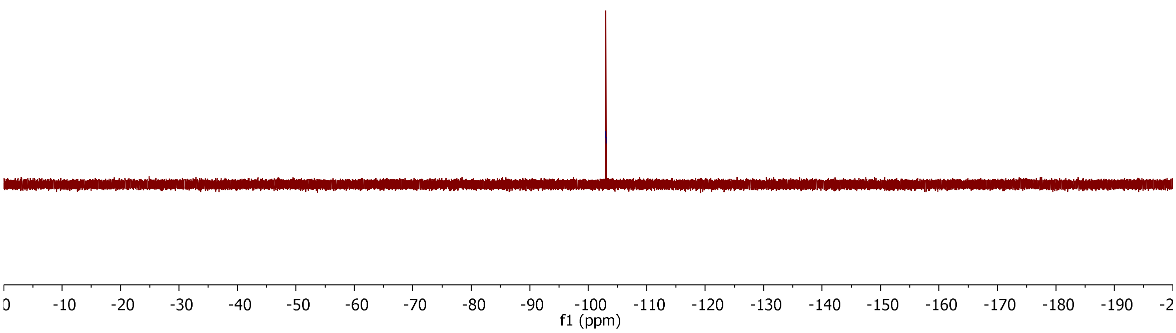

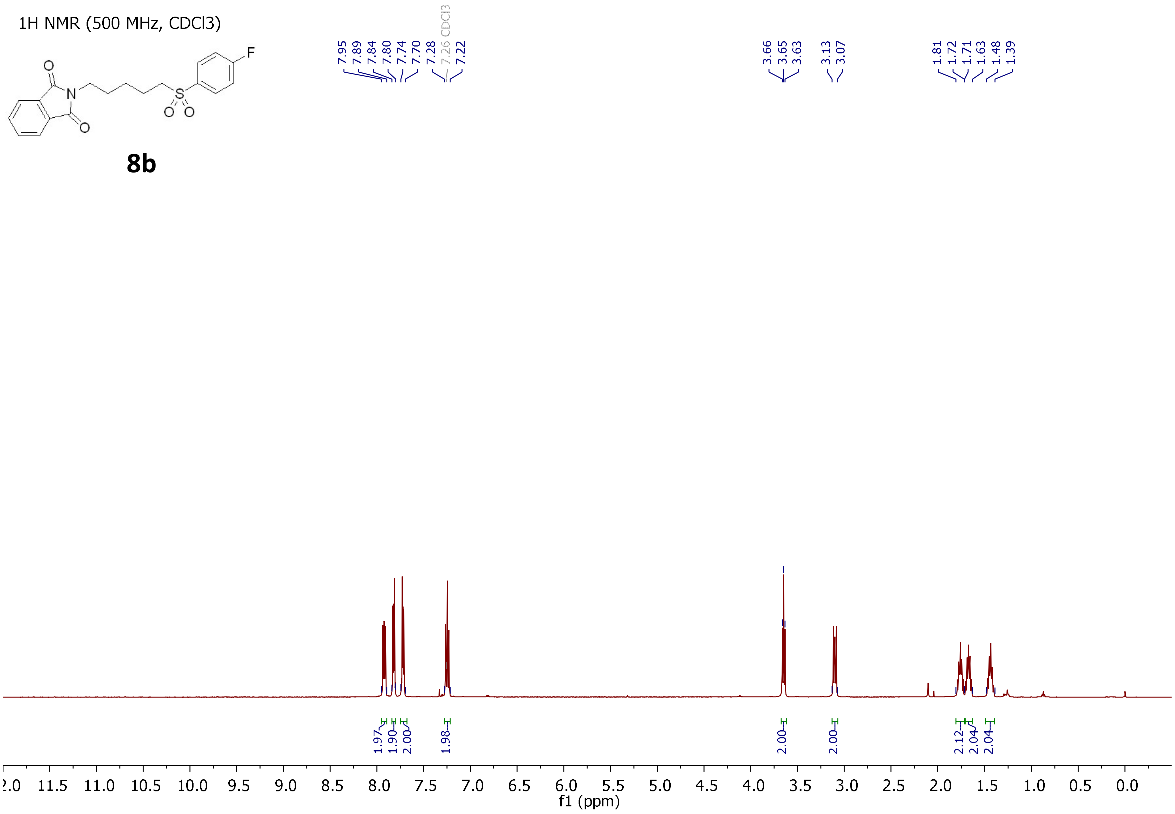
1H NMR (500 MHz, CDCl3)
F
O
N
S
O O
O
7.95
7.89
7.84
7.80
7.74
7.70
7.28
7.26 CDCl3
7.22
3.66
3.65
3.63
3.13
3.07
1.81
1.72
1.71
1.63
1.48
1.39
1.97
1.90
2.00
1.98
2.00
2.00
2.12
2.04
2.04
2.0 11.5 11.0 10.5 10.0 9.5 9.0 8.5 8.0 7.5 7.0 6.5 6.0 5.5 5.0 4.5 4.0 3.5 3.0 2.5 2.0 1.5 1.0 0.5 0.0
f1 (ppm)

8b

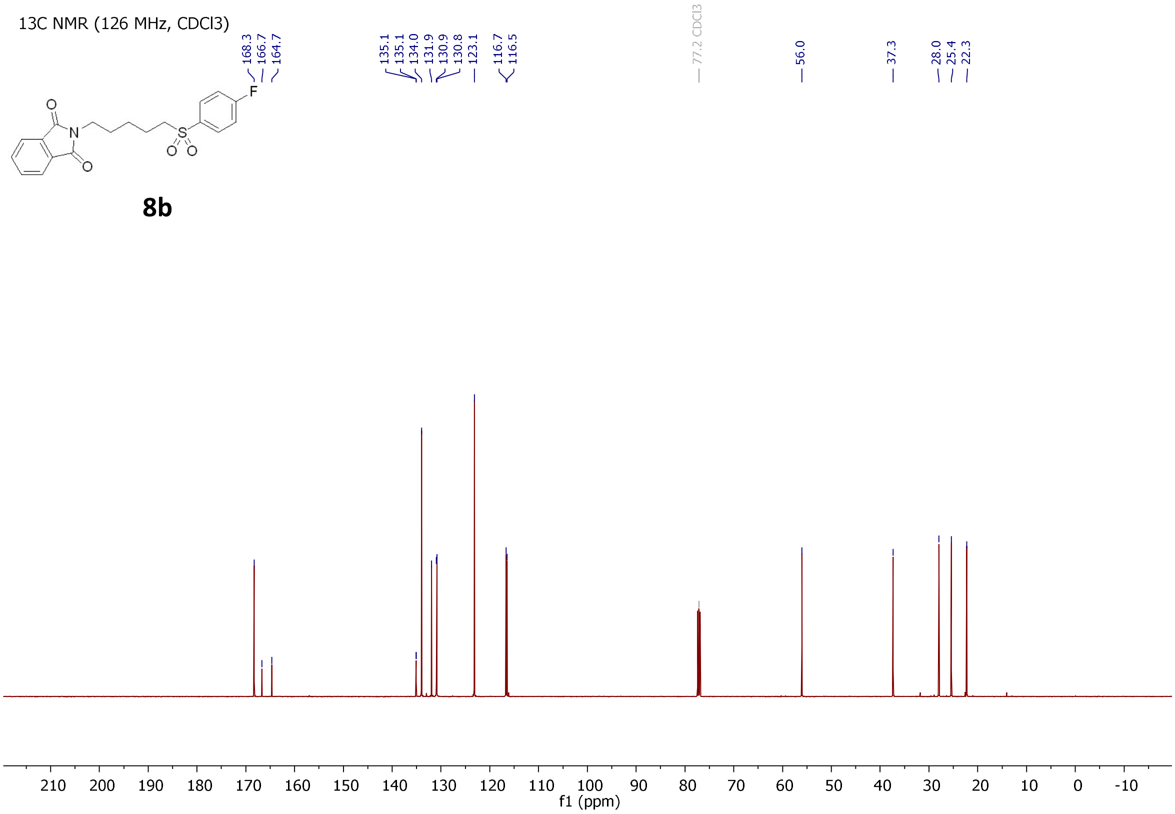
13C NMR (126 MHz, CDCl3)
F
O
N
S
O O
O
168.3
166.7
164.7
135.1
135.1
134.0
131.9
130.9
130.8
123.1
116.7
116.5
77.2 CDCl3
56.0
37.3
28.0
25.4
22.3
210 200 190 180 170 160 150 140 130 120 110 100 90 80 70 60 50 40 30 20 10 0 -10
f1 (ppm)

8b

19F NMR (471 MHz, CDCl3)

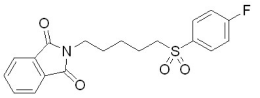

8b

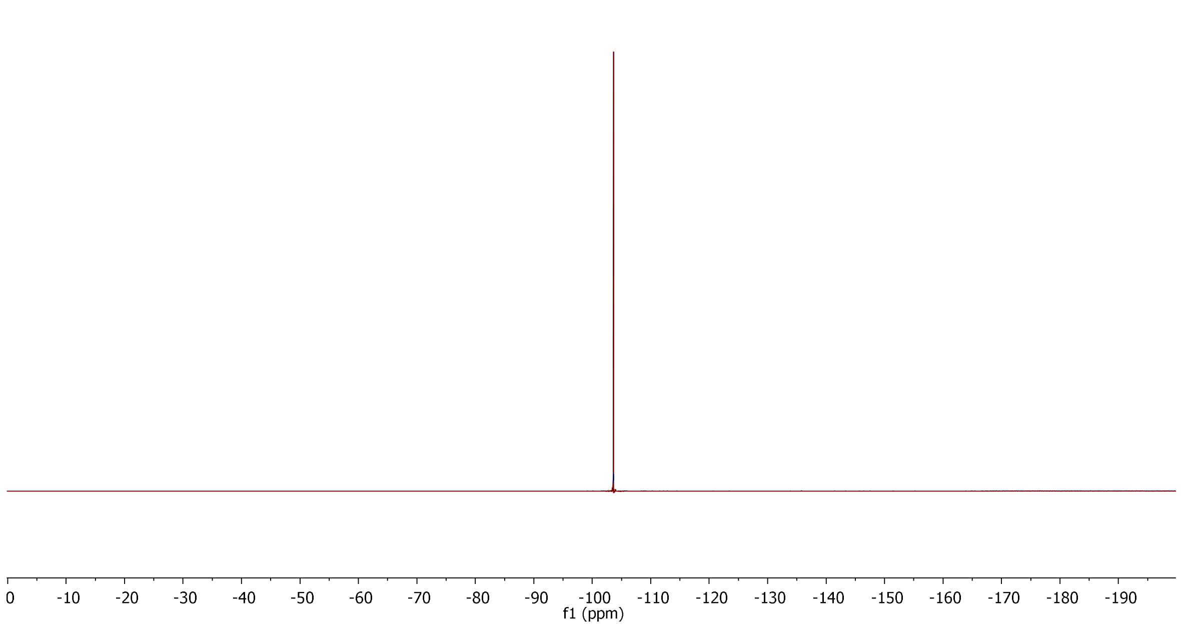

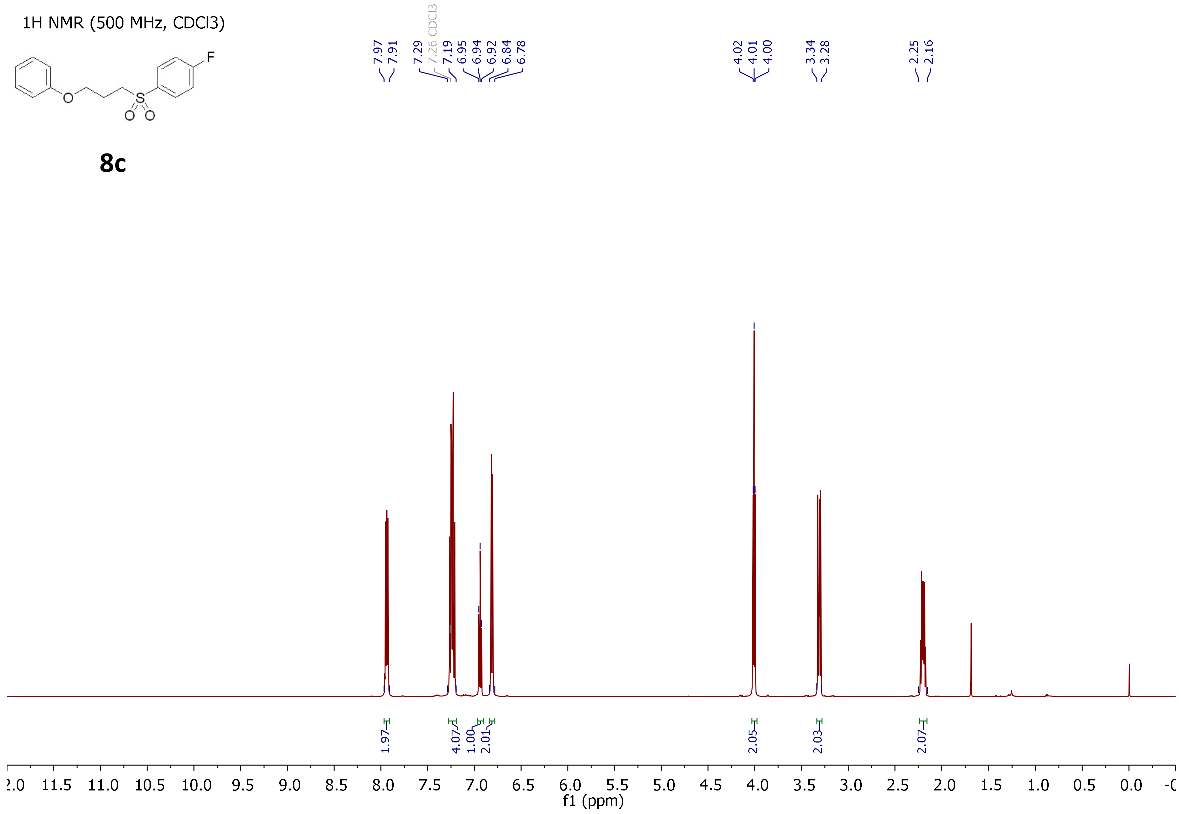
1H NMR (500 MHz, CDCl3)
F
O
S
O O
8c
7.97
7.91
7.29
7.26 CDCl3
7.19
6.95
6.94
6.92
6.84
6.78
4.02
4.01
4.00
3.34
3.28
2.25
2.16
1.97
4.07
1.00
2.01
2.05
2.03
2.07
f1 (ppm)

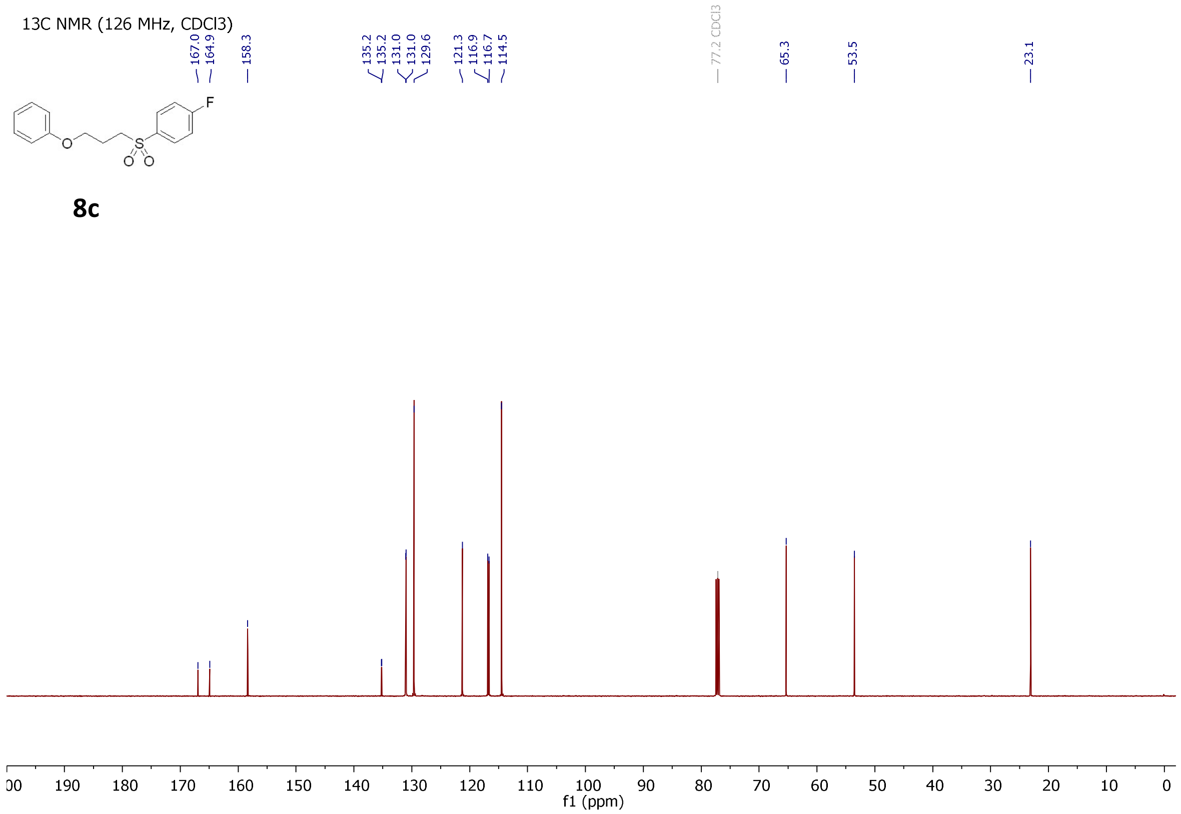
13C NMR (126 MHz, CDCl3)
F
O
S
O O
8c
167.0
164.9
158.3
135.2
135.2
131.0
131.0
129.6
121.3
116.9
116.7
114.5
77.2 CDCl3
65.3
53.5
23.1
f1 (ppm)

19F NMR (471 MHz, CDCl3)

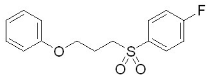

8c

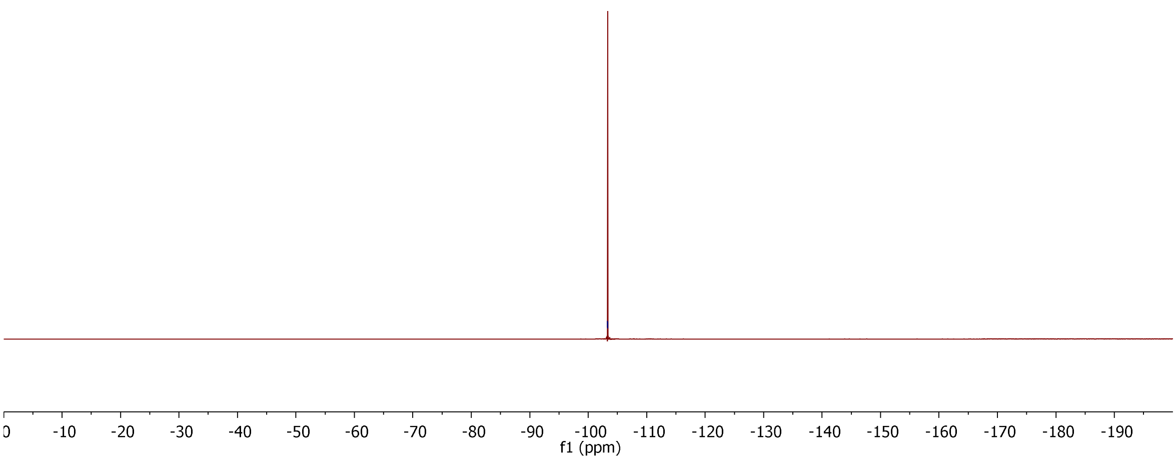

1H NMR (400 MHz, CDCl3)

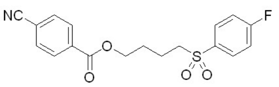

8d

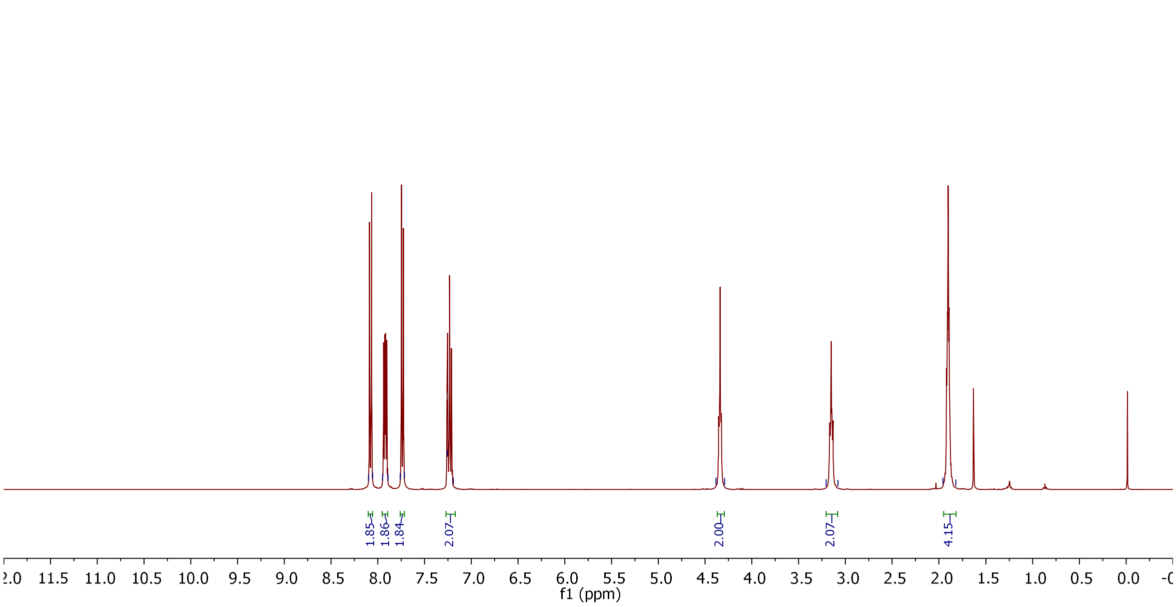

13C NMR (101 MHz, CDCl3)

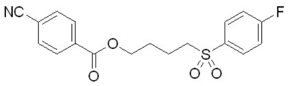

8c

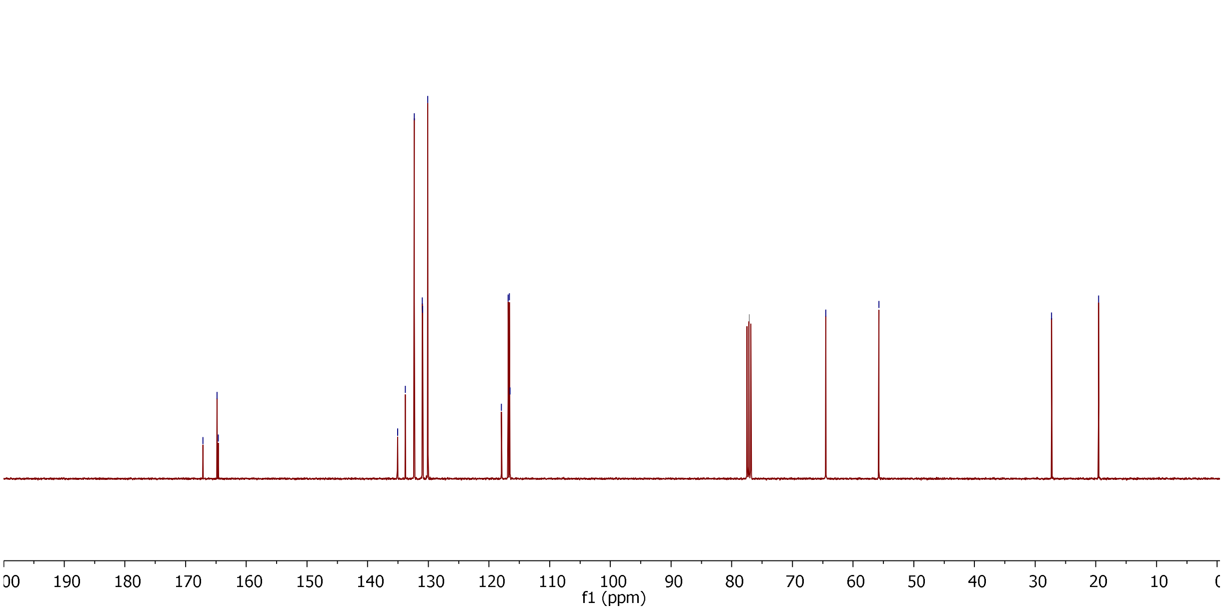

19F NMR (471 MHz, CDCl3)

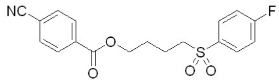

8c

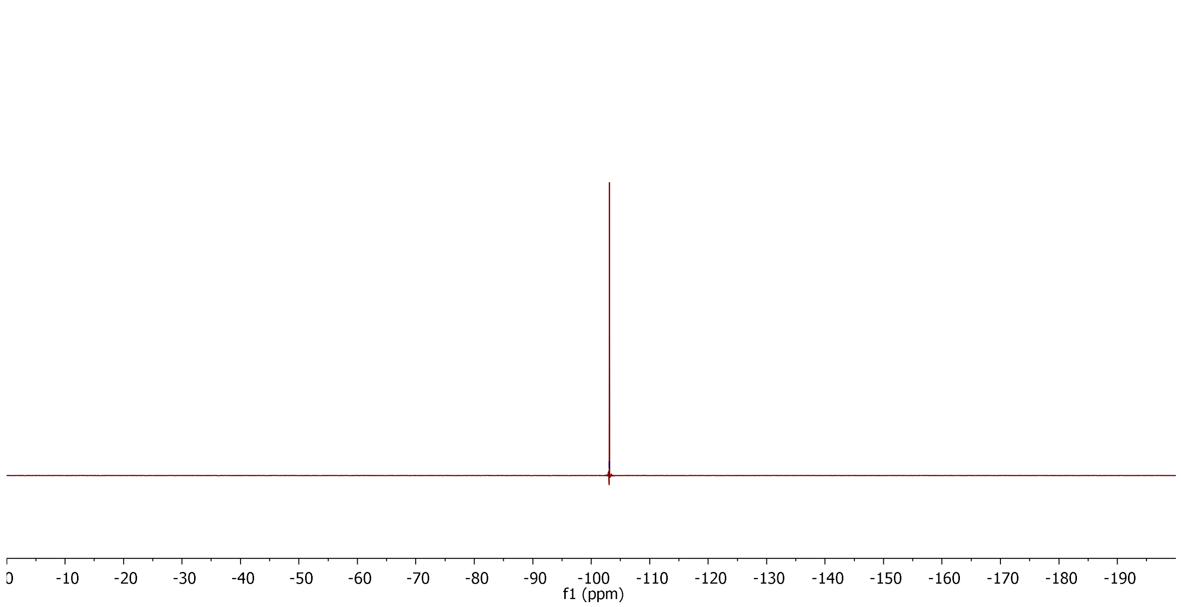

1H NMR (400 MHz, CDCl3)

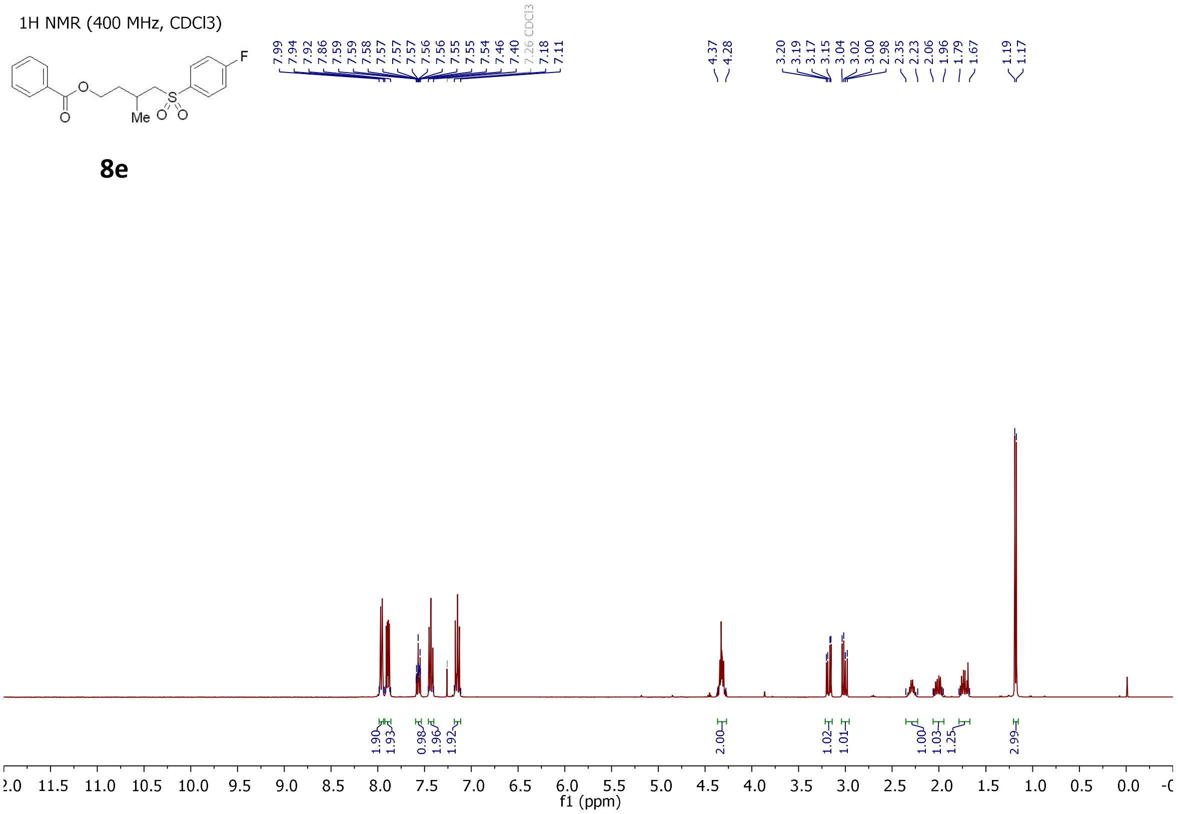

13C NMR (101 MHz, CDCl3)

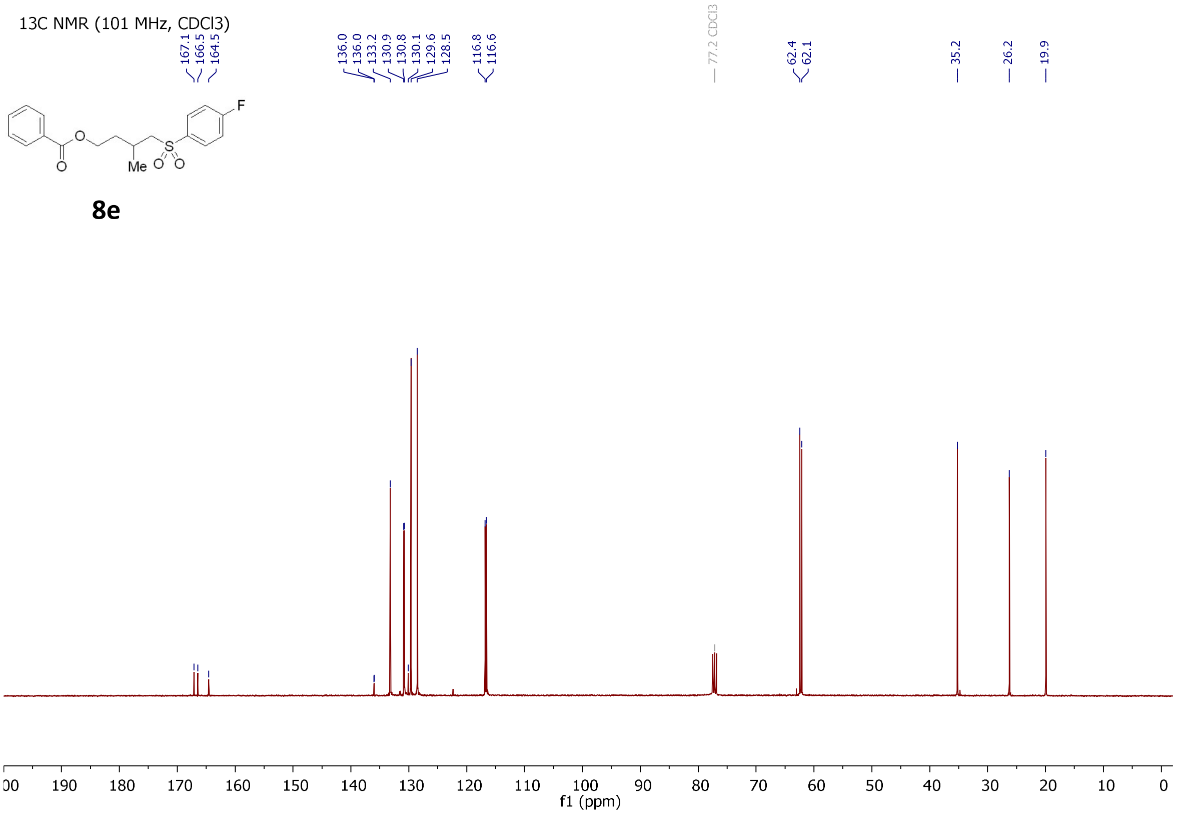

19F NMR (471 MHz, CDCl3)

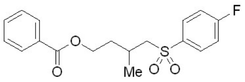

8e

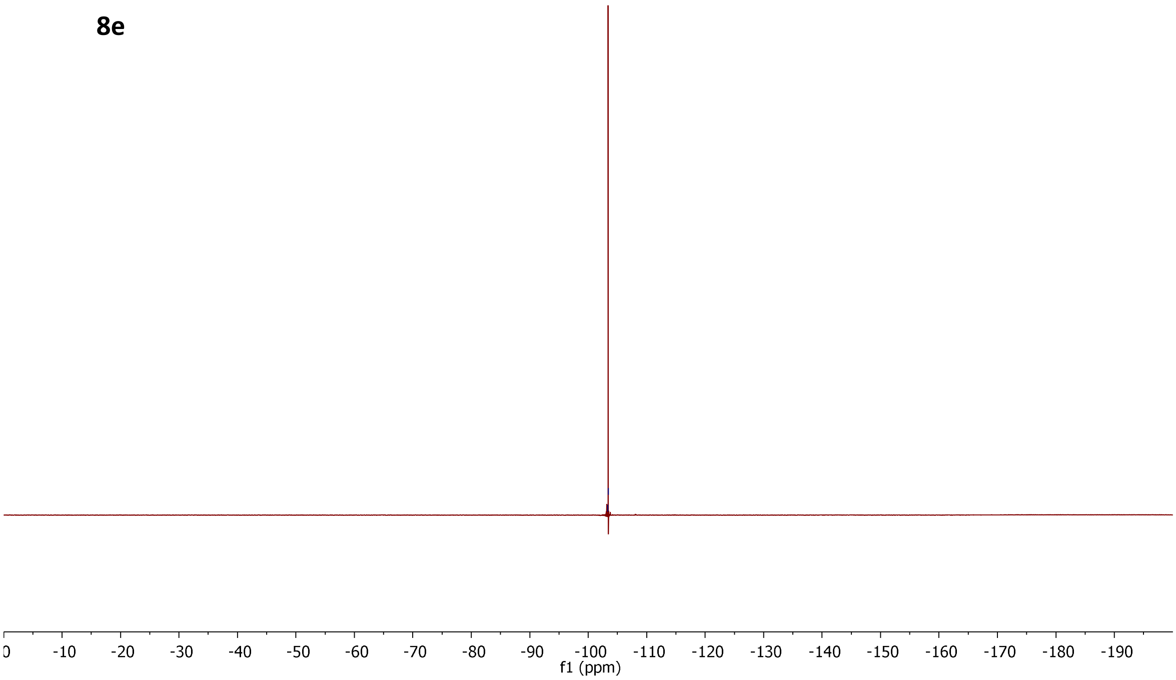

1H NMR (400 MHz, CDCl3)

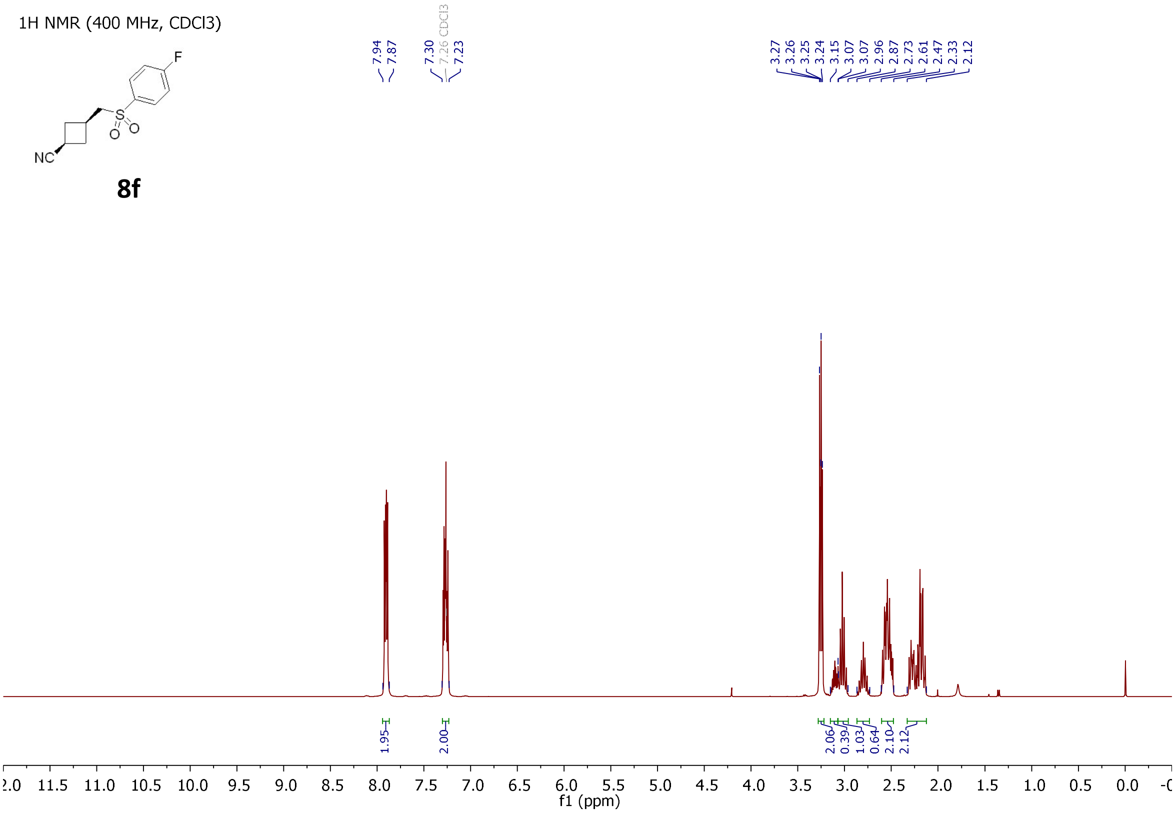

13C NMR (101 MHz, CDCl3)

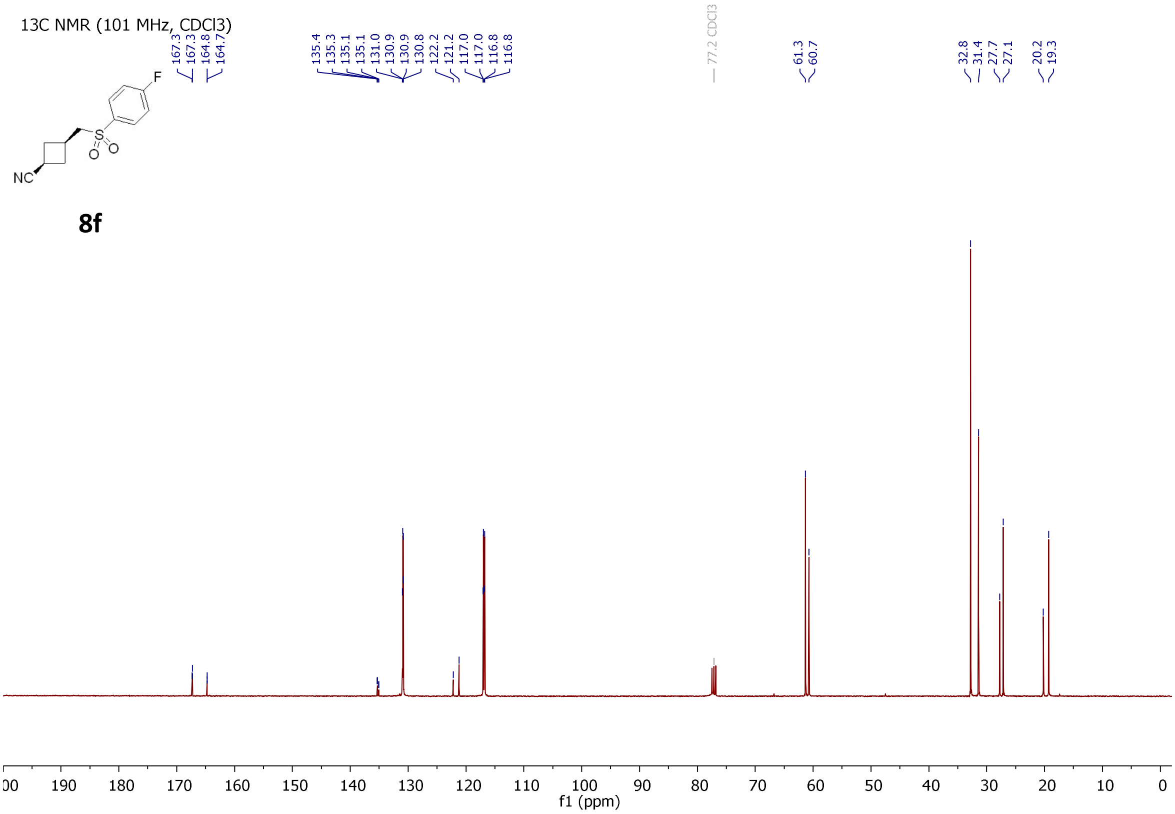

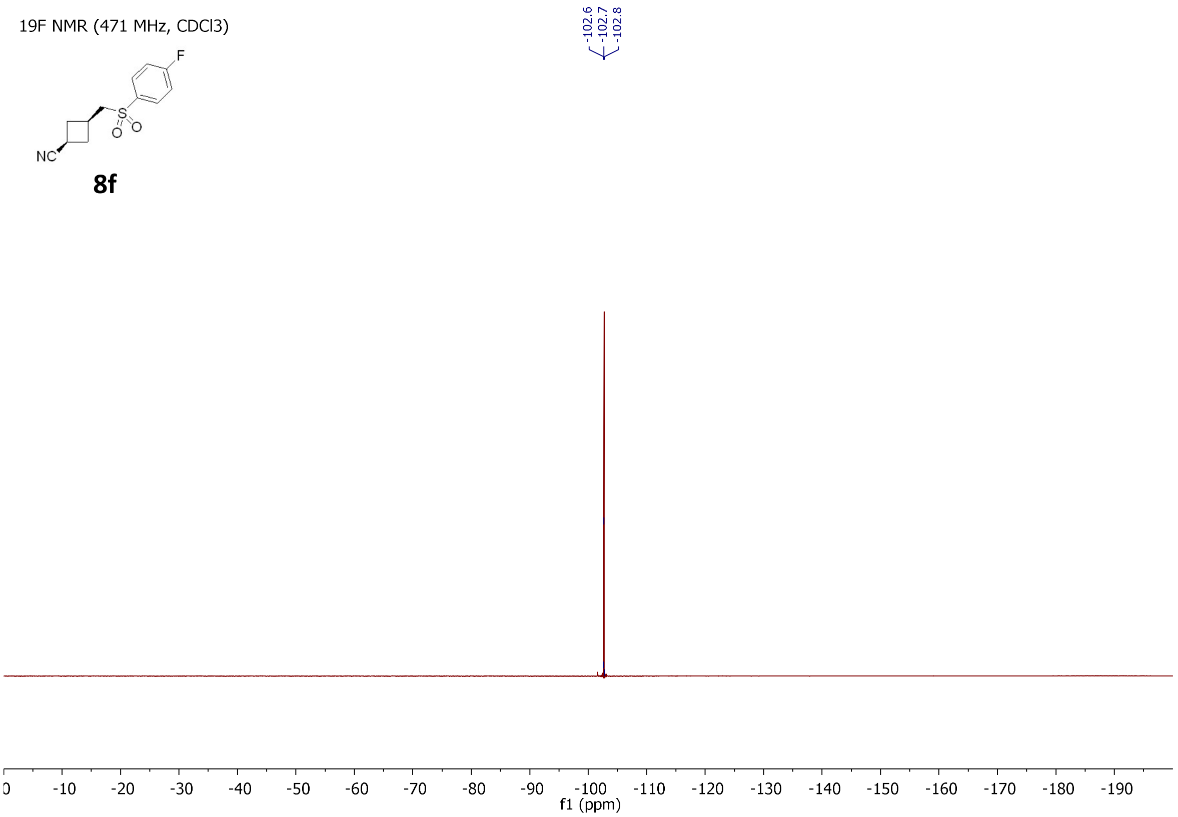
19F NMR (471 MHz, CDCl3)
F
S
O
O
NC
8f
-102.6
-102.7
-102.8
0
-10
-20
-30
-40
-50
-60
-70
-80
-90
-100
-110
-120
-130
-140
-150
-160
-170
-180
-190
f1 (ppm)

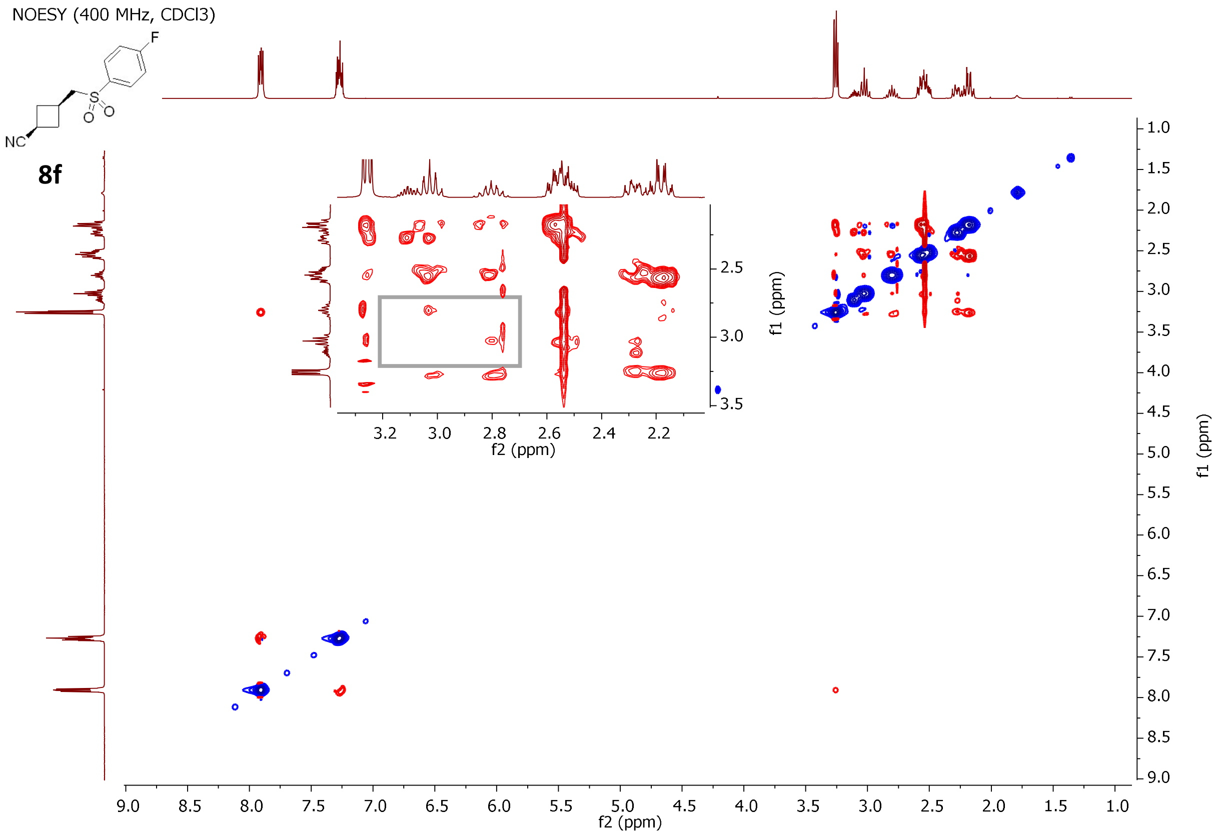
NOESY (400 MHz, CDCl3)
F
S
O
O
NC
8f
3.2
3.0
2.8
2.6
2.4
2.2
f2 (ppm)
2.5
3.0
3.5
f1 (ppm)
9.0
8.5
8.0
7.5
7.0
6.5
6.0
5.5
5.0
4.5
4.0
3.5
3.0
2.5
2.0
1.5
1.0
f2 (ppm)
f1 (ppm)

1H NMR (500 MHz, CDCl3)

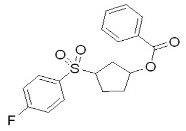

8g

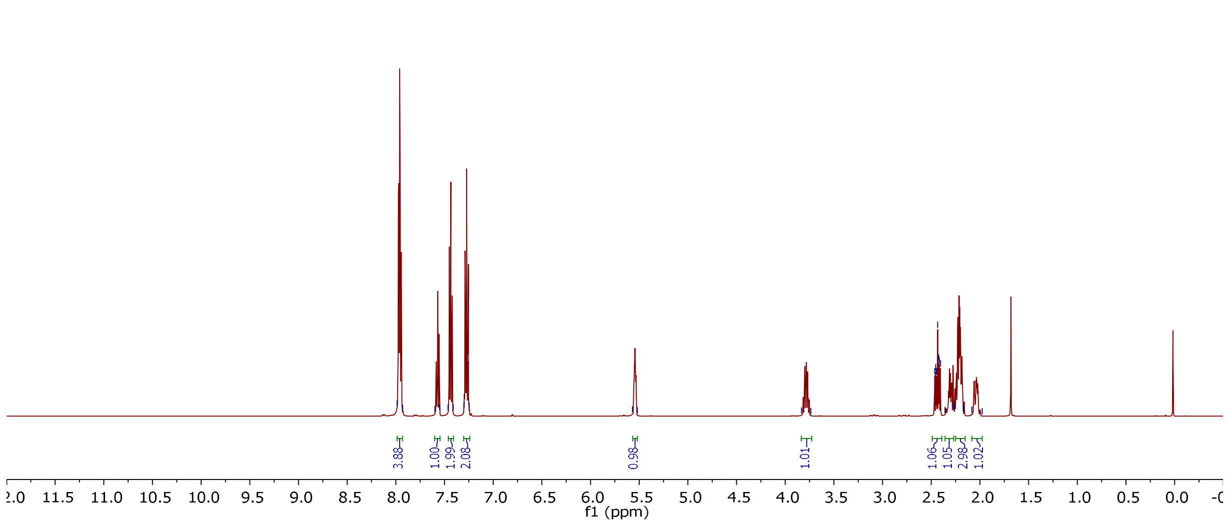

13C NMR (126 MHz, CDCl3)

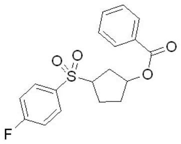

8g

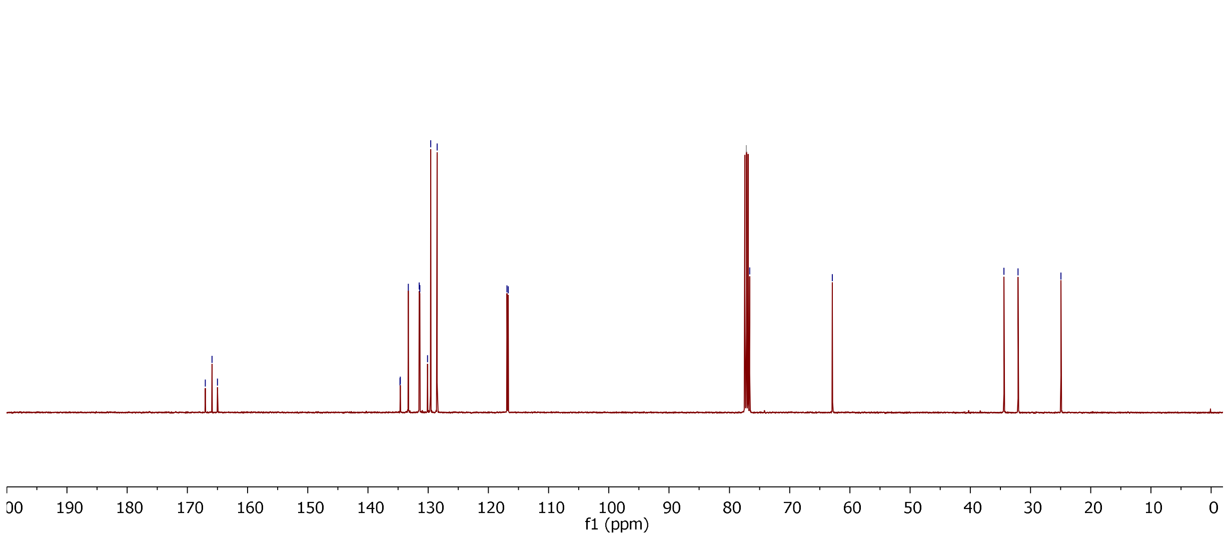

19F NMR (471 MHz, CDCl3)

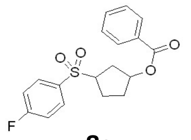

8g

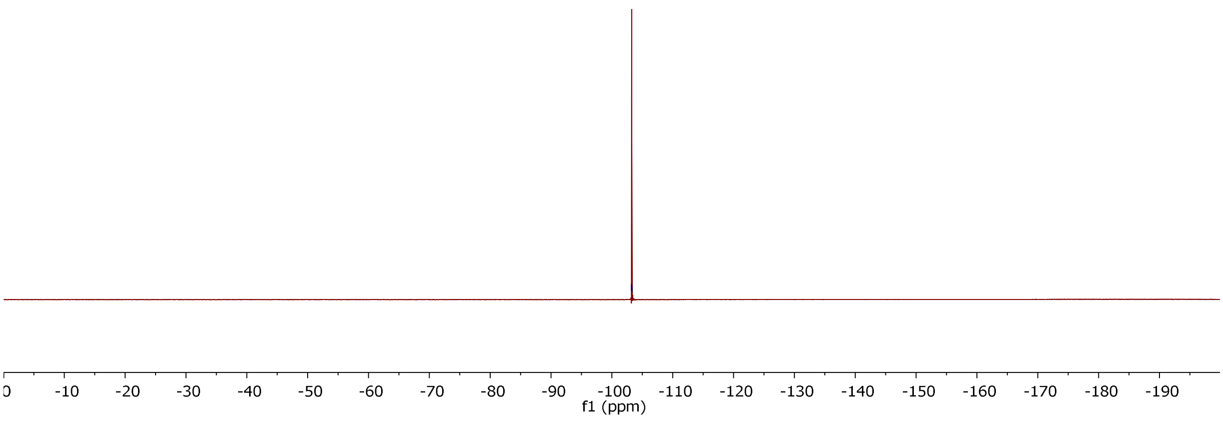

1H NMR (400 MHz, CDCl3)

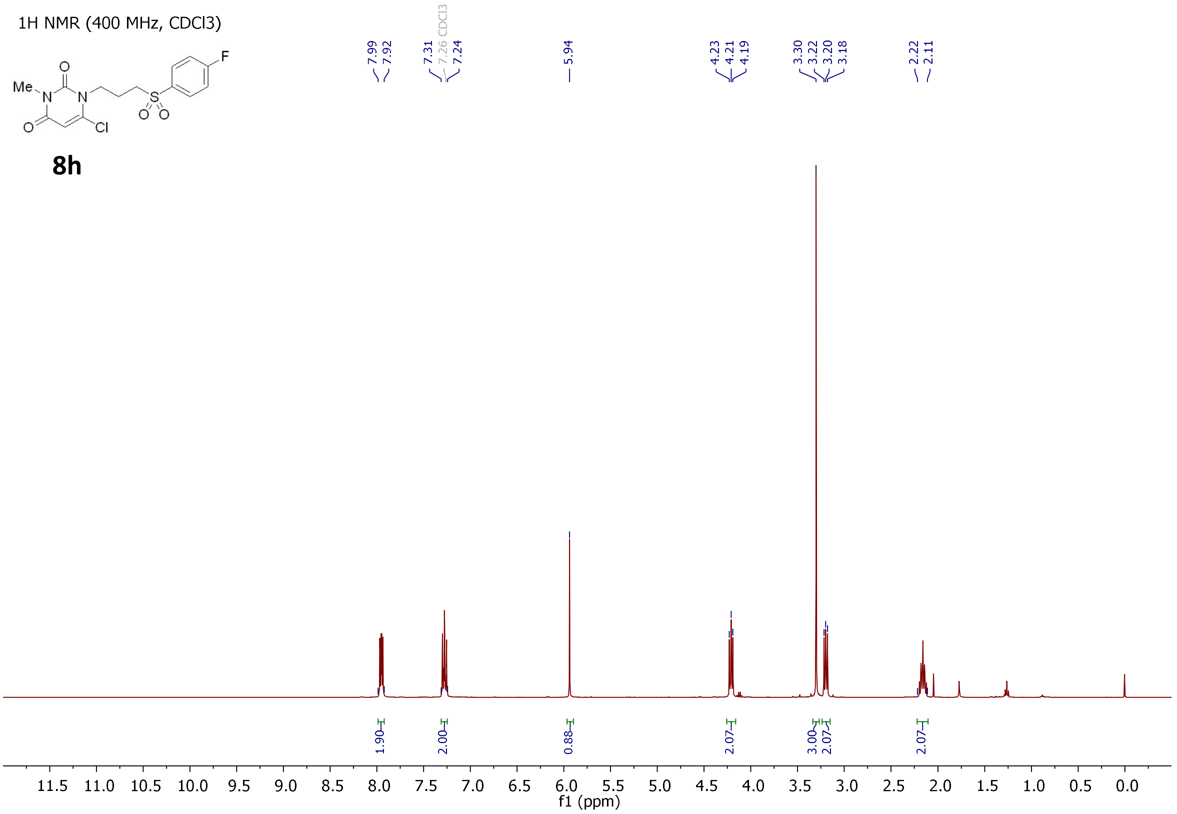

8h

13C NMR (101 MHz, CDCl3)

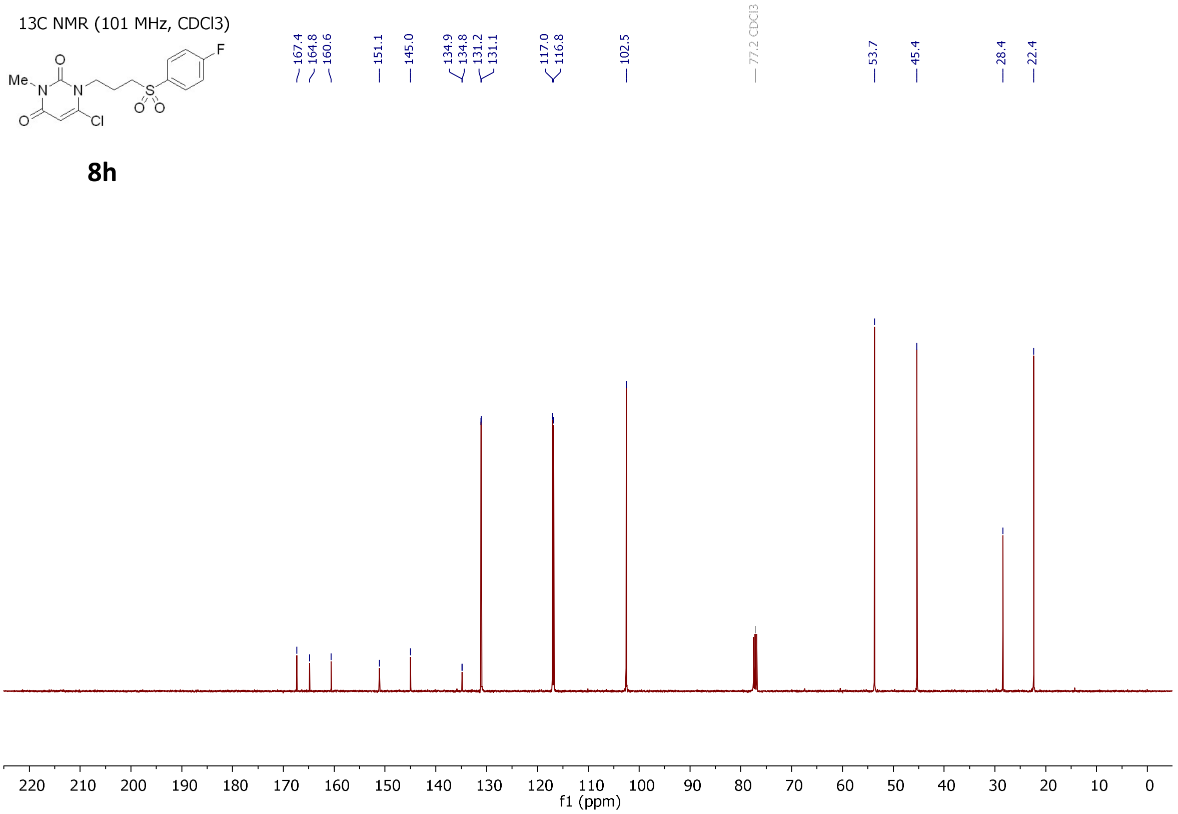

8h

19F NMR (471 MHz, CDCl3)

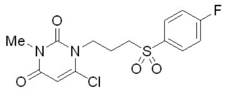

8h

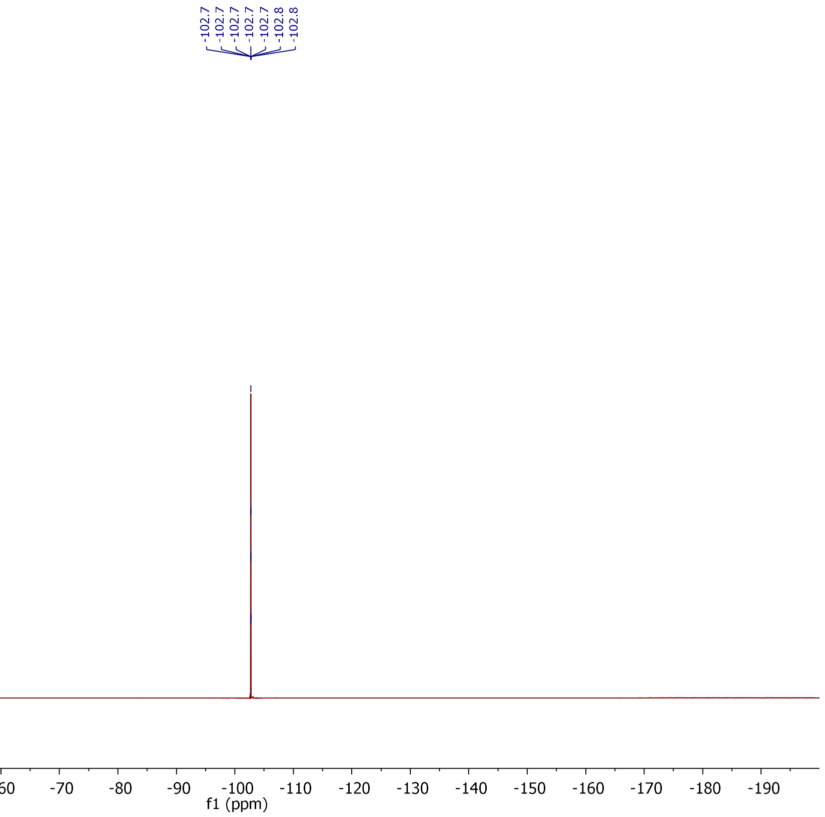

1H NMR (500 MHz, CDCl3)

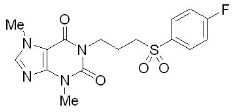

8i

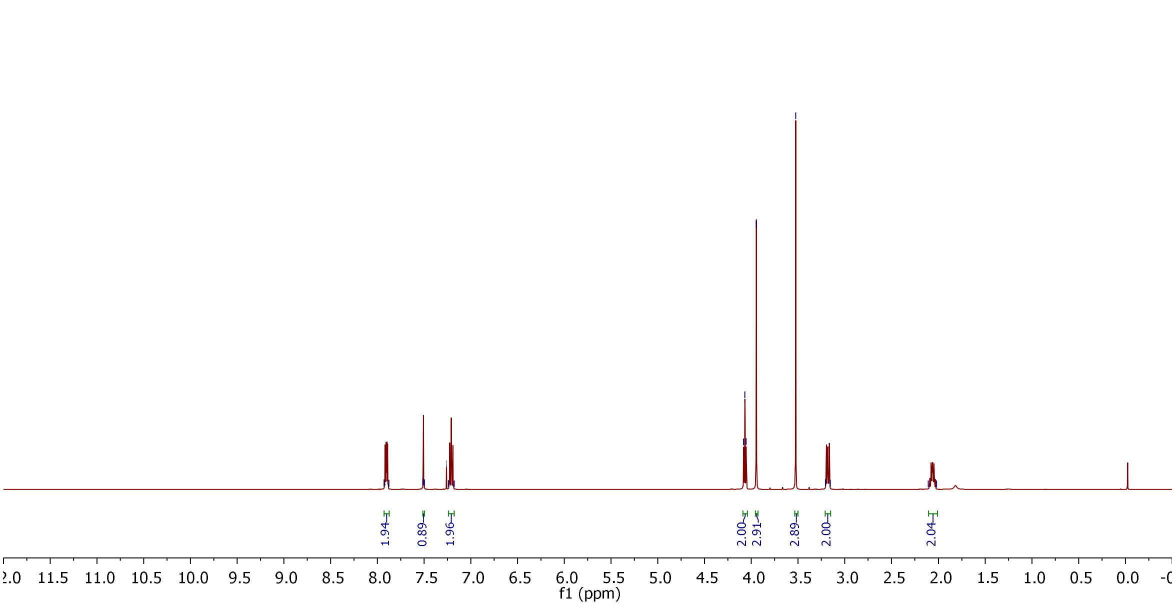

13C NMR (101 MHz, CDCl3)

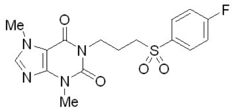

8i

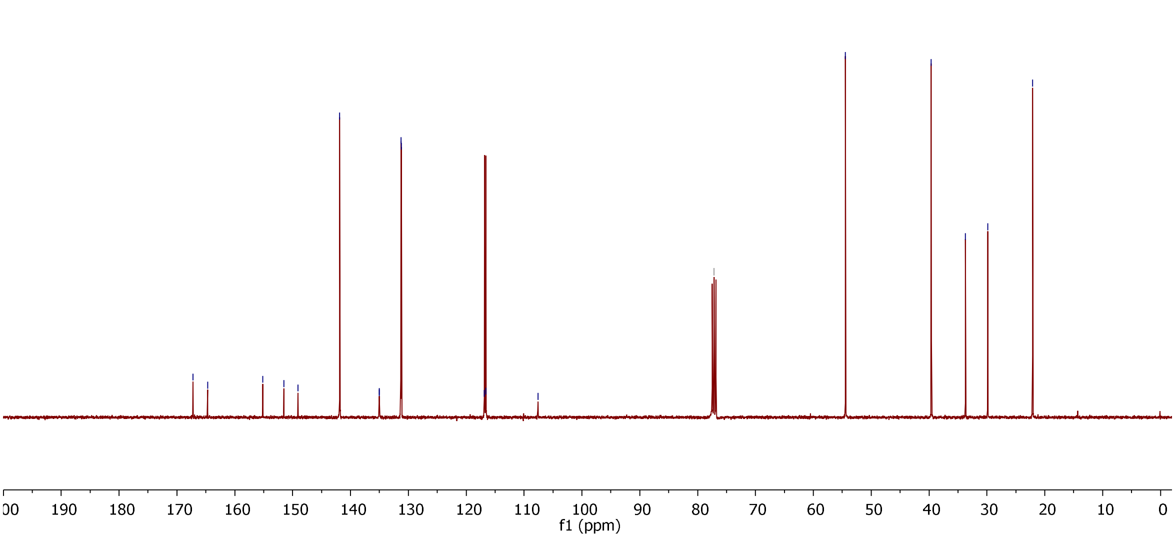

19F NMR (471 MHz, CDCl3)

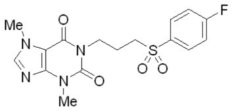

8i

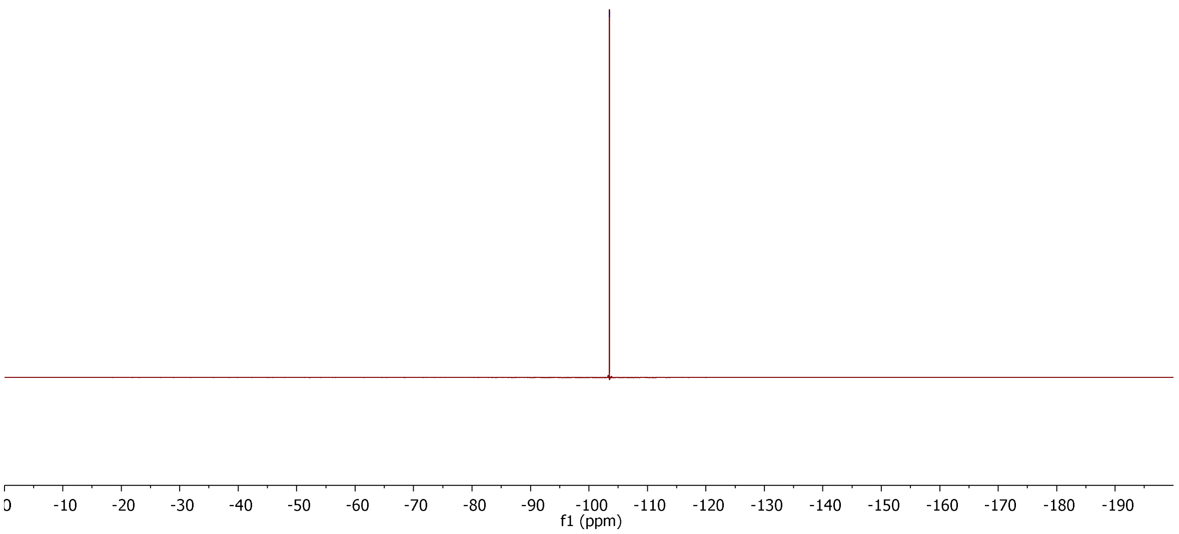

1H NMR (400 MHz, CDCl3)

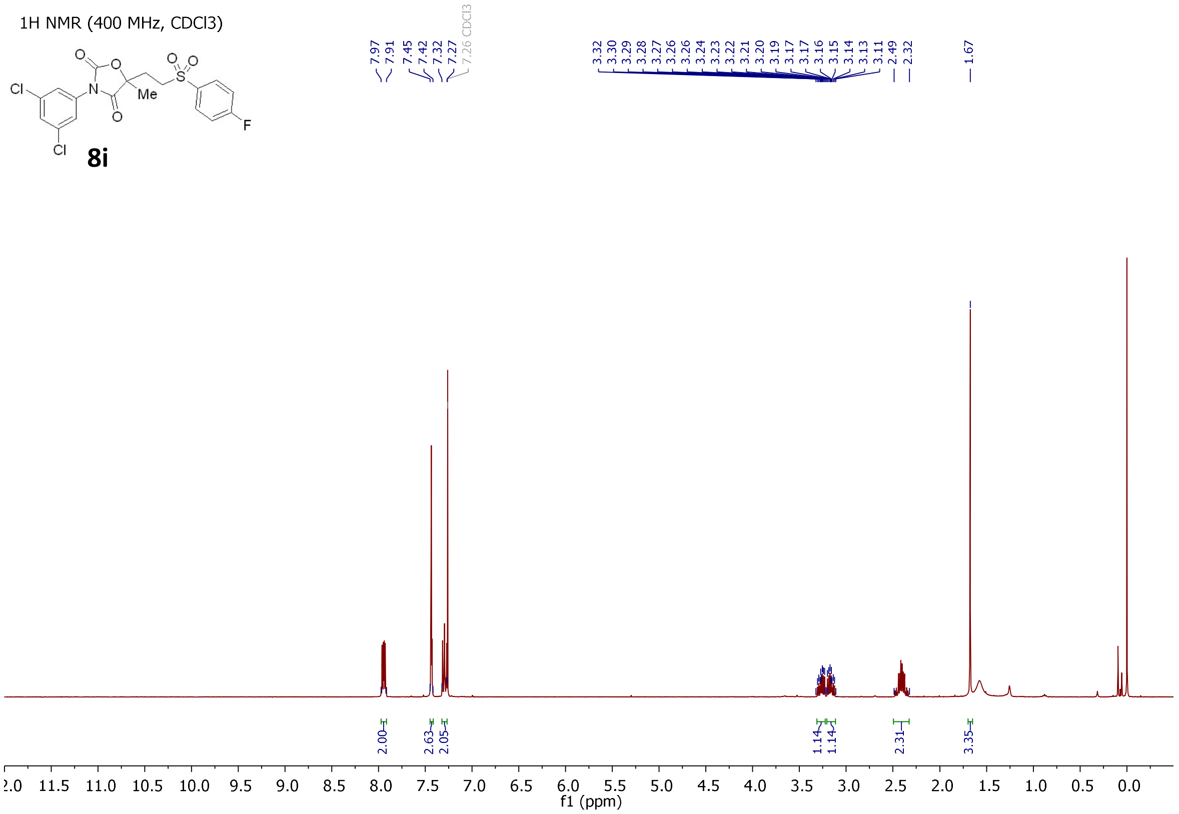

13C NMR (101 MHz, DMSO-d6)

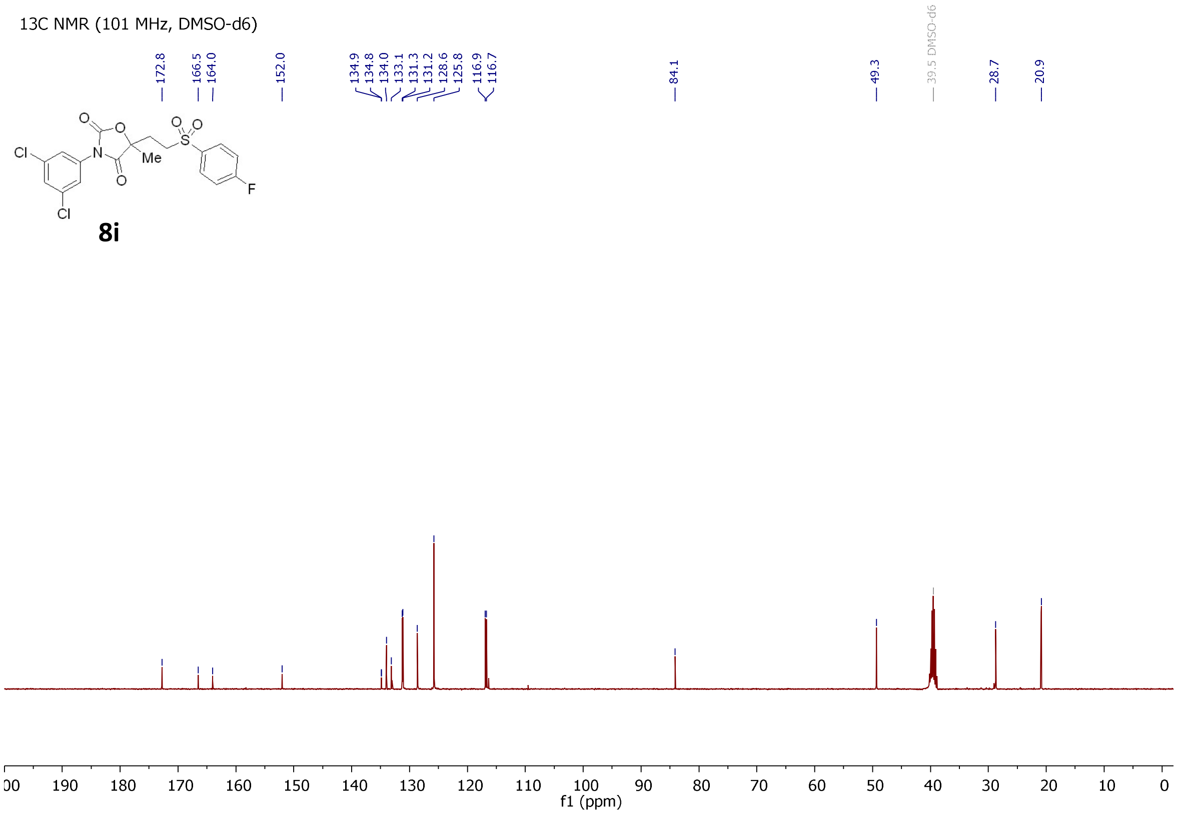

19F NMR (471 MHz, DMSO-d6)

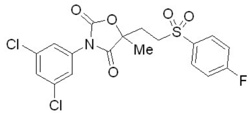

8i

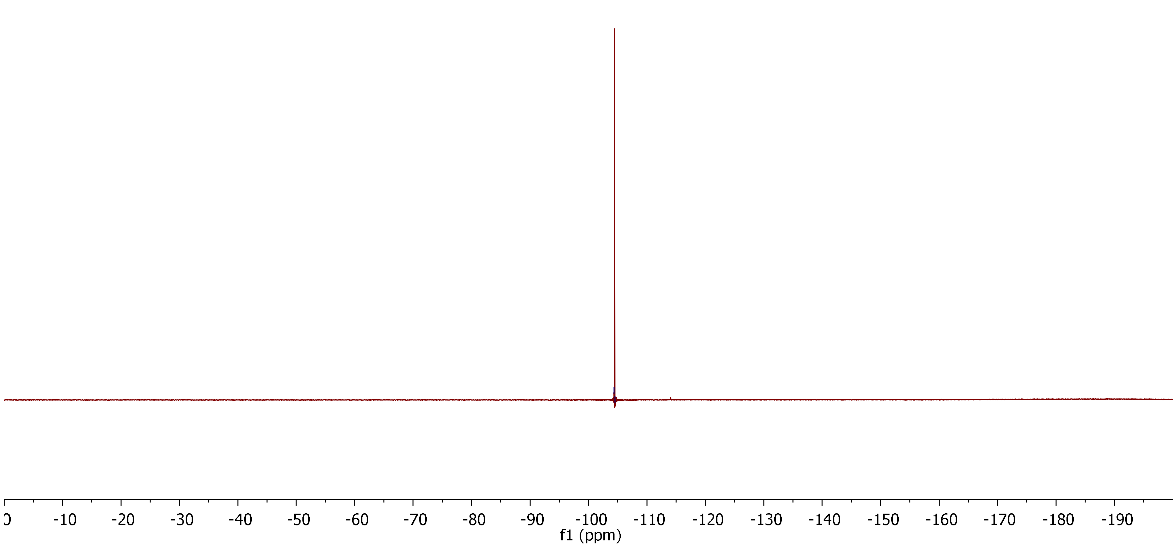

11. X-ray - Crystallographic Data for 6

Table S2. Crystal data and structure refinement for **6**.

Empirical formula	C17 H15 Cl F N O3 S	
Formula weight	367.83	
Temperature	150 K	
Wavelength	1.54184 Å	
Crystal system	Monoclinic	
Space group	P 21/n	
Unit cell dimensions	a = 6.8699(2) Å	α = 90°.
	b = 27.6557(7) Å	β = 101.073(2)°.
	c = 8.8183(2) Å	γ = 90°.
Volume	1644.22(7) $Å^3$	
Z	4	
Density (calculated)	1.486 Mg/m^3	
Absorption coefficient	3.486 mm^{-1}	
F(000)	760	
Crystal size	0.35 x 0.22 x 0.04 mm^3	
Theta range for data collection	5.356 to 76.157°.	
Index ranges	-5<=h<=8, -34<=k<=34, -11<=l<=10	
Reflections collected	11306	
Independent reflections	3403 [R(int) = 0.042]	
Completeness to theta = 74.634°	99.6 %	
Absorption correction	Semi-empirical from equivalents	
Max. and min. transmission	0.87 and 0.50	
Refinement method	Full-matrix least-squares on F^2	
Data / restraints / parameters	3403 / 0 / 217	
Goodness-of-fit on F^2	1.0142	
Final R indices [I>2sigma(I)]	R1 = 0.0396, wR2 = 0.0966	
R indices (all data)	R1 = 0.0490, wR2 = 0.1071	
Largest diff. peak and hole	0.51 and -0.35 e.$Å^{-3}$	

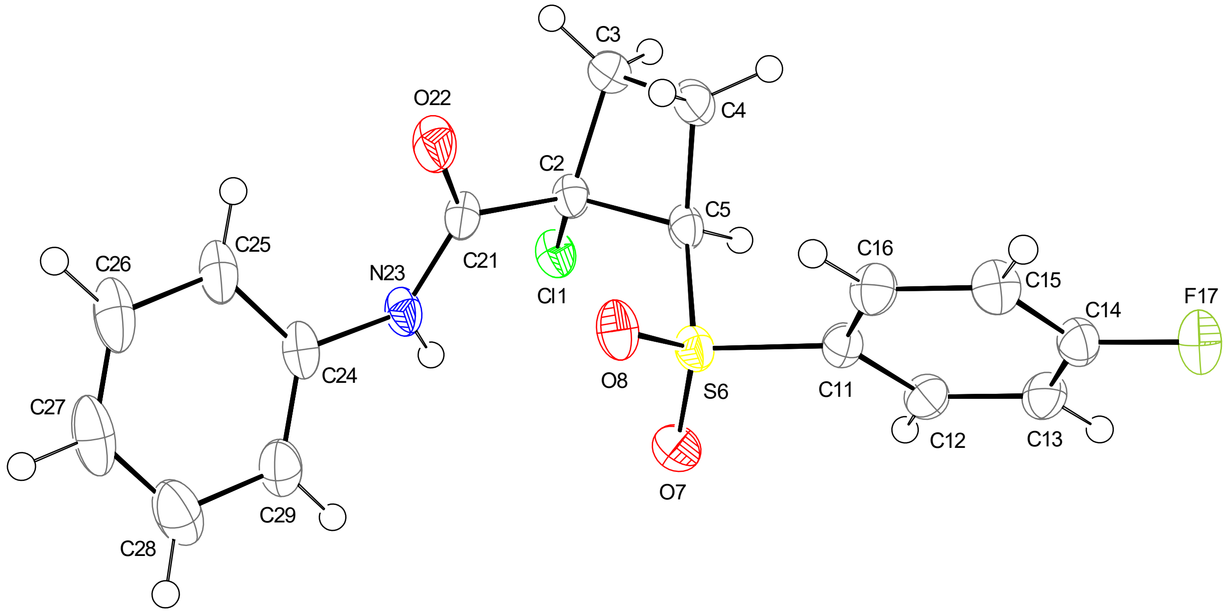
C3
O22
C4
C2
C5
C25
C16
C26
C21
N23
Cl1
C15
C14
F17
C24
O8
S6
C11
C27
C12
C13
O7
C29
C28
